# Simple sequence repeats in *Neurospora crassa*: distribution, polymorphism and evolutionary inference

**DOI:** 10.1186/1471-2164-9-31

**Published:** 2008-01-23

**Authors:** Tae-Sung Kim, James G Booth, Hugh G Gauch, Qi Sun, Jongsun Park, Yong-Hwan Lee, Kwangwon Lee

**Affiliations:** 1 Department of Plant Pathology and Plant-Microbe Biology, Cornell University, Ithaca, NY 14853 USA; 2 Department of Biological Statistics and Computational Biology, Cornell University, Ithaca, NY 14853 USA; 3 Department of Crop and Soil Sciences, Cornell University, Ithaca, NY 14853 USA; 4 Computational Biology Service Unit, Cornell University, Ithaca NY 14853 USA; 5 Department of Agricultural Biotechnology and Center for Fungal Genetic Resources, Seoul National University, Seoul 151-921, Republic of Korea

## Abstract

**Background:**

Simple sequence repeats (SSRs) have been successfully used for various genetic and evolutionary studies in eukaryotic systems. The eukaryotic model organism *Neurospora crassa *is an excellent system to study evolution and biological function of SSRs.

**Results:**

We identified and characterized 2749 SSRs of 963 SSR types in the genome of *N. crassa*. The distribution of tri-nucleotide (nt) SSRs, the most common SSRs in *N. crassa*, was significantly biased in exons. We further characterized the distribution of 19 abundant SSR types (AST), which account for 71% of total SSRs in the *N. crassa *genome, using a Poisson log-linear model. We also characterized the size variation of SSRs among natural accessions using Polymorphic Index Content (PIC) and ANOVA analyses and found that there are genome-wide, chromosome-dependent and local-specific variations. Using polymorphic SSRs, we have built linkage maps from three line-cross populations.

**Conclusion:**

Taking our computational, statistical and experimental data together, we conclude that 1) the distributions of the SSRs in the sequenced N. crassa genome differ systematically between chromosomes as well as between SSR types, 2) the size variation of tri-nt SSRs in exons might be an important mechanism in generating functional variation of proteins in *N. crassa*, 3) there are different levels of evolutionary forces in variation of amino acid repeats, and 4) SSRs are stable molecular markers for genetic studies in *N. crassa*.

## Background

Simple sequence repeats (SSRs) refer to the sequences that are one to six-nucleotides (nt) repeated in tandem in a genome. SSRs have many advantageous features for various biological studies: SSRs are ubiquitous and abundant in a genome, highly variable and suitable for high-throughput applications [1-8]. In addition to practical usages of SSRs for biological studies, the SSRs have also been under the intense scrutiny of researchers to elucidate the evolution of genomes: (1) why are they ubiquitously present in a genome, (2) how do they arise, (3) why are they are unusually polymorphic, and (4) what are their biological or structural functions are [1,9]? The evolutionary dynamics of SSRs have been actively discussed and hypotheses for experimental confirmation have been reviewed in the recent literature [1,9-11].

The growing numbers of completed genome sequences in eukaryotic organisms from fungi to human have greatly assisted understanding SSRs at the genome-wide level. One obvious observation from the genome-wide studies was that the distribution of SSRs in the genome was not random in several respects: tri-nt and hexa-nt SSRs in coding regions were the dominant SSR types; other SSR repeat types (except tri-nt or hexa-nt SSRs) were found in excess in the non-coding regions of the genome but were rare in coding regions; differential distribution in terms of abundance of SSRs was observed in between intronic and intergenic regions 5' and 3' UTRs, and different chromosomes; and lastly, different species have different frequencies of SSR types and repeat units [2,10,12,13]. The current experimental and observational evidence suggests that these non-random distributions of SSRs, both in coding and non-coding regions, may be associated with a functional significance, which presumably results in adaptive advantages [9,14-20]. Two alternative hypotheses were suggested to explain the genesis of SSRs. These hypotheses propose that SSRs originate either spontaneously from/within unique sequences (*de novo *genesis) or that they are brought about in a primal form into a receptive genomic location by mobile elements (adoptive genesis). These two hypotheses are both adequate for explaining the ubiquitous distribution of SSRs. However, there remains much to be understood to elucidate which one is right and how the non-random distribution of SSRs has emerged in the eukaryotic genome [1,9-11]. *N. crassa *has been well characterized for its diverse genome defence mechanisms that inactivate genetic mobile elements and gene duplication across the genome except in some restricted regions close to telomeres and centromeres [21-24]. Thus, we reasoned that characterizing the SSR distribution in the *N. crassa *genome would provide a unique opportunity to explore the non-random distribution of SSRs shaped by the *de novo *genesis in the eukaryotic genome.

In this report, we investigate the distribution and size variability of SSRs across the *N. crassa *genome. We had four specific questions in mind. 1) Is the distribution of SSRs random or not in the *N. crassa *genome? If it is not random, what factors could explain this? 2) What are the biological functions of SSRs? 3) What are the forces causing the size variation of SSRs? 4) Could we use SSRs for population studies in intra-species populations as previously suggested [8]?

Our data on the distribution and size variation of SSRs in the *N. crassa *genome reveal both similarities and uniqueness in composition and distribution patterns in comparison to the other eukaryotic genomes, including other sequenced fungal organisms. We discuss the potential forces for shaping non-random distribution and size variation of SSRs, and biological implications of size variations of SSRs in the *N. crassa *genome.

## Results

### Genome-wide distribution of SSRs by the SSR unit size

**Figure 1:**
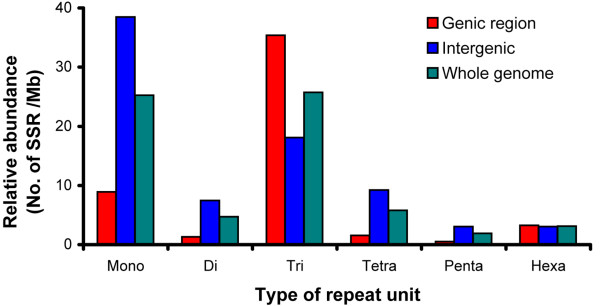
**Genome-wide distribution of relative abundance of SSRs by the unit size**. The relative abundance was calculated by the number of SSR type/mega base-pair (MB). Each bar represents the relative abundance of SSR type in different genome locations; genic (red bar), intergenic (blue bar), and whole genome (green bar), which was calculated by (genic + intergenic)/2. The x-axis represents SSRs that have different SSR units and the y-axis represents the relative abundance of each SSR type.

In order to systematically characterize the distribution of SSRs, we surveyed all of the SSRs in the *N. crassa *genome. With our filter conditions (Methods), we identified 2749 SSRs (Additional File 1). SSRs were present equally in the genic and intergenic regions in the *N. crassa *genome; 51% in the genic region and 49% in the intergenic region (Fig. 1 and Table 1). Tri-nt SSRs were the most abundant SSRs overall (Fig. 1 and Table 1). SSRs in different repeat units show differential or non-random distributions in the different genomic locations. It is noteworthy that tri-nt SSRs were the most abundant SSR type in the genic region, whereas, mono-nt SSRs were the most abundant SSR type in the intergenic region (Fig. 1). In an attempt to analyze the differential distribution of SSRs more clearly, we characterized the distribution of SSR types in each repeat unit across genomic locations.

**Table 1:** Relative abundance of SSR types by functional genome regions in *N. crassa*.

	Intergenic region (21.7 Mb)		Genic region (17.4 Mb)				Total (39.2 Mb)	
					
			Exon (14.7 Mb)		Intron (2.7 Mb)			
SSR types	Count	RA^a^	Count	RA^a^	Count	RA^a^	Count	RA^a^

Mono	835	38.4	3	0.2	152	56.3	990	25.3
Di	167	7.7	1	0.1	22	8.1	191	4.9
Tri	414	19.1	591	40.2	64	23.7	1084	27.7
Tetra	213	9.8	1	0.1	22	8.1	243	6.2
Penta	73	3.4	1	0.1	7	2.6	82	2.1
Hexa	84	3.9	64	4.4	7	2.6	159	4.1

^a ^Relative abundance = number of SSR/chromosome size in mega base (MB)

### Mono-nt SSR

**Figure 2:**
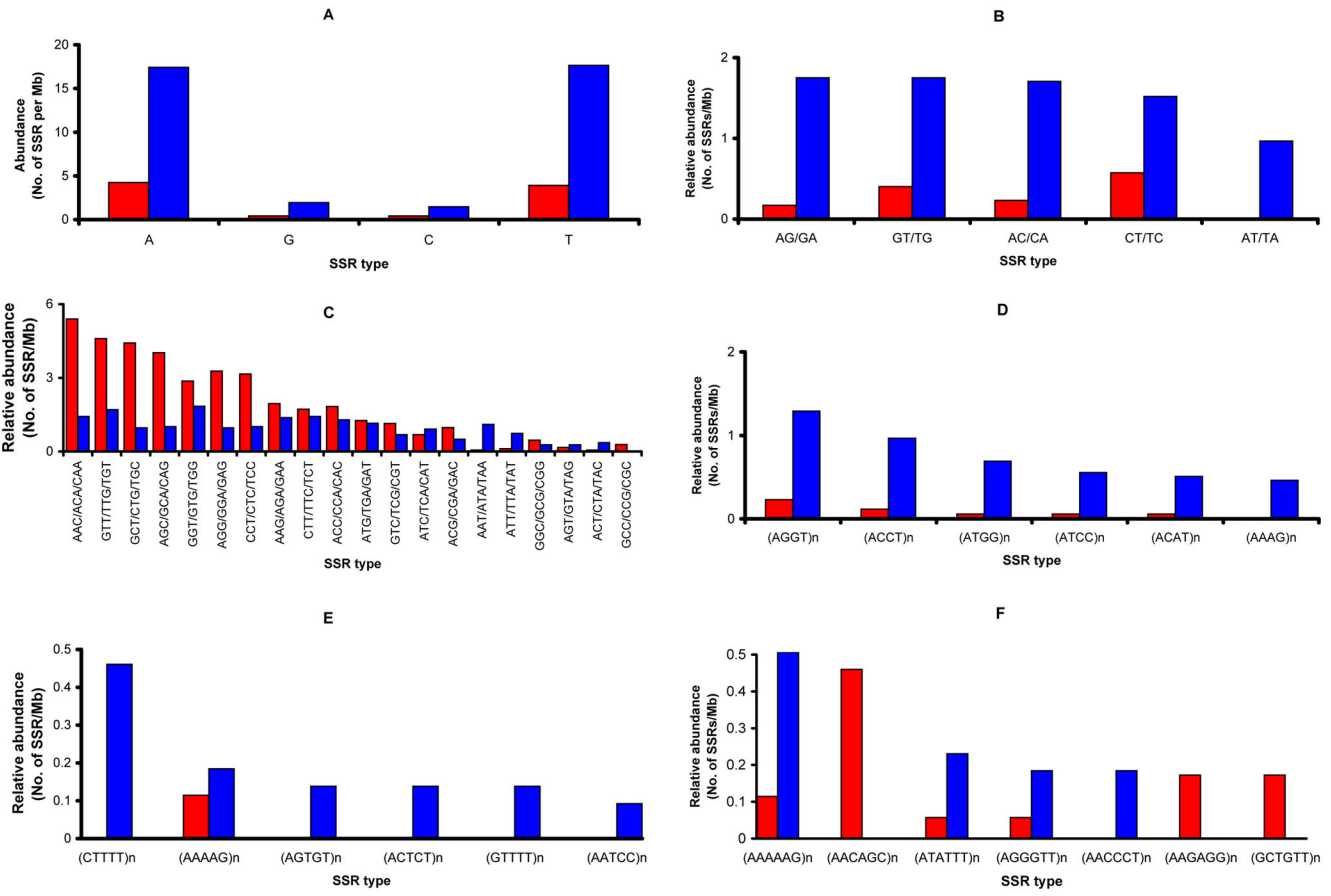
**Genome-wide distribution of relative abundance of SSRs by the SSR types in different SSR unit number**. The relative abundances of SSRs are presented by the genome region: genic region (red bar) and non-genic region (blue bar). Each panel represents a different SSR type: mono-nucleotide SSR (A), di-nucleotide SSR (B), tri-nucleotide SSR (C), tetra-nucleotide SSR (D), penta-nucleotide SSR (E), and hexa-nucleotide SSR (F). From tetra-nucleotide SSR (D), the possible repeats of each microsatellite type are shown as (nucleotide sequence)n. For example, (AGGT)n stands for AGGT/GGTA/GTAG/TAGG repeats. The x-axis represents the different sequence types and the y-axis represents relative abundance where the observed count of SSRs in each category is divided by megabase of sequence.

Mono-nt SSR was the second largest class by repeat unit, representing 36% of the total SSRs in *N. crassa*. Mono-nt SSRs were distributed preferentially in the intergenic and intronic regions and were rare in the exonic region (Fig. 1 and Table 1). The relative abundances of mono-nt SSRs in intergenic, intronic and exonic regions were 38.4, 56.3 and 0.2 per Mb, respectively. Among the possible four types of mono-nt repeats (poly-A, -T, -G and -C), poly-A and -T were the predominant forms: 11.4 poly-A per Mb and 11.3 poly-T per Mb in the genome (Fig. 2).

### Di-nt SSR

Unlike other organisms, the di-nt SSRs were a minor class SSR type in the *N. crassa *genome (Fig. 1 and Table 1) [11,25]. But it was consistent that the di-nt SSRs were preferentially distributed in the nongenic region (Fig. 2) as found in other organisms [11]: 88% of di-nt SSRs present in the intergenic region and 12% in the genic region (0.5% exon and 11.5% intron) (Fig. 2 and Table 1). AG/GA, GT/TG, AC/CA are the most abundant SSR types in di-nt SSRs (about 1 SSR per Mb in each case). The relative abundance of AT/TA was about half that of AG/GA, GT/TG, and AC/CA. No GC/CG SSR type was identified in our analysis.

### Tri-nt SSRs

The tri-nt repeat was the most abundant SSR in terms of unit number: 39.4% of the total SSRs (1084 out of 2749) (Fig. 1 and Table 1). The relative abundance of tri-nt SSRs in the exonic region was approximately two-fold higher than in the intergenic region (40.2 per Mb vs. 19.1 per Mb) (Fig. 1 and Table 1). Among the tri-nt SSRs, AAC/ACA/CAA was the most abundant (Table 1). We also found that some tri-nt SSR types were not randomly distributed in the genome. For example, a group of SSR types, AAC/ACA/CAA, GCT/CTG/TGC, AGC/GCA/CAG, was preferentially located in the exonic region (Fig. 2C). And AAT/ATA/TAA was exclusively located in the intergenic region (Fig. 2C). The tri-nt SSRs in the exonic region are translated into amino-acid repeats, which possibly contribute to the biological function of the protein.

**Figure 3:**
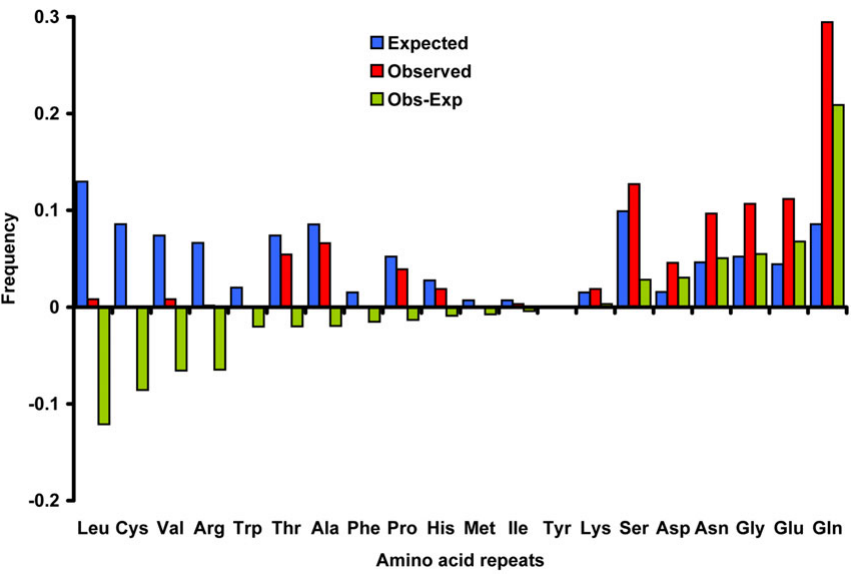
**The predicted and observed frequencies of amino acid repeats encoded by tri-nucleotide SSRs**. Predicted (blue bars) and observed (red bars) frequencies for each amino acid repeat are presented. Green bars represent the differences between the predicted and observed frequencies for each amino acid repeat. If the expected frequency is higher than the observed frequency, the green bar is drawn below the x-axis. If the observed frequency is higher than the expected frequency, the green bar is drawn above the x-axis. Please see Methods and main text for more detailed description.

**Figure 4:**
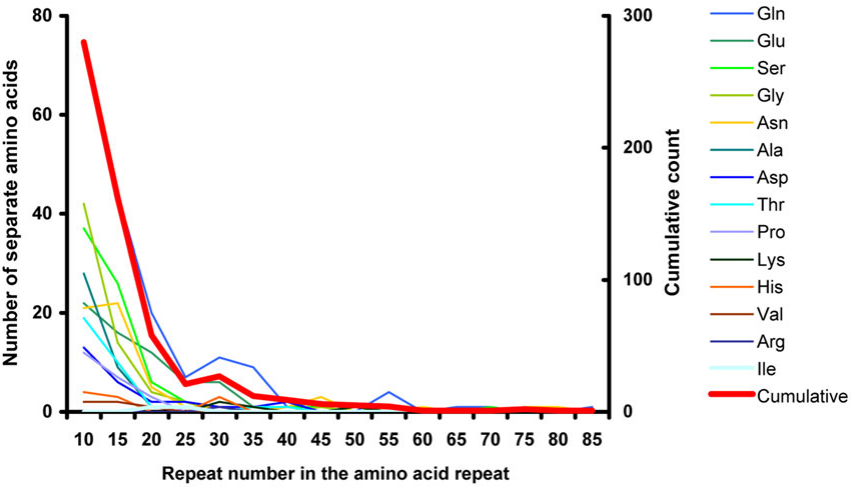
**The distributions of the repeat lengths of the different types of amino acid repeats encoded by tri-nucleotide SSRs**. The x-axis represents the length of amino acid repeat and the primary y-axis (left side) is the observed count of individual amino acid repeats in a given amino acid motif (thin lines) and the secondary y-axis (right side) is the observed count of all amino acid repeats combined (thick red line).

We investigated the frequency of the amino-acid repeats encoded by the tri-nt repeats in the exon (Fig. 3). The frequency was measured based on the encoded amino-acid repeats that are composed of at least 5 repeats of a single amino acid without any interruption. To see if there is a bias in the distribution of amino acid repeats (AAR) encoded by the tri-nt SSRs in the exonic region, we compared the expected and observed frequencies of the encoded AAR (Methods) (Fig. 3). Among the AAR, three AAR accounted for 50% of the total: Glutamine (Gln), 174 repeats, 29%; Serine (Ser), 75 repeats, 12.9%; and Glycine (Gly), 66 repeats, 11.1%. Interestingly, some AAR are present far more abundantly than the expected frequency in the exonic region (p < 0.001). These amino acids are Gln, Glutamic acid (Glu), and Asparagine (Asn), Gly. On the other hand, another group of amino acids, Cysteine (Cys), Tryptophan (Trp), Arginine (Arg), Leucine (Leu), and Valine (Val), are observed at less than expected frequencies (Fig. 3). The longest AAR encoded by tri-nt SSRs was observed for Gln with 81 repeats. Generally, the proportion of amino acid repeats exponentially decreases as the number of repeat units increase in all types of AAR (Fig. 4). This suggests that there could be functional adverse effects when an AAR becomes too large. To characterize the potential biological effects of the size variation of AAR, we grouped the proteins containing AAR using gene ontology (GO). This showed that the proteins containing the AAR that prevail in the *N. crassa *genome are involved in important biological functions in sustaining life, including physiological process (GO ID: 007582), binding (GO ID:0005488), and catalytic function (GO ID:0003824). Small modifications of these genes could trigger large effects in downstream pathways (Additional File 2).

### Tetra-, penta-, hexa-nt SSRs

Tetra-nt SSRs were predominantly distributed in the nongenic regions (Fig. 2D). The two most frequent tetra-nt SSRs were (TAGG)n and (ACCT)n, representing 1.58 and 1.07 repeats per Mb respectively in the genome (Fig. 2D). Penta-nt SSRs were also predominantly distributed in the nongenic regions (Fig. 2E). The most abundant penta-nt SSR was (CTTTT)n. The relative abundance of hexa-nt SSR in the intergenic region was slightly higher than those SSRs in the exonic region: 2.14 vs 1.74 SSRs/Mb, respectively. The most common amino acid repeat encoding hexa-nt SSRs was Gln-Gln repeats (13.8% of the total amino acids encoded by hexa-nt SSRs), which is the same as Gln repeats by tri-nt SSR. The second most common AAR encoded by hexa-nt SSRs was Gly-Ser repeats and Glu-Lys repeats: 6.1% each in hexa-nt SSRs.

### SSR genesis rate in chromosomes and genomic locations

The apparently non-random distribution (Fig. 2) prompted us to further characterize the distribution of SSRs in each chromosome and different genome locations. In this analysis, our goal was to test the *de novo *genesis model in detail: what are the potential parameters that cause the genesis rate of SSRs in the *N. crassa *genome? If the genesis of SSRs (birth of SSRs) in the genome is random, one could interpret that the high abundance of SSRs as the high occurrence rate of SSRs [26].

**Figure 5:**
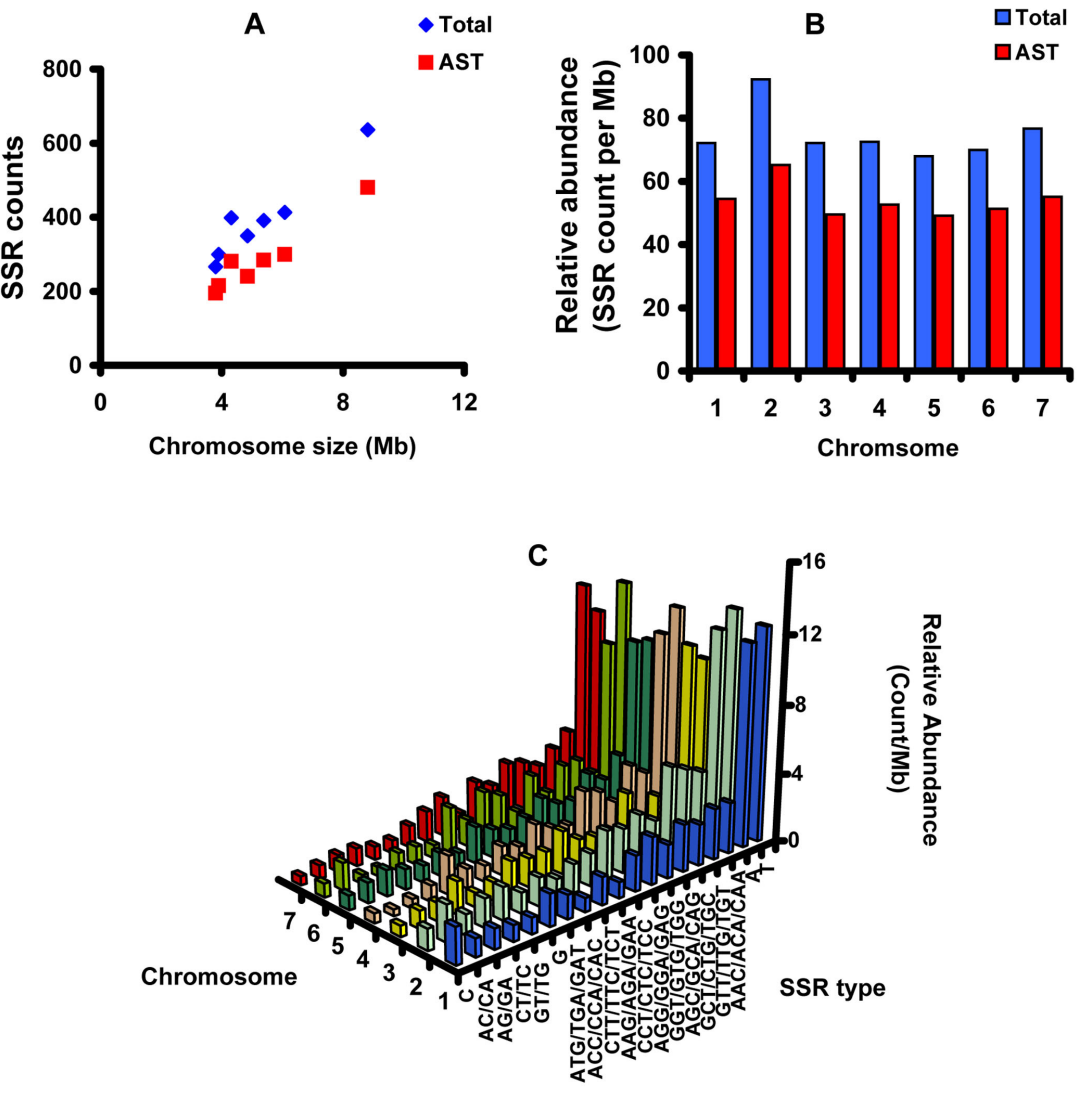
**The distribution SSRs in different chromosomes in the *N. crassa *genome**. A. The scatter plot for cumulative abundance of SSRs (y-axis) and size of chromosome (x-axis). Red squares represent the 19 abundant SSR types (AST) and blue diamonds represent all 963 SSR types (Total). B. Relative abundance of SSRs in different chromosomes. Red bars represent AST and blue bars represent Total. C. The relative abundances of total SSRs are presented by the chromosome and by the SSR type.

In general, the number of SSRs increases with the size of the chromosomes except for chromosome 2 (linkage group II) (Figure 5A, B). The average abundance of SSRs in chromosome 2 is significantly higher than the other chromosomes. Among 963 SSR types that we identified, only 19 different SSR types were present at least more than once per Mb (Table 2). Thus, we classified these SSR types as abundant SSR types (AST). Only mono-, di-, and tri-nt SSRs are included in the AST (Table 2). About 71% of the total *N. crassa *SSRs (1,990) belong to one of the 19 AST and the relative abundance of AST reflects the relative abundance of the total SSRs among chromosomes (Fig. 5B). Moreover, the high copy number of AST allows us to perform statistical tests to characterize the chromosomal distribution of different SSR types.

**Table 2:** Sequential analysis of deviance of log linear model for all 19 SSR types.

**Effect**	**Number of Parameters**	**Change in Deviance**	**Residual DF**	**Residual Deviance**
**Null (*α *only)**	1		265	2396.08
**SSR type (T)**	18	1504.79	247	891.30
**Chromosome (C)**	6	14.53	241	876.77
**Genomic location (G)**	1	43.98	240	832.79
**Interaction (T × G)**	18	604.00	222	228.79

In addition to the variation in the distribution of different SSR types in different functional regions (Fig. 2), the relative abundance of SSR types (SSR counts per Mb) also appears to be variable across the chromosomes (Fig. 5C). Thus, the data suggest that the occurrence rate of a SSR type may depend on both chromosome and functional region. To statistically validate these apparent differences, we performed an analysis of SSR abundance for the 19 AST using a Poisson log linear model [27]. The probabilistic motivation for the Poisson model is that random occurrence of an SSR in the genome is synonymous with SSR "events" occurring according to a Poisson process, when traversing the genome from one end to the other. The data presented in Fig. 2 and Fig. 5 indicates substantial variability in abundance rates among types in each chromosome and genomic location (Fig. 2 and 5). There was no a priori reason to expect variation in abundance between chromosomes. However, the log-linear modelling approach allowed multiple factors to be examined simultaneously in a unified statistical framework. Thus, we analyzed three factors in our analysis: chromosome, SSR type, and genomic location (genic vs. intergenic). Our analysis was based on the data for the 19 AST, with a cumulative total of 1990 SSR occurrences. For the purpose of our statistical analysis, the data were summarized as two 19 by 7 contingency tables (one for genic and one for intergenic regions) giving the frequencies of the 19 SSR types on each chromosome (Additional File 3). The abundance for each SSR type/chromosome/region combination was defined as the number of SSRs divided by the length of the relevant region on the chromosome in Mb. Our statistical model assumes that the effects are all additive on a log rate scale, and therefore multiplicative on the rate scale (Methods). The goodness-of-fit of this model is summarized in the analysis of deviance decomposition given in Table 2. In particular, the residual deviance for the full model, 228.79 with 222 degrees of freedom, indicates a good overall fit. In addition, all of the factors in the model, including the chromosome main effects, are statistically significant. In particular, adding SSR type as an explanatory factor to the null model reduces the residual deviance by over 1500, which is clearly statistically significant (p < 0.0001 when compared to a chi-squared distribution with 18 degrees of freedom). Thus, abundance is clearly not uniform over SSR types There is also a modest, but statistically significant, chromosome main effect (chi-squared = 14.53 with df = 6, p = 0.02). The cause of the significant chromosome effect was the higher overall SSR abundance on chromosome number 2 relative to all other chromosomes.

The statistical significance of the SSR type/genomic location interaction was partially explained by the fact that the 8 mono- and di-nt SSR types (among the 19 AST) were almost non-existent in the genic region, whereas the 11 tri-nt SSR types combined are approximately equally abundant in the genic and intergenic regions. For this reason we considered separate fits of the log linear model to the mono/di-nt SSR data and the tri-nt data, with the genomic category factor omitted from the model in the mono/di-nt data case. The sequential deviance decompositions for the two data sets are reported in Tables 3 and 4. Table 3 indicates a significant SSR type effect as observed in Table 2, but no strong evidence of differences between abundance rates of mono/di-nt SSR types among chromosomes. The statistical significance of the SSR type factor was a consequence of the large differences in the empirical abundance rates, which strongly suggest that the genesis rates of different SSR types were not uniform. The residual deviance after dropping chromosome as a factor in the model was 45.75 with 48 degrees of freedom, indicating a good fit for the Poisson model with SSR dependent abundance rates.

**Table 3:** Sequential analysis of deviance of log linear model for 8 mono/di-nt SSR types, intergenic region only.

**Effect**	**Number of Parameters**	**Change in Deviance**	**Residual DF**	**Residual Deviance**
**Null (*α *only)**	1		55	1130.89
**SSR type (T)**	7	1085.14	48	45.75
**Chromosome (C)**	6	8.89	42	36.86

**Table 4:** Sequential analysis of deviance of log linear model for 11 tri-nt SSR types.

**Effect**	**Number of Parameters**	**Change in Deviance**	**Residual DF**	**Residual Deviance**
**Null (*α *only)**	1		153	409.70
**SSR type (T)**	10	68.05	143	341.65
**Chromosome (C)**	6	10.37	137	331.28
**Genomic category (G)**	1	150.92	136	180.00
**Interaction (T × G)**	10	47.68	126	132.31

The story for the tri-nt SSR counts was more complex (Table 4). Dropping chromosome from the model increases the residual deviance by a statistically insignificant 10.38. Examination of the chromosome coefficients does indicate a significantly higher abundance value on chromosome number 2. This may be just a statistical anomaly or may indicate the existence of a differential SSR genesis rate in chromosome 2. The residual deviance for the reduced model was 142.69 with 136 degrees of freedom, again indicating that the Poisson variation model was reasonable. However, not only do the abundance rates vary by SSR type, but the differences depend upon the genomic location category (intergenic/genic). While abundance was generally higher in the genic region, the pattern was not uniform across all 11 tri-nt types. In some cases there was no significant difference between genic and intergenic regions (Table 5).

**Table 5:** Comparison of log abundance rates in genic and intergenic regions based on a Poisson model.

**SSR**	**Genic**	**Intergenic**	**Diff**.	**Std.err**	**Z-value**	**P-value**
**AAC/ACA/CAA**	1.689	0.335	1.354	0.216	6.280	0.000***
**AAG/AGA/GAA**	0.683	0.370	0.312	0.253	1.236	0.216
**ACC/CCA/CAC**	0.590	0.335	0.255	0.261	0.979	0.328
**AGC/GCA/CAG**	1.405	0.094	1.311	0.244	5.363	0.000***
**AGG/GGA/GAG**	1.182	0.047	1.134	0.256	4.433	0.000***
**ATG/TGA/GAT**	0.247	0.222	0.026	0.292	0.088	0.930
**CCT/CTC/TCC**	1.164	0.047	1.116	0.257	4.352	0.000***
**CTT/TTC/TCT**	0.557	0.437	0.121	0.256	0.471	0.638
**GCT/CTG/TGC**	1.474	0.047	1.426	0.247	5.778	0.000***
**GGT/GTG/TGG**	1.068	0.640	0.428	0.215	1.988	0.047*
**GTT/TTG/TGT**	**1.538**	**0.558**	**0.980**	**0.202**	**4.836**	0.000***

* indicates significance at the 5% level, ** 1%, and *** 0.1%

### Size polymorphism in SSRs among natural accessions

**Figure 6:**
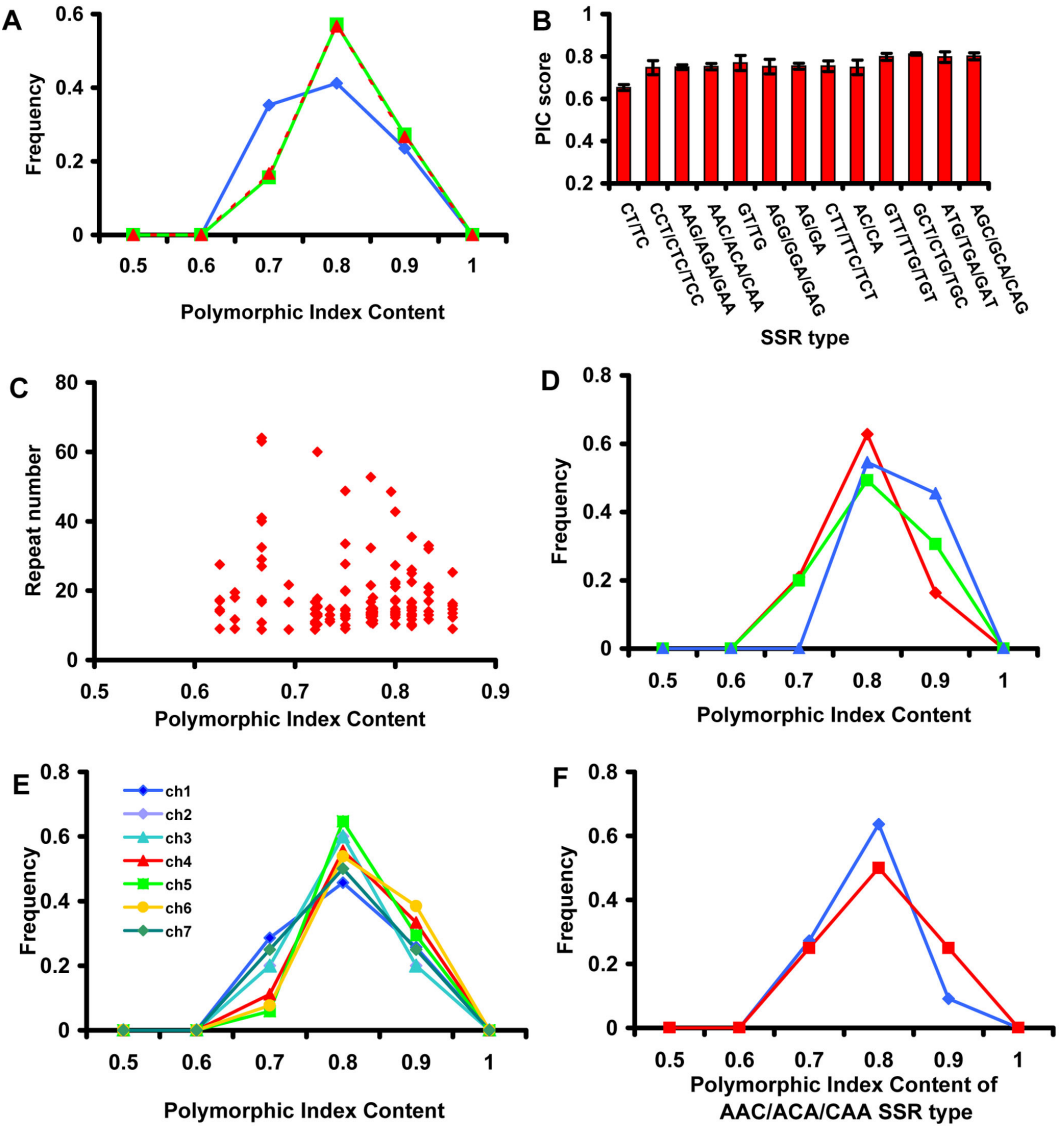
**The polymorphic information content analysis from 129 SSR loci**. A. The frequencies of the PIC values of 129 SSR loci are displayed by the SSR type: di-nucleotide SSR (blue diamond), tri-nucleotide SSR (green square), and tetra-nucleotide SSR (red triangle). B. The comparison of PIC scores in the sampled SSRs of different SSR types. The error bars show the standard error of the mean. C. The scatter plot for PIC score and the repeat number among the sampled SSRs. The frequencies of the PIC values of 129 SSR loci are displayed by the genome region (D), exon (red diamond), intron (blue triangle) and intergenic region (green square), and by the chromosome (E). F. PIC score distribution of one SSR type, AAC/ACA/CAA, located either in genic (blue diamond) or intergenic (red square) regions.

Next, we tested if the size variations of SSRs among natural accessions suggest evolutionary forces for the cause of SSR size variations. We scanned the genome using a 250 kb window and randomly selected a SSR within each window. Mono-nt SSRs are not easy to accurately assay for their repeat number and could mislead our analysis [28], so they were eliminated from this analysis. Of the 1759 SSRs (after removing mono-nt SSRs), we selected 162 SSRs for further analysis (Methods). We analyzed the characteristics of the 162 selected SSRs and found that their distribution, repeat units, and frequencies were comparable to those in the complete genome-wide collection (Additional File 4). To test size polymorphism of the 162 SSRs, primers were designed and used to screen the length polymorphism in a SSR locus with 7 natural accessions (Methods). Subsequently we accessed the size variability of SSRs represented by the polymorphic index content, PIC (Methods). The PIC value 0 represents no polymorphism among alleles and the PIC value 1 represents the most complete polymorphism. Of the 162 SSR loci, 33 SSR loci were eliminated from further characterization due to PCR failure or ambiguous results. We calculated the PIC scores for the remaining 129 SSR loci. The range of the PIC scores spans from 0.63 to 0.86. All the results of the polymorphism analysis for the 129 sampled SSR loci can be found in Additional File 5. In this analysis, we considered two different parameters, physical characteristics of SSRs (repeat number, type, and length) and genome location of SSRs (chromosomes and genic vs. intergenic). First, we grouped these experimentally characterized SSR loci to test if the distributions of PIC scores are associated with different physical characteristics of the SSRs. There were no significant differences in the mean values of PIC scores among repeat units or SSR types (p = 0.86 and p = 0.84 respectively, using one-way ANOVA) (Fig. 6A and 6B), and there was no significant correlation between PIC and repeat number (p = 0.4) (Fig. 6C). Second, we compared PIC scores of 129 SSRs in two functional regions (genic vs. intergenic) (Fig. 6D) and the seven chromosomes (Fig. 6E). We also found that there were no significant differences in the distribution of PIC scores in different functional genome regions (genic vs. intergenic) (p = 0.2, Fig. 6D) and chromosomes (p = 0.94, Fig. 6E). Thus, these data suggest that there was no systematic difference in terms of the variations of PIC values among different physical characteristics of the SSRs tested here, or across functional regions, or chromosomes. Finally, we also compared the PIC value distributions of the same SSR type (AAC/ACA/CAA) in 20 different loci at different functional genome locations and found that there were no significant differences in PIC scores between genic and intergenic regions (p = 0.84, Fig. 6F). These results suggest that there is no apparent bias in SSR genesis rates in 1) the physical characteristics of SSRs, 2) genomic locations, and 3) chromosomes. It is worth noting that the SSR size variability of *N. crassa *that is estimated from our study is relatively high, in comparison to other organisms [5,6], with an average PIC score = 0.8.

We were concerned that the PIC values calculated from seven accessions might not reflect the true PIC values among all accessions in nature. To test this, we randomly chose 32 strains from the collection of natural accessions in the Fugal Genetics Stock Center (FGSC, Kansas) and analyzed the PIC score of one SSR type, AC/CA, at three randomly chosen different loci. The PIC values of the AC/CA SSR type with two different population sizes, 7 vs. 32, were not significantly different (two sample t-test, p = 0.19, Additional File 6).

### Statistical inference for evolutionary forces of size variation of SSRs

We thought that the size variation of SSRs in seven different accessions could provide some insights in terms of the occurrence of size variations in nature. We hypothesized three simple scenarios regarding the scope of evolutionary forces in SSR size variation for statistical tests: 1) Hypothesis #1 (genome-wide effect), the sizes of the SSRs in the genome are either longer or shorter for a given strain in comparison to those in other strains, 2) Hypothesis #2 (local effect), the sizes of some SSRs are either significantly shorter or longer than other SSRs, 3) Hypothesis #3, there could be both significant differences among strains and within a strain (Additional File 7). To test these hypotheses, we used 33 SSRs that had no missing data in seven strains (Additional File 8). The distribution of repeat numbers across all seven strains and 33 markers is considerably right-skewed. This skewness was largely removed by a (natural) log transformation. In the following analysis we attempted to isolate the sources of variation in the log transformed repeat numbers by taking into account the strain, chromosome, genome regions (genic vs. intergenic), and SSR type. It is worth noting here that, unlike in the analysis of the SSR counts, there is no particular reason that the repeat number should have a Poisson distribution. Accordingly, our analysis of the repeat numbers uses classical linear models with the natural logarithm of the repeat number as the response variable.

Comparison of the averages of repeat numbers for the seven strains using one-way ANOVA indicates significant differences among the strains. Pairwise comparisons indicate that strain FGSC#2489 has a significantly higher average of repeat numbers than the other six strains (P < 0.05 for all pairwise comparisons with strain FGSC#2489). The strain FGSC#2489 is the sequenced standard laboratory strain that has been developed through an extensive backcrossing in the laboratory [29]. We are tempted to speculate that the systematic difference in the repeat numbers of SSRs between FGSC#2489 and other natural accessions could be a result of repeated selection in the laboratory environment. This supports our hypothesis #1 that there was a strain specific (genome-wide) effect in SSR size variation. There are also significant differences among the six natural strains. However, after Bonferroni adjustment of the pairwise P-values, the only significant differences were strains FGSC#3223 and FGSC#2489 having higher average repeat numbers than strains FGSC#4720 and FGSC#4724. Strains FGSC#4825, FGSC#2223, and FGSC#4715 have average repeat numbers in between these two pairs, none being significantly different from either extreme after Bonferroni adjustment.

In an attempt to more carefully analyze the size variation, we performed two-way ANOVA analyses: 1) strain by functional regions, 2) strain by chromosome, and 3) strain by SSR type. Analysis of the means by strain and region shows no significant interaction (P = 0.36) and no genic region main effect (P = 0.07). Analyses of the means by strain and chromosome shows no significant interaction (P > 0.99) and no significant chromosome main effects (P < 0.30), but a significant strain effect (P < 0.0001). The estimated strain effects have a similar pattern as in the one-way analysis. Thus, this result also supports the hypothesis that there was a genome-wide regulation of SSR repeat number.

Since each marker occurs exactly once in each strain, it was not possible to conduct a global test for strain by marker interaction. However, a singular-value decomposition of the 33 by 7 interaction matrix, *M*[30], reveals that there are two dominant components that account for more than 60% of the residual variation after accounting for strain and marker main effects. Thus, we consider a linear model of the form,

$$Y_{ij}=\mu +\beta_{i}^{S}+\beta_{j}^{M}+\lambda_{1}u_{1i}v_{1j}+\lambda_{2}u_{2i}v_{2j}+\varepsilon_{ij},$$

where *Y*_*ij *_is the log repeat number for marker *j *on strain *i*, *μ *was the overall mean, $\beta_{i}^{S}$ is the main effect of strain *i*, and $\beta_{j}^{M}$ is the main effect of marker *j*. The vectors *u*_*k *_and *v*_*k*_, *k *= 1,2, are the unit eigenvectors corresponding to the largest two eigenvalues of the matrices, *MM' *and *M'M*, respectively. This is an additive main effects and multiplicative interaction (AMMI) model that has been widely used in the analysis of agricultural yield trials [31]. The *ε*_*ij *_terms account for residual variation (interaction) not explained by the multiplicative component. The least squares estimate of the parameter, *λ*_*k*_, is equal to the singular value associated with the eigenvectors, *u*_*k *_and *v*_*k*_, in the singular value decomposition of the interaction matrix, *M*. The square of this singular value is equal to the sum of squares explained by the multiplicative interaction component in the ANOVA decomposition for this model as summarized in Table 6.

**Table 6:** ANOVA decomposition of log repeat number using strain and marker factors.

Source	DF	Sum Sq	Mean Sq	F-statistic	P-value
Strain	6	9.0540	1.5090	14.5277	<0.0001
Marker	32	25.8884	0.8090	7.7887	<0.0001
First Component	37	9.0534	0.2447	2.3557	0.0003
Second Component	35	7.9603	0.2274	2.0713	0.0009
Residual	120	12.4644	0.1039		

Examination of the first eigenvector for markers reveals a very large positive loading on marker 48. On the other hand, the first eigenvector for strain was essentially a contrast between two groups of strains, one group including FGSC#4720 and FGSC#4715 and other group including FGSC#4825, FGSC#2223, FGSC#4724, FGSC#3223, and FGSC#2489. Thus, one source of the strain by marker interaction appears to be caused by the extremely high repeat numbers for marker 48 in strains FGSC#4720 and FGSC#4715, relative to the other five strains. The second eigenvector for markers has a dominant positive loading on marker 201 and a dominant negative loading on marker 1. In this case, the corresponding eigenvector for strain was a contrast between the pair of strains, FGSC#4724 and FGSC#3223, and the remaining five strains. Thus, a second source of interaction appears to be due to the contrast between these two groups of strains with respect to the difference in repeat numbers between markers 1 and 201, relative to this contrast for any other pair of markers. These data support our hypothesis #3 that there are variations in SSR repeat numbers that genome-wide effects alone cannot explain.

An alternative simple analysis is to look for markers that have highly variable (log) repeat numbers across the seven strains. Under the assumption that the seven (log) repeat numbers for a particular marker are a random sample from a normal distribution, the sample variance is proportional to a chi-squared statistic with 6 degrees-of-freedom. Specifically, 6*s*^2^/*σ*^2 ^~ *χ*(6), where *σ*^2 ^is the unknown true variance. Using the chi-squared reference distribution with *σ*^2 ^replaced by the median sample variance from the 33 markers, we found 4 markers with significantly large sample variances (P < 0.005). In order of increasing variance these are markers 201, 34, 48 and 1. Thus, three of the four markers with the largest sample variances are the ones found using the ANOVA methods. Based on these data, we concluded that there are genome-wide, chromosomal, and local effects in size variation of SSRs.

### Genetic map construction

It was suggested that SSRs could be useful molecular markers for genetic analysis in intra-species populations due to the hypervariablity of SSRs [8]. Earlier in this paper we also confirmed this high variability of SSRs in *N. crassa*. In addition to hypervariability, a useful genetic marker should show stable inheritance. Thus, we wanted to examine the stability of the SSRs as genetic markers by constructing linkage maps from intra-species populations generated by crossing *N. crassa *natural accessions (Table 7). We also reasoned that the polymorphic SSR markers could provide a means of detecting chromosome rearrangement if there was a significant chromosome rearrangement among accessions. We found that 140 SSR markers out of the 162 (86.4%) exhibited polymorphisms of either co-dominant or presence/absence types in at least one pair of the mapping parents (Table 7). Utilizing the polymorphic SSR markers, 188 F1 haploid progeny derived from each mapping population were genotyped (Methods). We coalesced 109 SSR loci out of 140 SSR loci into the three genetic maps: N2 (50 out of 71 SSRs, 70.04%), N4 (69 out of 94 SSRs, 77.2%), and N6 (70 out of 91 SSRs, 76.9%) (Additional File 9).

**Table 7:** Line-cross populations from *N. crassa *accessions.

Cross number	Parents*	Mating type	Origin of collection	Polymorphic SSRs
N2	3223	*mat A*	Louisiana, U.S.A.	74
	4724	*mat a*	Penang, Malaysia	
N4	4720	*mat A*	India	94
	4715	*mat a*	Haiti	
N6	4825	*mat A*	TiassaleI, Ivory Coast	91
	2223	*mat a*	Iowa, U.S.A.	

* Fungal Genetics Stock Number

**Figure 7:**
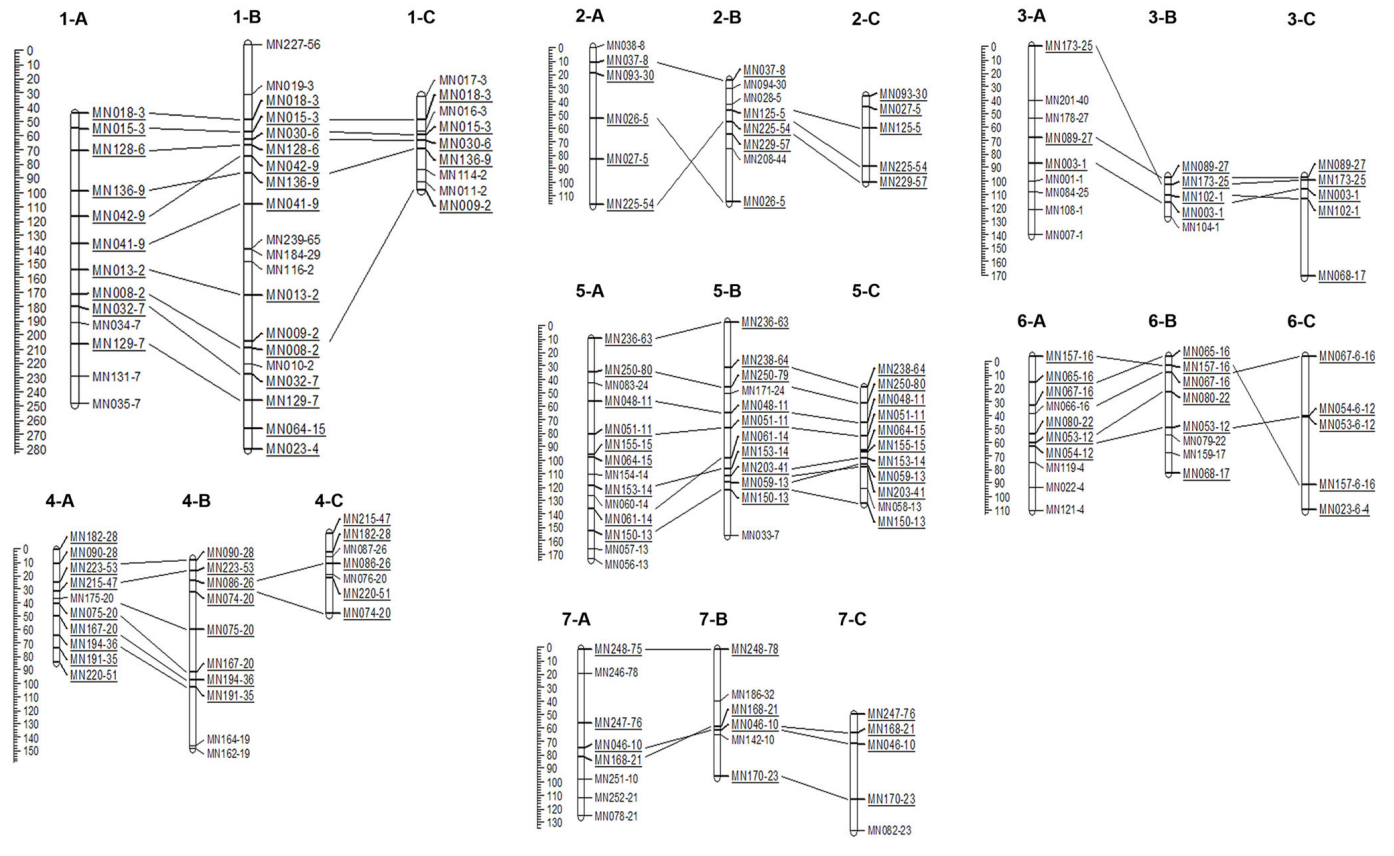
**Genetic linkage maps of three mapping populations**. Each linkage group is named according to their cross number and the corresponding chromosome number. Cross number is expressed by a letter; A (N6, FGSC#4825 × FGSC#2223), B (N4, FGSC#4720 × FGSC4715), and C (N2, FGSC#3223 × FGSC#4724). For example, 1-A indicates the linkage group that corresponding chromosome 1 in the cross N2. The corresponding linkage groups from different crosses are alignedbased on the relative positions of anchor markers. The anchor markers are underlined and connected by thin lines among the corresponding linkage groups. The physical location of each marker is indicated by the super-contig number followed by the marker name, e.g. MN018-3 and MN015-3 are two markers that are located in the super-contig 3 [70]. The scale on the left of each linkage group shows a relative map position denoted by centi-morgan (cM).

To evaluate the co-linearity of the mapped SSR loci among the three populations and the physical map based on the sequenced strain, FGSC# 2489, SSR marker orders among the three mapping population were cross examined by using commonly mapped loci and a physical map. The positions of the mapped SSR loci from the three mapping populations were highly consistent with the physical map positions, with few exceptions (Fig. 7). These exceptions are found in closely linked markers, especially when the markers are located in the same contig, for example, MN153 and MN061 on linkage group 5. No errors in genotyping or significant segregation distortion at adjacent markers were detected (Additional File 9).

## Discussion

### Distribution of SSRs in the sequenced *N. crassa *genome

There is a discrepancy between the numbers of the estimated SSRs in the *N. crassa *genome in our study and a previous report [32]. The discrepancy could be attributed to the following facts: 1) we used different algorithms from the one used in the previous analysis; and 2) we used the most up-to-date genome sequence (release 7), whereas, the previous analysis used an earlier version (release 3) of the genome sequence. In addition, it should be noted that there is currently no consensus among researchers regarding how to define SSRs [1]. To achieve our goals, we applied more stringent conditions to define the SSRs than previously used. Since rates of SSR mutations are positively correlated with SSR lengths, we chose to have the number of nt within the SSR locus to be greater than 21 [1,33,34].

We found similarities and differences in SSR compositions and distributions between the sequenced *N. crassa *genome and other eukaryotic genomes. In the mono-nt SSRs, which accounted for 36% of the total SSRs, poly-A/T was far more abundant than poly-G/C. Indeed, A/T is most abundant across the *N. crassa *genome. Most mono-nt SSRs were located in the intergenic and the intronic regions but rarely located in the exonic regions. This overrepresented A/T SSR tract in the *N. crassa *genome resembles the pattern found in the primate genome [2,32,35,36]. In general, di-nt SSRs are the most common SSRs in many organisms [2,36]. However, di-nt SSRs represent only 6.9% of the total SSRs in the *N. crassa *genome. Among the di-nt SSRs, the proportion of the AT/TA SSR type was smaller than those of the other di-nt SSRs: AG/GA, GT/TG, AC/CA, and CT/TC. This result may reflect the difference in SSR compositions between fungal and other organisms [1]. It is also possible that the AT/TA SSR type could have been underestimated because of our stringent SSR definition (discussed earlier), thus accounting for the difference between the studies.

Tri-nt SSR is the major class of SSRs in the *N. crassa *genome. In our analysis, tri-nt SSRs accounted for 39.4% of the total. This is larger than the di-, tetra-, penta- and hexa-SSRs combined (24.5%). The predominance of tri-nt SSRs in *N. crassa *appeared to be a unique feature compared to other sequenced fungal genomes [32]. In terms of relative abundance, there were twice as many tri-nt SSRs present in the exonic region than in the intronic and intragenic regions combined. The enrichment of the tri-nt in the exonic region has been observed in other eukaryotic organisms across taxa [2,10,35,37]. This pattern was attributed to a tight negative selection on the other SSRs (other than tri-nt SSRs) that would perturb the reading frame in the coding regions [2,10,38-40]. Our analysis shows that most SSRs (74%) are predominantly distributed in the intergenic and intronic regions, with tri-nt and hexa-nt SSRs being exceptions (Table 1 and Fig. 1). Moreover, the presence of SSRs, such as ATG variants that could act as a start codon, or TTA variants that could act as a stop codon, are restricted in the exonic region (See Table 1, Fig. 1 and Fig. 2).

### Potential role of AAR encoded by tri-nt SSRs

Our results suggest that AAR encoded by tri-nt SSRs have undergone positive and negative selections, depending on their sequence types: three AAR (Gln, Glu and Ser) were over-represented and three AAR (Leu, Cys and Val) were under-represented in the genome (Fig. 3). This suggested that the observed size variation of tri-nt SSRs within a gene may be differential, possibly due to functional selection on the amino acid reiteration in encoded proteins [10,11,41]. Previous analyses of protein database and genomic sequence in different taxa found that AAR stretches of small hydrophilic amino acids were more tolerated in proteins [10,41]. In agreement with previous reports, our data showed that the hydrophilic amino acids including Gln, Glu and Ser repeats are over-represented in the *N. crassa *genome (Fig. 4). However, the tolerance of AAR stretches in proteins has certain restrictions. Our results showed that the proportion of AARs exponentially decreased as the number of repeat units increased in all types of AARs, with 25 repeats being a critical threshold (Fig. 4). This may be because longer AAR repeats have such detrimental effects on protein functions that they are apt to be selected out in the genome [10,41,42].

Numerous lines of evidence have been accumulated to support the potential roles of the AAR encoded by tri-nt SSRs in the functional divergence of proteins [10,12,43,44]. Hydrophilic AAR stretches can be a major source of phenotypic variations [10,18]. For instance, expansion of CAG repeats resulting in poly-Gln repeats in various neurological genes in humans can cause changes to their original gene functions and lead to various neuronal disorders including Huntington's disease, detatorubro-pallidoluysian atrophy, spinbulbar muscular atrophy, and spinocerebellar ataxia [44].

It is suggested that gene duplication has a fundamental role in diversifying gene function[45]. However, diversifying gene function by gene duplication is probably not a good option for *N. crassa *because it has a genome defense mechanisms, Repeat-Induced Point mutation or RIP [45]. Neurospora detects duplicated copies of sequences in the genome and mutates both sequences by repeated point mutations during the sexual cycle [45]. Thus, the questions of whether and how *N. carssa *could generate diversified functional genes has been raised [46]. We propose that the AAR encoded by tri-nt SSRs might have a crucial role in creating functional variability of gene regulation. Since RIP requires a minimal duplicated sequence length of about 400 base pairs (bp) [46], a tandem repeat of SSRs less than 400 bp within a gene may escape from the influence of RIP and hence may modify the original gene functions efficiently [10,47]. The proteins including AAR are in diverse functional groups (Additional File 2). Furthermore, the size variations of tri-nt SSR in exonic regions are variable across the *N. crassa *genome (Fig. 6). These raise the possibility that highly active contraction and expansion of the tri-nt SSRs in exonic regions may play roles in the evolution of gene functions that may facilitate adaptation in new environments [10,14,47].

We explored the possibility of SSRs being a target of functional variation of circadian rhythms in nature. First, we surveyed the variation of repeat numbers of SSRs located in ORFs of known circadian clock genes, *white collar-1 *(*wc-1*), *white collar-2 *(*wc-2*), *vivid *(*vvd*), and *frequency *(*frq*), among 143 *N. crassa *natural accession collected from all over the world. We found significant size variations of SSRs in clock genes. Furthermore, these variations were associated with circadian rhythms [48]. WC-1 is a blue-light receptor for circadian clock and it functions as an activator in a complex with a partner, *wc-2*. We focused our study on the polyglutamine repeat domain in the amino-terminal of *wc-1*, NpolyQ, which has been proposed as an activation domain [49,50]. Previous studies also suggested that NpolyQ plays a role in clock-specific activation [51,52]. We found that NpolyQ is a target for period variation. Furthermore, we found evidence that variation in the circadian clock was associated with latitude of collection, which suggested that the WC-1 genotype provided an adaptive advantage in natural populations. The quantitative role of variation in the amino-terminal polyglutamine (NpolyQ) domain of WC-1 in period variation among accessions has been confirmed in an independent experimental line cross population [48]. Further functional characterization will be directed toward determining the effects of the variable AAR encoded by tri-nt SSRs on the corresponding gene functions and their ecological implication.

### Evolutionary inference of SSR variations in *N. crassa*

We attempted to infer factors on size variation of SSRs in the *N. crassa *genome by statistical analysis of size variations of 33 markers in seven accessions. Our results suggest that there were at least three different levels of statistically significant factors (genome-wide, chromosome-specific, and local effects) involved in size variations of SSRs in the *N. crassa *genome. Our study does not address the actual mechanism of variation in SSR repeat numbers; however, it provides foundations for further experimental verification. One of the widely discussed theories on the genesis of the length variation of SSRs is the strand-slippage theory, that the variation of length in SSRs is caused by slipped-strand mis-pairing and subsequent errors during DNA replication, repair, and recombination [1,11,53,54]. This could be a good explanation for the strand-slippage theory with an assumption that there is no bias in the rate of mis-pairing in genomic regions during the replication process. However, there are reports that the length variation does not follow in a step-wise manner because the efficiency of the length variation may differ due to numerous local circumstances in the genome [1,10,11]. Our data also suggest that the genome-wide mechanism cannot be the only source variation for SSR size variations. The existence of chromosome-specific and local effects suggests that genomic context is an important factor for the variation of SSR repeat numbers. More research should be focused on the factors influencing the local variation.

RIP could be a potential mechanism for the observed species-specific bias in SSR distribution. RIP refers to a genetic phenomenon that mutates duplicated sequences in a genome during the sexual cycle [22,55]. Both duplicated regions go through C:G to T:A mutations preferentially at CpA di-nt [56,57]. For example, a segment of the *Tad *1-1 sequence, ...ACACA..., is mutated to, ...ATATA..., after RIP in all progeny the authors analyzed (Fig. 4 in [21]). The systematic mutations of these types could accelerate the genesis of certain types of SSRs and interrupt others. We did not find CG/GC repeats in the *N. crassa *genome. This observation could be explained by RIP since the expanded CG/GC repeats could be a target for repeated RIP. Characterizing the roles of RIP in SSR evolution requires more careful study.

### Marker potential

The estimated PIC value was comparable and relatively high for SSR markers compared to other organisms [58-60] where the SSR marker system has been applied to many genetic analyses. The average PIC in rice is 0.637 [61], in soybean 0.43 [62], and in wheat 0.40 [63]. The mean PIC score in the rust fungus, *Puccinia graminis*, is 0.49 [64], and the mean PIC score in *Diplodia pinea *[= *Sphaeropsis sapinea*], a well-known pathogen causing a shoot or tip blight of numerous pine species and some other conifers, is 0.43 [65]. Compared to these organisms, SSR size variability estimated by PIC scores in *N. crassa *seems to be relatively high. To estimate a mean PIC value objectively, the tested SSR loci should be randomly sampled. However, unbiased sampling, as done in our study, is not easy to achieve even in sequenced organisms. Since the PIC calculations in many studies, including those mentioned above, are based on the available SSR marker set rather than a random sample, they could produce biased estimates of the PIC for the genome.

The high PIC value in *N. crassa *implies that the SSR marker system has sufficient resolution/polymorphism to be used for genetic studies. Even though our current study of polymorphism of the selected SSR types uses a rather small sample of 7 strains, our PIC estimation of the SSR type AC/CA was consistent with the estimation using a larger sample of 32 strains (Additional File 6). A larger scale study of genome-wide SSR analysis with a bigger population would be required to obtain a more comprehensive understanding of the distribution of SSRs in fungal genomes.

We investigated whether the polymorphism of SSRs could be affected by any of the factors including different repeat units, SSR types, chromosomes, repeat numbers, and total SSR lengths (Fig. 6). Our result showed that there were no significant differences in PIC scores among those criteria (Fig. 6) in the *N. crassa *genome. Since the mutation rate seems to be random across the genome, it was difficult to estimate the mutation rates of SSRs in different categories, i.e. SSR types or functional regions. Thus, empirical characterization of size variability for each SSR is necessary to estimate the usefulness of a particular SSR as a molecular marker.

Currently, genomic sequences of many fungal organisms are accessible through public genome databases. Identification of SSRs can be easily done using several publicly available software packages. However, despite the many advantages of SSR markers in various biological studies, the lack of experimental data on polymorphic SSR markers is still a major limitation for utilizing SSR markers in biological studies in fungal systems. Thus, community based databases for SSRs will expedite the implementation of SSR markers in genetic and genomic studies in *N. crassa *as well as in other fungal organisms.

### SSR based genetic map construction

Recently, molecular marker techniques for assisting efficient mapping/gene-cloning have been developed in *N. crassa *system [66-68]. All of these techniques utilize polymorphisms at the nucleotide level. The usefulness of polymorphisms found in SSRs for evolutionary studies was explored by Dettman and Taylor [8]; 13 SSRs in 147 strains from eight species of *Neurospora *have been analyzed. The authors sequenced 5 SSRs and about 500 nucleotides of the flanking sequences, and then characterized the genealogical relationships between SSR alleles by mapping them onto a tree drawn by flanking sequence data. This study revealed that SSRs are not appropriate for studies on inter-phylogenetic relationship among species due to high mutation rates in SSRs (about 2500 times greater than those of flanking sequences) and allele length homoplasy [8]. The same report also suggested that SSRs could be used for population studies in inter-species populations. In the current study, we wanted to test if we could experimentally confirm this prediction by constructed three linkage maps using three independent F1 populations.

Based on our SSR polymorphism data, we were able to construct three different genetic maps from the three different pairs of *N. crassa *natural accessions (Table 4). A previous study estimated that the *Neurospora *genome is about 1000 cM [29]. The discrepancy in the estimated genome-wide map units, between our estimation and the previous study, is mostly due to the different coverage of either molecular or genetic markers for each strain. Our linkage maps roughly agree with the previous estimation, about 1000 cM [29].

The orders of SSR markers along the chromosomes were conserved well among the three mapping populations in our analysis. Furthermore, the positions of mapped SSR loci from the three mapping population are highly consistent with the positions in the physical map, with a few exceptions, suggesting that the genetic architecture of the 6 natural accessions are highly similar to each others. One of the exceptions was the loci order between closely linked markers. Because of this, the inconsistency is mostly attributable to statistical complications caused by a lack of recombination information between two tightly linked markers, rather than chromosomal rearrangements due to missing values or segregation distortions. A previous simulation study also supports our interpretation [69].

## Conclusion

We conclude that the distributions and size variations of the SSRs in *N. crassa *showed statistically significant patterns. We could not find evidence that the mutation rates (manifested by PIC score) are correlated with various factors including chromosomes, genomic categories, SSR types, repeat numbers, and total SSR lengths. This suggests that the factors affecting the mutation rate could be random across the genome. Thus, the non-random distribution pattern of SSRs presumably reveals the functional significance of SSRs. The size variations of tri-nt SSR in exons might be an important mechanism in generating functional variation of proteins in the *N. crassa*. Using statistical analyses, we concluded that there are both genome-wide and local effects in size variation of SSRs. Since genetic mobile elements are inactive in the most of *N. crassa *genome, the detected size variation of SSRs cannot be explained by transposable elements as demonstrated in other systems. Considering their high PIC values, SSRs are good genetic markers for intra-species populations of *N. crassa*. However, since the polymorphism level is locus specific, more thorough empirical characterizations of size variability of SSRs across the genome are necessary to increase their efficiency as molecular markers.

## Methods

### SSR analysis in the Neurospora genome sequence

The 39.2 Mb *Neurospora *genome sequence, release 7, was downloaded from the Broad Institute [70], and was analyzed to identify SSRs. We utilized the "tandem repeat finder" program [71]. We used stringent cut-off parameters as follows: matching weight = 2, mismatching penalty = 7, indel penalty = 7, match probability = 80, indel probability = 10, minimum alignment score to report = 50, and maximum period size to report = 6. From the analysis, we selected 2749 SSRs and subsequently categorized the SSRs by unit size and repeat motif in different genomic locations. In our study, the genomic location categories were intergenic and genic (exon and intron) regions. Each of the SSRs was considered as unique and was subsequently classified according to theoretically possible combinations in each SSR. For example, (AC)_n _is equivalent to (CA)_n_, (TG)_n_, and (GT)_n_, while (AGC)_n _is equivalent to (GCA)_n_, (CAG)_n_, (CTG)_n_, and (TGC)_n_. Lastly, we determined the abundance of each SSR motif and unit size in the different genomic regions by normalizing the size of the corresponding genomic region. To describe the abundance of SSRs in different genomic region, we chose to use the "relative abundance", which is calculated by dividing the number of SSRs by mega base-pair (MB) of sequences in our analyses.

### SSR markers

To characterize the overall pattern of polymorphism of the SSRs in the *Neurospora *genome, we strived to select SSRs randomly from the *Neurospora *genome. We divided the genome into 250 kb windows and selected SSRs randomly within each window. A total of 164 SSR loci consisting of di- to hexa-SSRs with various sequence motifs were chosen for further analysis. The scatter plot of the selected markers showed that they were evenly distributed in the genome (Data not shown). With the SSRs selected, we designed oligos using Primer3 software (Whitehead Institute for Biomedical Research, Boston, USA) in flanking sequence to amplify the targeted SSR loci. The range of the annealing temperatures in each primer set was between 50°C and 60°C and the primer pairs yielded amplification products between 100 and 350 bp.

For semi-automated genotyping analysis, the 5' M13 sequence was attached to a forward primer in order to incorporate a florescent dye into the PCR product. Fluorescent dye labelled M13 forward primer and a marker specific reverse primer were used to generate fluorescent-labelled PCR product as previously described [72]. The composition of the PCR master mix was prepared as described in Cho et al. [73], and the PCR profile was modified from Schuelke as follows [72]. The basic profile was: 5 min at 94°C, 30 cycles of 30 sec at 94°C, 45 sec at 55°C, 1 min at 72°C, and 25 cycles of 15 sec at 94°C, 30 sec at 53°C, 1 min at 72°C, and 10 min at 72°C for final extension. Fluorescent-labelled PCR products for SSR loci were multiplexed with regard to each molecular weight and fluorescent dye. Each multiplexing set of primers was called a panel. One panel consisted of 12–15 SSR marker sets. The multiplexed PCR products were analyzed by an ABI 3730 (Applied Biosystems) according to the manufacturer's instructions. Allele sizes of SSR loci were determined using Genemapper3.0^® ^(Applied Biosystems).

### Estimation of predicted amino acid repeats encoded by exonic tri-nt SSRs

We generated the predicted amino acid sequences with an assumption that exonic tri-nt SSR sequences had an equal chance to be translated in all the possible reading frames of the tri-nt repeats. For example, SSR sequences GCTGCTGCTGCTGCTGCT can be translated in three different frames: 1) GCT GCT GCT GCT GCT GCT, which will be translated into Ala-Ala-Ala-Ala-Ala-Ala, 2) CTG CTG CTG CTG CTG CTG, which will be translated into Leu-Leu-Leu-Leu-Leu-Leu, and 3) TGC TGC TGC TGC TGC TGC, which will be translated into Cys-Cys-Cys-Cys-Cys-Cys. Only one of the three possible reading frames would be used to generate the "observed" amino acid repeats.

### Evaluation of polymorphism

The measurement of the allelic diversity or polymorphism information content (PIC) value was first described by Botstein et al. [74] and modified by Anderson et al. [75]. PIC was defined as the probability that two randomly chosen copies of gene will be different alleles different within a population. The formula for the PIC value applied in our study was as follows:

$${\text{PIC}}_{\text{i}}=1-\sum_{\text{j}=1}^{\text{n}}{\text{P}}_{\text{ij}}^{2}$$

where Pij represents the frequency of the jth allele for marker i, and summation extends over n alleles. The allelic polymorphism of the 162 SSR markers in the seven natural accessions, FGSC#2223, FGSC#4825, FGSC#4720, FGSC#4715, FGSC#3223, FGSC#4724, and FGSC#2478, were calculated following the formula. The genome structure of seven *N. crassa *strains are divergent and not related among each other (unrooted tree analysis, minimum pair-wise dissimilarity = 0.91).

### Genetic mapping analysis

The 564 F1 progenies (188 F1 haploid progeny from each line-cross, Table 7) were genotyped to determine the linkage maps for each cross. Genetic linkage maps of each population were constructed using two different algorithms, Map Manger QTX v. 0.3 [76] and GMENDEL v.3.0 [77] with the Kosambi mapping function [78]. Using Map manager, the initial linkage grouping was performed using the Double Haploid option with a threshold level of *P *= 0.001. Subsequently, Monte Carlo simulation with 500 iterations was used to test the marker locus order generated by GMENDEL.

### Statistical Analysis

Let Y denote a frequency and L the associated length, then the (relative) abundance is given by A = Y/L. Our modelling strategy incorporates the frequency and length information by assuming that the counts are Poisson random variables with expected values proportional to their associated lengths. If E denotes the expected value of a count, then the expected rate is R = E/L. A log-linear model is used to describe how these rates vary as a function of SST type, genomic region category, and chromosome. The fit of a particular model can be assessed by comparing the empirical abundance values, A, to maximum likelihood estimates of expected rates which satisfy the model assumptions, using the model deviance statistic,

$$D=2\sum Y\log\frac{Y}{\hat{E}}$$

where the summation is over all 266 cells in the 19 × 7 × 2 contingency table. Our most general model has the form,

log *R *= *α *+ *T *+ *C *+ *G *+ *T *× *G*,

or equivalently,

log *E *= log *L *+ *α *+ *T *+ *C *+ *G *+ *T *× *G*,

where T denotes the main effect of SSR type, C denotes the effect of chromosome, G is the main effect of genomic location category, and T × G allows for type by region interaction, that is, differential type effects by genomic category.

## Availability and Requirements

All data presented in the current report are freely available via our webpage, .

## List of abbreviations

SSRs, Simple sequence repeats; nt, nucleotide; AST, abundant SSR types; PIC, Polymorphic Index Content; AAR, amino acid repeats; GO, gene ontology.

## Authors' contributions

QS, JP and YL generated the bioinformatic data. TK analyzed bioinformatical data and experimental data. JB, HGG, and TK performed statistical analyses. KL conceived of the project and participated in its design. TK and KL wrote the manuscript. All authors read and approved the final manuscript.

## Supplementary Material

Additional file 1 List of 2749 SSR loci in the Neurospora crassa genome Click here for file

Additional file 2 GO analysis for proteins containing amino-acid repeats Click here for file

Additional file 3 Abundant SSR types (AST) in the *Neurospora crassa *genome Click here for file

Additional file 4 The distribution of the randomly selected SSRs by the unit number Click here for file

Additional file 5 The physical location and PIC values of 131 SSR loci in the *Neurospora crassa *genome Click here for file

Additional file 6 Comparison of PIC values of the AC/CA SSR type in two different population sizes Click here for file

Additional file 7 Three hypotheses for the size variation of SSRs Click here for file

Additional file 8 The list of 33 SSR loci for statistical analyses Click here for file

Additional file 9 The marker quality of the mapped SSR loci Click here for file
