# Supplementary material for: Simple sequence repeats in Neurospora crassa: distribution, polymorphism and evolutionary inference
Source: BMC Genomics. 2008 Jan 23;9:31. doi: 10.1186/1471-2164-9-31 (PMC2257937; doi:10.1186/1471-2164-9-31)
Supplement: Additional file 1 — List of 2749 SSR loci in the Neurospora crassa genome [file 1471-2164-9-31-S1.pdf]

No	Chromosome	Contig number	Position within contig	Unit Sequence	Unit length	Repeat number[b]
1	3	1	55403 ~ 55448	GAA	3	15.3
2	3	1	105699 ~ 105724	TC	2	13
3	3	1	109231 ~ 109259	ACA	3	9.7
4	3	1	117844 ~ 117875	GTCA	4	8
5	3	1	122640 ~ 122687	TCG	3	16
6	3	1	221811 ~ 221875	CCT	3	21.7
7	3	1	229721 ~ 229758	TGC	3	12.7
8	3	1	270856 ~ 270881	TGG	3	8.7
9	3	1	282363 ~ 282398	TG	2	18
10	3	1	346699 ~ 346744	CCTT	4	11.3
11	3	1	402189 ~ 402223	TCGG	4	8.8
12	3	1	413489 ~ 413558	AAGAA	5	14.4
13	3	1	449075 ~ 449099	GAT	3	8.3
14	3	1	473241 ~ 473317	CAACAG	6	12.8
15	3	1	550042 ~ 550096	GAA	3	18
16	3	1	575481 ~ 575521	CAT	3	13.7
17	3	1	576029 ~ 576062	TA	2	17
18	3	1	614830 ~ 614855	CAG	3	8.7
19	3	1	631979 ~ 632013	AG	2	17.5
20	3	1	634584 ~ 634609	GCA	3	8.7
21	3	1	653516 ~ 653568	TATTAC	6	8.8
22	3	1	659083 ~ 659111	AAG	3	9.7
23	3	1	692695 ~ 692726	GAA	3	10.7
24	3	1	730317 ~ 730381	GCCTGT	6	10.8
25	3	1	730845 ~ 730888	AGC	3	14.7
26	3	1	742867 ~ 742919	GGAA	4	13.5
27	3	1	743138 ~ 743185	AAG	3	16
28	3	1	743372 ~ 743397	GTT	3	8.7
29	3	1	750394 ~ 750428	TTTC	4	8.8
30	3	1	756204 ~ 756266	GAC	3	21
31	3	1	838136 ~ 838171	ACTG	4	9
32	3	1	856001 ~ 856027	GT	2	13.5
33	3	1	898184 ~ 898214	CA	2	15.5
34	3	1	939113 ~ 939144	GTT	3	10.7
35	3	1	956374 ~ 956488	ACA	3	38.3
36	3	1	998567 ~ 998606	AAC	3	13.3
37	3	1	999095 ~ 999163	TG	2	34.5
38	3	1	1008983 ~ 1009010	GCT	3	9.3
39	3	1	1009966 ~ 1010002	ACA	3	12.3
40	3	1	1018147 ~ 1018206	TGT	3	20
41	3	1	1024444 ~ 1024480	GGTA	4	9.3
42	3	1	1058321 ~ 1058346	CGG	3	8.7
43	3	1	1099914 ~ 1099970	TGTTT	5	11.4
44	3	1	1101594 ~ 1101638	GTT	3	15
45	3	1	1135176 ~ 1135226	CAA	3	17
46	3	1	1160765 ~ 1160816	AC	2	26
47	3	1	1211852 ~ 1211877	AAG	3	8.7
48	3	1	1227821 ~ 1227924	GAAAGA	6	17.3
49	3	1	1272386 ~ 1272428	GTC	3	14.3
50	3	1	1278051 ~ 1278090	GTG	3	13.3
51	3	1	1299750 ~ 1299794	GGAT	4	11.3
52	3	1	1334561 ~ 1334664	TGT	3	34.7
53	3	1	1384234 ~ 1384261	CTT	3	9.3
54	3	1	1387768 ~ 1387792	TGA	3	8.3
55	3	1	1440829 ~ 1440858	AGC	3	10
56	3	1	1442820 ~ 1442859	CAG	3	13
57	3	1	1452588 ~ 1452625	TGA	3	12.7
58	3	1	1453937 ~ 1453980	TG	2	22
59	3	1	1500560 ~ 1500605	TACA	4	11.5
60	3	1	1521350 ~ 1521474	TGT	3	41.7
61	3	1	1560143 ~ 1560191	ACTGG	5	9.8
62	3	1	1686727 ~ 1686769	CCT	3	14
63	1	2	88248 ~ 88299	GAG	3	17.3
64	1	2	116974 ~ 116998	CA	2	12.5
65	1	2	158910 ~ 158946	TTGG	4	9.3
66	1	2	277495 ~ 277591	GTT	3	32.3
67	1	2	283443 ~ 283467	CTT	3	8.3
68	1	2	304034 ~ 304089	GA	2	28
69	1	2	328861 ~ 328894	GTT	3	11.7
70	1	2	380469 ~ 380523	TTCC	4	13.8
71	1	2	470382 ~ 470423	CTC	3	14
72	1	2	498629 ~ 498664	TG	2	18
73	1	2	524605 ~ 524640	CTTC	4	9
74	1	2	547868 ~ 547906	TGA	3	13
75	1	2	549706 ~ 549738	CAG	3	11
76	1	2	584053 ~ 584086	GCA	3	11.3
77	1	2	598789 ~ 598826	ACCT	4	9
78	1	2	681593 ~ 681626	GTC	3	11.3
79	1	2	700491 ~ 700535	CAC	3	15
80	1	2	711489 ~ 711526	TGT	3	12.7
81	1	2	720540 ~ 720592	ATTAAT	6	8.3
82	1	2	743251 ~ 743285	CAT	3	11.7
83	1	2	795657 ~ 795714	AGGT	4	14.3
84	1	2	839521 ~ 839554	TC	2	17
85	1	2	839649 ~ 839673	CAT	3	8.3
86	1	2	852135 ~ 852171	GAAT	4	9.3
87	1	2	882110 ~ 882179	CAACAC	6	11.7
88	1	2	907228 ~ 907276	CATC	4	12.3
89	1	2	910242 ~ 910336	AGA	3	31.7
90	1	2	926896 ~ 926935	GAA	3	13.3
91	1	2	935933 ~ 935966	TGA	3	11
92	1	2	936391 ~ 936417	AG	2	13.5

No	e	number	within	Sequence	Unit length	number[b]
93	1	2	949447	GTG	3	10
94	1	2	979890	GAG	3	9.7
95	1	2	1056474	AAG	3	20
96	1	2	1069560	AGGT	4	9.5
97	1	2	1128706	TTGTT	5	9
98	1	2	1135880	AAAAG	5	18
99	1	2	1183638	TTTTC	5	9.8
100	1	2	1187253	CTT	3	11.7
101	1	2	1195144	CAT	3	12.3
102	1	2	1210104	GAGGTT	6	13.7
103	1	2	1221742	CAG	3	15
104	1	2	1270927	GTT	3	48.7
105	1	2	1308015	TACC	4	9
106	1	2	1365054	CAA	3	15.7
107	1	2	1395789	GAT	3	8.7
108	1	2	1436756	GTA	3	10.3
109	1	2	1473958	CAAGCA	6	11.3
110	1	2	1491148	GGA	3	10.7
111	1	2	1518514	GGA	3	9.7
112	1	2	1525384	CAG	3	9.3
113	1	2	1567227	CAT	3	9.3
114	1	2	1588539	ACA	3	12
115	1	2	1588998	CAA	3	14.3
116	1	2	1589234	GATT	4	8.3
117	1	2	1591183	TCT	3	12.3
118	1	2	1648894	TCT	3	29
119	1	2	1660013	CAA	3	20.7
120	1	3	70913	TCC	3	9
121	1	3	176319	AGC	3	8.7
122	1	3	196857	CAA	3	10.7
123	1	3	204655	GTGCA	5	8.4
124	1	3	232968	CAG	3	15.7
125	1	3	282434	TTTCCC	6	14.2
126	1	3	343379	GACT	4	11.3
127	1	3	355693	CGT	3	9.7
128	1	3	481326	CTGTCT	6	12.8
129	1	3	540601	CAA	3	8.3
130	1	3	584848	CAGGCT	6	8.3
131	1	3	588511	TGA	3	12.7
132	1	3	610355	GGA	3	12.7
133	1	3	624801	CTG	3	11
134	1	3	652064	ACG	3	13.7
135	1	3	663364	TCTTCG	6	13.3
136	1	3	685297	GAT	3	8.7
137	1	3	692771	GA	2	16.5
138	1	3	705464	AAC	3	14.3
139	1	3	711509	AAAG	4	14.3
140	1	3	712065	TGT	3	8.3
141	1	3	731009	TGT	3	10.7
142	1	3	773862	TCC	3	25.7
143	1	3	778758	TGC	3	9.3
144	1	3	786698	AAC	3	14.7
145	1	3	814010	ACC	3	8.3
146	1	3	877653	CTT	3	13.7
147	1	3	878589	GACT	4	8.3
148	1	3	896119	AC	2	25
149	1	3	939736	AC	2	24
150	1	3	944661	TTG	3	11.3
151	1	3	959906	CT	2	12.5
152	1	3	970413	GGA	3	12.7
153	1	3	1069237	GCT	3	28
154	1	3	1071122	CTT	3	18.3
155	1	3	1084166	TGG	3	10.7
156	1	3	1100983	TGT	3	13
157	1	3	1106604	AAGAC	5	8
158	1	3	1149995	TG	2	16
159	1	3	1208856	GGT	3	42
160	1	3	1218828	CAA	3	9.7
161	1	3	1247127	GGT	3	14.3
162	1	3	1247252	AGG	3	17
163	1	3	1258522	TCAG	4	9.8
164	1	3	1258836	GAA	3	11
165	1	3	1259042	TC	2	40.5
166	1	3	1263660	TGC	3	10.7
167	1	3	1267483	TGT	3	32.7
168	1	3	1285970	CCA	3	16.3
169	1	3	1302430	CT	2	12.5
170	1	3	1307340	TGT	3	20
171	6	4	75705	TAA	3	14.7
172	6	4	90977	CTAACC	6	13.5
173	6	4	136416	CTTT	4	10.5
174	6	4	159046	CAC	3	10
175	6	4	205445	CTG	3	11
176	6	4	216634	TCC	3	14.7
177	6	4	230441	TGC	3	12.3
178	6	4	234835	GCG	3	9.3
179	6	4	258331	GAA	3	23.3
180	6	4	293472	GTG	3	10.3
181	6	4	307925	AC	2	18
182	6	4	312106	TCTG	4	8.3
183	6	4	342430	TCT	3	9.7
184	6	4	347731	CTG	3	10.3

No	Chromosome	Contig number	Position within contig	Unit Sequence	Unit length	Repeat number[b]
185	6	4	351806 ~ 351861	ATCCGA	6	10
186	6	4	393129 ~ 393260	AGTA	4	33
187	6	4	402399 ~ 402433	TGG	3	11.7
188	6	4	412470 ~ 412501	TGC	3	10.7
189	6	4	415757 ~ 415800	CTT	3	14.7
190	6	4	430119 ~ 430153	GGA	3	11.7
191	6	4	447268 ~ 447297	GGT	3	10
192	6	4	484855 ~ 484890	TGT	3	12
193	6	4	490251 ~ 490280	GTT	3	10
194	6	4	595016 ~ 595060	TTG	3	15
195	6	4	596110 ~ 596175	TG	2	33
196	6	4	614597 ~ 614663	CCA	3	21.7
197	6	4	619845 ~ 619896	CAA	3	17.3
198	6	4	623700 ~ 623724	GAT	3	8.3
199	6	4	748060 ~ 748091	CAA	3	10.7
200	6	4	795125 ~ 795174	GAA	3	16.7
201	6	4	955937 ~ 955962	TCC	3	8.7
202	6	4	962304 ~ 962329	TCC	3	8.7
203	6	4	972152 ~ 972189	TGG	3	12.7
204	6	4	972600 ~ 972632	AGT	3	11
205	6	4	990653 ~ 990682	GGA	3	10
206	6	4	1022466 ~ 1022511	TTGG	4	11
207	6	4	1123850 ~ 1123923	CAT	3	24.7
208	2	5	21984 ~ 22009	CCT	3	8.7
209	2	5	24522 ~ 24587	CTTGAT	6	11.2
210	2	5	25169 ~ 25198	TG	2	15.5
211	2	5	68331 ~ 68363	TCT	3	11
212	2	5	69683 ~ 69708	TGT	3	8.7
213	2	5	71580 ~ 71610	GCT	3	10.3
214	2	5	96260 ~ 96285	GCG	3	8.7
215	2	5	98995 ~ 99022	TGG	3	9.3
216	2	5	145016 ~ 145053	TCC	3	12.7
217	2	5	154663 ~ 154688	GGA	3	8.7
218	2	5	167054 ~ 167110	CAGCAA	6	9.5
219	2	5	169720 ~ 169754	TGG	3	11.7
220	2	5	170024 ~ 170076	GGT	3	17.7
221	2	5	175701 ~ 175793	GCA	3	31
222	2	5	186178 ~ 186208	AGG	3	10.3
223	2	5	186298 ~ 186339	ACG	3	14
224	2	5	188548 ~ 188579	CGT	3	10.7
225	2	5	222527 ~ 222637	TACTGA	6	18.5
226	2	5	224236 ~ 224278	CAA	3	14.3
227	2	5	245461 ~ 245493	ACA	3	11
228	2	5	382773 ~ 382805	GT	2	16.5
229	2	5	424445 ~ 424483	TGC	3	13
230	2	5	443637 ~ 443732	GAT	3	32
231	2	5	459498 ~ 459523	CA	2	13
232	2	5	469488 ~ 469522	CGT	3	11.7
233	2	5	523288 ~ 523331	TCT	3	14.7
234	2	5	526384 ~ 526460	TG	2	36.5
235	2	5	532023 ~ 532101	GATG	4	20
236	2	5	574134 ~ 574161	CTG	3	9.3
237	2	5	609846 ~ 609877	AGC	3	10.7
238	2	5	637830 ~ 637856	GCA	3	9
239	2	5	681653 ~ 681695	ATC	3	14.3
240	2	5	702594 ~ 702688	AAG	3	31.7
241	2	5	720520 ~ 720544	ACA	3	8.3
242	2	5	723347 ~ 723377	CTG	3	10.3
243	2	5	743678 ~ 743710	GGA	3	11
244	2	5	806875 ~ 806901	GCC	3	9
245	2	5	810081 ~ 810115	GAG	3	11.7
246	2	5	833653 ~ 833728	GTT	3	25.3
247	2	5	866247 ~ 866290	TC	2	22.5
248	2	5	874855 ~ 874899	AC	2	22.5
249	2	5	884955 ~ 884984	AAC	3	10
250	2	5	886610 ~ 886635	GAA	3	8.7
251	2	5	904656 ~ 904692	AAC	3	12
252	2	5	914661 ~ 914699	AC	2	19.5
253	2	5	928640 ~ 928669	TGG	3	10
254	2	5	928760 ~ 928813	AG	2	27
255	2	5	1049810 ~ 1049851	GTAGT	5	8
256	2	5	1051494 ~ 1051540	AAAGAG	6	8
257	1	6	28954 ~ 28989	CTC	3	12
258	1	6	66556 ~ 66597	ACA	3	14
259	1	6	71593 ~ 71625	AG	2	16.5
260	1	6	119953 ~ 119980	CTG	3	9.3
261	1	6	182807 ~ 182847	TG	2	20.5
262	1	6	200004 ~ 200161	CAA	3	52.7
263	1	6	255912 ~ 255936	TTG	3	8.3
264	1	6	256165 ~ 256227	ATCCCC	6	10.5
265	1	6	285607 ~ 285649	GAG	3	14.3
266	1	6	311750 ~ 311800	GCA	3	17
267	1	6	322593 ~ 322621	AG	2	14.5
268	1	6	343952 ~ 343982	ACC	3	10.3
269	1	6	347257 ~ 347286	GT	2	15
270	1	6	395527 ~ 395570	CACG	4	11
271	1	6	450290 ~ 450315	TC	2	13
272	1	6	493915 ~ 493940	TGA	3	8.7
273	1	6	662756 ~ 662788	GA	2	17.5
274	1	6	672909 ~ 672947	TGC	3	13
275	1	6	725296 ~ 725359	TGT	3	21.7
276	1	6	876242 ~ 876266	TGC	3	8.3

No	e	number	within	Sequence	Unit length	number[b]
277	1	6	902923	AAAAG	5	9.6
278	1	7	44701	GAGAT	5	9.2
279	1	7	44867	ACA	3	9
280	1	7	70651	AAGA	4	10.5
281	1	7	116474	GT	2	14.5
282	1	7	129630	AC	2	16.5
283	1	7	142894	GCA	3	8.7
284	1	7	188893	TCC	3	10.7
285	1	7	235552	TACC	4	9
286	1	7	243545	ACA	3	11.3
287	1	7	414186	TCT	3	15.3
288	1	7	432876	CAG	3	11.3
289	1	7	456979	CTC	3	12.7
290	1	7	465480	CAA	3	11.7
291	1	7	550949	GAT	3	16.7
292	1	7	621991	CAGCAA	6	61.7
293	1	7	636632	AC	2	18
294	1	7	686033	TTG	3	9
295	1	7	695412	GAG	3	12.7
296	1	7	696319	CCTA	4	8
297	1	7	702490	TAA	3	18.3
298	1	7	715670	CA	2	23.5
299	1	7	720818	GAA	3	12.3
300	1	7	733528	AAC	3	10.3
301	1	7	734023	CTC	3	9
302	1	7	734405	CAG	3	11
303	1	7	734541	CTAC	4	11.3
304	1	7	748523	GCA	3	16.3
305	1	7	748729	TTC	3	13.7
306	1	7	749939	CGCAG	5	12
307	1	7	751385	GCT	3	8.3
308	1	7	770863	CAT	3	9.7
309	1	7	774620	CAG	3	8.3
310	1	7	793673	CAA	3	10.3
311	1	7	795253	TGC	3	8.7
312	1	7	798128	GTG	3	12.7
313	1	7	807037	ACAT	4	12
314	1	7	812781	CAA	3	24.3
315	1	7	823745	TGAC	4	21
316	1	7	823909	AAGC	4	14.5
317	1	7	867534	GCT	3	14.7
318	1	7	868029	CTG	3	10.7
319	1	7	868862	TTG	3	10.3
320	1	7	869591	GTC	3	8.7
321	1	7	870040	CAG	3	15
322	1	7	870131	TTG	3	9
323	1	7	870684	GTG	3	28.3
324	1	7	887127	TC	2	48.5
325	1	7	889005	GAG	3	10.3
326	1	7	892223	TGC	3	8.7
327	1	7	892374	GGT	3	11.3
328	1	7	906495	CTTCTG	6	9.8
329	1	7	906624	TGG	3	12.3
330	1	7	907125	ATAGC	5	9
331	1	7	913468	CAC	3	8.7
332	1	7	915560	TTC	3	12
333	1	7	921726	TGT	3	10.3
334	1	7	923333	AGG	3	8.3
335	1	7	933767	CGTCCC	6	8.2
336	1	7	939883	CATC	4	15.3
337	1	7	940286	AGTTC	5	11.8
338	1	7	956824	GTC	3	8.3
339	1	7	970976	CAG	3	8.7
340	1	7	971303	CAG	3	10.7
341	1	7	972173	CAGCAA	6	10.8
342	1	7	972819	CAA	3	11.3
343	1	7	983775	TTCC	4	8.8
344	1	7	985650	CAGT	4	9.5
345	2	8	25083	TGT	3	11
346	2	8	41361	GTACTA	6	8.8
347	2	8	53547	CGA	3	10
348	2	8	171857	TG	2	19.5
349	2	8	214872	AGC	3	15
350	2	8	215339	TCC	3	10
351	2	8	225814	GGT	3	13.3
352	2	8	239006	GGCTCT	6	11.5
353	2	8	239729	TCCT	4	10.8
354	2	8	252818	GAG	3	9
355	2	8	257137	AGTCA	5	10.4
356	2	8	338550	TTG	3	82
357	2	8	344967	GA	2	17
358	2	8	355125	ATCCA	5	8
359	2	8	355351	TGA	3	9
360	2	8	365534	ACC	3	19.7
361	2	8	366493	CCTA	4	10.5
362	2	8	397466	AGG	3	10.7
363	2	8	442067	TG	2	21
364	2	8	445986	CAT	3	16.3
365	2	8	464108	GGA	3	12
366	2	8	474591	GTC	3	10
367	2	8	494759	GAA	3	11.3
368	2	8	495005	CTT	3	12.3

No	Chromosome	Contig number	Position within contig	Unit Sequence	Unit length	Repeat number[b]
369	2	8	495435 ~ 495465	AGT	3	10.3
370	2	8	499393 ~ 499423	GCT	3	10.3
371	2	8	499499 ~ 499532	GT	2	17
372	2	8	499830 ~ 499856	GTA	3	9
373	2	8	501252 ~ 501279	TG	2	14
374	2	8	502641 ~ 502678	CAT	3	12.3
375	2	8	505428 ~ 505461	TAGG	4	8.5
376	2	8	510428 ~ 510462	GTT	3	11.7
377	2	8	512812 ~ 512847	TGG	3	12
378	2	8	522557 ~ 522619	TTTTC	5	12.4
379	2	8	552296 ~ 552320	CCT	3	8.3
380	2	8	557761 ~ 557791	CAA	3	10.3
381	2	8	590238 ~ 590281	TGT	3	14.7
382	2	8	602669 ~ 602703	CAGC	4	8.8
383	2	8	602904 ~ 602950	AAGAC	5	9.6
384	2	8	636053 ~ 636122	CT	2	35
385	2	8	636471 ~ 636522	TACC	4	13
386	2	8	647542 ~ 647585	CAG	3	14.7
387	2	8	647877 ~ 647960	ACA	3	28
388	2	8	663522 ~ 663548	GGA	3	9
389	2	8	663836 ~ 663886	ATGGTT	6	8.7
390	2	8	672896 ~ 672931	AT	2	18
391	2	8	674522 ~ 674576	TCCCAC	6	8.8
392	2	8	697381 ~ 697405	TCG	3	8.3
393	2	8	700106 ~ 700137	GCT	3	10.7
394	2	8	704193 ~ 704253	CTT	3	20.3
395	2	8	731019 ~ 731055	GACC	4	9.3
396	2	8	732697 ~ 732731	TGC	3	11.7
397	2	8	741658 ~ 741724	GCAGG	5	13.6
398	2	8	799958 ~ 799993	GTT	3	12
399	2	8	809205 ~ 809242	ACA	3	12.7
400	2	8	817463 ~ 817516	TGGA	4	13.5
401	2	8	822697 ~ 822726	GTT	3	10
402	2	8	823170 ~ 823209	GTT	3	13.3
403	2	8	827597 ~ 827645	ATGTC	5	9.8
404	2	8	837013 ~ 837098	CAAGCT	6	14.3
405	2	8	837994 ~ 838019	CAG	3	8.7
406	2	8	839894 ~ 839935	GGTA	4	10.3
407	2	8	844761 ~ 844837	TGG	3	25.7
408	2	8	858849 ~ 859003	GACTT	5	31.8
409	2	8	859057 ~ 859088	GAAG	4	8
410	2	8	860683 ~ 860717	TAGG	4	8.8
411	2	8	869759 ~ 869826	AAC	3	22
412	2	8	875259 ~ 875285	TC	2	13.5
413	2	8	884971 ~ 885017	TC	2	23.5
414	2	8	889789 ~ 889815	TGT	3	9
415	2	8	904693 ~ 904742	TCG	3	17.3
416	2	8	906480 ~ 906533	GCATA	5	10.8
417	1	9	26212 ~ 26247	AAAG	4	9.5
418	1	9	125930 ~ 125984	AGG	3	18.3
419	1	9	168620 ~ 168670	AATCAG	6	8.5
420	1	9	261776 ~ 261829	ACAT	4	13.5
421	1	9	266760 ~ 266801	CTAC	4	10.5
422	1	9	347032 ~ 347065	ATC	3	11.3
423	1	9	412838 ~ 412890	TCC	3	17.7
424	1	9	427042 ~ 427095	TCACGG	6	9
425	1	9	478497 ~ 478537	CGTT	4	10.3
426	1	9	503362 ~ 503393	AGG	3	11
427	1	9	503771 ~ 503796	GCA	3	8.7
428	1	9	548484 ~ 548509	GTG	3	8.7
429	1	9	559910 ~ 559946	TC	2	19.5
430	1	9	629657 ~ 629683	TCC	3	9
431	1	9	640786 ~ 640818	CAG	3	11
432	1	9	648688 ~ 648740	GTG	3	17.7
433	1	9	679283 ~ 679345	ACA	3	21
434	1	9	688053 ~ 688105	GTT	3	17.7
435	1	9	761405 ~ 761441	TG	2	18.5
436	1	9	773366 ~ 773405	ATC	3	13.3
437	1	9	798368 ~ 798400	GCT	3	11
438	1	9	814636 ~ 814666	CTG	3	10.3
439	1	9	867775 ~ 867842	GAG	3	22.7
440	1	9	881547 ~ 881586	CCTG	4	10
441	1	9	882685 ~ 882716	AC	2	16
442	1	9	895086 ~ 895147	TTTCT	5	13.8
443	1	9	897461 ~ 897505	GCTT	4	11.3
444	1	9	911494 ~ 911531	CATC	4	9.5
445	1	9	924185 ~ 924219	GGT	3	11.7
446	1	9	953976 ~ 954025	TGC	3	16.7
447	1	9	954265 ~ 954296	GGA	3	10.7
448	1	9	959593 ~ 959688	TCC	3	32
449	7	10	63062 ~ 63104	CCTA	4	10.3
450	7	10	79761 ~ 79815	AAAATA	6	9.2
451	7	10	85836 ~ 85877	CTACA	5	8
452	7	10	86049 ~ 86091	CCAT	4	10.3
453	7	10	97220 ~ 97261	CCT	3	14
454	7	10	129139 ~ 129182	CAA	3	14.7
455	7	10	135238 ~ 135285	TGT	3	16
456	7	10	169551 ~ 169591	TCTGA	5	8.2
457	7	10	170383 ~ 170431	GGAGA	5	9.8
458	7	10	174470 ~ 174507	TGA	3	12.7
459	7	10	177000 ~ 177058	GACTCG	6	9.8
460	7	10	178411 ~ 178445	AC	2	17.5

No	e	number	within	Sequence	Unit length	number[b]
461	7	10	184622	GA	2	18.5
462	7	10	208810	CAT	3	12.3
463	7	10	209019	AG	2	34
464	7	10	224190	CAC	3	15.3
465	7	10	224763	ACA	3	13
466	7	10	237731	GGA	3	12.7
467	7	10	247052	TACA	4	11.8
468	7	10	252120	TG	2	32.5
469	7	10	279322	GTG	3	8.3
470	7	10	312767	ATCA	4	12
471	7	10	316509	TCG	3	11
472	7	10	321205	AGA	3	13.7
473	7	10	417644	GAT	3	11.7
474	7	10	426669	CAG	3	10
475	7	10	432910	AGC	3	9.3
476	7	10	458845	GCT	3	8.7
477	7	10	465004	CAG	3	10.7
478	7	10	466698	CAA	3	10.7
479	7	10	466991	TCT	3	17.7
480	7	10	520237	GT	2	24
481	7	10	537050	CCCT	4	9
482	7	10	619493	CTG	3	12.3
483	7	10	619813	TGC	3	11.7
484	7	10	623941	ACT	3	18.7
485	7	10	624091	CTG	3	14.3
486	7	10	627451	CAG	3	13.3
487	7	10	627841	CAA	3	11.3
488	7	10	636531	TGT	3	17
489	7	10	640359	CT	2	22
490	7	10	644955	ACTAC	5	10
491	7	10	678518	GCA	3	9.3
492	7	10	722745	CT	2	27
493	7	10	723363	AGG	3	10.7
494	7	10	730824	CTC	3	15
495	7	10	763335	AT	2	14.5
496	7	10	796983	GAC	3	16.7
497	7	10	822814	TC	2	13
498	7	10	902748	AGGT	4	35.5
499	5	11	38455	CT	2	15.5
500	5	11	83482	CCTGA	5	8.2
501	5	11	103678	CTGC	4	18.3
502	5	11	160173	CA	2	26
503	5	11	185782	ACC	3	11
504	5	11	225870	TCT	3	27.3
505	5	11	244450	ACC	3	12.3
506	5	11	250068	TG	2	13
507	5	11	288860	CCA	3	9
508	5	11	320499	GCCA	4	10
509	5	11	329720	ACG	3	8.7
510	5	11	340225	AAC	3	9
511	5	11	341276	TGAC	4	14
512	5	11	371335	AGGA	4	51.3
513	5	11	378014	TC	2	15
514	5	11	486018	CAG	3	8.3
515	5	11	550884	CTC	3	10
516	5	11	562878	GCT	3	10.7
517	5	11	659246	AGG	3	16.3
518	5	11	690119	TGC	3	8.3
519	5	11	717196	GAA	3	12.3
520	5	11	754276	ACGA	4	14.5
521	5	11	764878	CTC	3	14.7
522	5	11	789813	GGT	3	9.3
523	5	11	806428	GAG	3	10.7
524	5	11	809931	CAA	3	10
525	5	11	835843	CAA	3	11.7
526	5	11	845592	CAC	3	12.3
527	5	11	885275	AG	2	33.5
528	5	11	892877	TGT	3	11.7
529	5	11	892980	TTG	3	17.3
530	6	12	19904	TTA	3	50
531	6	12	39397	CCTCAC	6	11.2
532	6	12	79382	TC	2	27.5
533	6	12	115511	GTC	3	8.3
534	6	12	136083	TAA	3	8.7
535	6	12	181117	CCT	3	12
536	6	12	210961	AAG	3	19.7
537	6	12	218098	AAG	3	25
538	6	12	285860	AGC	3	9.3
539	6	12	291779	TCA	3	13
540	6	12	354709	AGC	3	11.3
541	6	12	374148	GAA	3	12.7
542	6	12	394418	AC	2	13.5
543	6	12	404606	ACA	3	11
544	6	12	422920	CAC	3	8.3
545	6	12	423051	GGTA	4	13.3
546	6	12	434289	TGC	3	9.7
547	6	12	484398	GAA	3	20
548	6	12	503959	CTC	3	8.7
549	6	12	518073	TGC	3	10
550	6	12	525578	GCT	3	9
551	6	12	554101	AGGT	4	19.5
552	6	12	634199	AGC	3	12.7

No	Chromosome	Contig number	Position within contig	Unit Sequence	Unit length	Repeat number[b]
553	6	12	640023 ~ 640058	TTC	3	12
554	6	12	739484 ~ 739520	TG	2	18.5
555	6	12	825653 ~ 825694	CAC	3	14
556	6	12	826148 ~ 826505	GGAAGA	6	61.2
557	6	12	828558 ~ 828646	TCT	3	29.7
558	6	12	841127 ~ 841168	GGAAG	5	8.4
559	6	12	842227 ~ 842264	GGA	3	12.7
560	5	13	9463 ~ 9491	CCG	3	9.7
561	5	13	18959 ~ 19016	GATG	4	14.5
562	5	13	23727 ~ 23760	TTC	3	11
563	5	13	30949 ~ 30982	GAG	3	11.3
564	5	13	51968 ~ 51992	AGC	3	8.3
565	5	13	73017 ~ 73079	GGGAA	5	12
566	5	13	99789 ~ 99818	GGT	3	10
567	5	13	111380 ~ 111409	CAC	3	10
568	5	13	112232 ~ 112263	TTG	3	10.7
569	5	13	158965 ~ 159082	AGTA	4	29.5
570	5	13	164269 ~ 164298	AG	2	15
571	5	13	170658 ~ 170813	AGGT	4	39
572	5	13	184470 ~ 184502	CCG	3	11
573	5	13	215478 ~ 215509	TCC	3	10.7
574	5	13	262433 ~ 262469	CTC	3	12.3
575	5	13	264664 ~ 264705	AGGT	4	10.3
576	5	13	267442 ~ 267470	TTG	3	9.7
577	5	13	292980 ~ 293034	AG	2	27.5
578	5	13	293188 ~ 293230	GTG	3	15
579	5	13	294067 ~ 294104	TC	2	18
580	5	13	306735 ~ 306782	AACTCG	6	8
581	5	13	342183 ~ 342233	ACA	3	17
582	5	13	361740 ~ 361779	TTC	3	13.3
583	5	13	392741 ~ 392773	GGT	3	11
584	5	13	400818 ~ 400954	TACC	4	34.3
585	5	13	466107 ~ 466139	AGTG	4	8.3
586	5	13	483074 ~ 483107	CAAT	4	8.5
587	5	13	485167 ~ 485206	ACAGC	5	8
588	5	13	487055 ~ 487079	AC	2	12.5
589	5	13	514079 ~ 514116	GTAT	4	9.5
590	5	13	521169 ~ 521206	ACC	3	12.7
591	5	13	531135 ~ 531163	GCT	3	9.7
592	5	13	538939 ~ 538972	CTG	3	11.3
593	5	13	645842 ~ 645916	CAA	3	25
594	5	13	658348 ~ 658379	TG	2	16
595	5	13	665583 ~ 665617	GTCG	4	8.8
596	5	13	738623 ~ 738654	AGG	3	10.7
597	5	13	758598 ~ 758631	CCA	3	11.3
598	5	13	765292 ~ 765333	GAA	3	13.3
599	5	13	815497 ~ 815526	CAC	3	10
600	5	13	820933 ~ 820966	GAAG	4	8.5
601	5	13	828101 ~ 828131	TG	2	15.5
602	5	13	831848 ~ 831892	CAT	3	15
603	5	13	848805 ~ 848833	CTG	3	9.7
604	5	13	863126 ~ 863157	CAG	3	10.7
605	5	14	99312 ~ 99350	CCA	3	13
606	5	14	147430 ~ 147467	TCC	3	12.7
607	5	14	164030 ~ 164069	TGA	3	13.3
608	5	14	166889 ~ 166952	TGTAG	5	12.8
609	5	14	190015 ~ 190091	TCTGGC	6	12.8
610	5	14	225000 ~ 225029	CA	2	15
611	5	14	264615 ~ 264639	GCA	3	8.3
612	5	14	275448 ~ 275544	AC	2	48.5
613	5	14	289919 ~ 289968	TTC	3	17
614	5	14	292643 ~ 292668	TGC	3	8.7
615	5	14	301632 ~ 301666	GTT	3	11
616	5	14	327016 ~ 327048	ACA	3	11
617	5	14	340003 ~ 340028	GAA	3	8.7
618	5	14	425087 ~ 425111	TTG	3	8.3
619	5	14	442602 ~ 442626	TGT	3	8.3
620	5	14	449098 ~ 449139	GCT	3	14
621	5	14	500331 ~ 500383	GGA	3	17.7
622	5	14	564948 ~ 565014	AG	2	33.5
623	5	14	593867 ~ 593918	ATATTA	6	8.2
624	5	14	631448 ~ 631477	TCA	3	10
625	5	14	689349 ~ 689381	CTC	3	11.3
626	5	14	696659 ~ 696684	TCT	3	8.7
627	5	14	721666 ~ 721753	TG	2	44
628	5	14	730224 ~ 730452	ACA	3	76.3
629	5	14	731790 ~ 731824	GAG	3	11.7
630	5	14	736414 ~ 736476	CGT	3	21
631	5	14	737566 ~ 737595	TTC	3	10
632	5	14	741593 ~ 741654	GAT	3	20.7
633	5	14	743488 ~ 743516	GAG	3	9.7
634	5	14	748233 ~ 748279	CCA	3	15.7
635	5	15	20814 ~ 20839	CAG	3	8.7
636	5	15	75351 ~ 75385	AGC	3	11.7
637	5	15	94654 ~ 94678	ATA	3	8.3
638	5	15	260377 ~ 260414	GTT	3	12.7
639	5	15	308987 ~ 309013	TGA	3	9
640	5	15	338321 ~ 338353	GA	2	16.5
641	5	15	406380 ~ 406411	TGT	3	10.7
642	5	15	507153 ~ 507341	CTCCTT	6	31.5
643	6	16	59833 ~ 59907	AAG	3	26
644	6	16	95572 ~ 95620	AG	2	24.5

No	e	number	within	Sequence	Unit length	number[b]
645	6	16	111208	AGA	3	13
646	6	16	112536	GT	2	12.5
647	6	16	130047	ACG	3	8.7
648	6	16	147507	AC	2	15.5
649	6	16	147751	AC	2	18
650	6	16	194482	AGGT	4	10.3
651	6	16	205237	TATG	4	28.3
652	6	16	231244	GCT	3	15.7
653	6	16	245519	GT	2	21.5
654	6	16	250849	CCA	3	11
655	6	16	313810	AC	2	12.5
656	6	16	331414	GTT	3	8.3
657	6	16	356747	GGT	3	10
658	6	16	372987	AAG	3	11.3
659	6	16	382147	GCT	3	9
660	6	16	385870	TGG	3	12.7
661	6	16	386619	CAG	3	12.3
662	6	16	394852	CCT	3	20.3
663	6	16	425630	CTT	3	8.3
664	6	16	425753	GGA	3	9.7
665	6	16	440331	CAA	3	10
666	6	16	464189	TTG	3	20
667	6	16	489131	CAC	3	10
668	6	16	489400	CCGA	4	8.5
669	6	16	501828	ACA	3	12.7
670	6	16	506272	TGC	3	10
671	6	16	511929	GTT	3	12
672	6	16	524454	TTG	3	12.3
673	6	16	555736	TCC	3	16.7
674	6	16	572218	GATGCC	6	12.8
675	6	16	585564	TGTGC	5	14.4
676	6	16	586283	AGGGC	5	8.2
677	6	16	587159	AGTCC	5	8.8
678	6	16	611638	CAGGCA	6	8.7
679	6	16	630814	GCT	3	13
680	6	16	684564	GA	2	14.5
681	3	17	76453	GAG	3	9
682	3	17	251751	TAA	3	13
683	3	17	288982	CAC	3	8.7
684	3	17	291021	TGA	3	21
685	3	17	296502	TTCG	4	8
686	3	17	302348	GCAG	4	9.8
687	3	17	320837	CTC	3	11.3
688	3	17	321174	ATG	3	10
689	3	17	323662	GTC	3	12.3
690	3	17	331176	TCC	3	10
691	3	17	334077	CTAC	4	9.3
692	3	17	397658	ACG	3	12
693	3	17	442341	ACG	3	11.7
694	3	17	460485	CAA	3	11.3
695	3	17	460844	TGG	3	9.3
696	3	17	513395	GGA	3	8.7
697	3	17	533972	GAG	3	10
698	3	17	548797	GGT	3	10.3
699	3	17	554298	GGTA	4	9.8
700	3	17	565382	ATCC	4	8
701	3	17	582216	GAT	3	8.7
702	3	17	631491	TGAC	4	9.5
703	4	18	7686 ~ 7717	TC	2	17
704	4	18	7869 ~ 7907	AGCC	4	9.5
705	4	18	8606 ~ 8632	GCA	3	9
706	4	18	14445	TCT	3	11
707	4	18	15934	GGC	3	9.3
708	4	18	54433	CACCAT	6	11.3
709	4	18	61434	CCAT	4	8.8
710	4	18	69529	TG	2	19
711	4	18	88564	TTG	3	11.7
712	4	18	88885	CATC	4	8
713	4	18	91952	GTG	3	9.7
714	4	18	145349	GTG	3	9
715	4	18	205045	AGAAAA	6	9.3
716	4	18	252099	TTAGGG	6	8.7
717	4	18	308571	AGGA	4	8
718	4	18	347432	GTT	3	51
719	4	18	380079	TTG	3	9
720	4	18	393275	TGT	3	11.7
721	4	18	455218	CTGCAC	6	8.2
722	4	18	480671	TAT	3	102
723	4	18	487783	GCACCT	6	11.7
724	4	18	542116	CT	2	19
725	4	18	551709	TATAC	5	16
726	4	18	566420	TTA	3	105.3
727	4	18	607743	ATA	3	145.7
728	4	18	608933	AAAAAG	6	13.8
729	4	19	13895	ACA	3	16.7
730	4	19	36951	ACA	3	14.3
731	4	19	51363	GTT	3	18.3
732	4	19	102537	TCCA	4	8.8
733	4	19	129017	GAC	3	13
734	4	19	138822	TCT	3	11.3
735	4	19	154640	ACA	3	9.7
736	4	19	169154	GAT	3	12

No	Chromosome	Contig number	Position within contig	Unit Sequence	Unit length	Repeat number[b]
737	4	19	224852 ~ 224891	AAG	3	13.3
738	4	19	228991 ~ 229016	AGA	3	8.7
739	4	19	244156 ~ 244193	GAC	3	12.7
740	4	19	252789 ~ 252831	GGT	3	14
741	4	19	253645 ~ 253677	GGT	3	11
742	4	19	270621 ~ 270662	CAA	3	14
743	4	19	275870 ~ 275895	GTC	3	8.7
744	4	19	290107 ~ 290160	CTGGTG	6	9
745	4	19	290348 ~ 290391	TGG	3	14.7
746	4	19	298895 ~ 298939	ATGG	4	11.3
747	4	19	312942 ~ 312995	TACC	4	13.3
748	4	19	323447 ~ 323477	TG	2	15.5
749	4	19	349891 ~ 349915	AC	2	12.5
750	4	19	355497 ~ 355528	GTT	3	10.7
751	4	19	358736 ~ 358773	TATG	4	9.5
752	4	19	375261 ~ 375301	GT	2	21.5
753	4	19	376420 ~ 376446	CAC	3	9
754	4	19	381840 ~ 381878	ACC	3	13
755	4	19	398608 ~ 398664	ACAT	4	14.8
756	4	19	399216 ~ 399276	ACG	3	20.3
757	4	19	424233 ~ 424267	TCC	3	11.7
758	4	19	431326 ~ 431355	ACC	3	10
759	4	19	431509 ~ 431544	GCT	3	12
760	4	19	439820 ~ 439856	CAA	3	12.3
761	4	19	442304 ~ 442376	AGA	3	24.3
762	4	19	461781 ~ 461898	AAGG	4	29.5
763	4	19	462199 ~ 462277	CTGT	4	19.8
764	4	19	473619 ~ 473657	AAG	3	13
765	4	19	481576 ~ 481610	GGT	3	11.7
766	4	19	490958 ~ 490988	CACT	4	8
767	4	19	495223 ~ 495298	AGTTC	5	15.2
768	4	19	495907 ~ 495940	TACC	4	8.3
769	4	19	499078 ~ 499114	GGT	3	12.3
770	4	19	502817 ~ 502843	GTG	3	9
771	4	19	520961 ~ 520995	TGGT	4	8.8
772	4	19	526500 ~ 526525	TGA	3	8.7
773	4	19	534616 ~ 534668	CCAT	4	14
774	4	19	534736 ~ 534787	ATAC	4	13
775	4	19	534976 ~ 535092	GATA	4	29.3
776	4	19	552090 ~ 552128	CGT	3	13
777	4	19	579114 ~ 579172	TTTTC	5	11.8
778	4	19	580638 ~ 580678	ACC	3	13.7
779	4	19	614198 ~ 614222	GCG	3	8.3
780	4	19	634852 ~ 634915	GTCT	4	16.3
781	4	19	648391 ~ 648468	GGAT	4	19.5
782	4	19	648625 ~ 648662	GAG	3	12.3
783	4	20	31954 ~ 31984	CAA	3	10.3
784	4	20	34807 ~ 34837	AG	2	15.5
785	4	20	74760 ~ 74788	TGT	3	9.7
786	4	20	128399 ~ 128428	GCT	3	10
787	4	20	153811 ~ 153842	CAGC	4	8
788	4	20	204217 ~ 204261	AAC	3	14.7
789	4	20	221029 ~ 221073	CTG	3	15
790	4	20	240606 ~ 240631	GAA	3	8.7
791	4	20	256193 ~ 256291	TCACCA	6	16.5
792	4	20	333585 ~ 333609	TCG	3	8.3
793	4	20	365114 ~ 365138	AG	2	12.5
794	4	20	474799 ~ 474823	TCC	3	8.3
795	4	20	483775 ~ 483800	TGA	3	8.7
796	4	20	495040 ~ 495066	GTG	3	9
797	4	20	505127 ~ 505208	TGT	3	27.3
798	4	20	514711 ~ 514745	GT	2	17.5
799	4	20	547543 ~ 547569	CAC	3	9
800	4	20	557878 ~ 557902	GAT	3	8.3
801	4	20	590521 ~ 590552	AGC	3	10.7
802	4	20	610906 ~ 610936	GT	2	15.5
803	4	20	617949 ~ 617973	TTC	3	8.3
804	4	20	620975 ~ 620999	AGC	3	8.3
805	4	20	621911 ~ 621950	AAG	3	13.3
806	4	20	636038 ~ 636086	GCAACT	6	8.2
807	4	20	640169 ~ 640200	GTT	3	10.7
808	4	20	643984 ~ 644018	ACAT	4	9.3
809	7	21	15171 ~ 15383	TAC	3	71.7
810	7	21	16214 ~ 16255	TGT	3	14
811	7	21	80385 ~ 80410	CCT	3	8.7
812	7	21	90564 ~ 90600	AAC	3	12.3
813	7	21	106328 ~ 106357	GTG	3	10
814	7	21	106583 ~ 106608	TTG	3	8.7
815	7	21	110026 ~ 110055	TGA	3	10
816	7	21	115974 ~ 116001	AGC	3	9.3
817	7	21	157800 ~ 157838	TGA	3	13
818	7	21	161867 ~ 161908	TGC	3	14
819	7	21	161988 ~ 162017	TGC	3	9.7
820	7	21	164587 ~ 164611	CTT	3	8.3
821	7	21	188948 ~ 189003	TCGCA	5	10.4
822	7	21	198589 ~ 198645	AAC	3	19
823	7	21	218147 ~ 218197	CGTCCC	6	8.5
824	7	21	234800 ~ 234825	CGG	3	8.7
825	7	21	275287 ~ 275330	GA	2	22
826	7	21	546937 ~ 546982	GTT	3	15.7
827	7	21	556615 ~ 556655	TTG	3	13.7
828	7	21	570316 ~ 570340	TGG	3	8.3

No	e	number	within	Sequence	Unit length	number[b]
829	7	21	581460	TTG	3	8.7
830	7	21	596952	ACC	3	11
831	7	21	615259	ACA	3	60
832	6	22	23563	AC	2	33
833	6	22	34149	CAT	3	23.3
834	6	22	46129	ACC	3	9.3
835	6	22	46616	CAG	3	11.3
836	6	22	46731	AGTG	4	13.5
837	6	22	54783	GAA	3	8.7
838	6	22	106844	GTAG	4	106.8
839	6	22	118065	TTC	3	8.7
840	6	22	122141	AAAAG	5	8.2
841	6	22	128170	ACC	3	8.7
842	6	22	128527	CCTA	4	22.5
843	6	22	147886	GTAA	4	12.5
844	6	22	263376	GAT	3	10.3
845	6	22	311804	CAT	3	11
846	6	22	411693	AGAGGA	6	13.2
847	6	22	447798	GTCCAG	6	9.5
848	6	22	460401	ACC	3	10.3
849	6	22	487393	TGT	3	18.7
850	6	22	507594	CAG	3	12.7
851	6	22	518321	TTG	3	9
852	6	22	520240	CCT	3	12.7
853	6	22	536488	GAAA	4	9
854	6	22	548624	GGTA	4	10.8
855	6	22	571785	CGGT	4	8.8
856	7	23	4553 ~ 4592	TAT	3	13.3
857	7	23	52275	GTG	3	9.3
858	7	23	54597	TACC	4	9.8
859	7	23	97619	CACTG	5	10.4
860	7	23	169140	GTG	3	16.7
861	7	23	179844	TGG	3	10
862	7	23	190198	CAA	3	16.3
863	7	23	194352	TCA	3	40
864	7	23	245348	ATCTC	5	10.8
865	7	23	248578	TCAA	4	8.3
866	7	23	259594	GCAGGA	6	11.8
867	7	23	291509	TTGATG	6	10.5
868	7	23	296734	ACC	3	11.7
869	7	23	330104	GGA	3	11.7
870	7	23	332657	GCA	3	9
871	7	23	341467	GCA	3	8.3
872	7	23	343587	GTA	3	27.7
873	7	23	347403	TCT	3	12.7
874	7	23	368260	AAG	3	9.3
875	7	23	381343	TTG	3	9
876	7	23	419247	GTC	3	10
877	7	23	425414	ACACA	5	8
878	7	23	431003	AGC	3	14
879	7	23	440155	CCT	3	10.7
880	7	23	460180	GGA	3	14
881	7	23	494301	GAA	3	10
882	7	23	500278	CAG	3	11
883	7	23	537174	GGAT	4	8
884	5	24	18245	CCT	3	13.3
885	5	24	23959	TAT	3	32.7
886	5	24	96690	AG	2	52.5
887	5	24	97082	AC	2	13.5
888	5	24	115739	ACAAT	5	10.4
889	5	24	121187	CGA	3	10
890	5	24	139287	GTC	3	10.3
891	5	24	195291	CCA	3	10.7
892	5	24	202726	TGC	3	9
893	5	24	235621	GTA	3	9.3
894	5	24	269623	AAG	3	9
895	5	24	270765	GT	2	19.5
896	5	24	272452	CTG	3	11
897	5	24	290135	GGTA	4	15.5
898	5	24	292230	CAG	3	12.7
899	5	24	292924	CAATGG	6	10.5
900	5	24	296572	CACTAC	6	14.2
901	5	24	302277	ACA	3	32.3
902	5	24	323549	GAGT	4	8.5
903	5	24	327522	CAGCAC	6	9.3
904	5	24	330293	CTG	3	14
905	5	24	340782	GAG	3	12
906	5	24	346574	GTG	3	8.3
907	5	24	352805	TCA	3	10.3
908	5	24	362676	TTTC	4	8.8
909	5	24	362936	AGTC	4	8.8
910	5	24	364652	CTG	3	8.7
911	5	24	367959	TGT	3	10.7
912	5	24	396537	TGC	3	12.7
913	5	24	399405	CAA	3	14.3
914	5	24	436899	TTCT	4	8
915	5	24	464072	AGG	3	18.7
916	5	24	493238	TC	2	14.5
917	3	25	25581	TTC	3	13.7
918	3	25	43863	CAA	3	8.3
919	3	25	82318	TCTT	4	8
920	3	25	106402	TTC	3	11

No	Chromosome	Contig number	Position within contig	Unit Sequence	Unit length	Repeat number[b]
921	3	25	107106 ~ 107168	CAA	3	21.3
922	3	25	108072 ~ 108096	ACC	3	8.3
923	3	25	126474 ~ 126506	GAGG	4	8.3
924	3	25	161323 ~ 161396	CT	2	37.5
925	3	25	161776 ~ 161838	TGCGAC	6	10.3
926	3	25	167895 ~ 167935	ACCAC	5	8.4
927	3	25	169082 ~ 169155	CAG	3	24.7
928	3	25	171590 ~ 171615	GAC	3	8.7
929	3	25	199625 ~ 199674	TC	2	26.5
930	3	25	210503 ~ 210540	CAA	3	12.7
931	3	25	213152 ~ 213177	AAC	3	8.7
932	3	25	265539 ~ 265570	GGA	3	10.7
933	3	25	289671 ~ 289709	AGC	3	13
934	3	25	299829 ~ 299950	CATA	4	30.5
935	3	25	300349 ~ 300385	CAGT	4	9.3
936	3	25	317630 ~ 317669	CAG	3	13.3
937	3	25	391113 ~ 391158	CAA	3	15.3
938	3	25	405798 ~ 405857	CTAC	4	15
939	3	25	416564 ~ 416589	CT	2	13
940	3	25	423416 ~ 423452	TGTT	4	9.3
941	3	25	437246 ~ 437273	GCT	3	9.3
942	4	26	55559 ~ 55585	GCA	3	9
943	4	26	63216 ~ 63248	TTC	3	11.3
944	4	26	122799 ~ 122831	TCA	3	11
945	4	26	129541 ~ 129575	TCC	3	11.7
946	4	26	149789 ~ 149816	GGT	3	9.3
947	4	26	160116 ~ 160157	CAG	3	14
948	4	26	161813 ~ 161848	GCA	3	12
949	4	26	171173 ~ 171211	GTT	3	13
950	4	26	237869 ~ 237904	AGC	3	12
951	4	26	244842 ~ 244866	ACC	3	8.3
952	4	26	340105 ~ 340154	GGA	3	16.7
953	4	26	346981 ~ 347019	GCA	3	13
954	4	26	434579 ~ 434626	GT	2	24
955	3	27	4055 ~ 4090	CCA	3	12
956	3	27	14620 ~ 14673	CCT	3	18.3
957	3	27	18453 ~ 18549	CT	2	48.5
958	3	27	33515 ~ 33546	GATG	4	8
959	3	27	34569 ~ 34598	GCA	3	10
960	3	27	95594 ~ 95620	AGC	3	9
961	3	27	105758 ~ 105957	TTC	3	66.7
962	3	27	121599 ~ 121632	CTT	3	11.3
963	3	27	139770 ~ 139807	CAA	3	12.7
964	3	27	153363 ~ 153415	TGCC	4	13.3
965	3	27	157485 ~ 157512	AC	2	14
966	3	27	171978 ~ 172033	CAAAA	5	11
967	3	27	185385 ~ 185425	GCAG	4	10.3
968	3	27	189900 ~ 189944	ATGG	4	11.3
969	3	27	238013 ~ 238056	AAC	3	14.7
970	3	27	253642 ~ 253667	GTC	3	8.7
971	3	27	286944 ~ 286999	AGTCC	5	11.2
972	3	27	333278 ~ 333309	AGAC	4	8
973	3	27	368574 ~ 368598	CGG	3	8.3
974	3	27	368832 ~ 368868	CCA	3	12.3
975	3	27	380491 ~ 380528	TGT	3	12.7
976	3	27	400773 ~ 400817	GAT	3	15
977	3	27	420473 ~ 420508	CTG	3	12
978	4	28	1986 ~ 2056	AGATGT	6	11
979	4	28	56547 ~ 56571	GGA	3	8.3
980	4	28	57284 ~ 57340	TGTACC	6	9.5
981	4	28	86122 ~ 86156	TGC	3	11.7
982	4	28	107242 ~ 107273	ATC	3	10.7
983	4	28	138179 ~ 138224	ACA	3	14.7
984	4	28	138649 ~ 138692	TGT	3	14.7
985	4	28	140079 ~ 140104	AG	2	13
986	4	28	141787 ~ 141849	CTC	3	21
987	4	28	175061 ~ 175128	TG	2	34
988	4	28	198245 ~ 198275	CTG	3	10.3
989	4	28	206169 ~ 206236	GAA	3	22.7
990	4	28	238694 ~ 238731	GTTG	4	9.5
991	4	28	274102 ~ 274145	GGTA	4	11
992	4	28	303120 ~ 303197	TCG	3	26
993	4	28	321120 ~ 321154	GTT	3	11.7
994	4	28	404742 ~ 404769	GGT	3	9.3
995	4	28	407379 ~ 407414	CTG	3	12
996	1	29	13096 ~ 13146	CTC	3	17.3
997	1	29	23835 ~ 23882	TCG	3	16
998	1	29	30295 ~ 30329	AGG	3	11.7
999	1	29	34248 ~ 34282	ACC	3	11.7
1000	1	29	63515 ~ 63548	TGG	3	11.3
1001	1	29	84087 ~ 84155	GGA	3	23
1002	1	29	166482 ~ 166535	CAG	3	18
1003	1	29	259278 ~ 259304	GGC	3	9
1004	1	29	336104 ~ 336128	TAA	3	8.3
1005	1	29	339225 ~ 339265	TAT	3	13.7
1006	1	29	397564 ~ 397696	TCC	3	44.3
1007	2	30	38751 ~ 38791	TAT	3	13.7
1008	2	30	44746 ~ 44779	TC	2	17
1009	2	30	67349 ~ 67379	TGA	3	10.3
1010	2	30	71096 ~ 71129	GGTA	4	8
1011	2	30	104275 ~ 104315	TAG	3	13.7
1012	2	30	153807 ~ 153851	TTG	3	15

No	e	number	within	Sequence	Unit length	number[b]
1013	2	30	216377	CTCCGG	6	9.5
1014	2	30	245723	CAA	3	18.3
1015	2	30	245842	CAGCAA	6	10.8
1016	2	30	246139	GAT	3	15.7
1017	2	30	248717	TGC	3	29
1018	2	30	255808	TTG	3	13.3
1019	2	30	256219	GTGG	4	8.5
1020	2	30	299809	CTT	3	16
1021	2	30	304712	GCA	3	8.3
1022	2	30	352172	TGT	3	15
1023	2	30	361272	AAC	3	79.7
1024	2	30	386968	TTACGA	6	11.3
1025	2	30	402861	GGAATA	6	18.3
1026	5	31	1784 ~ 1952	AGG	3	56.3
1027	5	31	92820	CAA	3	10
1028	5	31	106764	GCT	3	11
1029	5	31	157885	GTC	3	8.3
1030	5	31	189232	GTC	3	8.3
1031	5	31	235375	TTC	3	12
1032	5	31	298235	CAA	3	17.3
1033	5	31	316578	ATGCC	5	8.2
1034	7	32	24717	CTC	3	14.7
1035	7	32	25424	TGC	3	11.7
1036	7	32	79878	AAT	3	8.7
1037	7	32	89597	TCT	3	28.3
1038	7	32	90856	TCT	3	12
1039	7	32	95410	CTG	3	9.7
1040	7	32	104945	GGT	3	15.7
1041	7	32	168569	GTC	3	20.7
1042	7	32	179798	TGC	3	9
1043	7	32	210585	GTC	3	9
1044	7	32	210866	TCT	3	10
1045	7	32	255785	TGT	3	9.3
1046	7	32	262346	GGGAAA	6	13.3
1047	7	32	276592	TATATT	6	15
1048	7	32	299389	GTA	3	11
1049	7	32	300046	GGT	3	8.3
1050	7	32	305931	CGA	3	10.7
1051	7	32	306249	CAA	3	9
1052	7	32	321005	GATGGG	6	8.7
1053	7	32	324854	CCAG	4	9.8
1054	7	32	351449	CAGG	4	9.8
1055	7	32	359249	CAC	3	14
1056	7	32	372232	TAGG	4	8.5
1057	2	33	7576 ~ 7606	CAG	3	10.3
1058	2	33	10805	CTC	3	16.3
1059	2	33	37321	CCT	3	9.3
1060	2	33	71267	TCC	3	11
1061	2	33	76795	GCT	3	8.3
1062	2	33	77879	CTC	3	9.7
1063	2	33	126685	GAG	3	9
1064	2	33	129411	TCT	3	19.7
1065	2	33	132520	TGC	3	9
1066	2	33	133579	TTG	3	11.3
1067	2	33	142334	GAT	3	10.7
1068	2	33	144787	GT	2	13.5
1069	2	33	255067	GAA	3	19.7
1070	2	33	259160	AC	2	17.5
1071	2	33	274417	GCT	3	9.3
1072	2	33	285266	CAT	3	8.3
1073	2	33	300253	GTT	3	15.3
1074	2	33	301880	TGC	3	11
1075	2	33	304444	ACT	3	12.7
1076	2	33	341953	TC	2	63
1077	2	33	346820	GCTG	4	9.8
1078	2	33	357636	GGGT	4	10.5
1079	2	33	373469	AC	2	25
1080	6	34	2 ~ 168	ACCCTA	6	27.8
1081	6	34	98260	TTA	3	8.7
1082	6	34	165334	TTTCT	5	19.6
1083	6	34	189230	AATCC	5	9
1084	6	34	241935	TACC	4	19
1085	6	34	242969	AAAG	4	20.3
1086	6	34	260684	GGT	3	10
1087	6	34	260956	GGC	3	10.7
1088	6	34	282991	CTCA	4	8.8
1089	6	34	284724	CCTT	4	9
1090	6	34	322835	TC	2	12.5
1091	6	34	348043	TGG	3	17.7
1092	4	35	42658	CTGGGA	6	8.7
1093	4	35	90969	CAA	3	13
1094	4	35	100106	GATT	4	11
1095	4	35	100361	TTTCT	5	9.6
1096	4	35	134438	GGAT	4	19.8
1097	4	35	139587	GT	2	15.5
1098	4	35	150436	TTG	3	9.7
1099	4	35	168202	CAA	3	8.7
1100	4	35	180711	TGG	3	13
1101	4	35	191727	GGT	3	9.3
1102	4	35	193697	TG	2	127.5
1103	4	35	193774	TA	2	13.5
1104	4	35	199345	TATG	4	8.5

No	Chromosome	Contig number	Position within contig	Unit Sequence	Unit length	Repeat number[b]
1105	4	35	213953 ~ 213981	CGA	3	9.7
1106	4	35	223121 ~ 223152	TTCC	4	8
1107	4	35	224393 ~ 224431	TG	2	19.5
1108	4	35	245873 ~ 245918	AGGGAA	6	8
1109	4	35	251198 ~ 251240	GCA	3	14.3
1110	4	35	272232 ~ 272257	CAG	3	8.7
1111	4	35	276969 ~ 277010	CCA	3	14
1112	4	35	280244 ~ 280268	GAA	3	8.3
1113	4	35	294293 ~ 294334	TGT	3	14.3
1114	4	35	338507 ~ 338554	TCCA	4	12
1115	4	35	340008 ~ 340032	GGT	3	8.3
1116	4	35	340772 ~ 340807	TTG	3	12
1117	4	35	360248 ~ 360389	TCT	3	47.7
1118	4	36	24527 ~ 24554	GGA	3	9.3
1119	4	36	46198 ~ 46280	GAA	3	27.7
1120	4	36	72580 ~ 72611	CCT	3	10.7
1121	4	36	73051 ~ 73093	AGA	3	14
1122	4	36	159897 ~ 159961	CCTG	4	16.3
1123	4	36	169375 ~ 169466	TTTATA	6	14.3
1124	4	36	205969 ~ 205997	CCT	3	9.7
1125	4	36	260119 ~ 260171	GGA	3	17.7
1126	4	36	305113 ~ 305170	TGGTCG	6	9.7
1127	5	37	7522 ~ 7613	GTT	3	31
1128	5	37	56196 ~ 56226	TCA	3	10
1129	5	37	143021 ~ 143141	TTG	3	40.3
1130	5	37	223562 ~ 223611	GCA	3	16.7
1131	5	37	296569 ~ 296600	CCTG	4	8
1132	5	37	310784 ~ 310809	GCT	3	8.7
1133	1	38	31217 ~ 31245	ACA	3	9.7
1134	1	38	46149 ~ 46215	TCCTTT	6	10.7
1135	1	38	50586 ~ 50619	GA	2	17
1136	1	38	77628 ~ 77668	CTT	3	13.7
1137	1	38	86079 ~ 86106	GCT	3	9.3
1138	1	38	157604 ~ 157643	AAGA	4	10
1139	1	38	226910 ~ 226939	ATA	3	10
1140	1	38	256351 ~ 256377	GAC	3	9
1141	1	38	273170 ~ 273203	AG	2	17
1142	1	38	314327 ~ 314354	AT	2	14
1143	1	39	10510 ~ 10552	CATC	4	10.8
1144	1	39	13946 ~ 13980	TGT	3	11.7
1145	1	39	18966 ~ 18999	TA	2	17
1146	1	39	24626 ~ 24660	TCGT	4	8.8
1147	1	39	24948 ~ 24978	CTC	3	10.3
1148	1	39	57367 ~ 57397	CAG	3	10.3
1149	1	39	58270 ~ 58390	TGT	3	40.3
1150	1	39	76429 ~ 76455	ATC	3	9
1151	1	39	81847 ~ 81887	CCAG	4	10.5
1152	1	39	82234 ~ 82304	GAA	3	24
1153	1	39	98865 ~ 98901	TTC	3	12.3
1154	1	39	137780 ~ 137824	TG	2	22.5
1155	1	39	138307 ~ 138338	GCCT	4	8
1156	1	39	153745 ~ 153800	CGGT	4	14.3
1157	1	39	159700 ~ 159724	AAC	3	8.3
1158	1	39	162005 ~ 162057	CCACCT	6	9.3
1159	1	39	175900 ~ 175941	GGA	3	14
1160	1	39	180270 ~ 180300	TCA	3	10.3
1161	1	39	195178 ~ 195214	AGTA	4	9.3
1162	1	39	206392 ~ 206446	TAAG	4	13.8
1163	1	39	214107 ~ 214154	ATG	3	16
1164	1	39	217959 ~ 217994	TCC	3	12
1165	1	39	244860 ~ 244891	CCA	3	10.7
1166	1	39	247476 ~ 247508	GAT	3	11
1167	1	39	251375 ~ 251408	CATC	4	8.3
1168	1	39	267537 ~ 267564	CA	2	14
1169	1	39	267980 ~ 268011	ACAA	4	8
1170	1	39	303673 ~ 303799	GAAT	4	33.8
1171	3	40	34391 ~ 34416	GAA	3	8.7
1172	3	40	124387 ~ 124417	TC	2	15.5
1173	3	40	133085 ~ 133126	CAG	3	14.3
1174	3	40	133954 ~ 134007	ACAGCA	6	9
1175	3	40	213697 ~ 213734	TAT	3	12.7
1176	3	40	218684 ~ 218749	AG	2	33
1177	3	40	221198 ~ 221272	GTTTT	5	15
1178	3	40	240679 ~ 240725	GTAG	4	11.8
1179	3	40	269119 ~ 269157	GTT	3	13
1180	5	41	98557 ~ 98581	TTG	3	8.3
1181	5	41	109371 ~ 109399	CCG	3	9.7
1182	5	41	125876 ~ 125912	AGGC	4	9.3
1183	5	41	161480 ~ 161532	GAGGAA	6	8.8
1184	5	41	178027 ~ 178065	GGA	3	13
1185	5	41	229996 ~ 230091	ACA	3	32
1186	5	41	237823 ~ 237857	TGG	3	11.7
1187	5	41	243656 ~ 243682	AG	2	13.5
1188	5	41	245613 ~ 245649	TA	2	19
1189	3	42	15494 ~ 15546	TCGCTA	6	8.8
1190	3	42	20061 ~ 20117	CGT	3	19
1191	3	42	31942 ~ 31997	TAGG	4	13.5
1192	3	42	111912 ~ 111948	ATGG	4	9.3
1193	3	42	121593 ~ 121666	TTCG	4	18
1194	3	42	126487 ~ 126514	TGG	3	9.3
1195	3	42	135511 ~ 135546	CTT	3	12
1196	3	42	135857 ~ 135895	CT	2	19.5

No	e	number	within	Sequence	Unit length	number[b]
1197	3	42	135991	TTC	3	9
1198	3	42	149121	CA	2	19
1199	3	42	171174	TTAGGG	6	9.7
1200	3	42	181337	CT	2	15
1201	3	42	208668	GA	2	13
1202	3	42	213323	GAGT	4	9.3
1203	3	42	246972	AAC	3	64
1204	3	42	275836	GTTAGG	6	14.8
1205	3	42	296784	ACT	3	21.3
1206	4	43	582 ~ 640	AT	2	31
1207	4	43	4766 ~ 4811	TTGC	4	11.5
1208	4	43	5332 ~ 5374	GACT	4	10.8
1209	4	43	18713	TGG	3	8.3
1210	4	43	19356	AAG	3	50.7
1211	4	43	20477	TGT	3	13
1212	4	43	24986	TTG	3	13
1213	4	43	42273	AGC	3	11.7
1214	4	43	42518	TC	2	16
1215	4	43	74143	CCTTTC	6	16
1216	4	43	120751	TGC	3	56
1217	4	43	122101	GGC	3	8.3
1218	4	43	139345	TC	2	13
1219	4	43	169648	ACTGGT	6	8.8
1220	4	43	197766	GGT	3	9.3
1221	4	43	222905	GT	2	26
1222	4	43	277143	TAC	3	9.7
1223	4	43	293808	TAC	3	13
1224	2	44	11452	CAA	3	8.3
1225	2	44	40055	AAC	3	9.7
1226	2	44	47354	TA	2	20.5
1227	2	44	47706	AT	2	21.5
1228	2	44	53162	TA	2	18.5
1229	2	44	53513	AT	2	20.5
1230	2	44	55312	TAA	3	26
1231	2	44	71060	GA	2	21.5
1232	2	44	96186	CCA	3	11.7
1233	2	44	115690	GCT	3	8.3
1234	2	44	116161	TGT	3	13.7
1235	2	44	127446	GAG	3	11
1236	2	44	134611	CATCG	5	8.8
1237	2	44	134980	AT	2	19.5
1238	2	44	135665	GATG	4	8.5
1239	2	44	151253	GAA	3	9.7
1240	2	44	151714	GCT	3	8.3
1241	2	44	225533	TGA	3	13.7
1242	2	44	230409	TA	2	18.5
1243	2	44	230760	AT	2	20.5
1244	2	44	232573	TAA	3	31
1245	2	44	233715	TAA	3	19.7
1246	2	44	234172	AAT	3	29.3
1247	2	44	249498	GA	2	21.5
1248	2	44	274624	CCA	3	11.7
1249	2	44	294145	GCT	3	8.3
1250	2	44	294616	TGT	3	13.7
1251	3	45	38360	GAT	3	16
1252	3	45	58220	TCG	3	10.3
1253	3	45	100820	CACCAG	6	17.3
1254	3	45	101257	GTGGGA	6	10
1255	3	45	112638	GTT	3	99.3
1256	3	45	120684	GA	2	16
1257	3	45	127453	GA	2	17.5
1258	3	45	139219	ACG	3	10.3
1259	3	45	140301	GAA	3	11.7
1260	3	45	144520	GAG	3	8.3
1261	3	45	152653	CGCA	4	9.8
1262	3	45	168795	GAAA	4	24.3
1263	3	45	179954	CCCTA	5	12
1264	3	45	180346	AGGGAG	6	12.3
1265	3	45	180616	AAGT	4	8.3
1266	3	45	214915	ACC	3	9.3
1267	3	45	227116	GTAG	4	15
1268	3	45	236676	AACG	4	10.3
1269	3	45	290561	GAG	3	13.7
1270	5	46	31042	CACGA	5	8.8
1271	5	46	43017	ACAT	4	38.3
1272	5	46	44113	TTCT	4	13.8
1273	5	46	53648	TACA	4	8
1274	5	46	76882	GAA	3	33.7
1275	5	46	79175	CCTT	4	9.5
1276	5	46	126111	GGA	3	18.3
1277	5	46	132812	CAA	3	12
1278	5	46	198484	CT	2	33
1279	5	46	209458	ATGG	4	9
1280	5	46	209747	TAGG	4	13.5
1281	5	46	257563	TCTTCC	6	10.3
1282	5	46	296217	CAA	3	76.7
1283	4	47	18651	CCA	3	10
1284	4	47	84670	AGCA	4	10.8
1285	4	47	183332	GCA	3	11.3
1286	4	47	195856	TTG	3	9.7
1287	5	48	27352	ACA	3	14.7
1288	5	48	28008	TGCTGG	6	9

No	Chromosome	Contig number	Position within contig	Unit Sequence	Unit length	Repeat number[b]
1289	5	48	37032 ~ 37056	CAT	3	8.3
1290	5	48	45746 ~ 45790	CAG	3	15
1291	5	48	62915 ~ 62942	GCT	3	9.3
1292	5	48	72107 ~ 72141	GAC	3	11.7
1293	5	48	109113 ~ 109180	ACA	3	22.7
1294	5	48	115956 ~ 116009	GCTAT	5	10.8
1295	5	48	125286 ~ 125449	AAGAGC	6	27.3
1296	5	48	125521 ~ 125677	TC	2	78.5
1297	5	48	138163 ~ 138197	GGT	3	11.3
1298	5	48	140938 ~ 140974	TGCC	4	9.3
1299	5	48	159975 ~ 160001	CAA	3	9
1300	5	48	182465 ~ 182508	TAGG	4	11
1301	5	48	201153 ~ 201214	TAATAG	6	10.8
1302	5	48	248205 ~ 248240	TGAG	4	9
1303	4	49	5274 ~ 5310	TG	2	18.5
1304	4	49	28588 ~ 28708	CCT	3	40.3
1305	4	49	118286 ~ 118318	CTTA	4	8.3
1306	4	49	129062 ~ 129105	GGAAA	5	9.2
1307	4	49	132631 ~ 132663	GAG	3	11
1308	4	49	202513 ~ 202549	ACA	3	12.3
1309	4	49	252722 ~ 252750	CCA	3	9.7
1310	NA[a]	50	19903 ~ 19943	ACTCT	5	8.2
1311	NA	50	22411 ~ 22450	ATT	3	13.3
1312	NA	50	29144 ~ 29175	TTA	3	10.7
1313	NA	50	85822 ~ 85856	TAA	3	11.7
1314	NA	50	105354 ~ 105404	TAGAG	5	10.2
1315	NA	50	141371 ~ 141624	ATA	3	84.7
1316	NA	50	145482 ~ 145549	CCCATA	6	11.3
1317	NA	50	225218 ~ 225278	TAGAG	5	12.2
1318	4	51	129044 ~ 129077	GGA	3	11.3
1319	4	51	173992 ~ 174041	CAA	3	16.7
1320	4	51	249649 ~ 249678	TGC	3	10
1321	7	52	35151 ~ 35197	TACC	4	11.8
1322	7	52	63346 ~ 63370	GAT	3	8.3
1323	7	52	87965 ~ 88008	CCTA	4	10.8
1324	7	52	142198 ~ 142232	TG	2	17.5
1325	7	52	161554 ~ 161598	CA	2	22.5
1326	7	52	165825 ~ 165869	AAGA	4	11.8
1327	7	52	186099 ~ 186205	CAA	3	35.7
1328	7	52	206812 ~ 206864	GGA	3	17.7
1329	7	52	221137 ~ 221170	GGT	3	11.3
1330	7	52	227086 ~ 227191	TTG	3	35.3
1331	7	52	227420 ~ 227449	TTG	3	10
1332	7	52	252450 ~ 252475	AGA	3	8.7
1333	4	53	39921 ~ 39987	AAAAAG	6	11.7
1334	4	53	49920 ~ 49957	CCT	3	12.7
1335	4	53	139486 ~ 139517	CCT	3	10.7
1336	2	54	3523 ~ 3549	AC	2	13.5
1337	2	54	34616 ~ 34642	CTG	3	9
1338	2	54	39259 ~ 39348	ACA	3	30
1339	2	54	53293 ~ 53328	TC	2	18.5
1340	2	54	53982 ~ 54021	TCATA	5	8
1341	2	54	67345 ~ 67384	TGG	3	13.3
1342	2	54	71633 ~ 71667	GTAG	4	8.8
1343	2	54	76582 ~ 76616	AGGT	4	8.8
1344	2	54	76697 ~ 76728	GCTG	4	8.3
1345	2	54	76980 ~ 77010	GCT	3	10.3
1346	2	54	81262 ~ 81287	TGC	3	8.7
1347	2	54	84073 ~ 84102	GCC	3	10
1348	2	54	87535 ~ 87571	CTT	3	12.3
1349	2	54	103578 ~ 103602	TGC	3	8.3
1350	2	54	112532 ~ 112624	TTCCCT	6	15.5
1351	2	54	125528 ~ 125562	AC	2	17.5
1352	2	54	126328 ~ 126352	TCA	3	8.3
1353	2	54	139615 ~ 139673	TGAA	4	14.8
1354	2	54	171553 ~ 171641	TCC	3	29.7
1355	2	54	174837 ~ 174862	CCA	3	8.7
1356	2	54	207690 ~ 207729	GGT	3	13.3
1357	2	54	210836 ~ 210872	ACA	3	12.3
1358	2	54	212930 ~ 212964	GAG	3	11.7
1359	7	55	12873 ~ 12920	TAA	3	16
1360	7	55	27960 ~ 27999	AAT	3	13.3
1361	7	55	35657 ~ 35796	ACTCT	5	28
1362	7	55	125506 ~ 125535	TTA	3	10
1363	7	55	137473 ~ 137597	AAT	3	41.7
1364	7	55	175357 ~ 175403	TAT	3	15.7
1365	1	56	5342 ~ 5366	TA	2	12.5
1366	1	56	5786 ~ 5841	AT	2	29.5
1367	1	56	36569 ~ 36607	ACT	3	13
1368	1	56	61294 ~ 61318	TG	2	12.5
1369	1	56	76716 ~ 76746	CGA	3	10.3
1370	1	56	86816 ~ 86845	TGT	3	10
1371	1	56	88744 ~ 88785	CTTG	4	10.5
1372	1	56	119896 ~ 119944	TAA	3	16.3
1373	1	56	145825 ~ 145858	AAG	3	11.3
1374	2	57	69352 ~ 69404	CA	2	26.5
1375	2	57	79864 ~ 79896	TGTT	4	8.3
1376	2	57	104129 ~ 104172	CCGA	4	11
1377	2	57	104482 ~ 104514	GAC	3	11
1378	2	57	132591 ~ 132641	CGTT	4	12.8
1379	2	57	145859 ~ 145892	GCT	3	11.3
1380	2	57	179064 ~ 179105	CTCAC	5	8.4

No	e	number	within	Sequence	Unit length	number[b]
1381	1	58	61848	TGC	3	11.7
1382	1	58	84106	CAA	3	29
1383	1	58	110887	GACC	4	8.8
1384	1	58	138523	GAG	3	29
1385	1	58	153767	TGT	3	10.7
1386	1	58	158074	GAG	3	11.3
1387	1	58	165511	TAGAGG	6	16.8
1388	1	58	172382	GTG	3	14.7
1389	1	58	173156	CTG	3	19.3
1390	1	58	181626	GTG	3	16.7
1391	1	59	24786	AAGA	4	9.5
1392	1	59	43502	CAGC	4	8.3
1393	1	59	57108	TTG	3	8.3
1394	1	59	96789	TGT	3	12.3
1395	1	59	125016	TGC	3	11
1396	1	59	150739	CAA	3	23.3
1397	1	59	161128	GGC	3	8.3
1398	1	59	162884	GAT	3	12.3
1399	1	59	163056	TGC	3	8.3
1400	4	60	30319	CAA	3	14
1401	4	60	106293	CA	2	32
1402	4	60	153525	AGC	3	10.7
1403	4	60	153780	TGT	3	11.3
1404	4	60	169285	CTT	3	12.3
1405	NA	61	58234	ACTCT	5	11.2
1406	NA	61	62716	CCAATC	6	10.3
1407	NA	61	86790	TTA	3	12
1408	NA	61	130174	AG	2	22
1409	NA	61	130589	TAT	3	10
1410	1	62	8763 ~ 8813	CCT	3	17
1411	1	62	32703	TAA	3	14.7
1412	1	62	53261	ATA	3	16.7
1413	1	62	125394	TAA	3	13
1414	5	63	8603 ~ 8704	TG	2	51
1415	5	63	11384	CGA	3	11
1416	5	63	42282	TAGG	4	10
1417	5	63	65723	TCT	3	9.7
1418	5	63	82467	CGA	3	13.3
1419	5	63	97816	AAG	3	10.3
1420	5	63	115704	CAA	3	17.3
1421	5	63	173099	AGAAAA	6	9.8
1422	5	64	20947	CTG	3	12
1423	5	64	35223	CAC	3	9
1424	5	64	38014	TGG	3	11.3
1425	5	64	42798	TGA	3	14.7
1426	5	64	54478	CT	2	19.5
1427	5	64	55282	AG	2	21.5
1428	5	64	55427	AG	2	16.5
1429	5	64	73891	TACC	4	8.5
1430	5	64	76666	CTG	3	11
1431	5	64	76960	GGA	3	13
1432	5	64	85086	ATCTTC	6	15.2
1433	5	64	93123	GTG	3	9.3
1434	5	64	110563	CGG	3	8.7
1435	5	64	110662	TACT	4	8.3
1436	5	64	134032	CTG	3	10
1437	5	64	137478	CCT	3	16.3
1438	5	64	141328	TCT	3	23.3
1439	5	64	145743	TCGT	4	9.5
1440	5	64	163944	GGCA	4	9
1441	1	65	1 ~ 41	TAT	3	13.7
1442	1	65	20266	CAT	3	8.3
1443	1	65	24382	GTT	3	14.3
1444	1	65	38278	GTG	3	13
1445	1	65	46623	GAG	3	20
1446	1	65	48723	TAGTAC	6	8.3
1447	1	65	76438	CAG	3	10
1448	1	65	80074	GAG	3	12.3
1449	1	65	84621	GAAGA	5	9.8
1450	7	66	53130	TC	2	13
1451	7	66	60677	GGA	3	17.3
1452	7	66	71602	AAC	3	42.7
1453	7	66	71872	AAGAAA	6	13.8
1454	7	66	119506	CCT	3	8.3
1455	7	66	125601	CTC	3	10.3
1456	3	67	1873 ~ 2017	AGG	3	48.3
1457	3	67	24742	CTC	3	9.3
1458	3	67	89664	AC	2	17
1459	3	67	94732	TTG	3	10.3
1460	3	67	115180	GGT	3	8.3
1461	3	67	121059	GTG	3	8.7
1462	2	68	34815	GA	2	22
1463	2	68	48332	TAA	3	9.7
1464	3	69	83807	AGA	3	48.7
1465	3	69	84129	CCAAAG	6	8.3
1466	3	69	84484	TAGG	4	9.5
1467	3	69	85085	TC	2	15
1468	3	69	90793	GTCG	4	8.3
1469	3	69	112425	CTG	3	12.7
1470	3	69	128036	CCT	3	14.3
1471	3	69	134807	CAA	3	12.3
1472	3	69	158547	TTTATA	6	16.5

No	Chromosome	Contig number	Position within contig	Unit Sequence	Unit length	Repeat number[b]
1473	1	70	25572 ~ 25601	GGT	3	10
1474	1	70	29723 ~ 29765	AAGAA	5	8.6
1475	1	70	30523 ~ 30548	TCG	3	8.7
1476	1	70	67709 ~ 67744	TGTA	4	9
1477	1	70	73889 ~ 73925	GA	2	18.5
1478	1	70	85158 ~ 85197	TGCT	4	9.5
1479	1	70	90660 ~ 90714	TGTTTT	6	9.3
1480	1	70	114555 ~ 114586	GTCA	4	8
1481	1	70	115880 ~ 115924	CAGCA	5	9
1482	1	70	117232 ~ 117259	CT	2	14
1483	1	70	141941 ~ 141986	TTGACC	6	8.2
1484	2	71	16446 ~ 16498	TACAC	5	10.6
1485	2	71	19225 ~ 19250	CAA	3	8.7
1486	2	71	21468 ~ 21506	AGGTA	5	8
1487	2	71	25077 ~ 25136	CAGGAA	6	10
1488	2	71	33412 ~ 33442	CAT	3	10.3
1489	2	71	61452 ~ 61491	GTT	3	13.3
1490	2	71	90491 ~ 90516	AGA	3	8.7
1491	1	72	35307 ~ 35340	CTC	3	11.3
1492	1	72	40176 ~ 40240	CGGGTG	6	10.8
1493	1	72	109668 ~ 109706	GGA	3	13
1494	7	73	47462 ~ 47518	TTC	3	19
1495	7	73	64095 ~ 64136	GGAT	4	10.5
1496	7	73	80961 ~ 81010	AGTCAA	6	8.3
1497	7	73	98019 ~ 98045	CCA	3	9
1498	7	73	103836 ~ 103868	GGT	3	11
1499	3	74	2479 ~ 2513	TAT	3	11.7
1500	3	74	6864 ~ 6890	CCA	3	9
1501	3	74	33978 ~ 34002	CAG	3	8.3
1502	3	74	43810 ~ 43875	AAG	3	22
1503	3	74	83161 ~ 83191	TAC	3	10.3
1504	3	74	101040 ~ 101074	TCG	3	11.7
1505	3	74	122079 ~ 122115	GCT	3	12.3
1506	7	75	23294 ~ 23327	AG	2	17.5
1507	7	75	24819 ~ 24941	AAC	3	41
1508	7	75	38314 ~ 38341	CGT	3	9.3
1509	7	75	46689 ~ 46720	ACA	3	10.7
1510	7	75	50049 ~ 50145	AGGA	4	24.3
1511	7	75	51033 ~ 51060	GCA	3	9.3
1512	7	75	87340 ~ 87389	TAATAT	6	8.3
1513	7	75	103361 ~ 103392	GGT	3	10.7
1514	7	75	115097 ~ 115131	ACC	3	11.7
1515	7	75	121929 ~ 121954	TTC	3	8.7
1516	7	75	125079 ~ 125139	TCTTTT	6	9.5
1517	7	76	9575 ~ 9612	AGG	3	12.7
1518	7	76	13668 ~ 13733	ACA	3	21.7
1519	7	76	38546 ~ 38579	ATC	3	11.3
1520	7	76	103231 ~ 103271	ACCGA	5	8
1521	7	76	103594 ~ 103623	CAC	3	10
1522	7	76	104133 ~ 104171	TCT	3	13
1523	7	76	115927 ~ 115966	ACAGG	5	8
1524	7	76	116416 ~ 116453	CCTA	4	9.5
1525	2	77	1 ~ 160	CCCTAA	6	26.7
1526	2	77	36876 ~ 36900	GTC	3	8.3
1527	2	77	57451 ~ 57481	CTC	3	10.3
1528	2	77	58965 ~ 59025	CAA	3	20.3
1529	2	77	67377 ~ 67454	GCCAGA	6	13
1530	2	77	91276 ~ 91304	CAG	3	9.7
1531	2	77	94387 ~ 94415	AGC	3	9.7
1532	7	78	46844 ~ 46906	ACA	3	21
1533	7	78	50060 ~ 50088	TA	2	15
1534	7	78	57835 ~ 57870	TCC	3	12
1535	7	78	117566 ~ 117627	TACC	4	15.5
1536	4	79	7982 ~ 8029	AGAAA	5	9.2
1537	4	79	29004 ~ 29041	TC	2	19
1538	4	79	29576 ~ 29612	GGTG	4	9
1539	4	79	60095 ~ 60140	AAC	3	15.3
1540	4	79	78378 ~ 78412	ACG	3	11.7
1541	4	79	84026 ~ 84060	TGC	3	11.7
1542	4	79	114476 ~ 114644	TTAGGG	6	28.2
1543	NA	80	21580 ~ 21612	GAG	3	11
1544	NA	80	24332 ~ 24372	GAC	3	13.7
1545	NA	80	68314 ~ 68349	CTG	3	12
1546	NA	80	93379 ~ 93413	CAC	3	11.7
1547	NA	80	98139 ~ 98183	AG	2	23
1548	NA	80	98465 ~ 98514	TCTG	4	12.3
1549	2	81	19657 ~ 19707	TTG	3	17
1550	2	81	53923 ~ 53950	AAG	3	9.3
1551	1	82	89779 ~ 89813	CAG	3	11.7
1552	4	83	14144 ~ 14175	GTG	3	10.7
1553	4	83	48260 ~ 48365	AGC	3	35.3
1554	4	83	83125 ~ 83154	CAA	3	10
1555	7	84	88127 ~ 88155	TGG	3	9.7
1556	6	85	10774 ~ 10799	CAA	3	8.7
1557	6	85	29064 ~ 29094	GGT	3	10.3
1558	6	85	36864 ~ 36913	CCA	3	16.3
1559	6	85	39041 ~ 39092	CAA	3	17.3
1560	6	85	46156 ~ 46181	GGT	3	8.7
1561	6	85	50959 ~ 51059	GTG	3	32.7
1562	6	85	54865 ~ 54907	CGT	3	14.3
1563	6	85	69512 ~ 69574	GTAG	4	15.8
1564	1	86	22790 ~ 22866	TTTTC	5	17.2

No	e	number	within	Sequence	Unit length	number[b]
1565	1	86	32344	GCT	3	13.3
1566	1	86	47407	AAC	3	14.3
1567	2	87	45703	TGG	3	31
1568	2	87	46423	TTAGGG	6	8.7
1569	5	88	8868 ~ 8899	TTTC	4	8
1570	5	88	10828	CTT	3	9.3
1571	5	88	26585	TGA	3	12.7
1572	5	88	32902	CAGT	4	10.8
1573	5	88	51287	CAC	3	13.7
1574	5	88	53824	TCTTCA	6	9.3
1575	5	88	55017	GGTA	4	17
1576	5	88	70777	CA	2	27.5
1577	5	88	73505	CCTGAT	6	9.8
1578	1	89	12071	AACGAC	6	18.3
1579	1	89	13205	AGA	3	12
1580	1	89	66744	TTC	3	82.3
1581	1	89	67634	ATG	3	9.7
1582	1	89	67840	TTC	3	13.7
1583	1	89	71276	TATG	4	8.3
1584	1	89	71837	TGTAG	5	8.6
1585	NA	93	1 ~ 231	CCTAAC	6	38.5
1586	NA	93	281 ~ 342	TTTTAA	6	10.2
1587	NA	93	2023 ~ 2060	TTG	3	13
1588	NA	93	23930	TAA	3	13
1589	2	94	28578	GGT	3	13
1590	2	94	29352	AC	2	23
1591	2	94	32719	GAC	3	10.3
1592	2	94	35621	CCT	3	10.7
1593	2	94	37993	CTG	3	9
1594	3	95	8103 ~ 8130	GCG	3	9.3
1595	3	95	33922	GTT	3	13.7
1596	3	95	35019	CAG	3	12
1597	5	97	1815 ~ 1875	AGAAAA	6	9.8
1598	5	97	10701	AGAAAA	6	9.8
1599	5	97	19532	AGAAAA	6	9.8
1600	5	97	28372	AGAAAA	6	9.8
1601	5	97	37197	AGAAAA	6	9.8
1602	5	97	46042	AGAAAA	6	9.8
1603	1	99	9862 ~ 9904	CATCC	5	8.2
1604	NA	100	26713	GAA	3	14
1605	NA	101	2987 ~ 3053	GTAG	4	16.8
1606	NA	101	4215 ~ 4250	GA	2	18.5
1607	NA	101	26687	GGT	3	9.7
1608	NA	101	28565	GTT	3	13.7
1609	NA	101	36503	GTG	3	10
1610	NA	103	16151	CAA	3	10
1611	NA	104	3579 ~ 3719	ACA	3	47
1612	NA	105	187 ~ 245	AT	2	30
1613	NA	106	21028	TAT	3	11.3
1614	NA	107	19452	GAGGGA	6	12.8
1615	NA	109	12773	CCT	3	10.3
1616	NA	109	14968	ACA	3	26
1617	NA	114	11192	CAA	3	10
1618	NA	115	13391	AGAAAA	6	9.8
1619	NA	123	2135 ~ 2171	TC	2	18.5
1620	3	1	198280	AAATAT	6	9.5
1621	3	1	317292	CCT	3	8.3
1622	3	1	317373	TCT	3	20.7
1623	3	1	471127	CAGCAG	6	16.8
1624	3	1	471142	CAA	3	20.7
1625	3	1	654097	AAT	3	30.7
1626	3	1	908904	AGA	3	27
1627	3	1	909014	GAT	3	10.7
1628	3	1	1135829	CAG	3	30
1629	3	1	1240031	TTTTTC	6	9.8
1630	3	1	1240036	TTTTC	5	11.4
1631	1	2	1664999	CAGCAA	6	14.3
1632	1	2	1664978	CAA	3	19.7
1633	1	3	803362	AAAAGA	6	11.7
1634	1	3	803362	AAAG	4	17.8
1635	6	4	920154	AAGGAG	6	12.5
1636	6	4	920180	AGA	3	23
1637	6	4	996239	TGT	3	16.7
1638	6	4	996216	GTT	3	8.3
1639	6	4	1044084	AATAGG	6	14
1640	6	4	1044221	TAGGGA	6	25.7
1641	6	4	1045578	GAAAGG	6	11.5
1642	2	5	999632	TGCCAC	6	8
1643	2	5	999795	TGA	3	9.7
1644	1	6	358720	ATG	3	8.3
1645	1	6	358870	GGA	3	20.3
1646	1	6	556845	CAA	3	11.7
1647	1	6	556912	CAG	3	12
1648	1	7	667816	CTTT	4	11
1649	1	7	789012	GCA	3	9.3
1650	1	7	789097	CAT	3	16.7
1651	1	7	862677	TTTCC	5	32.4
1652	1	7	862951	TCTG	4	10.5
1653	1	7	863065	TCC	3	17.3
1654	1	7	956155	AGC	3	9.7
1655	1	7	956249	GAATGG	6	9.2
1656	2	8	504664	GGTA	4	9.8

No	Chromosome	Contig number	Position within contig	Unit Sequence	Unit length	Repeat number[b]
1657	2	8	504708 ~ 504826	AAC	3	12.3
1658	2	8	603116 ~ 603164	TGT	3	15
1659	2	8	603207 ~ 603348	TGAAGT	6	8.7
1660	2	8	787110 ~ 787197	GAGAAA	6	14.7
1661	7	10	482910 ~ 482967	ACATTT	6	9
1662	7	10	640085 ~ 640179	TGCTGT	6	15.8
1663	7	10	640100 ~ 640182	TGT	3	22.7
1664	5	11	754579 ~ 754617	AGG	3	13
1665	5	11	754616 ~ 754687	GGT	3	11.7
1666	5	13	15688 ~ 15732	AGA	3	15
1667	5	13	15731 ~ 15823	GAT	3	16.7
1668	5	13	147165 ~ 147209	AAC	3	15
1669	5	13	147231 ~ 147338	TCCAC	5	8.4
1670	5	13	158258 ~ 158343	TACCTC	6	16.7
1671	5	14	269174 ~ 269234	CAA	3	20.3
1672	5	14	269249 ~ 269391	CCT	3	22.7
1673	5	15	506271 ~ 506354	CTC	3	28
1674	5	15	506272 ~ 506358	TCCTCA	6	14.3
1675	5	15	551868 ~ 551910	TTTCT	5	8.6
1676	6	16	55903 ~ 56042	AAATAT	6	18.2
1677	6	16	633664 ~ 633697	CCAT	4	8.5
1678	6	16	633706 ~ 633777	CTC	3	10
1679	3	17	336923 ~ 337070	GGAAAA	6	24
1680	3	17	459509 ~ 459552	ACC	3	14.7
1681	3	17	459551 ~ 459627	ACA	3	11.7
1682	3	17	555171 ~ 555205	CAC	3	11.7
1683	3	17	555204 ~ 555409	CAA	3	57.7
1684	4	18	221821 ~ 221905	GCCTGT	6	14.2
1685	4	18	221836 ~ 221900	TGTGCT	6	8.3
1686	4	18	548434 ~ 548500	TAGTCG	6	11.2
1687	4	18	548434 ~ 548484	TAG	3	17
1688	4	19	373105 ~ 373190	TCT	3	26.7
1689	4	19	373207 ~ 373341	TGCT	4	8.3
1690	4	19	443561 ~ 443600	CTAC	4	10
1691	4	19	443645 ~ 443793	TATGC	5	13
1692	4	20	562907 ~ 562968	TGT	3	20.7
1693	4	20	562967 ~ 563058	TGC	3	10.7
1694	7	23	102164 ~ 102250	CTGCTC	6	13
1695	7	23	102167 ~ 102248	CTC	3	23.3
1696	7	23	460352 ~ 460487	GGA	3	34.3
1697	5	24	363149 ~ 363188	ATAC	4	10
1698	5	24	363209 ~ 363308	AC	2	20
1699	3	25	395599 ~ 395632	TAC	3	11.3
1700	3	25	395653 ~ 395746	CATA	4	10
1701	3	25	395690 ~ 395814	AT	2	17
1702	2	30	209409 ~ 209547	CTTTT	5	27.8
1703	2	30	209562 ~ 209761	TGG	3	15.7
1704	4	35	273402 ~ 273640	TATAC	5	9.8
1705	4	35	286407 ~ 286441	CTGA	4	8.8
1706	4	35	286486 ~ 286624	GAA	3	20
1707	5	37	128435 ~ 128467	GTG	3	11
1708	5	37	128466 ~ 128667	TGT	3	57
1709	5	37	289415 ~ 289557	ACCGTC	6	22.7
1710	1	39	57096 ~ 57124	TCT	3	9.7
1711	1	39	57126 ~ 57194	TCC	3	13
1712	1	39	61455 ~ 61541	GGTTGT	6	14.5
1713	1	39	61493 ~ 61576	TTG	3	15.3
1714	4	43	4396 ~ 4484	AGTT	4	22.8
1715	4	43	4494 ~ 4694	AGTC	4	25.8
1716	4	43	281597 ~ 281657	ATG	3	20.3
1717	2	44	121094 ~ 121185	AGTA	4	23
1718	2	44	121199 ~ 121343	GATG	4	10
1719	2	44	299553 ~ 299644	AGTA	4	23
1720	2	44	299667 ~ 299819	ATGG	4	9.8
1721	3	45	77142 ~ 77167	GTT	3	8.7
1722	3	45	77201 ~ 77289	AAG	3	10
1723	4	47	248949 ~ 249009	AGAAGG	6	10.2
1724	4	47	248960 ~ 249034	GAG	3	21.3
1725	NA	50	71944 ~ 72044	AAATAT	6	13.5
1726	4	51	106714 ~ 106751	CAA	3	12.7
1727	4	51	106750 ~ 106814	CAG	3	9.7
1728	4	53	138426 ~ 138451	GGC	3	8.7
1729	4	53	138461 ~ 138597	ACAGCA	6	17
1730	2	54	142503 ~ 142530	GAA	3	9.3
1731	2	54	142533 ~ 142587	GTG	3	8.3
1732	7	55	199091 ~ 199152	CA	2	31
1733	7	55	199152 ~ 199243	AT	2	15.5
1734	1	56	122172 ~ 122282	TTCCCT	6	17.5
1735	1	56	123630 ~ 123787	CCTATT	6	10.2
1736	2	57	148874 ~ 148950	TGCTGT	6	12.8
1737	2	57	148874 ~ 148953	TGC	3	26.7
1738	2	57	193637 ~ 193698	TTTATA	6	9.5
1739	1	58	164562 ~ 164596	AC	2	17.5
1740	1	58	164596 ~ 164660	AG	2	15.5
1741	1	58	171861 ~ 171922	TGCTGT	6	10.3
1742	1	58	171953 ~ 172079	TTG	3	11.7
1743	NA	61	145209 ~ 145315	TTTATA	6	16.5
1744	5	64	129693 ~ 129723	AAG	3	10.3
1745	5	64	129722 ~ 129803	GAG	3	17.7
1746	3	67	106026 ~ 106053	CAG	3	9.3
1747	3	67	106078 ~ 106175	AGC	3	15.3
1748	3	74	65111 ~ 65198	TTTCTA	6	13.5

No	e	number	within	Sequence	Unit length	number[b]
1749	7	76	86550	CCT	3	15
1750	7	76	86689	ACG	3	12
1751	1	82	68030	TTTATA	6	23.5
1752	6	85	67325	GAC	3	14.7
1753	6	85	67395	GAT	3	10
1754	5	88	68469	CAA	3	30.7
1755	5	88	68625	CAG	3	22.7
1756	5	88	70028	CTTCGT	6	14.5
1757	1	89	58385	GTC	3	11
1758	1	89	58483	TGG	3	9.7
1759	2	94	20752	CCA	3	9
1760	2	94	20861	TCC	3	12.7
1761	3	1	52184	A	1	61
1762	3	1	78595	A	1	25
1763	3	1	78881	T	1	43
1764	3	1	119321	T	1	38
1765	3	1	159622	T	1	42
1766	3	1	160232	A	1	48
1767	3	1	174656	T	1	28
1768	3	1	189907	A	1	31
1769	3	1	199366	A	1	34
1770	3	1	244279	A	1	34
1771	3	1	292517	A	1	35
1772	3	1	465691	A	1	48
1773	3	1	469818	T	1	27
1774	3	1	475724	T	1	38
1775	3	1	484475	A	1	45
1776	3	1	488332	T	1	38
1777	3	1	524148	T	1	46
1778	3	1	537133	T	1	52
1779	3	1	542625	T	1	33
1780	3	1	571296	T	1	31
1781	3	1	612156	A	1	27
1782	3	1	732043	T	1	51
1783	3	1	837678	A	1	42
1784	3	1	856620	A	1	39
1785	3	1	993826	A	1	31
1786	3	1	1013011	T	1	42
1787	3	1	1014068	A	1	34
1788	3	1	1039664	T	1	41
1789	3	1	1077630	T	1	39
1790	3	1	1084651	T	1	45
1791	3	1	1091026	A	1	52
1792	3	1	1100993	A	1	52
1793	3	1	1109445	T	1	30
1794	3	1	1143917	T	1	44
1795	3	1	1161561	A	1	48
1796	3	1	1241494	A	1	37
1797	3	1	1250222	T	1	28
1798	3	1	1411872	T	1	52
1799	3	1	1459382	A	1	36
1800	3	1	1495514	T	1	39
1801	3	1	1499514	A	1	37
1802	3	1	1575538	A	1	39
1803	3	1	1651160	T	1	40
1804	3	1	1759032	A	1	25
1805	3	1	1764379	A	1	42
1806	3	1	1809411	T	1	36
1807	3	1	1827455	A	1	47
1808	7	10	63295	A	1	32
1809	7	10	94582	T	1	55
1810	7	10	116440	T	1	36
1811	7	10	184111	A	1	28
1812	7	10	198681	T	1	28
1813	7	10	199361	A	1	42
1814	7	10	201902	T	1	43
1815	7	10	209136	G	1	31
1816	7	10	209418	A	1	34
1817	7	10	252158	A	1	31
1818	7	10	283654	T	1	28
1819	7	10	311622	T	1	49
1820	7	10	356557	A	1	37
1821	7	10	407359	T	1	71
1822	7	10	432807	A	1	31
1823	7	10	481853	T	1	32
1824	7	10	537609	T	1	43
1825	7	10	558337	G	1	33
1826	7	10	563937	T	1	31
1827	7	10	635669	A	1	32
1828	7	10	669366	A	1	52
1829	7	10	823083	T	1	60
1830	7	10	861538	T	1	49
1831	7	10	873972	A	1	41
1832	7	10	888457	T	1	72
1833	7	10	910539	T	1	36
1834	7	10	924202	A	1	39
1835	NA	100	270 ~ 298	A	1	29
1836	NA	101	2732 ~ 2773	A	1	42
1837	NA	101	23838	A	1	42
1838	NA	101	27946	T	1	43
1839	NA	101	29906	A	1	30
1840	NA	102	24249	T	1	40

No	Chromosome	Contig number	Position within contig	Unit Sequence	Unit length	Repeat number[b]
1841	NA	102	24254 ~ 24283	G	1	30
1842	NA	106	10553 ~ 10625	A	1	73
1843	NA	107	20013 ~ 20057	A	1	45
1844	5	11	9103 ~ 9128	G	1	26
1845	5	11	87520 ~ 87561	T	1	42
1846	5	11	128803 ~ 128870	T	1	68
1847	5	11	155849 ~ 155882	T	1	34
1848	5	11	166438 ~ 166493	T	1	56
1849	5	11	172115 ~ 172155	T	1	41
1850	5	11	226338 ~ 226378	T	1	41
1851	5	11	312680 ~ 312706	C	1	27
1852	5	11	319454 ~ 319504	T	1	51
1853	5	11	408568 ~ 408603	A	1	36
1854	5	11	416867 ~ 416898	A	1	32
1855	5	11	468332 ~ 468359	T	1	28
1856	5	11	498519 ~ 498561	T	1	43
1857	5	11	521910 ~ 521962	A	1	53
1858	5	11	584034 ~ 584061	G	1	28
1859	5	11	772731 ~ 772801	T	1	71
1860	5	11	834180 ~ 834229	T	1	50
1861	NA	111	7663 ~ 7714	A	1	52
1862	NA	112	2082 ~ 2124	A	1	43
1863	5	116	4137 ~ 4194	A	1	58
1864	5	116	12976 ~ 13040	A	1	65
1865	6	12	59342 ~ 59374	T	1	33
1866	6	12	189133 ~ 189175	A	1	43
1867	6	12	201633 ~ 201662	A	1	30
1868	6	12	218100 ~ 218129	A	1	30
1869	6	12	246163 ~ 246196	G	1	34
1870	6	12	266321 ~ 266388	A	1	68
1871	6	12	277532 ~ 277558	T	1	27
1872	6	12	332545 ~ 332584	T	1	40
1873	6	12	335514 ~ 335549	T	1	36
1874	6	12	341052 ~ 341085	A	1	34
1875	6	12	376252 ~ 376362	A	1	111
1876	6	12	393697 ~ 393743	T	1	47
1877	6	12	417683 ~ 417711	T	1	29
1878	6	12	465120 ~ 465145	T	1	26
1879	6	12	504297 ~ 504321	T	1	25
1880	6	12	548798 ~ 548829	T	1	32
1881	NA	125	6419 ~ 6469	T	1	51
1882	NA	127	6699 ~ 6738	T	1	40
1883	5	13	24159 ~ 24190	A	1	32
1884	5	13	34101 ~ 34150	T	1	50
1885	5	13	111993 ~ 112018	A	1	26
1886	5	13	157915 ~ 157952	T	1	38
1887	5	13	159800 ~ 159825	A	1	26
1888	5	13	214027 ~ 214069	T	1	43
1889	5	13	325920 ~ 325962	T	1	43
1890	5	13	408699 ~ 408734	A	1	36
1891	5	13	440145 ~ 440179	A	1	35
1892	5	13	454878 ~ 454915	A	1	38
1893	5	13	538786 ~ 538874	A	1	89
1894	5	13	560314 ~ 560350	A	1	37
1895	5	13	619861 ~ 619918	A	1	58
1896	5	13	680696 ~ 680729	A	1	34
1897	5	13	788232 ~ 788268	T	1	37
1898	5	13	805782 ~ 805807	C	1	26
1899	5	13	847916 ~ 847977	A	1	62
1900	5	14	40658 ~ 40682	A	1	25
1901	5	14	165411 ~ 165459	T	1	49
1902	5	14	170561 ~ 170585	T	1	25
1903	5	14	188042 ~ 188081	A	1	40
1904	5	14	211251 ~ 211296	A	1	46
1905	5	14	214975 ~ 215009	A	1	35
1906	5	14	217345 ~ 217393	T	1	49
1907	5	14	339761 ~ 339806	T	1	46
1908	5	14	386861 ~ 386897	T	1	37
1909	5	14	442120 ~ 442147	T	1	28
1910	5	14	551246 ~ 551286	A	1	41
1911	5	14	647172 ~ 647230	T	1	59
1912	5	14	676326 ~ 676361	T	1	36
1913	5	14	677868 ~ 677896	T	1	29
1914	5	14	683341 ~ 683376	T	1	36
1915	5	14	768530 ~ 768564	A	1	35
1916	5	14	781107 ~ 781150	A	1	44
1917	NA	142	5179 ~ 5226	A	1	48
1918	NA	143	5601 ~ 5642	A	1	42
1919	NA	144	8425 ~ 8469	A	1	45
1920	NA	145	2358 ~ 2401	A	1	44
1921	NA	146	1 ~ 46	A	1	46
1922	NA	147	1 ~ 27	A	1	27
1923	5	15	107012 ~ 107037	T	1	26
1924	5	15	216414 ~ 216467	T	1	54
1925	5	15	373429 ~ 373468	T	1	40
1926	5	15	391853 ~ 391882	G	1	30
1927	5	15	479947 ~ 479981	T	1	35
1928	5	15	494487 ~ 494512	C	1	26
1929	5	15	727057 ~ 727083	A	1	27
1930	5	15	802493 ~ 802518	A	1	26
1931	NA	150	1 ~ 45	T	1	45
1932	NA	155	6436 ~ 6477	A	1	42

No	e	number	within	Sequence	Unit length	number[b]
1933	NA	155	15303	A	1	44
1934	6	16	56535	A	1	48
1935	6	16	67290	A	1	35
1936	6	16	95527	A	1	44
1937	6	16	125133	T	1	50
1938	6	16	135088	T	1	52
1939	6	16	314056	T	1	31
1940	6	16	343072	A	1	26
1941	6	16	362860	T	1	31
1942	6	16	367819	A	1	31
1943	6	16	368458	A	1	30
1944	6	16	445155	T	1	53
1945	6	16	459011	T	1	44
1946	6	16	510798	T	1	31
1947	6	16	521764	T	1	26
1948	6	16	541137	T	1	25
1949	6	16	583042	T	1	56
1950	6	16	583606	A	1	27
1951	6	16	587445	T	1	32
1952	6	16	631440	T	1	35
1953	NA	162	4850 ~ 4895	T	1	46
1954	NA	167	1490 ~ 1533	A	1	44
1955	3	17	18567	A	1	55
1956	3	17	128733	A	1	40
1957	3	17	488070	T	1	116
1958	3	17	552630	A	1	44
1959	6	170	32210	A	1	50
1960	4	18	30957	T	1	41
1961	4	18	44448	A	1	33
1962	4	18	154782	G	1	31
1963	4	18	169084	G	1	28
1964	4	18	181617	A	1	36
1965	4	18	209087	A	1	56
1966	4	18	210490	T	1	26
1967	4	18	234755	G	1	26
1968	4	18	279148	A	1	28
1969	4	18	293145	T	1	43
1970	4	18	318925	T	1	45
1971	4	18	319903	T	1	55
1972	4	18	358322	T	1	34
1973	4	18	400556	A	1	28
1974	4	18	404708	T	1	44
1975	4	18	519642	T	1	31
1976	4	18	519832	T	1	39
1977	4	18	597234	T	1	48
1978	4	18	597410	T	1	25
1979	4	18	622034	A	1	29
1980	4	18	649779	T	1	46
1981	4	18	653702	T	1	38
1982	NA	184	2810 ~ 2852	A	1	43
1983	NA	184	3145 ~ 3203	A	1	59
1984	NA	184	11677	A	1	42
1985	4	19	104360	C	1	28
1986	4	19	166208	T	1	35
1987	4	19	170444	A	1	26
1988	4	19	202780	G	1	29
1989	4	19	210654	T	1	30
1990	4	19	276747	A	1	30
1991	4	19	300177	T	1	41
1992	4	19	312683	A	1	25
1993	4	19	349268	A	1	43
1994	4	19	350022	A	1	44
1995	4	19	432273	T	1	49
1996	4	19	441299	T	1	35
1997	4	19	447386	A	1	37
1998	4	19	517690	T	1	26
1999	4	19	538646	T	1	30
2000	4	19	540453	A	1	34
2001	4	19	544477	T	1	47
2002	4	19	566940	T	1	45
2003	4	19	614542	T	1	39
2004	4	19	614853	A	1	43
2005	4	19	633142	T	1	37
2006	4	19	649889	A	1	25
2007	4	19	657877	T	1	56
2008	4	19	667197	T	1	62
2009	1	2	46114	T	1	51
2010	1	2	55492	A	1	34
2011	1	2	77469	A	1	28
2012	1	2	84896	C	1	25
2013	1	2	107015	A	1	31
2014	1	2	108038	A	1	38
2015	1	2	118481	T	1	28
2016	1	2	221016	A	1	39
2017	1	2	232723	C	1	33
2018	1	2	270461	A	1	26
2019	1	2	282668	G	1	28
2020	1	2	288356	A	1	45
2021	1	2	317013	A	1	39
2022	1	2	416857	A	1	42
2023	1	2	465213	A	1	30
2024	1	2	540833	T	1	44

No	Chromosome	Contig number	Position within contig	Unit Sequence	Unit length	Repeat number[b]
2025	1	2	541692 ~ 541763	A	1	72
2026	1	2	672057 ~ 672096	T	1	40
2027	1	2	690990 ~ 691032	A	1	43
2028	1	2	691169 ~ 691195	A	1	27
2029	1	2	758624 ~ 758659	A	1	36
2030	1	2	769389 ~ 769426	A	1	38
2031	1	2	778669 ~ 778709	A	1	41
2032	1	2	795397 ~ 795429	A	1	33
2033	1	2	820896 ~ 820925	G	1	30
2034	1	2	838590 ~ 838617	T	1	28
2035	1	2	845970 ~ 845994	A	1	25
2036	1	2	933469 ~ 933500	T	1	32
2037	1	2	951224 ~ 951264	T	1	41
2038	1	2	953246 ~ 953294	A	1	49
2039	1	2	970617 ~ 970657	A	1	41
2040	1	2	1038370 ~ 1038418	T	1	49
2041	1	2	1051001 ~ 1051050	T	1	50
2042	1	2	1104979 ~ 1105012	C	1	34
2043	1	2	1110106 ~ 1110179	T	1	74
2044	1	2	1120815 ~ 1120839	A	1	25
2045	1	2	1132651 ~ 1132702	A	1	52
2046	1	2	1192231 ~ 1192261	T	1	31
2047	1	2	1218678 ~ 1218709	G	1	32
2048	1	2	1349524 ~ 1349563	T	1	40
2049	1	2	1355980 ~ 1356004	A	1	25
2050	1	2	1429186 ~ 1429215	C	1	30
2051	1	2	1432402 ~ 1432447	A	1	46
2052	1	2	1433394 ~ 1433419	T	1	26
2053	1	2	1441535 ~ 1441566	T	1	32
2054	1	2	1456462 ~ 1456496	A	1	35
2055	1	2	1472800 ~ 1472827	T	1	28
2056	1	2	1505572 ~ 1505610	T	1	39
2057	1	2	1553770 ~ 1553822	A	1	53
2058	1	2	1571474 ~ 1571502	T	1	29
2059	1	2	1592209 ~ 1592235	C	1	27
2060	1	2	1611117 ~ 1611142	A	1	26
2061	1	2	1612752 ~ 1612785	G	1	34
2062	1	2	1626054 ~ 1626134	T	1	81
2063	1	2	1665938 ~ 1665971	T	1	34
2064	1	2	1737246 ~ 1737274	T	1	29
2065	1	2	1743780 ~ 1743807	G	1	28
2066	1	2	1769366 ~ 1769396	A	1	31
2067	4	20	9242 ~ 9270	A	1	29
2068	4	20	38670 ~ 38704	A	1	35
2069	4	20	119162 ~ 119199	A	1	38
2070	4	20	236598 ~ 236639	T	1	42
2071	4	20	248459 ~ 248486	T	1	28
2072	4	20	249104 ~ 249152	A	1	49
2073	4	20	329423 ~ 329458	A	1	36
2074	4	20	354311 ~ 354338	T	1	28
2075	4	20	430644 ~ 430695	A	1	52
2076	4	20	443664 ~ 443706	T	1	43
2077	4	20	529139 ~ 529173	T	1	35
2078	4	20	650451 ~ 650476	T	1	26
2079	NA	200	198 ~ 249	A	1	52
2080	NA	202	1532 ~ 1574	A	1	43
2081	NA	205	1335 ~ 1382	A	1	48
2082	NA	205	10164 ~ 10206	A	1	43
2083	NA	205	18988 ~ 19033	A	1	46
2084	NA	207	1 ~ 49	A	1	49
2085	NA	208	2481 ~ 2527	A	1	47
2086	NA	209	8215 ~ 8263	A	1	49
2087	NA	209	17045 ~ 17089	A	1	45
2088	7	21	12606 ~ 12656	T	1	51
2089	7	21	12883 ~ 12914	A	1	32
2090	7	21	21704 ~ 21733	A	1	30
2091	7	21	23672 ~ 23728	A	1	57
2092	7	21	41649 ~ 41680	T	1	32
2093	7	21	55708 ~ 55761	T	1	54
2094	7	21	84674 ~ 84698	A	1	25
2095	7	21	147670 ~ 147718	A	1	49
2096	7	21	266114 ~ 266157	T	1	44
2097	7	21	280820 ~ 280883	A	1	64
2098	7	21	305951 ~ 305985	T	1	35
2099	7	21	319323 ~ 319353	A	1	31
2100	7	21	328850 ~ 328876	A	1	27
2101	7	21	342706 ~ 342753	T	1	48
2102	7	21	371459 ~ 371485	A	1	27
2103	7	21	389789 ~ 389857	A	1	69
2104	7	21	415841 ~ 415872	T	1	32
2105	7	21	476509 ~ 476549	T	1	41
2106	7	21	590227 ~ 590269	A	1	43
2107	NA	210	6088 ~ 6130	A	1	43
2108	NA	211	5106 ~ 5149	A	1	44
2109	NA	218	1461 ~ 1502	A	1	42
2110	6	22	88562 ~ 88595	A	1	34
2111	6	22	118677 ~ 118702	C	1	26
2112	6	22	138330 ~ 138366	G	1	37
2113	6	22	140588 ~ 140654	T	1	67
2114	6	22	246597 ~ 246635	T	1	39
2115	6	22	247445 ~ 247491	T	1	47
2116	6	22	248574 ~ 248612	T	1	39

No	e	number	within	Sequence	Unit length	number[b]
2117	6	22	287962	T	1	39
2118	6	22	389346	G	1	28
2119	6	22	406452	T	1	35
2120	6	22	407108	T	1	26
2121	6	22	408250	T	1	33
2122	6	22	487041	A	1	45
2123	6	22	510625	T	1	59
2124	NA	224	1 ~ 44	T	1	44
2125	NA	226	805 ~ 864	T	1	60
2126	NA	228	1 ~ 41	T	1	41
2127	7	23	3703 ~ 3738	T	1	36
2128	7	23	27429	T	1	48
2129	7	23	200944	A	1	39
2130	7	23	268242	A	1	25
2131	7	23	343662	T	1	37
2132	7	23	351517	A	1	33
2133	7	23	373023	T	1	31
2134	7	23	408766	A	1	39
2135	7	23	417839	T	1	27
2136	7	23	448088	A	1	41
2137	7	23	453223	T	1	38
2138	7	23	515276	A	1	40
2139	NA	234	4642 ~ 4679	T	1	38
2140	NA	237	2433 ~ 2481	T	1	49
2141	5	24	47405	A	1	45
2142	5	24	97801	T	1	39
2143	5	24	251626	T	1	26
2144	5	24	290202	G	1	30
2145	5	24	328106	T	1	35
2146	5	24	346308	A	1	27
2147	5	24	347496	T	1	30
2148	5	24	363757	A	1	61
2149	5	24	373665	G	1	27
2150	5	24	381585	G	1	25
2151	5	24	388348	T	1	69
2152	5	24	414568	T	1	25
2153	5	24	432107	T	1	29
2154	5	24	432141	G	1	34
2155	5	24	453005	T	1	36
2156	5	24	466984	T	1	48
2157	NA	241	1688 ~ 1715	A	1	28
2158	NA	241	1866 ~ 1895	A	1	30
2159	NA	242	8660 ~ 8717	T	1	58
2160	7	249	80884	T	1	42
2161	7	249	81420	A	1	34
2162	3	25	25637	T	1	58
2163	3	25	36046	A	1	35
2164	3	25	54662	A	1	28
2165	3	25	63003	G	1	26
2166	3	25	115886	T	1	52
2167	3	25	235041	T	1	40
2168	3	25	292576	A	1	26
2169	3	25	323134	A	1	45
2170	3	25	386049	T	1	37
2171	3	25	390035	T	1	28
2172	3	25	419968	C	1	25
2173	3	25	423014	C	1	29
2174	3	25	431544	A	1	37
2175	4	26	56134	T	1	33
2176	4	26	86798	A	1	65
2177	4	26	146684	A	1	43
2178	4	26	170057	T	1	63
2179	4	26	217756	A	1	49
2180	4	26	218446	A	1	34
2181	4	26	258982	C	1	34
2182	4	26	302090	A	1	37
2183	4	26	402187	T	1	52
2184	3	27	75998	A	1	47
2185	3	27	131602	T	1	36
2186	3	27	132255	A	1	28
2187	3	27	134957	A	1	28
2188	3	27	158875	A	1	68
2189	3	27	199404	T	1	45
2190	3	27	238103	A	1	49
2191	3	27	326811	A	1	26
2192	3	27	407498	T	1	59
2193	3	27	412264	A	1	27
2194	4	28	2945 ~ 2980	A	1	36
2195	4	28	27954	T	1	51
2196	4	28	30823	T	1	38
2197	4	28	46278	T	1	42
2198	4	28	111756	A	1	42
2199	4	28	194383	T	1	35
2200	4	28	209656	T	1	26
2201	4	28	220811	G	1	28
2202	4	28	257657	A	1	38
2203	4	28	407094	A	1	34
2204	1	29	65529	T	1	28
2205	1	29	105653	T	1	41
2206	1	29	144928	T	1	28
2207	1	29	243056	T	1	31
2208	1	29	308847	T	1	45

No	Chromosome	Contig number	Position within contig	Unit Sequence	Unit length	Repeat number[b]
2209	1	3	16757 ~ 16782	C	1	26
2210	1	3	62221 ~ 62257	T	1	37
2211	1	3	77501 ~ 77542	A	1	42
2212	1	3	95576 ~ 95615	T	1	40
2213	1	3	148571 ~ 148598	T	1	28
2214	1	3	297474 ~ 297511	T	1	38
2215	1	3	313343 ~ 313383	T	1	41
2216	1	3	346793 ~ 346830	A	1	38
2217	1	3	347214 ~ 347269	A	1	56
2218	1	3	411806 ~ 411845	T	1	40
2219	1	3	526282 ~ 526309	A	1	28
2220	1	3	662072 ~ 662122	T	1	51
2221	1	3	712502 ~ 712545	T	1	44
2222	1	3	752105 ~ 752157	T	1	53
2223	1	3	833106 ~ 833151	A	1	46
2224	1	3	844577 ~ 844614	A	1	38
2225	1	3	869098 ~ 869151	A	1	54
2226	1	3	897491 ~ 897519	T	1	29
2227	1	3	903422 ~ 903465	A	1	44
2228	1	3	1028324 ~ 1028351	C	1	28
2229	1	3	1060121 ~ 1060147	T	1	27
2230	1	3	1082204 ~ 1082235	G	1	32
2231	1	3	1105822 ~ 1105851	T	1	30
2232	1	3	1147847 ~ 1147895	T	1	49
2233	1	3	1149393 ~ 1149431	A	1	39
2234	1	3	1222890 ~ 1222929	T	1	40
2235	1	3	1238036 ~ 1238076	T	1	41
2236	1	3	1259156 ~ 1259186	C	1	31
2237	1	3	1271855 ~ 1271907	A	1	53
2238	2	30	52191 ~ 52224	A	1	34
2239	2	30	55668 ~ 55696	A	1	29
2240	2	30	66189 ~ 66214	A	1	26
2241	2	30	71046 ~ 71077	T	1	32
2242	2	30	100354 ~ 100391	T	1	38
2243	2	30	104180 ~ 104206	A	1	27
2244	2	30	108783 ~ 108823	A	1	41
2245	2	30	114780 ~ 114828	A	1	49
2246	2	30	279244 ~ 279297	A	1	54
2247	2	30	313932 ~ 313962	T	1	31
2248	2	30	350510 ~ 350569	T	1	60
2249	2	30	374666 ~ 374698	A	1	33
2250	5	31	41871 ~ 41924	T	1	54
2251	5	31	51898 ~ 51935	T	1	38
2252	5	31	57885 ~ 57921	T	1	37
2253	5	31	106504 ~ 106545	C	1	42
2254	5	31	232044 ~ 232078	T	1	35
2255	5	31	242376 ~ 242410	T	1	35
2256	5	31	306202 ~ 306246	T	1	45
2257	5	31	320393 ~ 320423	G	1	31
2258	5	31	338241 ~ 338283	A	1	43
2259	5	31	376807 ~ 376834	A	1	28
2260	5	31	378699 ~ 378726	A	1	28
2261	7	32	157872 ~ 157912	A	1	41
2262	7	32	183215 ~ 183258	A	1	44
2263	7	32	186570 ~ 186595	A	1	26
2264	7	32	206959 ~ 207000	A	1	42
2265	7	32	275573 ~ 275603	T	1	31
2266	7	32	372109 ~ 372168	A	1	60
2267	2	33	55619 ~ 55645	A	1	27
2268	2	33	67117 ~ 67147	T	1	31
2269	2	33	113285 ~ 113346	T	1	62
2270	2	33	268651 ~ 268689	A	1	39
2271	2	33	285020 ~ 285052	A	1	33
2272	2	33	294605 ~ 294632	T	1	28
2273	2	33	319178 ~ 319225	T	1	48
2274	2	33	352649 ~ 352676	A	1	28
2275	2	33	373769 ~ 373817	T	1	49
2276	6	34	11755 ~ 11791	A	1	37
2277	6	34	12804 ~ 12829	T	1	26
2278	6	34	165279 ~ 165337	T	1	59
2279	6	34	332055 ~ 332083	A	1	29
2280	6	34	341461 ~ 341492	T	1	32
2281	6	34	361207 ~ 361236	C	1	30
2282	4	35	58995 ~ 59043	A	1	49
2283	4	35	134302 ~ 134341	A	1	40
2284	4	35	193005 ~ 193034	A	1	30
2285	4	35	219968 ~ 220016	A	1	49
2286	4	35	221561 ~ 221592	T	1	32
2287	4	35	224469 ~ 224507	A	1	39
2288	4	35	235986 ~ 236015	T	1	30
2289	4	35	241186 ~ 241228	A	1	43
2290	4	35	245356 ~ 245410	T	1	55
2291	4	35	280271 ~ 280311	A	1	41
2292	4	35	285910 ~ 285950	A	1	41
2293	4	35	360405 ~ 360433	G	1	29
2294	4	36	25384 ~ 25439	A	1	56
2295	4	36	50569 ~ 50610	A	1	42
2296	4	36	118250 ~ 118282	A	1	33
2297	4	36	168435 ~ 168477	T	1	43
2298	4	36	181706 ~ 181731	T	1	26
2299	4	36	218349 ~ 218389	A	1	41
2300	5	37	20455 ~ 20484	T	1	30

No	e	number	within	Sequence	Unit length	number[b]
2301	5	37	127890	T	1	45
2302	1	38	22450	T	1	38
2303	1	38	84707	T	1	29
2304	1	38	123133	T	1	39
2305	1	38	135464	T	1	52
2306	1	38	195899	T	1	29
2307	1	38	218491	A	1	68
2308	1	38	252390	C	1	26
2309	1	39	10803	A	1	42
2310	1	39	21415	T	1	26
2311	1	39	75432	T	1	45
2312	1	39	111022	T	1	77
2313	1	39	118994	T	1	48
2314	1	39	131015	A	1	40
2315	1	39	131353	G	1	28
2316	1	39	136216	A	1	32
2317	1	39	174350	T	1	39
2318	1	39	174828	C	1	26
2319	1	39	206717	A	1	25
2320	1	39	215004	G	1	29
2321	1	39	230834	T	1	55
2322	1	39	232302	T	1	59
2323	1	39	268602	T	1	26
2324	6	4	182166	T	1	36
2325	6	4	195705	T	1	42
2326	6	4	257980	A	1	41
2327	6	4	276230	T	1	40
2328	6	4	291771	A	1	33
2329	6	4	297671	A	1	40
2330	6	4	310672	T	1	54
2331	6	4	331594	C	1	28
2332	6	4	341435	T	1	31
2333	6	4	343911	A	1	41
2334	6	4	456533	A	1	59
2335	6	4	463934	T	1	26
2336	6	4	471960	T	1	68
2337	6	4	477761	A	1	49
2338	6	4	636884	A	1	52
2339	6	4	643822	T	1	49
2340	6	4	672904	A	1	83
2341	6	4	767993	A	1	32
2342	6	4	771599	T	1	28
2343	6	4	789973	T	1	46
2344	6	4	823002	A	1	65
2345	6	4	891754	A	1	30
2346	6	4	899674	A	1	61
2347	6	4	954680	A	1	40
2348	6	4	966089	A	1	25
2349	6	4	967720	G	1	25
2350	6	4	973409	A	1	46
2351	6	4	995755	T	1	28
2352	6	4	995943	A	1	43
2353	6	4	1021411	T	1	39
2354	6	4	1025183	A	1	47
2355	6	4	1064698	T	1	39
2356	6	4	1141195	T	1	38
2357	6	4	1168113	A	1	42
2358	3	40	25258	T	1	29
2359	3	40	82809	A	1	49
2360	3	40	90020	T	1	37
2361	3	40	93741	T	1	25
2362	3	40	291939	T	1	48
2363	3	40	292186	G	1	28
2364	5	41	79933	A	1	39
2365	5	41	88177	T	1	46
2366	5	41	180227	A	1	34
2367	5	41	194468	A	1	34
2368	5	41	200165	A	1	35
2369	5	41	225231	T	1	44
2370	5	41	295671	A	1	32
2371	3	42	42004	T	1	31
2372	3	42	61345	A	1	27
2373	3	42	108539	A	1	28
2374	3	42	185698	A	1	26
2375	3	42	196860	A	1	43
2376	3	42	201943	A	1	40
2377	3	42	209052	T	1	27
2378	3	42	239802	T	1	29
2379	3	42	289783	A	1	39
2380	4	43	6753 ~ 6779	A	1	27
2381	4	43	41923	T	1	44
2382	4	43	160431	T	1	88
2383	4	43	166539	A	1	73
2384	4	43	196257	A	1	40
2385	4	43	198587	A	1	26
2386	4	43	256966	T	1	39
2387	4	43	262384	A	1	29
2388	2	44	43049	T	1	43
2389	2	44	77886	T	1	34
2390	2	44	189776	T	1	47
2391	2	44	194550	A	1	34
2392	2	44	212446	A	1	25

No	Chromosome	Contig number	Position within contig	Unit Sequence	Unit length	Repeat number[b]
2393	2	44	221913 ~ 221941	T	1	29
2394	2	44	226851 ~ 226947	A	1	97
2395	2	44	256291 ~ 256324	T	1	34
2396	2	44	282723 ~ 282752	C	1	30
2397	3	45	19897 ~ 19927	C	1	31
2398	3	45	22047 ~ 22079	T	1	33
2399	3	45	65281 ~ 65311	A	1	31
2400	3	45	85342 ~ 85422	A	1	81
2401	3	45	181503 ~ 181550	A	1	48
2402	3	45	265223 ~ 265252	G	1	30
2403	5	46	24759 ~ 24823	A	1	65
2404	5	46	29382 ~ 29422	A	1	41
2405	5	46	29671 ~ 29715	T	1	45
2406	5	46	67081 ~ 67139	A	1	59
2407	5	46	104610 ~ 104645	A	1	36
2408	5	46	169345 ~ 169369	A	1	25
2409	5	46	177247 ~ 177301	T	1	55
2410	5	46	179201 ~ 179229	A	1	29
2411	5	46	184496 ~ 184521	T	1	26
2412	5	46	212793 ~ 212841	T	1	49
2413	5	46	284320 ~ 284350	C	1	31
2414	4	47	24558 ~ 24599	T	1	42
2415	4	47	116941 ~ 116983	T	1	43
2416	4	47	162900 ~ 162924	A	1	25
2417	4	47	186389 ~ 186447	T	1	59
2418	4	47	202281 ~ 202310	T	1	30
2419	4	47	232081 ~ 232121	A	1	41
2420	5	48	8485 ~ 8546	T	1	62
2421	5	48	67492 ~ 67518	A	1	27
2422	5	48	75282 ~ 75329	T	1	48
2423	5	48	132880 ~ 132912	A	1	33
2424	5	48	140767 ~ 140792	T	1	26
2425	5	48	144458 ~ 144493	T	1	36
2426	5	48	156873 ~ 156899	T	1	27
2427	5	48	248822 ~ 248863	A	1	42
2428	4	49	4043 ~ 4090	T	1	48
2429	4	49	19723 ~ 19759	T	1	37
2430	4	49	189310 ~ 189338	A	1	29
2431	4	49	244097 ~ 244126	T	1	30
2432	4	49	262000 ~ 262024	C	1	25
2433	4	49	269915 ~ 269950	T	1	36
2434	2	5	35068 ~ 35105	A	1	38
2435	2	5	35168 ~ 35196	C	1	29
2436	2	5	84241 ~ 84310	T	1	70
2437	2	5	155352 ~ 155378	G	1	27
2438	2	5	262698 ~ 262731	A	1	34
2439	2	5	376049 ~ 376087	T	1	39
2440	2	5	407528 ~ 407575	T	1	48
2441	2	5	443107 ~ 443131	T	1	25
2442	2	5	453158 ~ 453206	T	1	49
2443	2	5	456961 ~ 457020	A	1	60
2444	2	5	471342 ~ 471378	A	1	37
2445	2	5	620887 ~ 620942	A	1	56
2446	2	5	635179 ~ 635234	T	1	56
2447	2	5	643067 ~ 643092	G	1	26
2448	2	5	654409 ~ 654449	T	1	41
2449	2	5	664569 ~ 664596	A	1	28
2450	2	5	683900 ~ 683924	A	1	25
2451	2	5	686826 ~ 686866	T	1	41
2452	2	5	686973 ~ 687004	T	1	32
2453	2	5	695002 ~ 695033	T	1	32
2454	2	5	721391 ~ 721419	A	1	29
2455	2	5	729936 ~ 729996	A	1	61
2456	2	5	862708 ~ 862777	A	1	70
2457	2	5	887808 ~ 887834	A	1	27
2458	2	5	901204 ~ 901248	A	1	45
2459	2	5	985931 ~ 985971	T	1	41
2460	2	5	1025638 ~ 1025662	G	1	25
2461	2	5	1026913 ~ 1026964	A	1	52
2462	2	5	1026989 ~ 1027016	C	1	28
2463	2	5	1065450 ~ 1065498	A	1	49
2464	2	5	1096687 ~ 1096715	A	1	29
2465	2	5	1121017 ~ 1121069	T	1	53
2466	NA	50	62861 ~ 62921	T	1	61
2467	NA	50	72880 ~ 72926	A	1	47
2468	4	51	140153 ~ 140206	T	1	54
2469	4	51	148891 ~ 148942	A	1	52
2470	4	51	171873 ~ 171899	T	1	27
2471	4	51	227992 ~ 228030	T	1	39
2472	4	51	231441 ~ 231526	T	1	86
2473	4	51	243780 ~ 243813	T	1	34
2474	7	52	89864 ~ 89926	A	1	63
2475	7	52	92591 ~ 92617	A	1	27
2476	7	52	98469 ~ 98518	T	1	50
2477	7	52	142618 ~ 142645	C	1	28
2478	7	52	166219 ~ 166252	T	1	34
2479	7	52	174866 ~ 174906	T	1	41
2480	7	52	175226 ~ 175303	T	1	78
2481	7	52	204123 ~ 204166	A	1	44
2482	7	52	211919 ~ 211949	A	1	31
2483	7	52	253106 ~ 253157	A	1	52
2484	4	53	96789 ~ 96830	A	1	42

No	e	number	within	Sequence	Unit length	number[b]
2485	4	53	97296	A	1	27
2486	4	53	157250	T	1	31
2487	4	53	159285	T	1	49
2488	4	53	189839	A	1	29
2489	2	54	92397	T	1	29
2490	2	54	99997	A	1	31
2491	2	54	156896	C	1	30
2492	2	54	160458	T	1	34
2493	2	54	183384	T	1	49
2494	7	55	98814	T	1	32
2495	7	55	98987	T	1	27
2496	1	56	159818	T	1	43
2497	2	57	138 ~ 173	A	1	36
2498	2	57	13068	T	1	27
2499	2	57	105910	A	1	41
2500	2	57	132026	T	1	28
2501	2	57	143939	T	1	26
2502	2	57	146107	A	1	29
2503	2	57	151933	A	1	33
2504	2	57	178862	T	1	58
2505	2	57	184078	T	1	40
2506	1	58	1544 ~ 1586	T	1	43
2507	1	58	131710	T	1	28
2508	1	58	132851	T	1	58
2509	1	58	174415	C	1	28
2510	1	58	182037	T	1	42
2511	1	59	61153	A	1	39
2512	1	59	96283	A	1	73
2513	1	59	107612	T	1	63
2514	1	6	18019	C	1	25
2515	1	6	24365	T	1	50
2516	1	6	43544	A	1	28
2517	1	6	65283	A	1	27
2518	1	6	131327	T	1	57
2519	1	6	157509	T	1	45
2520	1	6	170980	T	1	39
2521	1	6	227701	T	1	50
2522	1	6	344292	A	1	95
2523	1	6	358939	A	1	29
2524	1	6	394824	T	1	56
2525	1	6	396713	T	1	52
2526	1	6	405197	T	1	29
2527	1	6	409882	A	1	39
2528	1	6	422602	T	1	52
2529	1	6	423739	C	1	26
2530	1	6	430815	T	1	50
2531	1	6	436591	A	1	41
2532	1	6	519634	T	1	39
2533	1	6	523337	A	1	26
2534	1	6	591486	T	1	29
2535	1	6	652857	C	1	25
2536	1	6	854675	T	1	42
2537	1	6	865676	T	1	32
2538	1	6	990043	T	1	44
2539	1	6	991337	T	1	49
2540	1	6	1011607	A	1	47
2541	1	6	1052007	G	1	26
2542	4	60	49338	A	1	26
2543	4	60	53084	A	1	40
2544	4	60	66219	T	1	28
2545	4	60	76468	A	1	25
2546	4	60	79585	A	1	41
2547	4	60	88602	A	1	26
2548	NA	61	46532	A	1	35
2549	NA	61	144384	T	1	26
2550	NA	61	153368	G	1	25
2551	1	62	9696 ~ 9731	A	1	36
2552	1	62	9880 ~ 9910	A	1	31
2553	1	62	56146	T	1	33
2554	1	62	56323	T	1	30
2555	1	62	168856	T	1	47
2556	5	63	15507	A	1	25
2557	5	63	37066	A	1	51
2558	5	63	172748	A	1	38
2559	5	64	25765	A	1	30
2560	5	64	38176	A	1	42
2561	5	64	55240	A	1	29
2562	5	64	86070	T	1	36
2563	5	64	135256	A	1	70
2564	5	64	136544	A	1	36
2565	1	65	38599	T	1	51
2566	1	65	103257	A	1	28
2567	1	65	108354	T	1	50
2568	1	65	155644	A	1	25
2569	7	66	9688 ~ 9729	A	1	42
2570	7	66	17571	T	1	39
2571	7	66	41342	T	1	38
2572	7	66	64403	C	1	28
2573	7	66	71840	A	1	44
2574	7	66	87671	A	1	43
2575	7	66	104474	T	1	103
2576	3	67	12474	T	1	55

No	Chromosome	Contig number	Position within contig	Unit Sequence	Unit length	Repeat number[b]
2577	3	67	13897 ~ 13929	T	1	33
2578	3	67	29196 ~ 29237	T	1	42
2579	3	67	119413 ~ 119484	T	1	72
2580	3	67	137561 ~ 137588	A	1	28
2581	2	68	58108 ~ 58133	T	1	26
2582	3	69	46562 ~ 46615	A	1	54
2583	3	69	157574 ~ 157617	T	1	44
2584	1	7	31177 ~ 31203	A	1	27
2585	1	7	34398 ~ 34446	T	1	49
2586	1	7	75107 ~ 75155	A	1	49
2587	1	7	110352 ~ 110410	T	1	59
2588	1	7	144036 ~ 144074	T	1	39
2589	1	7	158773 ~ 158816	A	1	44
2590	1	7	159172 ~ 159203	T	1	32
2591	1	7	179457 ~ 179481	A	1	25
2592	1	7	200448 ~ 200500	T	1	53
2593	1	7	352174 ~ 352212	A	1	39
2594	1	7	367600 ~ 367646	T	1	47
2595	1	7	377592 ~ 377623	A	1	32
2596	1	7	451686 ~ 451714	T	1	29
2597	1	7	528465 ~ 528516	T	1	52
2598	1	7	537377 ~ 537408	T	1	32
2599	1	7	538123 ~ 538148	A	1	26
2600	1	7	547099 ~ 547154	A	1	56
2601	1	7	557692 ~ 557718	C	1	27
2602	1	7	557956 ~ 557992	T	1	37
2603	1	7	561958 ~ 561983	A	1	26
2604	1	7	603657 ~ 603711	T	1	55
2605	1	7	619471 ~ 619522	T	1	52
2606	1	7	646410 ~ 646441	A	1	32
2607	1	7	662747 ~ 662776	A	1	30
2608	1	7	667793 ~ 667844	T	1	52
2609	1	7	686140 ~ 686171	A	1	32
2610	1	7	702745 ~ 702781	T	1	37
2611	1	7	727520 ~ 727559	A	1	40
2612	1	7	729783 ~ 729835	T	1	53
2613	1	7	730667 ~ 730694	A	1	28
2614	1	7	788737 ~ 788775	A	1	39
2615	1	7	805441 ~ 805467	A	1	27
2616	1	7	814182 ~ 814222	A	1	41
2617	1	7	862960 ~ 862984	A	1	25
2618	1	7	906708 ~ 906745	A	1	38
2619	1	7	932482 ~ 932522	T	1	41
2620	1	70	8664 ~ 8703	T	1	40
2621	1	70	13696 ~ 13722	A	1	27
2622	1	70	32916 ~ 32971	A	1	56
2623	1	70	33164 ~ 33193	A	1	30
2624	1	70	71673 ~ 71731	A	1	59
2625	1	70	96051 ~ 96080	A	1	30
2626	1	70	104274 ~ 104303	A	1	30
2627	1	70	128232 ~ 128277	A	1	46
2628	2	71	59620 ~ 59655	T	1	36
2629	1	72	19052 ~ 19092	A	1	41
2630	1	72	34884 ~ 34934	A	1	51
2631	7	73	47135 ~ 47161	A	1	27
2632	3	74	11811 ~ 11839	A	1	29
2633	3	74	16103 ~ 16127	G	1	25
2634	3	74	64242 ~ 64325	T	1	84
2635	7	75	28362 ~ 28389	G	1	28
2636	7	75	51210 ~ 51243	T	1	34
2637	7	75	104282 ~ 104324	A	1	43
2638	7	75	125786 ~ 125819	T	1	34
2639	7	76	37450 ~ 37516	A	1	67
2640	7	76	115799 ~ 115837	A	1	39
2641	7	76	123892 ~ 123948	A	1	57
2642	2	77	7440 ~ 7465	A	1	26
2643	7	78	31751 ~ 31805	T	1	55
2644	7	78	79179 ~ 79208	A	1	30
2645	7	78	120193 ~ 120229	A	1	37
2646	4	79	4422 ~ 4457	T	1	36
2647	4	79	58601 ~ 58629	T	1	29
2648	2	8	109413 ~ 109444	T	1	32
2649	2	8	197557 ~ 197582	G	1	26
2650	2	8	211167 ~ 211200	T	1	34
2651	2	8	234573 ~ 234597	A	1	25
2652	2	8	253577 ~ 253614	A	1	38
2653	2	8	313520 ~ 313562	A	1	43
2654	2	8	318699 ~ 318738	T	1	40
2655	2	8	344591 ~ 344648	A	1	58
2656	2	8	344891 ~ 344926	T	1	36
2657	2	8	355897 ~ 355929	T	1	33
2658	2	8	364433 ~ 364466	T	1	34
2659	2	8	366330 ~ 366378	T	1	49
2660	2	8	378444 ~ 378471	T	1	28
2661	2	8	438702 ~ 438728	A	1	27
2662	2	8	452245 ~ 452269	A	1	25
2663	2	8	458791 ~ 458831	A	1	41

No	e	number	within	Sequence	Unit length	number[b]
2664	2	8	466545	T	1	39
2665	2	8	475340	T	1	43
2666	2	8	495605	A	1	52
2667	2	8	504095	T	1	27
2668	2	8	511206	T	1	25
2669	2	8	537041	T	1	42
2670	2	8	547660	A	1	42
2671	2	8	570556	G	1	27
2672	2	8	582687	A	1	29
2673	2	8	590703	T	1	29
2674	2	8	665105	T	1	34
2675	2	8	703614	T	1	29
2676	2	8	704086	A	1	45
2677	2	8	705961	A	1	49
2678	2	8	735869	A	1	32
2679	2	8	760183	T	1	28
2680	2	8	765199	T	1	65
2681	2	8	818524	T	1	32
2682	2	8	848680	T	1	29
2683	2	8	856962	C	1	31
2684	2	8	938095	A	1	42
2685	NA	80	70389	A	1	50
2686	NA	80	77343	T	1	44
2687	NA	80	110543	T	1	39
2688	2	81	27523	C	1	25
2689	2	81	64181	A	1	27
2690	1	82	1362 ~ 1423	A	1	62
2691	1	82	33503	A	1	31
2692	1	82	38829	T	1	30
2693	1	82	40910	A	1	30
2694	1	82	67080	T	1	30
2695	1	82	72550	A	1	59
2696	1	82	93169	A	1	54
2697	6	85	36335	T	1	37
2698	6	85	38896	T	1	46
2699	1	86	22855	T	1	68
2700	1	86	36628	A	1	32
2701	1	86	41365	A	1	25
2702	5	88	62621	T	1	43
2703	5	88	64950	A	1	72
2704	1	89	38066	A	1	32
2705	1	89	66282	A	1	36
2706	1	89	66808	T	1	59
2707	1	9	15243	A	1	31
2708	1	9	22239	T	1	43
2709	1	9	40213	A	1	26
2710	1	9	80596	A	1	83
2711	1	9	84896	A	1	30
2712	1	9	94388	C	1	41
2713	1	9	124458	T	1	35
2714	1	9	164258	C	1	33
2715	1	9	170390	A	1	27
2716	1	9	193059	A	1	54
2717	1	9	252779	A	1	28
2718	1	9	273888	G	1	25
2719	1	9	292037	G	1	29
2720	1	9	483099	G	1	29
2721	1	9	532697	A	1	37
2722	1	9	583996	T	1	29
2723	1	9	584024	G	1	25
2724	1	9	603256	A	1	37
2725	1	9	651439	T	1	28
2726	1	9	781662	T	1	64
2727	1	9	810028	T	1	53
2728	1	9	817332	A	1	31
2729	1	9	819468	C	1	25
2730	1	9	838157	T	1	28
2731	1	9	849540	T	1	65
2732	1	9	886636	G	1	28
2733	1	9	912197	G	1	25
2734	1	9	913322	T	1	26
2735	NA	90	32838	T	1	38
2736	5	91	2780 ~ 2809	A	1	30
2737	1	92	8929 ~ 8954	G	1	26
2738	NA	93	14598	A	1	59
2739	NA	93	61639	T	1	29
2740	2	94	41821	A	1	40
2741	3	95	23938	T	1	35
2742	3	95	23984	G	1	28
2743	NA	96	44503	T	1	45
2744	5	97	1460 ~ 1524	A	1	65
2745	5	97	10350	A	1	45
2746	5	97	19181	A	1	50
2747	5	97	28021	A	1	59
2748	5	97	36846	A	1	44
2749	5	97	45691	A	1	64

[a], NA stands for Not Aquired

[b], The repeat number refers to the repeat number of the given unit sequence. Each repeat number is obtained by the calculation; Repeat number = Total length within a SSR locus / Unit length
