# Supplementary material for: Simple sequence repeats in Neurospora crassa: distribution, polymorphism and evolutionary inference
Source: BMC Genomics. 2008 Jan 23;9:31. doi: 10.1186/1471-2164-9-31 (PMC2257937; doi:10.1186/1471-2164-9-31)
Supplement: Additional file 2 — GO analysis for proteins containing amino-acid repeats [file 1471-2164-9-31-S2.pdf]

PolyQ

Cellular Components

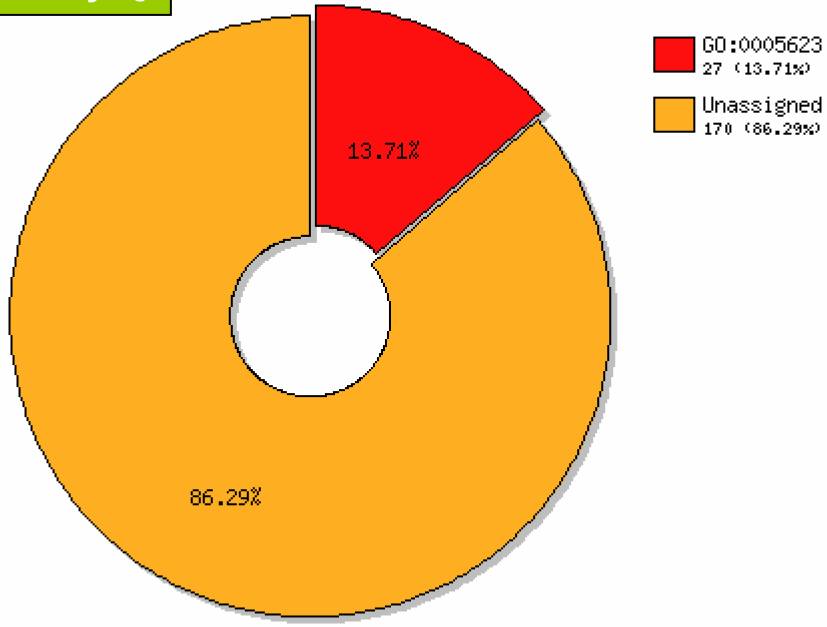

Molecular function

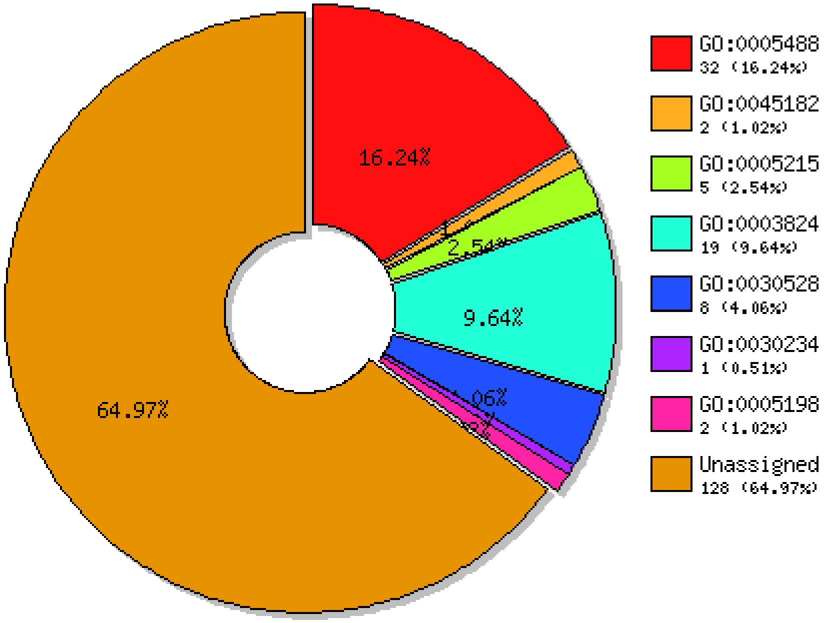

Biological Process

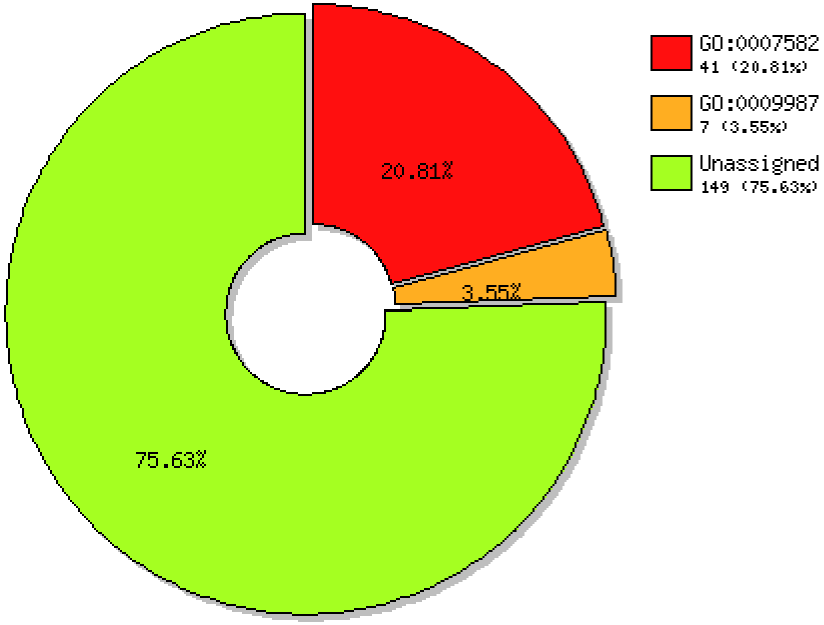

Gene_Ontology (GO:0003673)	0
cellular_component (GO:0005575)	0
cell (GO:0005623)	27
extracellular (GO:0005576)	0
molecular_function (GO:0003674)	0
binding (GO:0005488)	32
translation regulator activity (GO:0045182)	2
transporter activity (GO:0005215)	5
catalytic activity (GO:0003824)	19
transcription regulator activity (GO:0030528)	8
enzyme regulator activity (GO:0030234)	1
molecular_function unknown (GO:0005554)	0
structural molecule activity (GO:0005198)	2
biological_process (GO:0008150)	0
physiological process (GO:0007582)	41
cellular process (GO:0009987)	7
Unassigned (-)	141

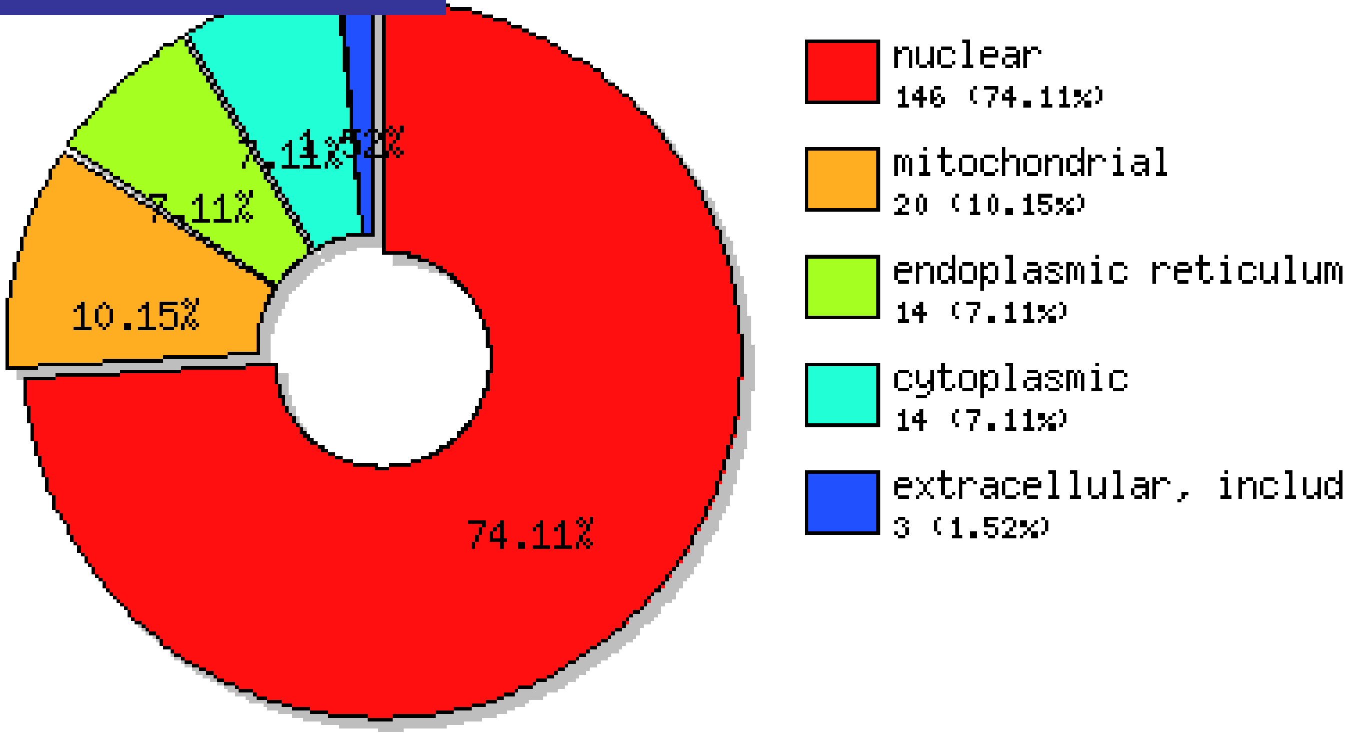

Location	Count	Ratio1
nuclear	146	74.11%
mitochondrial	20	10.15%
endoplasmic reticulum	14	7.11%
cytoplasmic	14	7.11%
extracellular, including cell wa	3	1.52%

PolyQ **Transcription Factors**

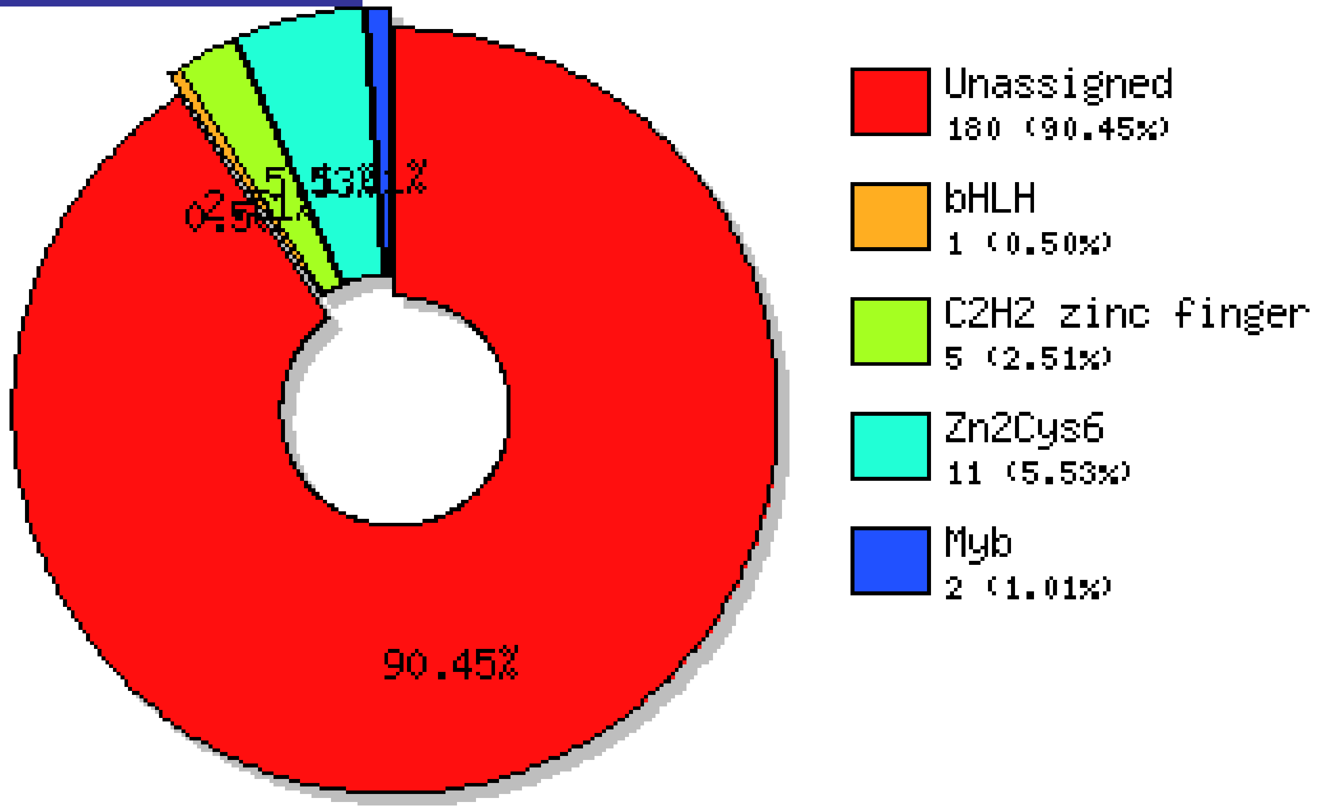

TF Family	Count	Ratio
Unassigned	180	-
bHLH	1	5.26%
C2H2 zinc finger	5	26.32%
Zn2Cys6	11	57.89%
Myb	2	10.53%

PolyD

Cellular Components

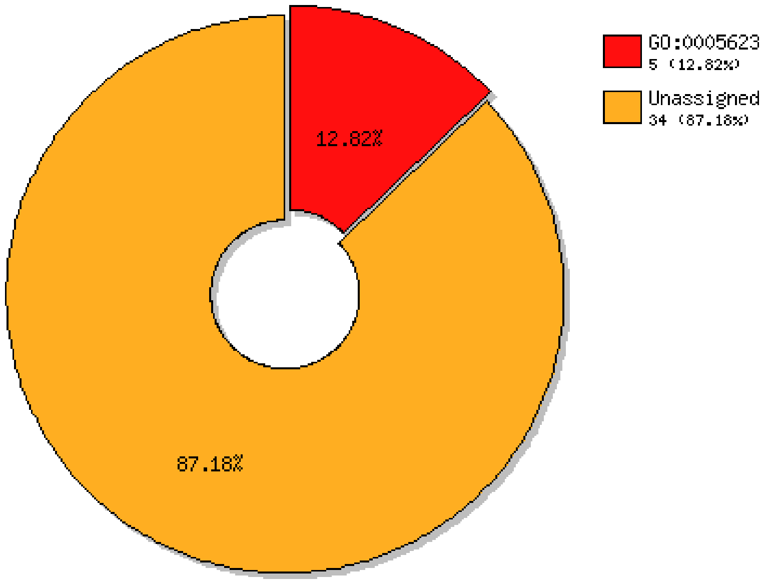

Molecular function

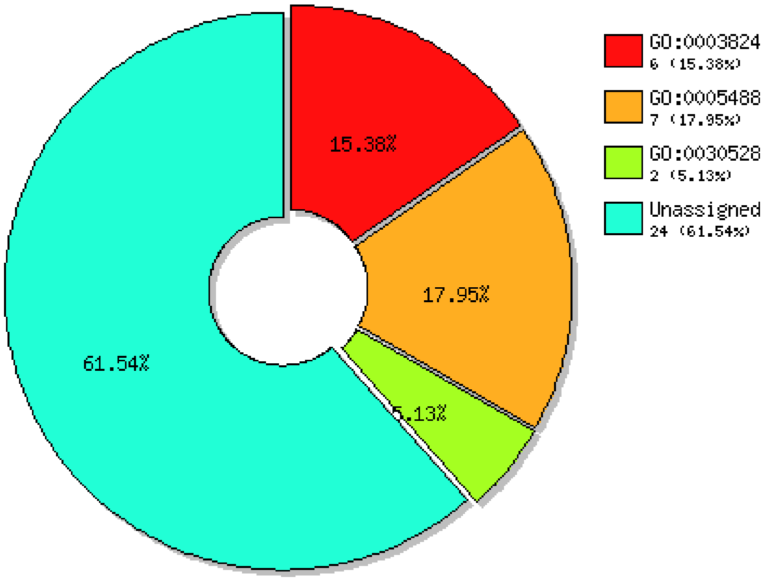

Biological Process

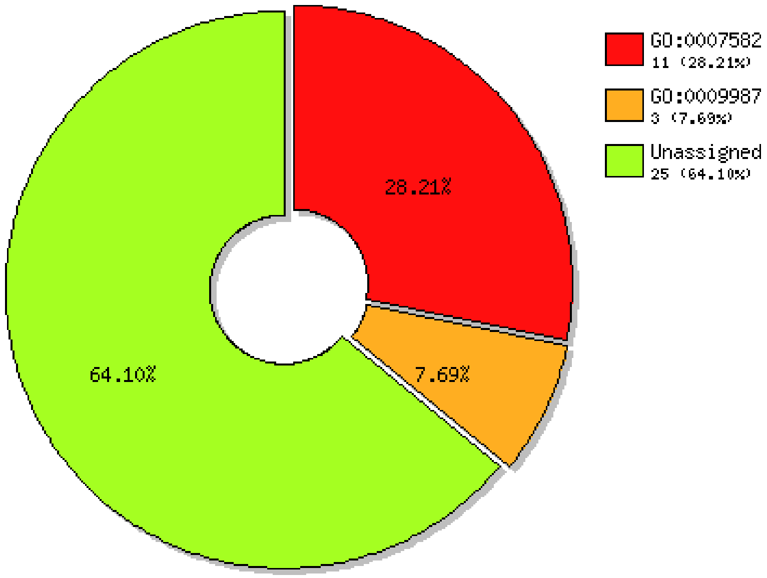

Gene_Ontology (GO:0003673)	0
cellular_component (GO:0005575)	0
cell (GO:0005623)	5
biological_process (GO:0008150)	0
physiological process (GO:0007582)	11
cellular process (GO:0009987)	3
molecular_function (GO:0003674)	0
catalytic activity (GO:0003824)	6
binding (GO:0005488)	7
transporter activity (GO:0005215)	0
transcription regulator activity (GO:0030528)	2
Unassigned (-)	26

PolyD Subcellular Localization

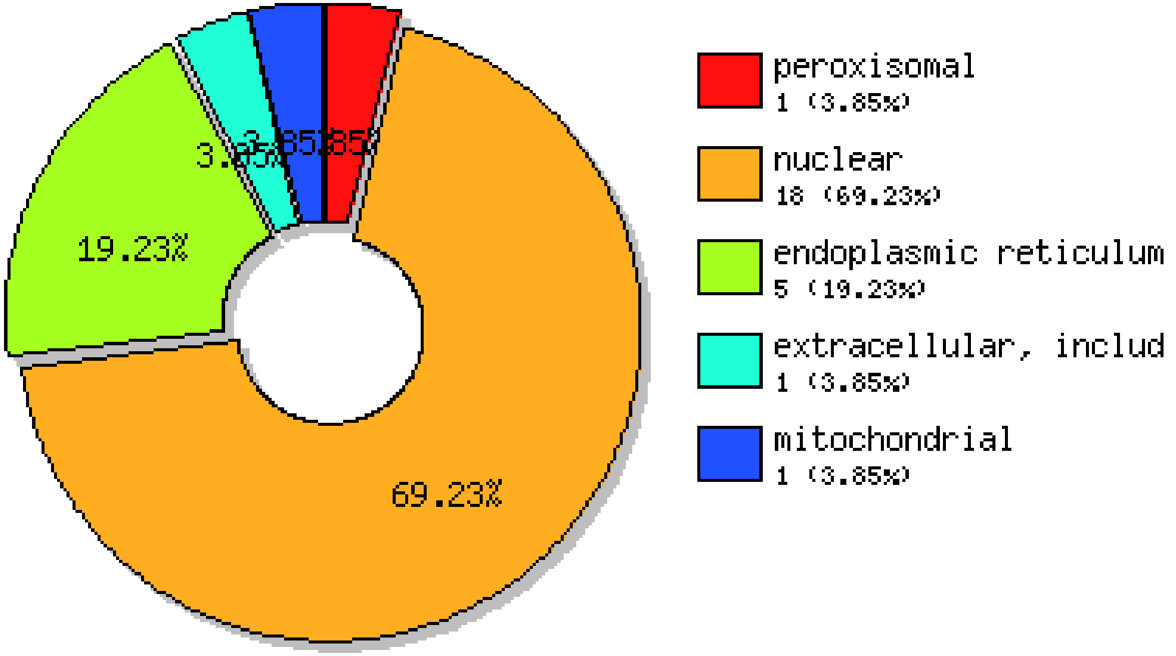

Location	Count	Ratio1
peroxisomal	1	3.85%
nuclear	18	69.23%
endoplasmic reticulum	5	19.23%
extracellular, including cell wa	1	3.85%
mitochondrial	1	3.85%

PolyD **Transcription Factors**

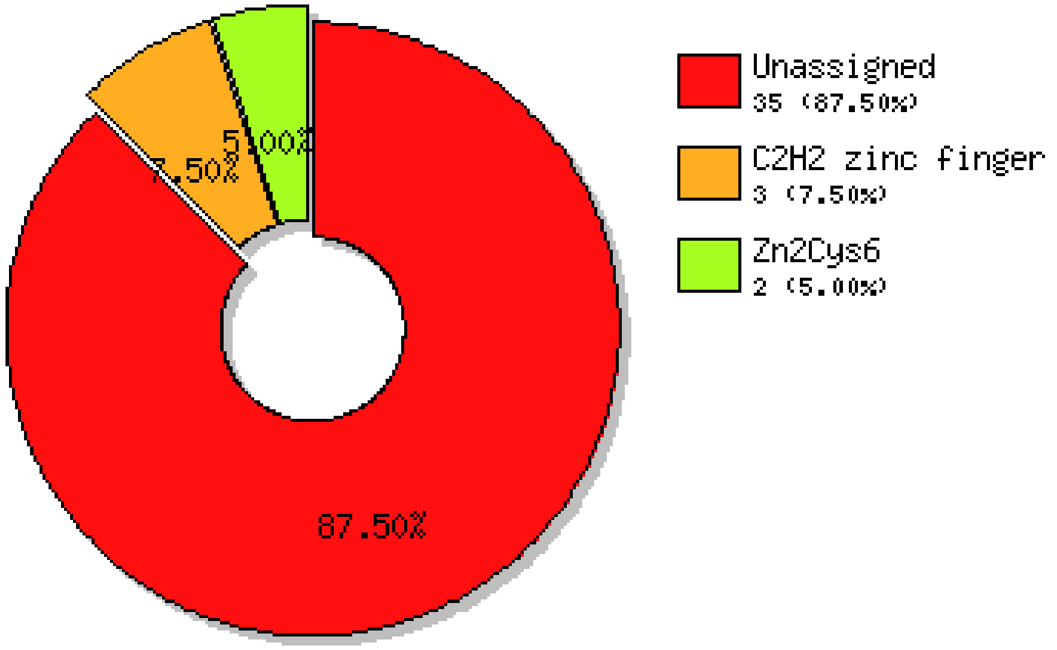

TF Family	Count	Ratio
Unassigned	35	-
C2H2 zinc finger	3	60.00%
Zn2Cys6	2	40.00%

PolyS

Cellular Components

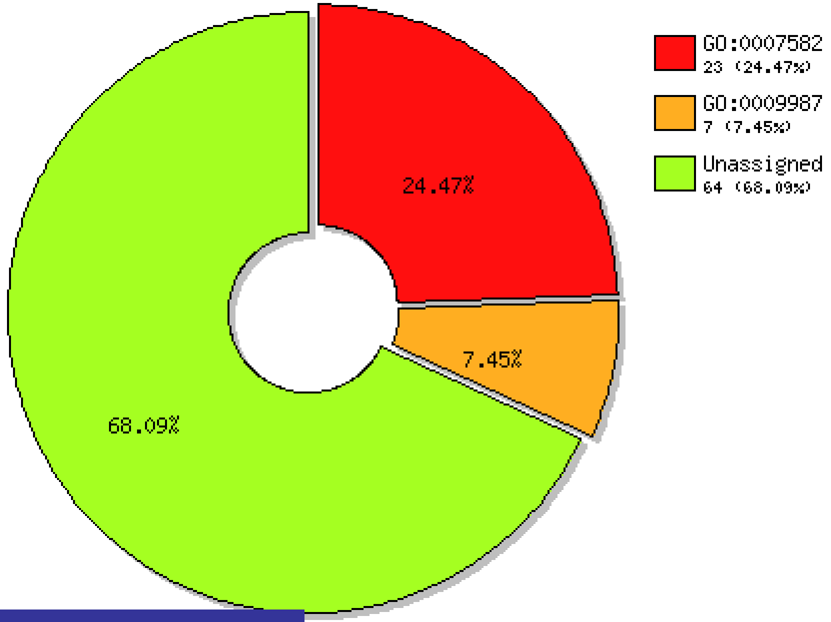

Molecular function

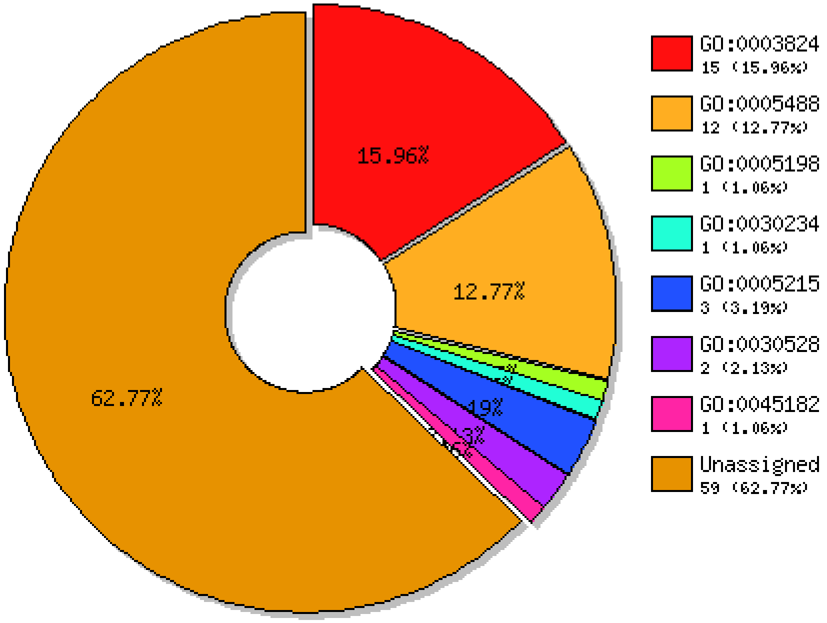

Biological Process

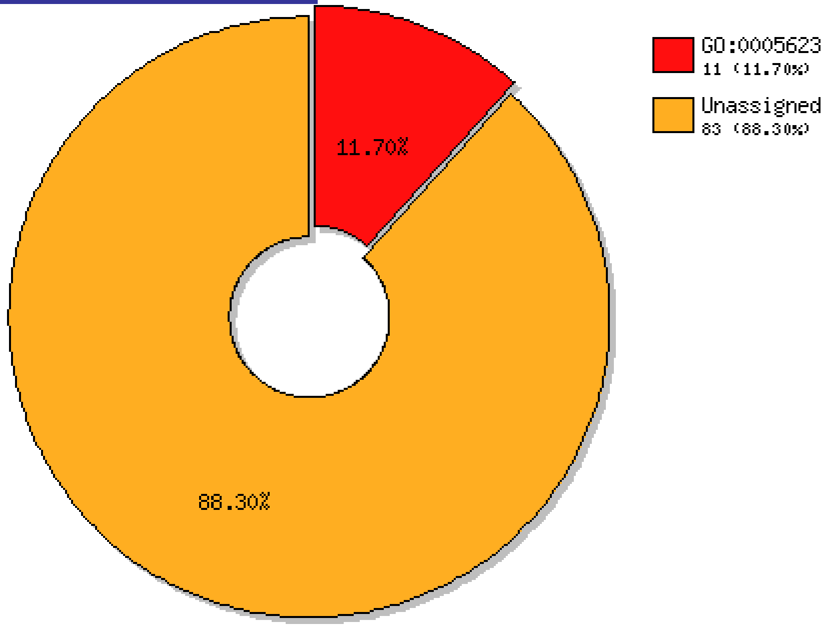

Gene_Ontology (GO:0003673)	0
molecular_function (GO:0003674)	0
catalytic activity (GO:0003824)	15
binding (GO:0005488)	12
structural molecule activity (GO:0005198)	1
enzyme regulator activity (GO:0030234)	1
transporter activity (GO:0005215)	3
transcription regulator activity (GO:0030528)	2
translation regulator activity (GO:0045182)	1
biological_process (GO:0008150)	0
physiological process (GO:0007582)	23
cellular process (GO:0009987)	7
cellular_component (GO:0005575)	0
cell (GO:0005623)	11
extracellular (GO:0005576)	0
Unassigned (-)	63

PolyS **Subcellular Localization**

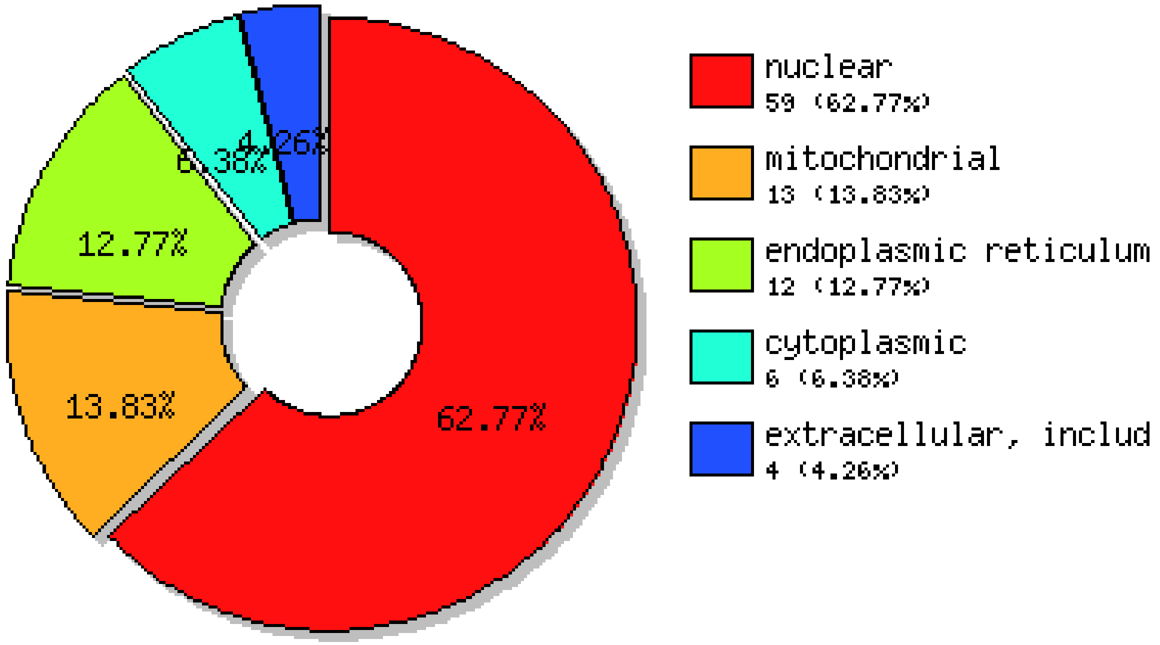

Location	Count	Ratio1
nuclear	59	62.77%
mitochondrial	13	13.83%
endoplasmic reticulum	12	12.77%
cytoplasmic	6	6.38%
extracellular, including cell wa	4	4.26%

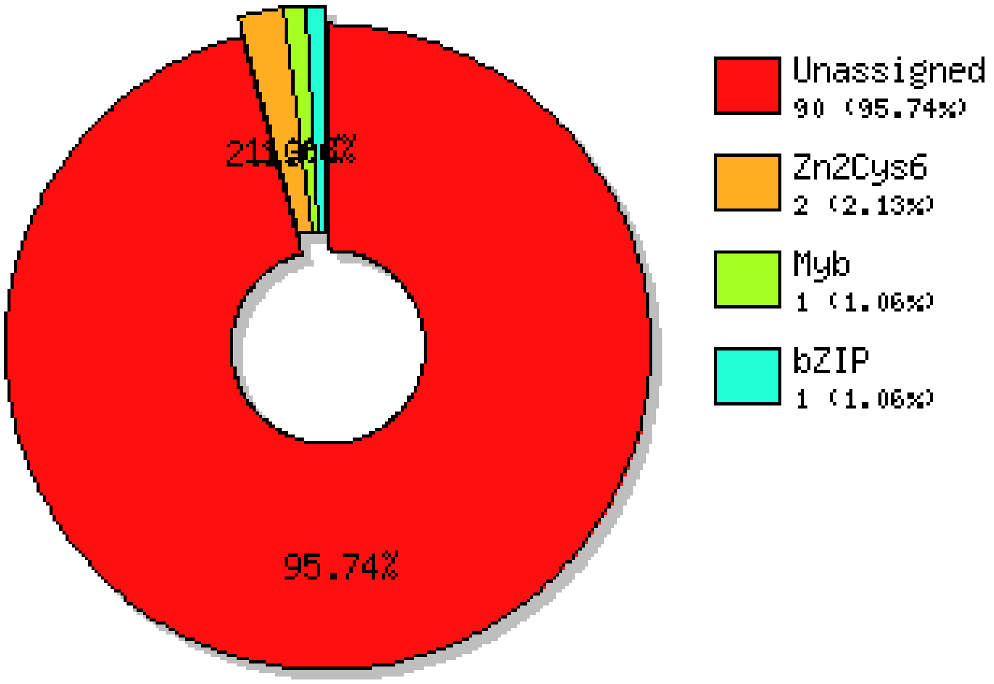

TF Family	Count	Ratio
Unassigned	90	-
Zn2Cys6	2	50.00%
Myb	1	25.00%
bZIP	1	25.00%

PolyA

Cellular Components

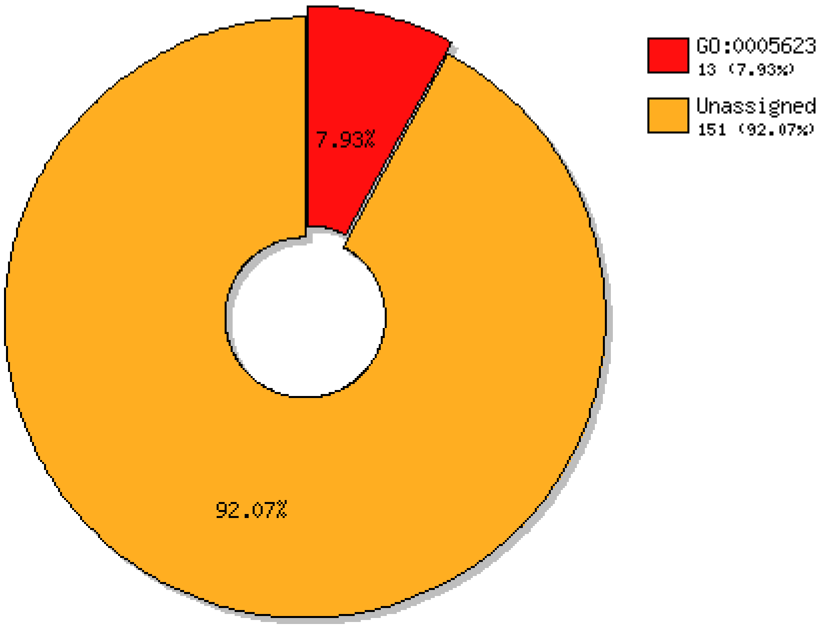

Molecular function

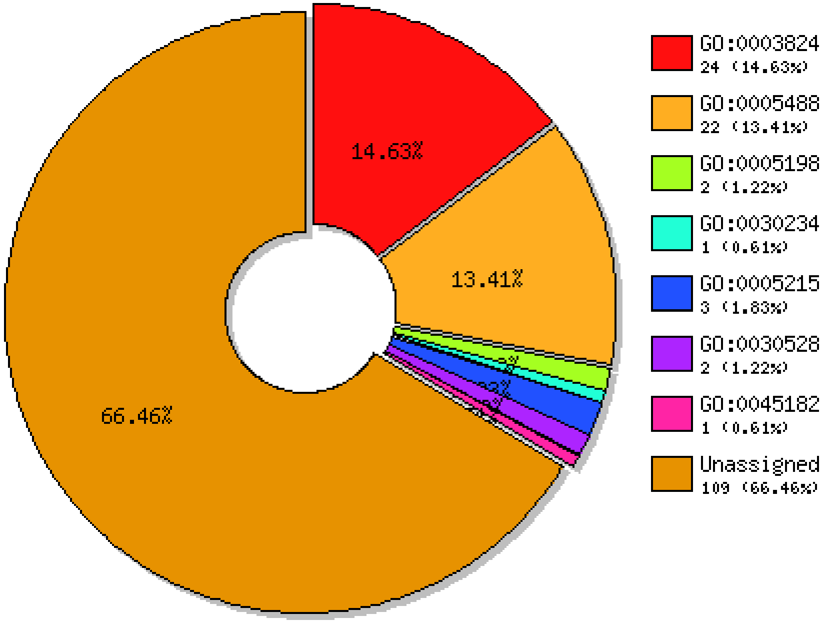

Biological Process

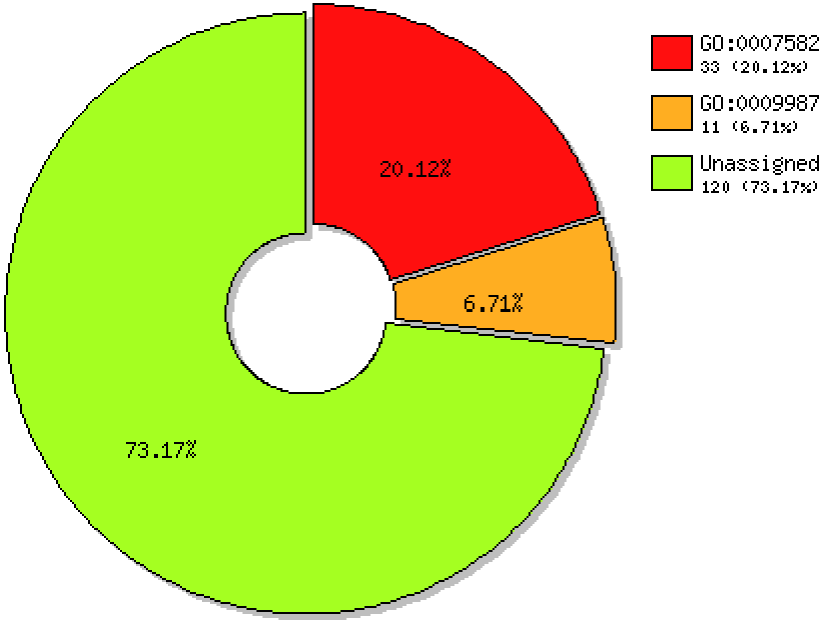

Gene_Ontology (GO:0003673)	0
biological_process (GO:0008150)	0
physiological process (GO:0007582)	33
cellular process (GO:0009987)	11
molecular_function (GO:0003674)	0
catalytic activity (GO:0003824)	24
molecular_function unknown (GO:0005554)	0
binding (GO:0005488)	22
structural molecule activity (GO:0005198)	2
enzyme regulator activity (GO:0030234)	1
transporter activity (GO:0005215)	3
transcription regulator activity (GO:0030528)	2
motor activity (GO:0003774)	0
translation regulator activity (GO:0045182)	1
cellular_component (GO:0005575)	0
cell (GO:0005623)	13
extracellular (GO:0005576)	0
Unassigned (-)	113

PolyA **Subcellular Localization**

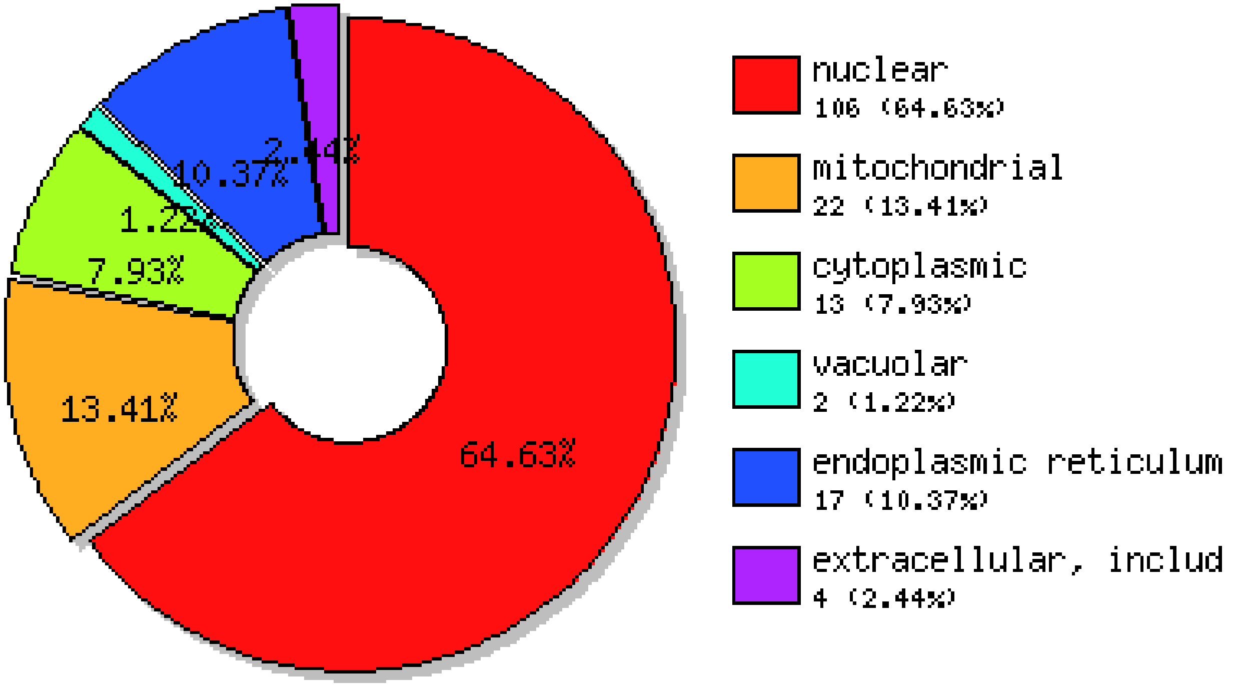

Location	Count	Ratio1
nuclear	106%	64.63%
mitochondrial	22%	13.41%
cytoplasmic	13%	7.93%
vacuolar	2%	1.22%
endoplasmic reticulum	17%	10.37%
extracellular, including cell wa	4%	2.44%

PolyA **Transcription Factors**

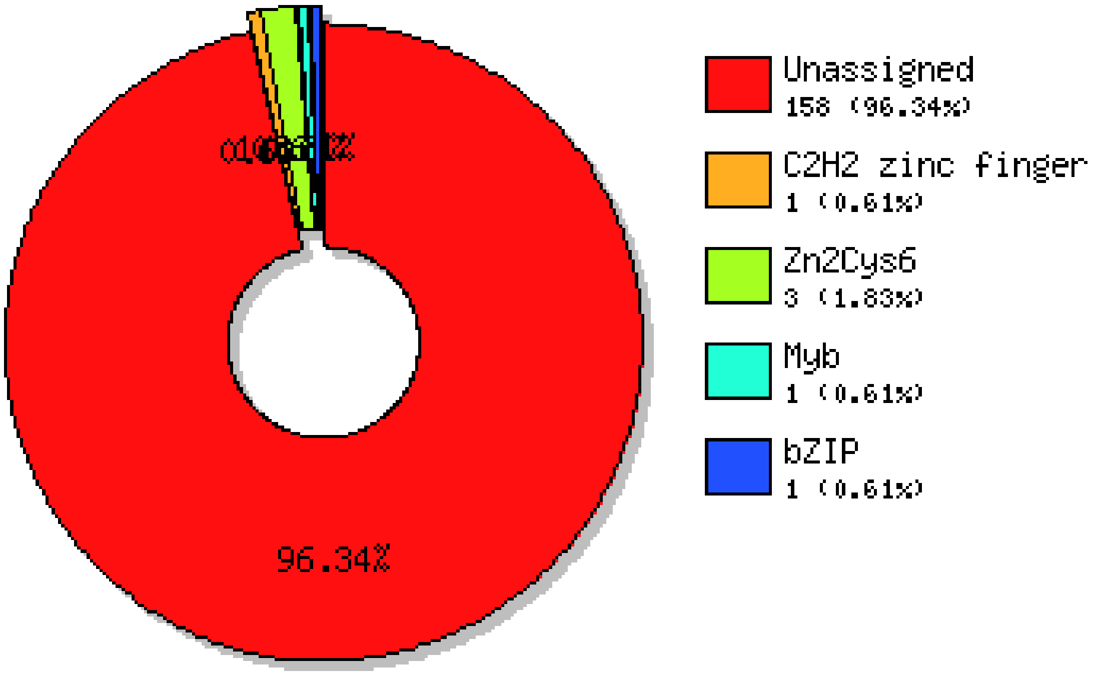

TF Family	Count	Ratio
Unassigned	158	-
C2H2 zinc finger	1	16.67%
Zn2Cys6	3	50.00%
Myb	1	16.67%
bZIP	1	16.67%

PolyG

Cellular Components

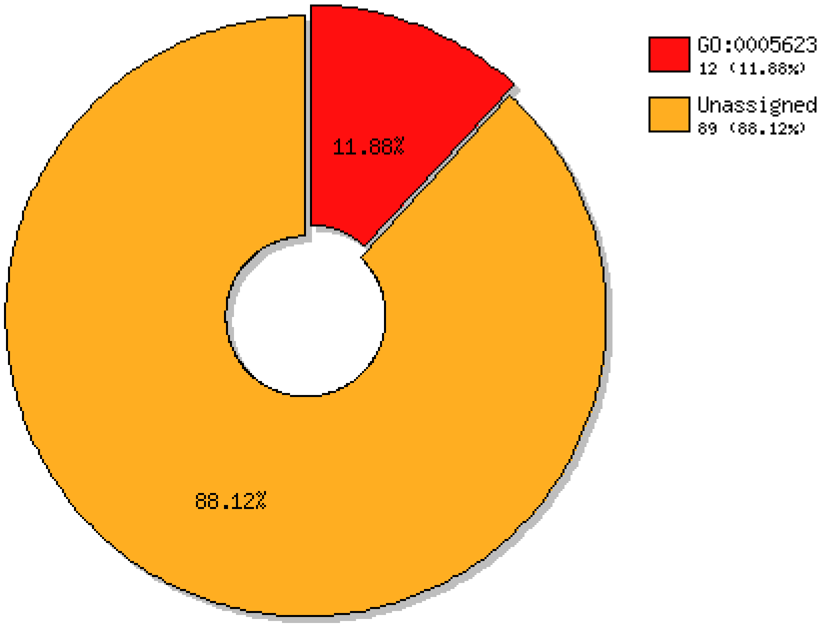

Molecular function

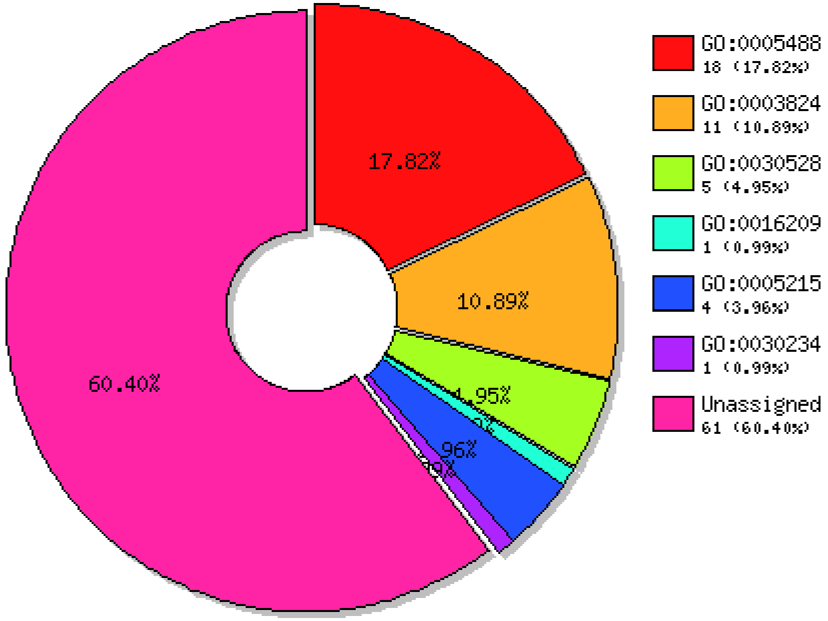

Biological Process

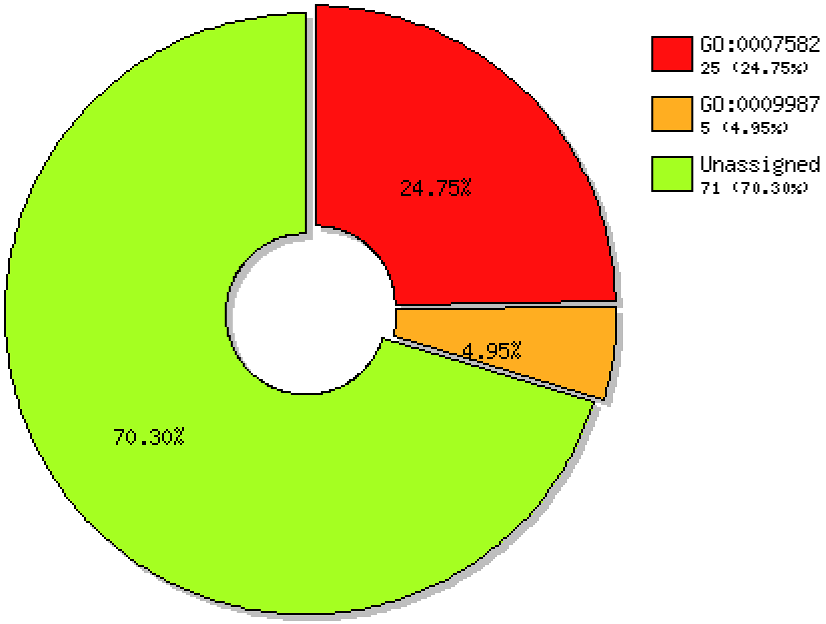

Gene Ontology (GO:0003673)	0
cellular component (GO:0005575)	0
cell (GO:0005623)	12
extracellular (GO:0005576)	0
molecular function (GO:0003674)	0
binding (GO:0005488)	18
catalytic activity (GO:0003824)	11
transcription regulator activity (GO:0030528)	5
antioxidant activity (GO:0016209)	1
chaperone activity (GO:0003754)	0
transporter activity (GO:0005215)	4
enzyme regulator activity (GO:0030234)	1
biological process (GO:0008150)	0
physiological process (GO:0007582)	25
cellular process (GO:0009987)	5
Unassigned (-)	70

PolyG **Subcellular Localization**

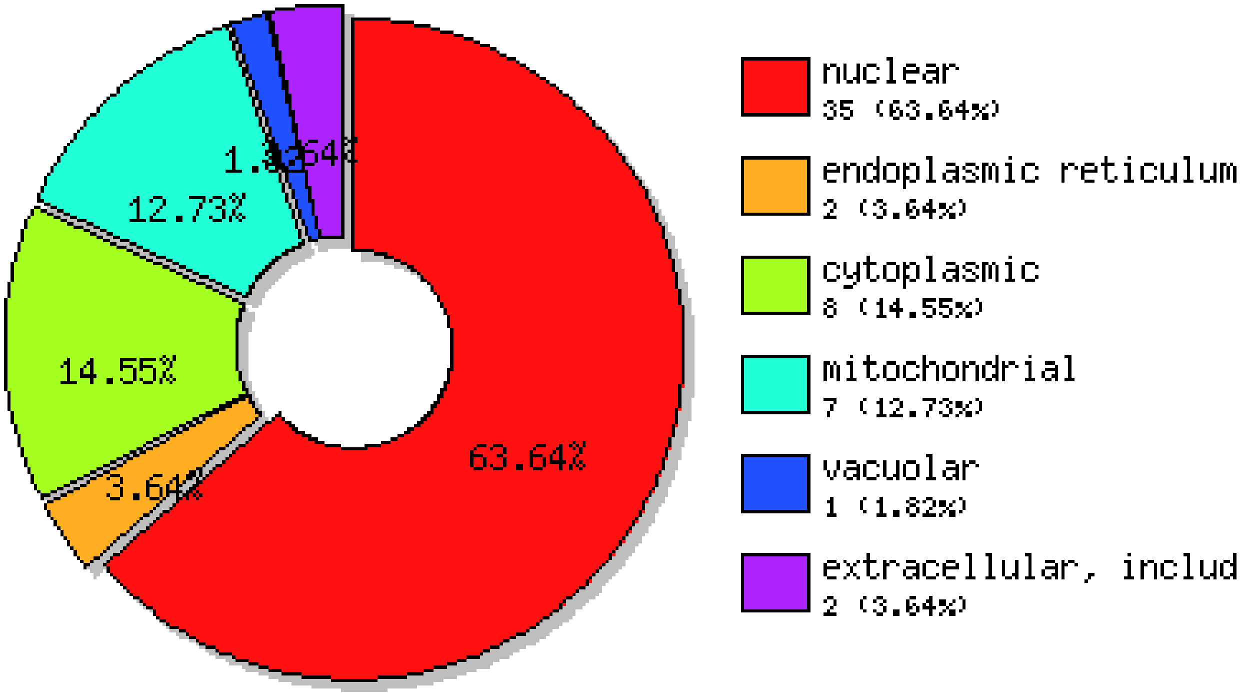

Location	Count	Ratio1
nuclear	35	63.64%
endoplasmic reticulum	2	3.64%
cytoplasmic	8	14.55%
mitochondrial	7	12.73%
vacuolar	1	1.82%
extracellular, including cell wa	2	3.64%

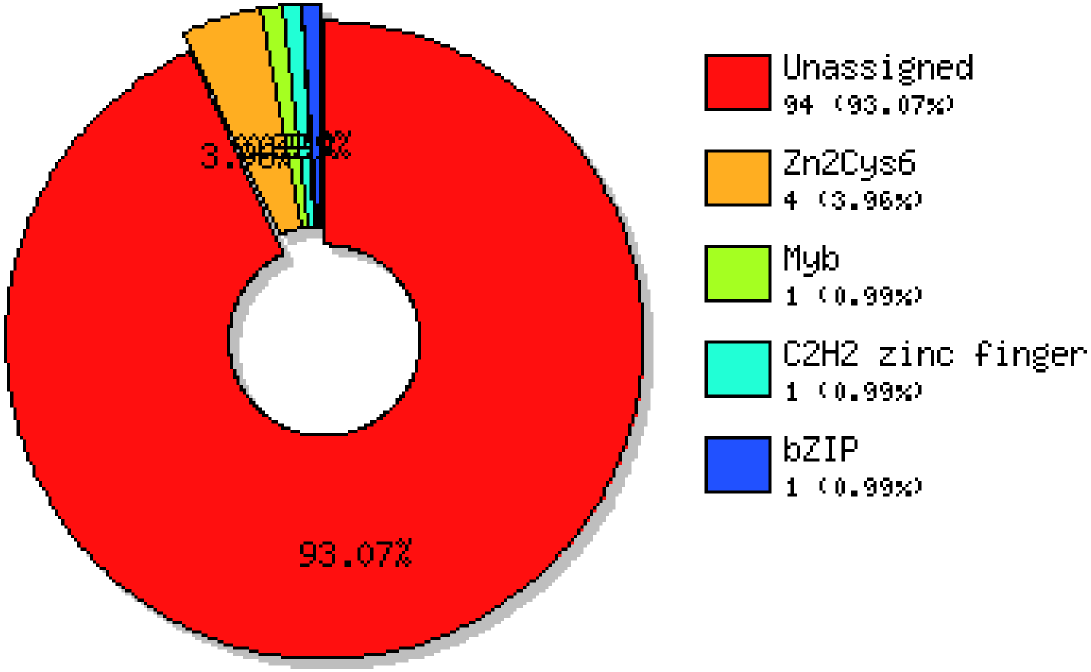

TF Family	Count	Ratio
Unassigned	94	-
Zn2Cys6	4	57.14%
Myb	1	14.29%
C2H2 zinc finger	1	14.29%
bZIP	1	14.29%

PolyP

Cellular Components

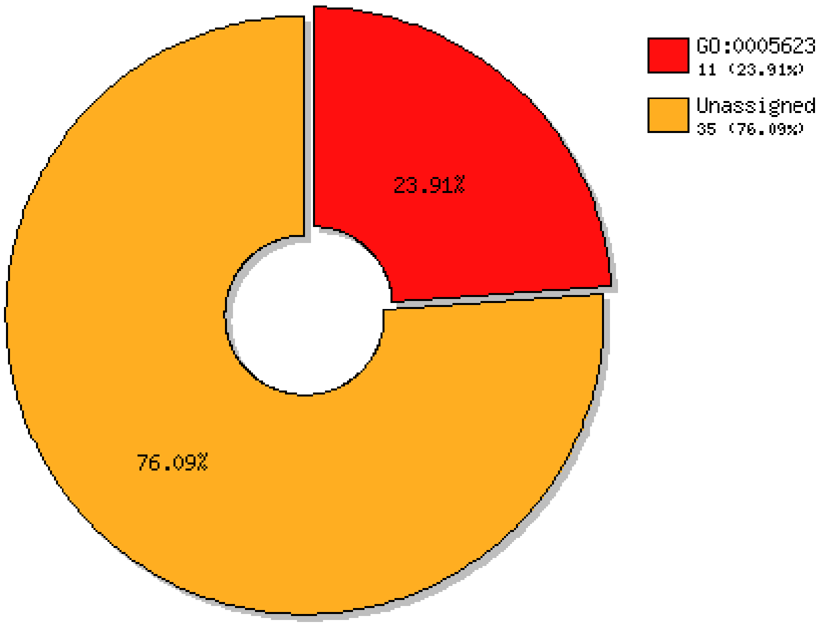

Molecular function

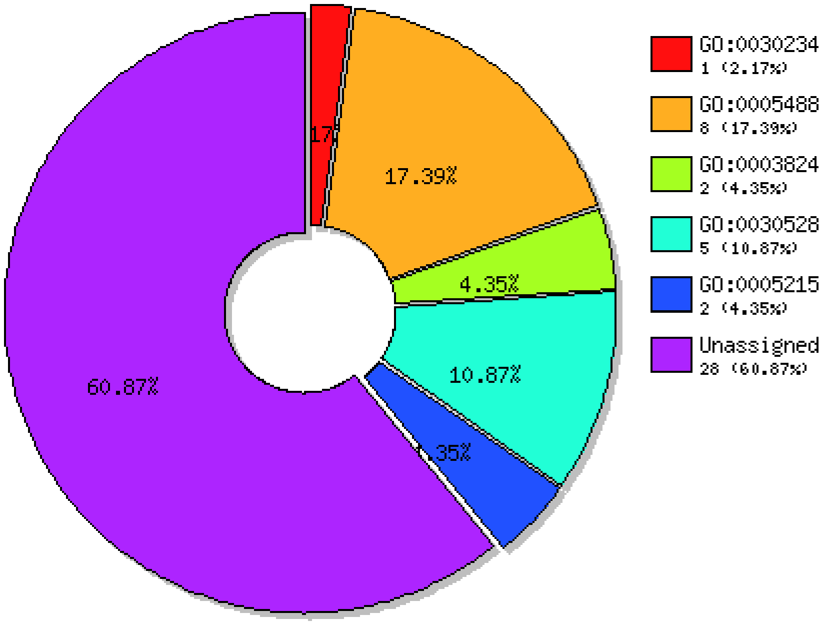

Biological Process

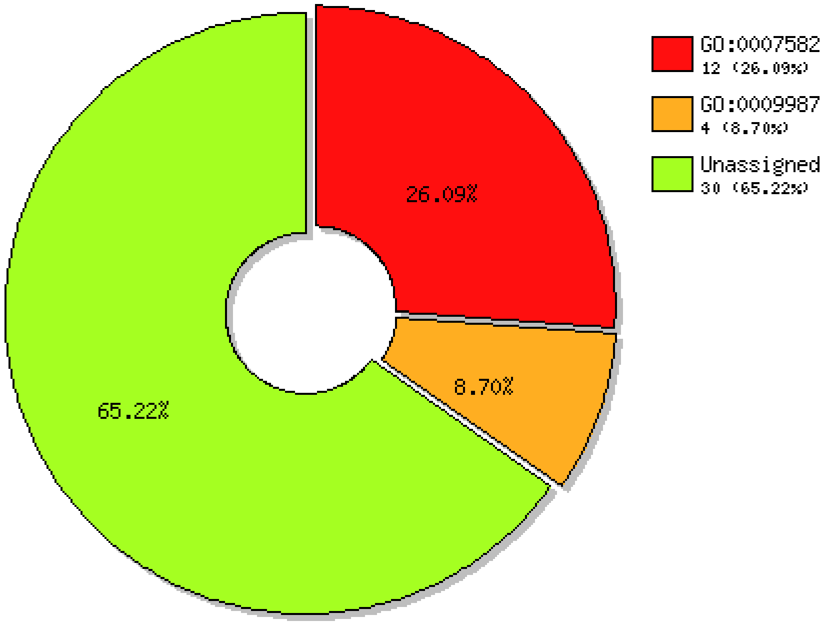

Gene Ontology (GO:0003673)	0
biological process (GO:0008150)	0
physiological process (GO:0007582)	12
cellular process (GO:0009987)	4
molecular function (GO:0003674)	0
chaperone activity (GO:0003754)	0
enzyme regulator activity (GO:0030234)	1
binding (GO:0005488)	8
catalytic activity (GO:0003824)	2
transcription regulator activity (GO:0030528)	5
transporter activity (GO:0005215)	2
cellular component (GO:0005575)	0
cell (GO:0005623)	11
Unassigned (-)	27

PolyP **Subcellular Localization**

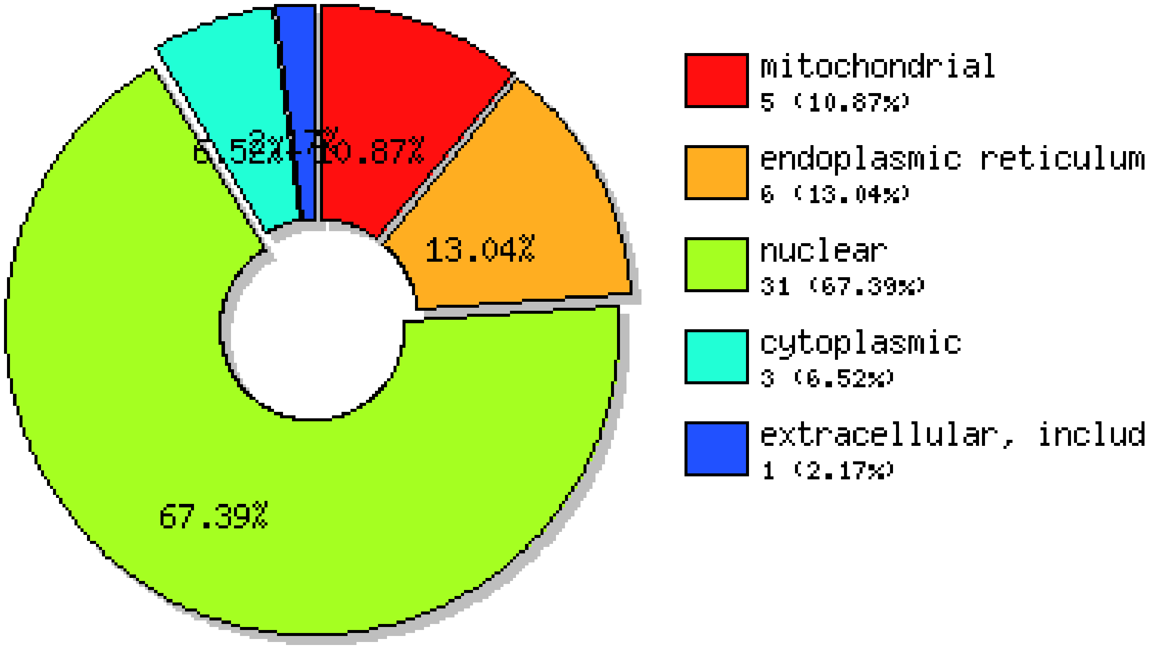

Location	Count	Ratio1
mitochondrial	5%	10.87%
endoplasmic reticulum	6%	13.04%
nuclear	31%	67.39%
cytoplasmic	3%	6.52%
extracellular, including cell wa	1%	2.17%

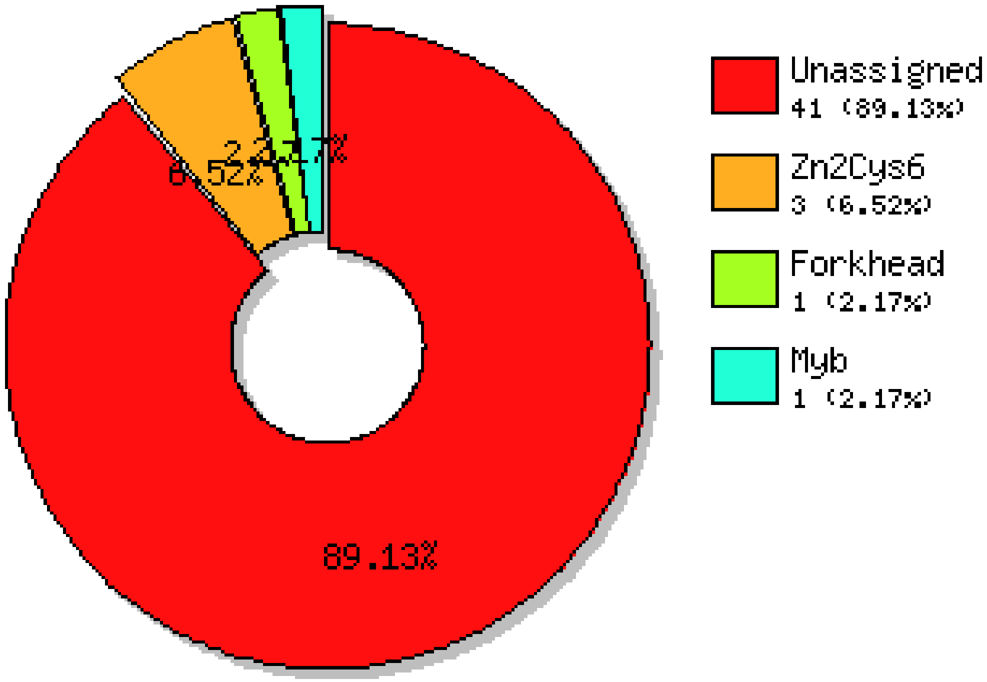

TF Family	Count	Ratio
Unassigned	41	-
Zn2Cys6	3	60.00%
Forkhead	1	20.00%
Myb	1	20.00%

PolyE

Cellular Components

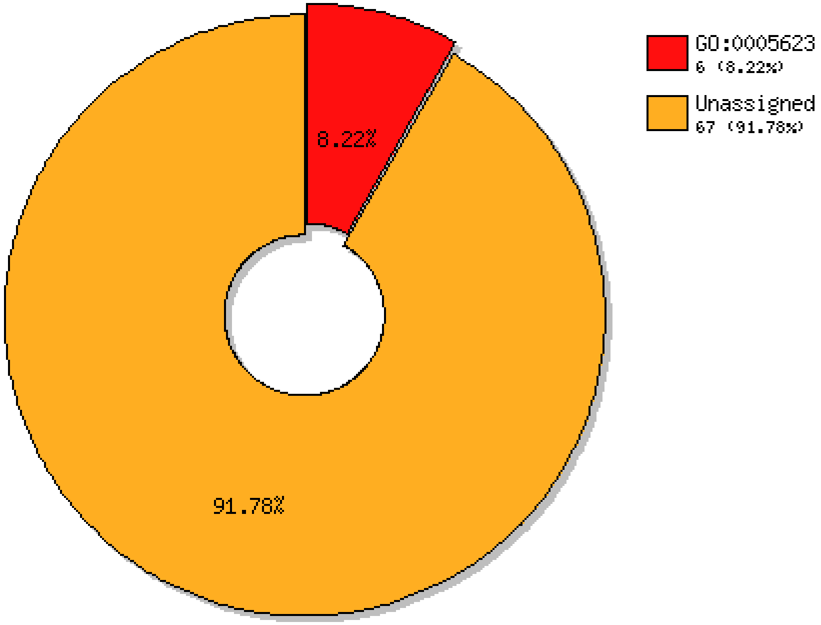

Molecular function

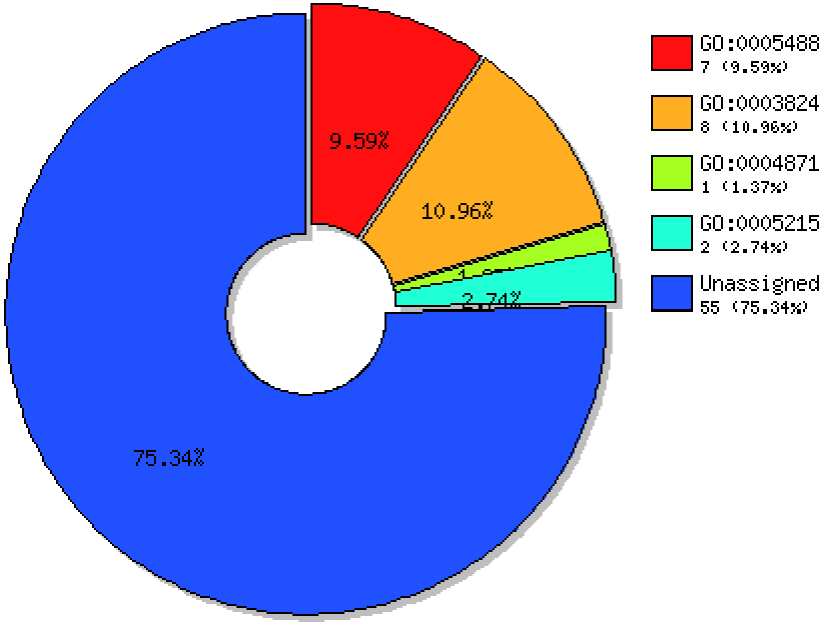

Biological Process

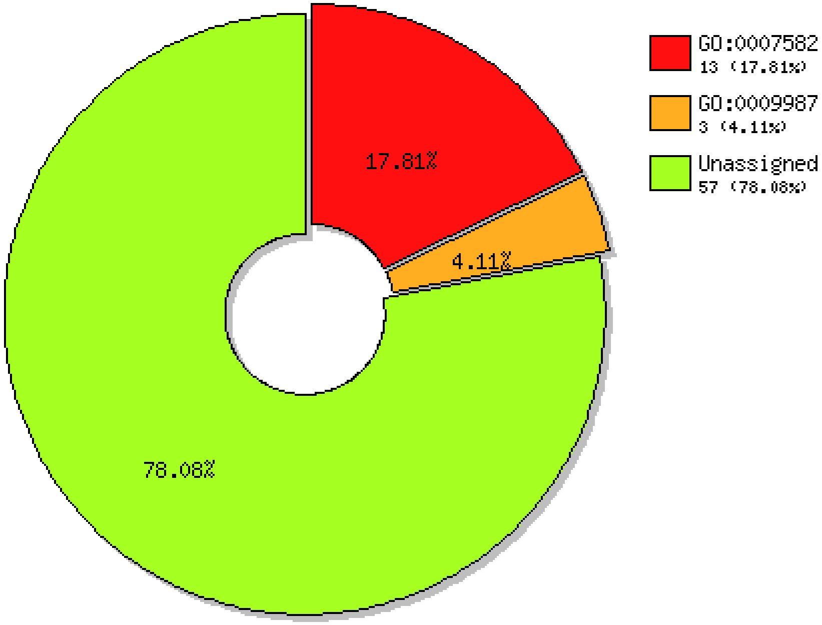

Gene Ontology (GO:0003673)	0
molecular_function (GO:0003674)	0
binding (GO:0005488)	7
catalytic activity (GO:0003824)	8
signal transducer activity (GO:0004871)	1
transporter activity (GO:0005215)	2
biological_process (GO:0008150)	0
physiological process (GO:0007582)	13
cellular process (GO:0009987)	3
cellular_component (GO:0005575)	0
cell (GO:0005623)	6
Unassigned (-)	56

PolyE **Subcellular Localization**

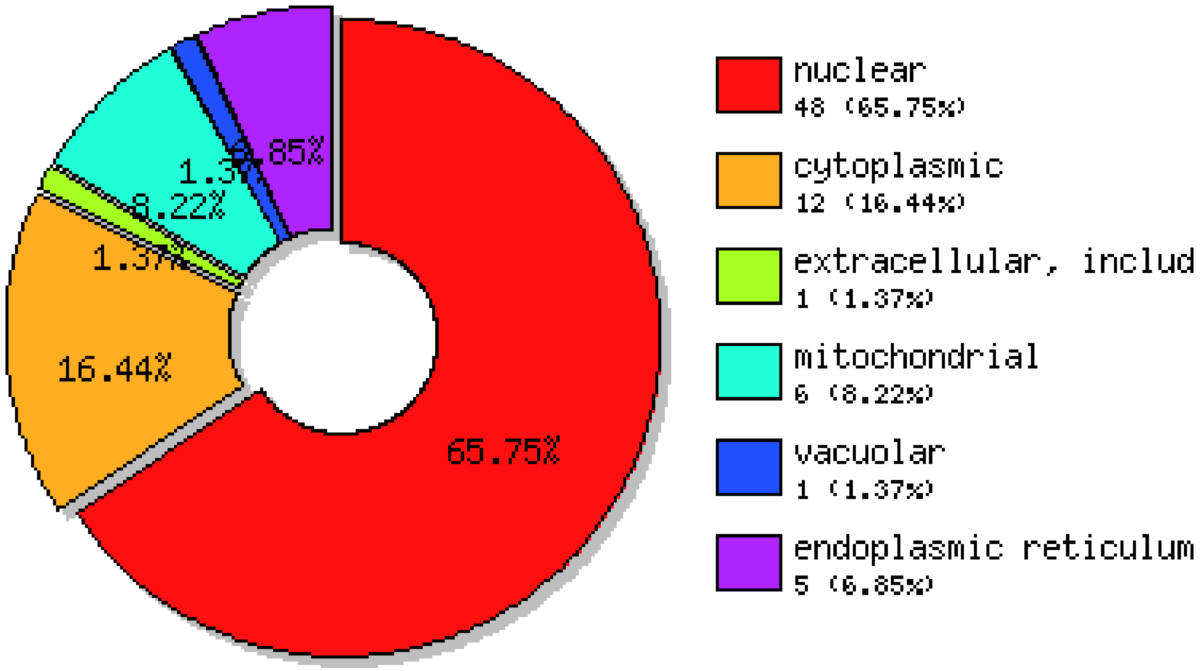

Location	Count	Ratio1
nuclear	48%	65.75%
cytoplasmic	12%	16.44%
extracellular, including cell wa	1%	1.37%
mitochondrial	6%	8.22%
vacuolar	1%	1.37%
endoplasmic reticulum	5%	6.85%

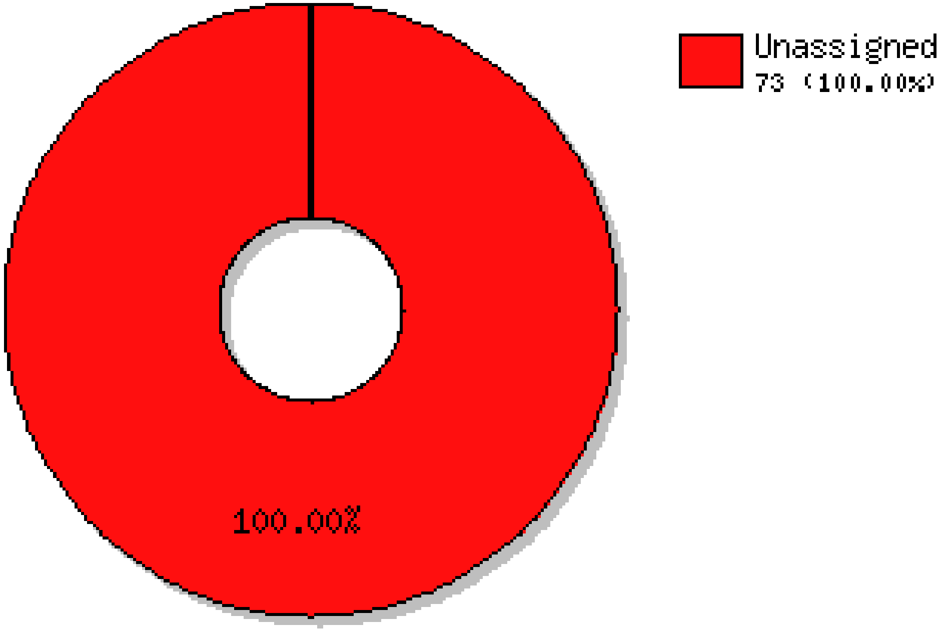

TF Family	Count	Ratio
Unassigned	73	0.00%

PolyN

Cellular Components

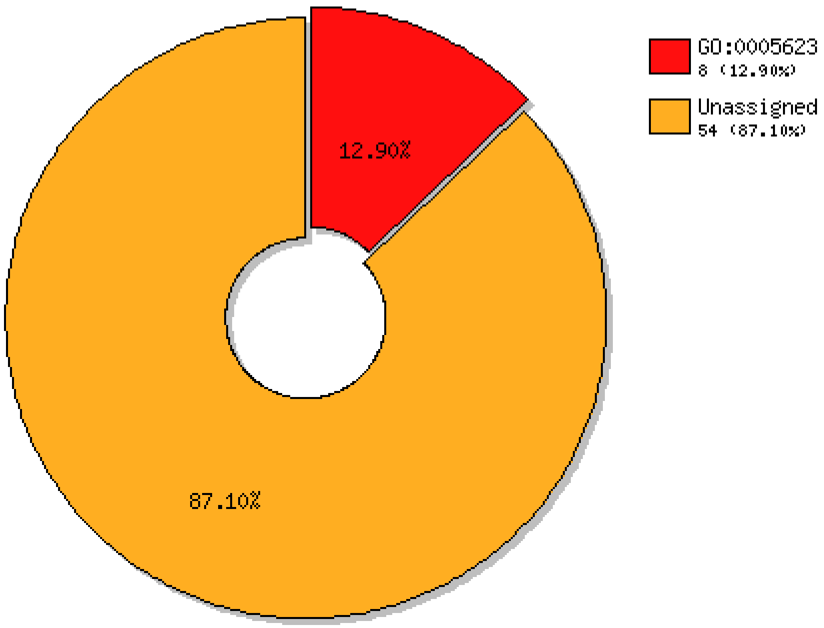

Molecular function

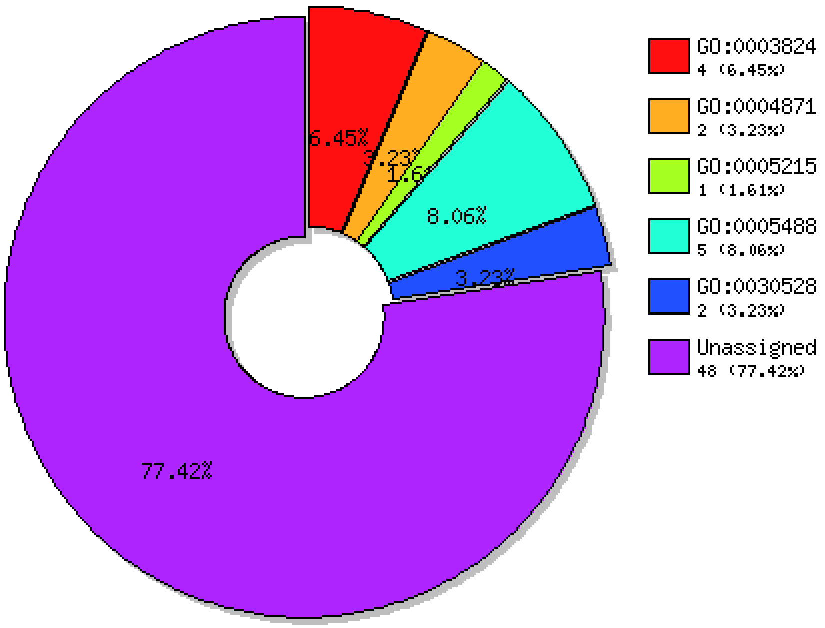

Biological Process

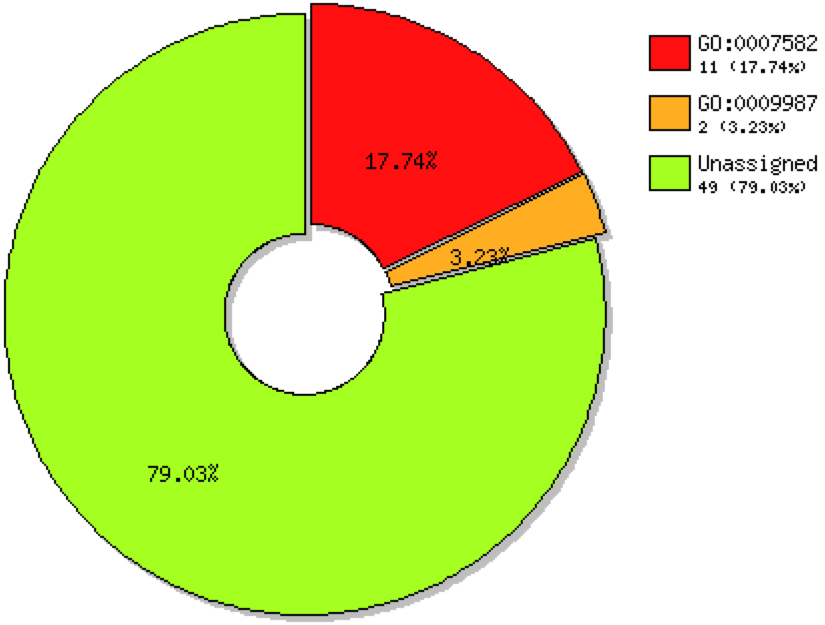

Gene_Ontology (GO:0003673)	0
biological_process (GO:0008150)	0
physiological process (GO:0007582)	11
cellular process (GO:0009987)	2
molecular_function (GO:0003674)	0
catalytic activity (GO:0003824)	4
signal transducer activity (GO:0004871)	2
transporter activity (GO:0005215)	1
binding (GO:0005488)	5
molecular_function unknown (GO:0005554)	0
transcription regulator activity (GO:0030528)	2
cellular_component (GO:0005575)	0
extracellular (GO:0005576)	0
cell (GO:0005623)	8
Unassigned (-)	44

PolyN

Subcellular Localization

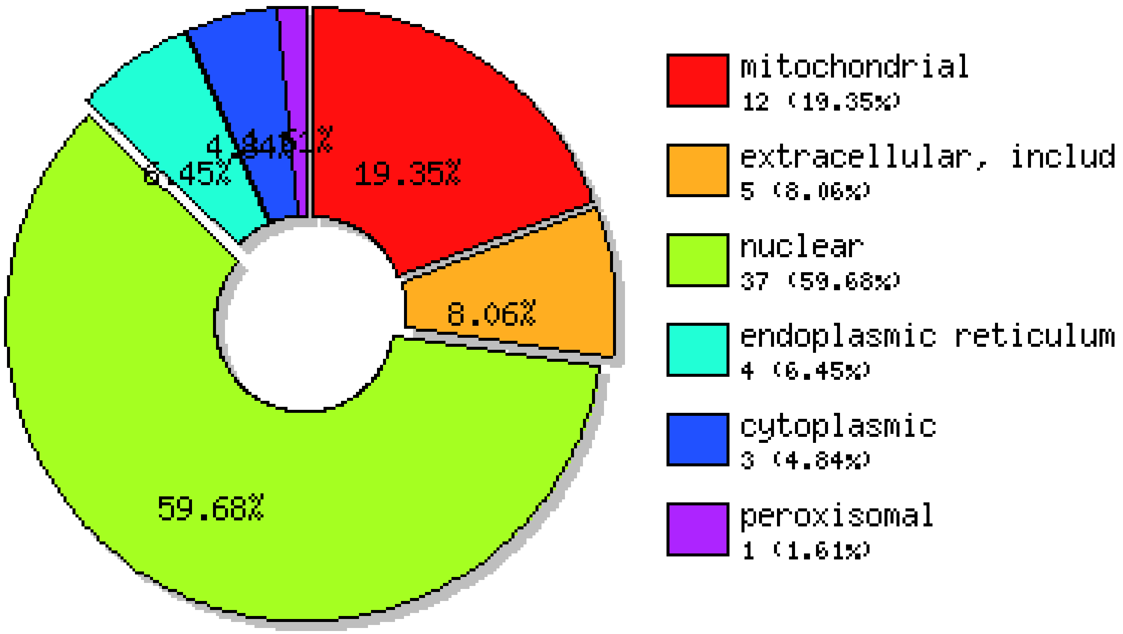

Location	Count	Ratio1
mitochondrial	12%	19.35%
extracellular, including cell wa	5%	8.06%
nuclear	37%	59.68%
endoplasmic reticulum	4%	6.45%
cytoplasmic	3%	4.84%
peroxisomal	1%	1.61%

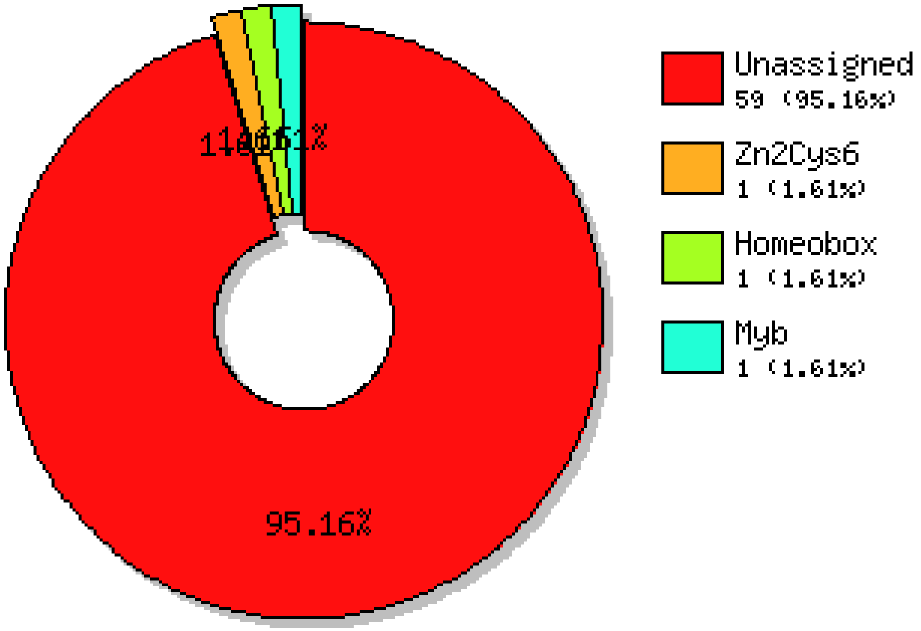

TF Family	Count	Ratio
Unassigned	59	-
Zn2Cys6	1	33.33%
Homeobox	1	33.33%
Myb	1	33.33%

PolyH

Cellular Components

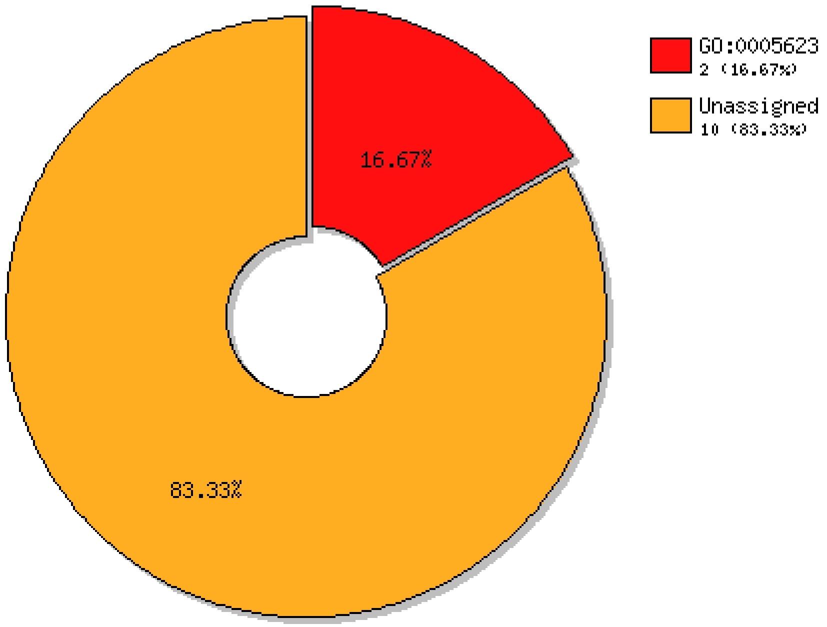

Molecular function

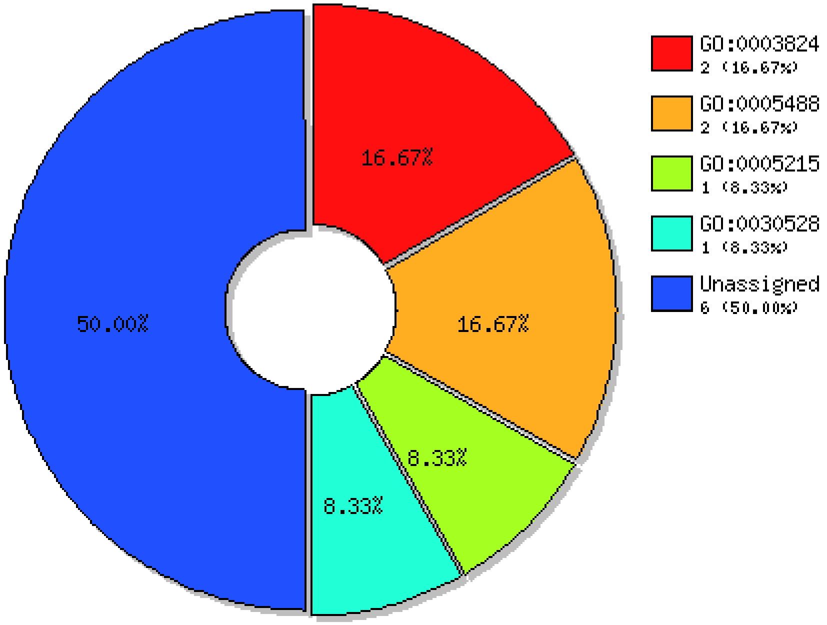

Biological Process

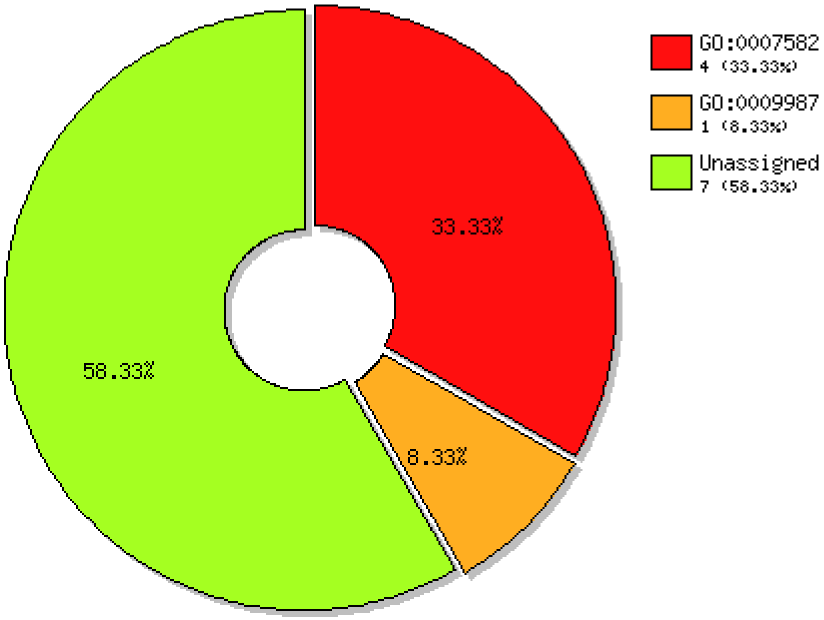

Gene_Ontology (GO:0003673)	0
biological_process (GO:0008150)	0
physiological process (GO:0007582)	4
cellular process (GO:0009987)	1
molecular_function (GO:0003674)	0
catalytic activity (GO:0003824)	2
binding (GO:0005488)	2
transporter activity (GO:0005215)	1
transcription regulator activity (GO:0030528)	1
cellular_component (GO:0005575)	0
cell (GO:0005623)	2
Unassigned (-)	7

PolyH **Subcellular Localization**

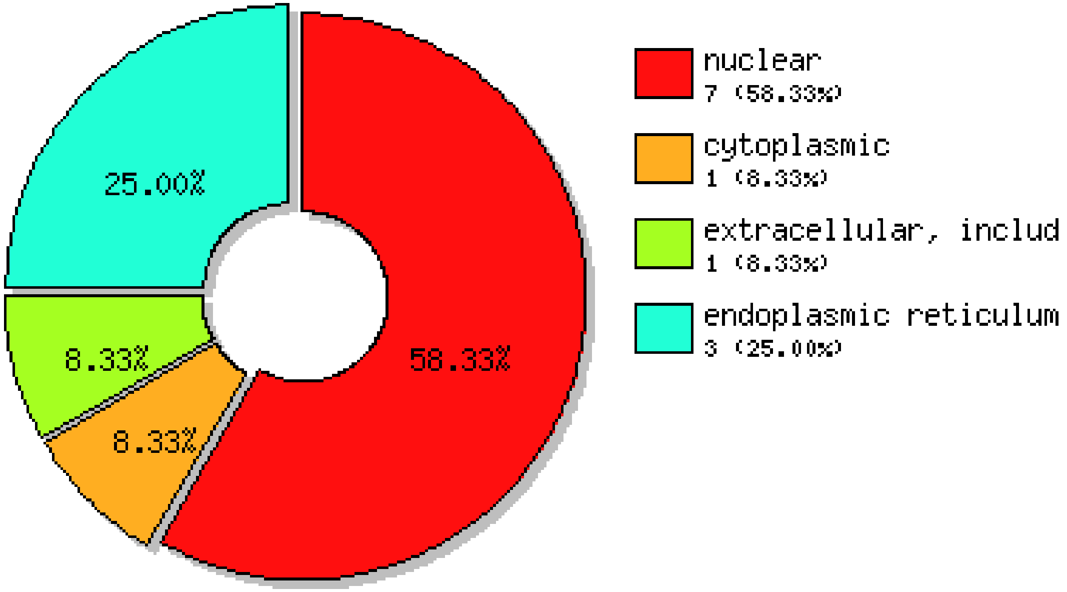

Location	Count	Ratio1
nuclear	7%	58.33%
cytoplasmic	1%	8.33%
extracellular, including cell wa	1%	8.33%
endoplasmic reticulum	3%	25.00%

PolyH **Transcription Factors**

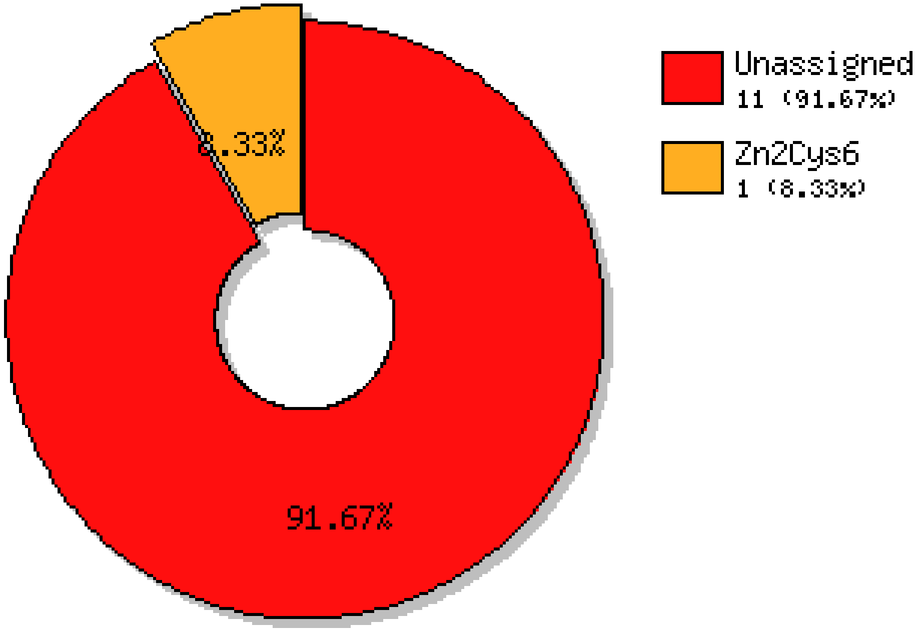

TF Family	Count	Ratio
Unassigned	11	-
Zn2Cys6	1	100.00%

PolyT

Cellular Components

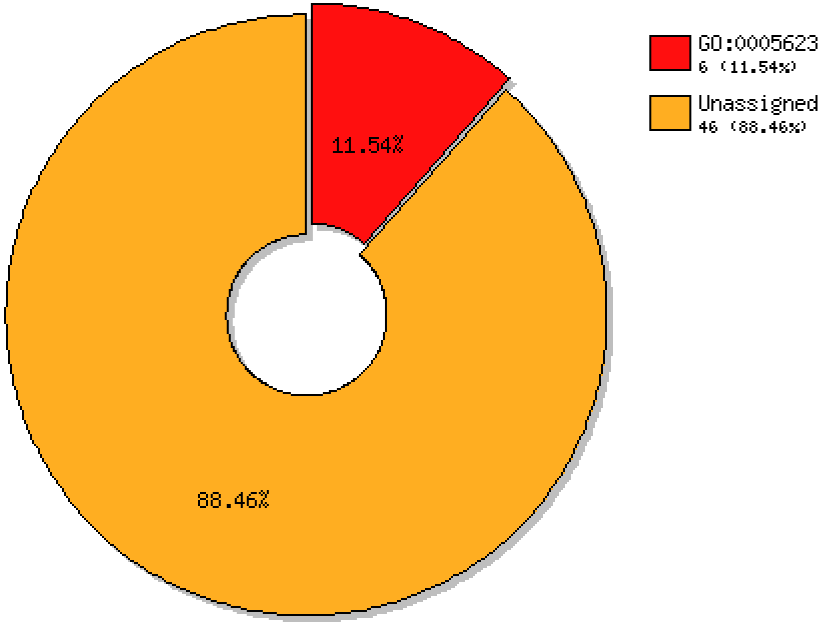

Molecular function

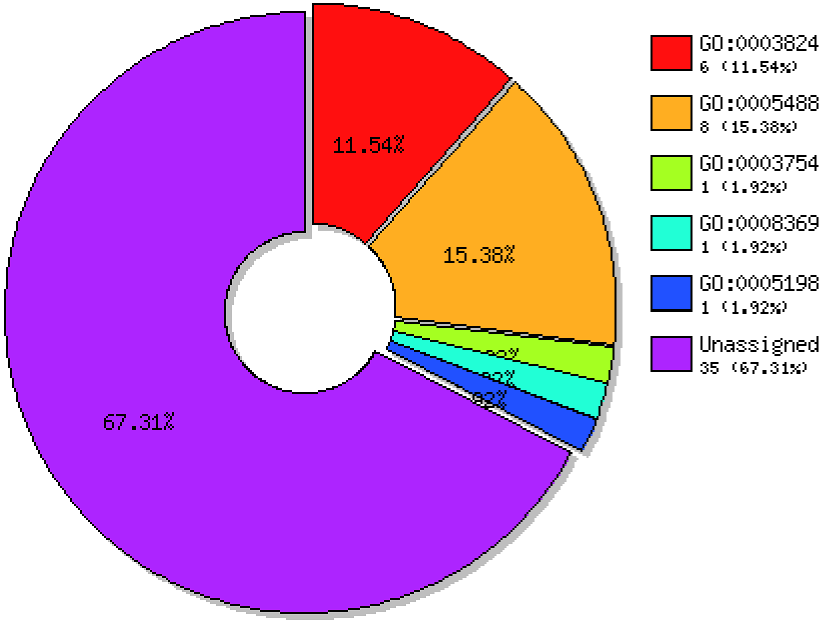

Biological Process

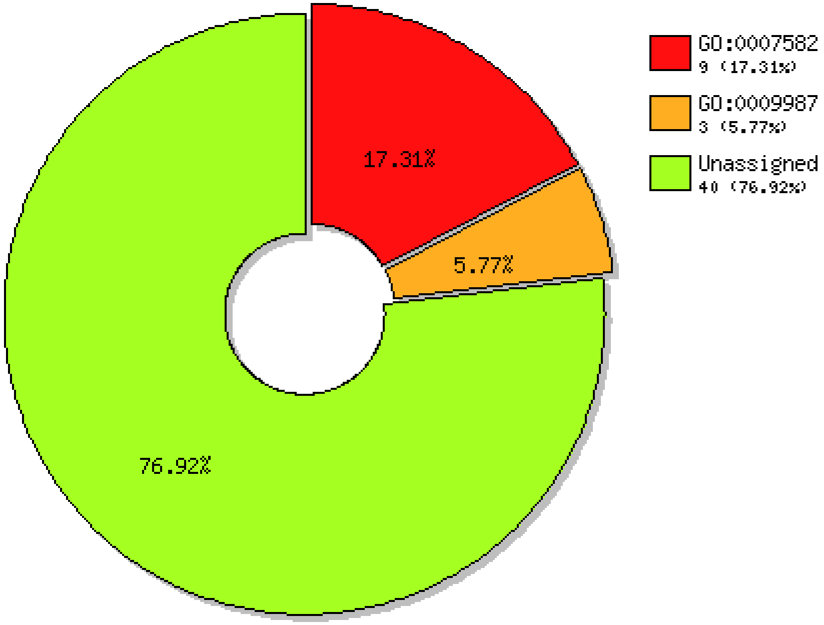

Gene_Ontology (GO:0003673)	0
molecular_function (GO:0003674)	0
catalytic activity (GO:0003824)	6
binding (GO:0005488)	8
chaperone activity (GO:0003754)	1
obsolete molecular function (GO:0008369)	1
structural molecule activity (GO:0005198)	1
cellular_component (GO:0005575)	0
cell (GO:0005623)	6
biological_process (GO:0008150)	0
physiological process (GO:0007582)	9
cellular process (GO:0009987)	3
Unassigned (-)	38

PolyT **Subcellular Localization**

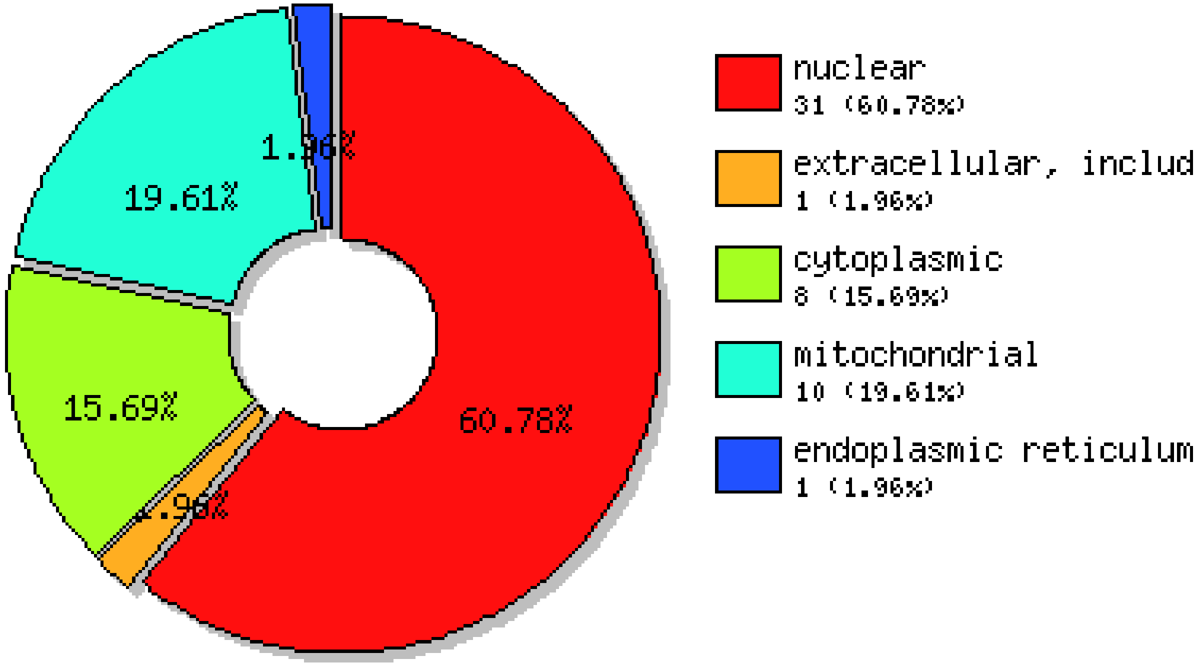

Location	Count	Ratio1
nuclear	31%	60.78%
extracellular, including cell wa	1%	1.96%
cytoplasmic	8%	15.69%
mitochondrial	10%	19.61%
endoplasmic reticulum	1%	1.96%

PolyT **Transcription Factors**

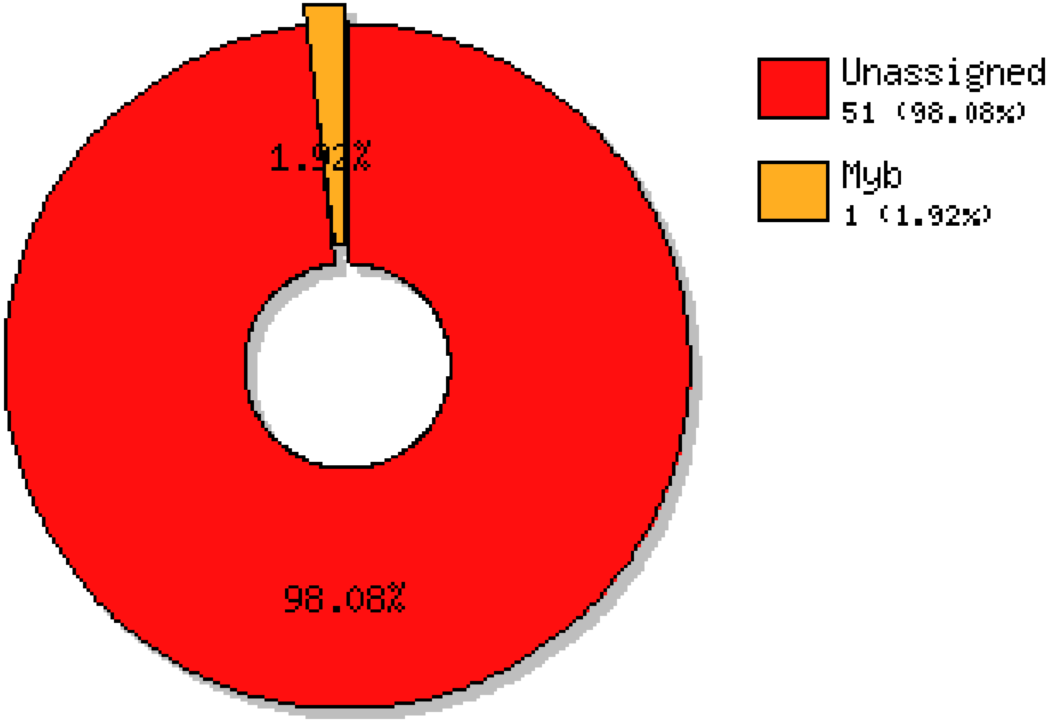

TF Family	Count	Ratio
Unassigned	51	-
Myb	1	100.00%

PolyK

Cellular Components

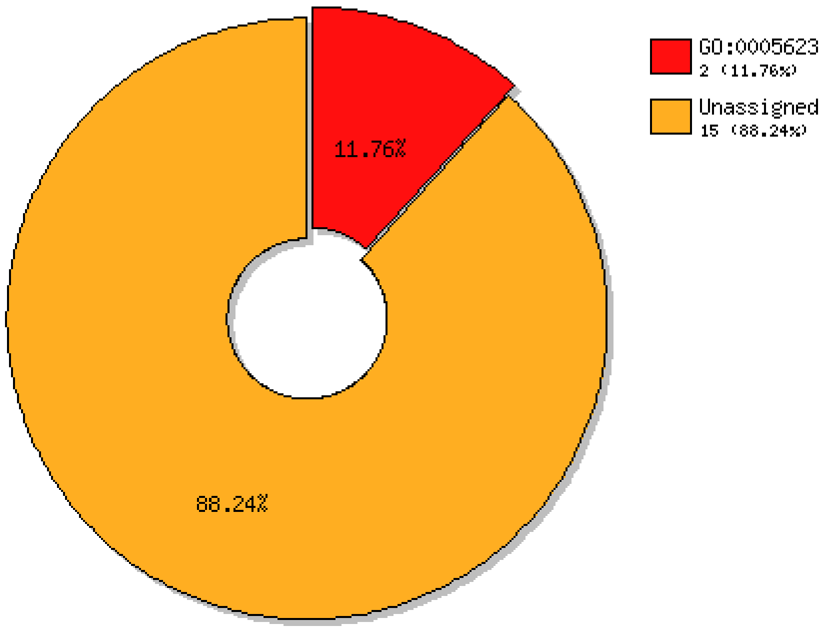

Molecular function

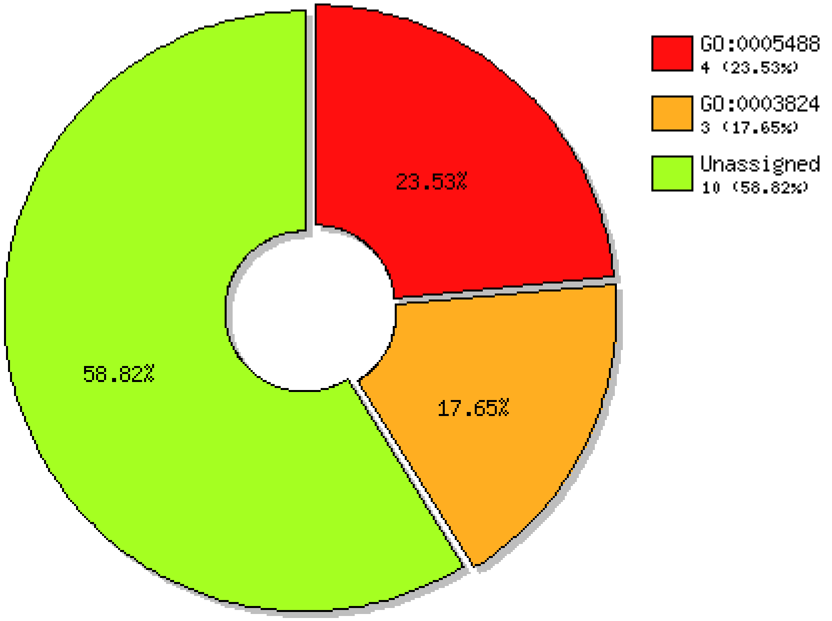

Biological Process

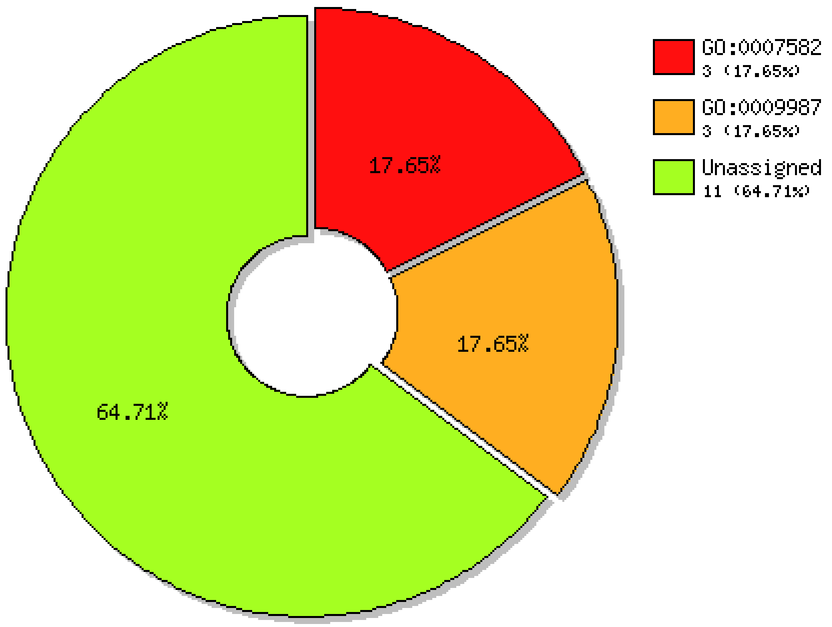

Gene_Ontology (GO:0003673)	0
molecular_function (GO:0003674)	0
binding (GO:0005488)	4
catalytic activity (GO:0003824)	3
cellular_component (GO:0005575)	0
cell (GO:0005623)	2
biological_process (GO:0008150)	0
physiological process (GO:0007582)	3
cellular process (GO:0009987)	3
Unassigned (-)	13

Subcellular Localization

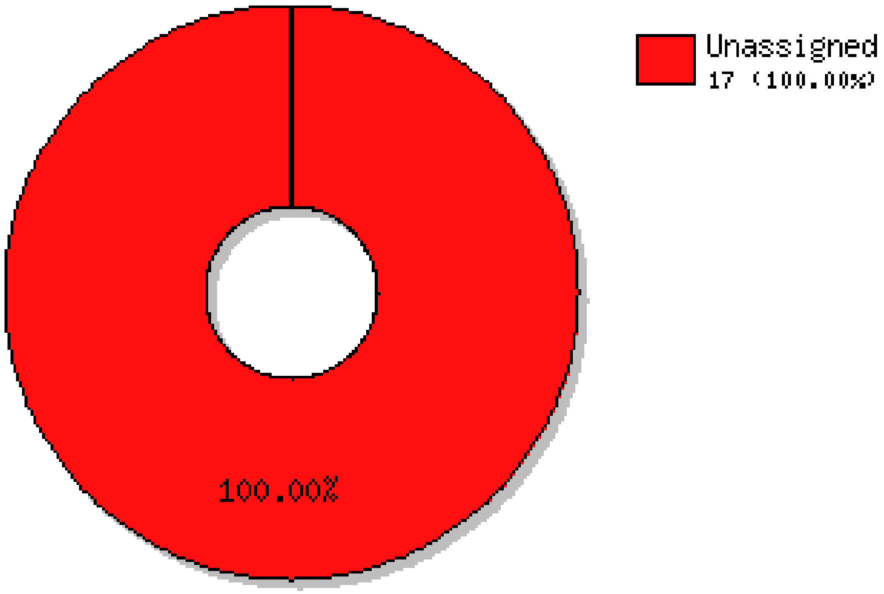

TF Family	Count	Ratio
Unassigned	17	0.00%

PolyL

Cellular Components

None

Molecular function

None

Biological Process

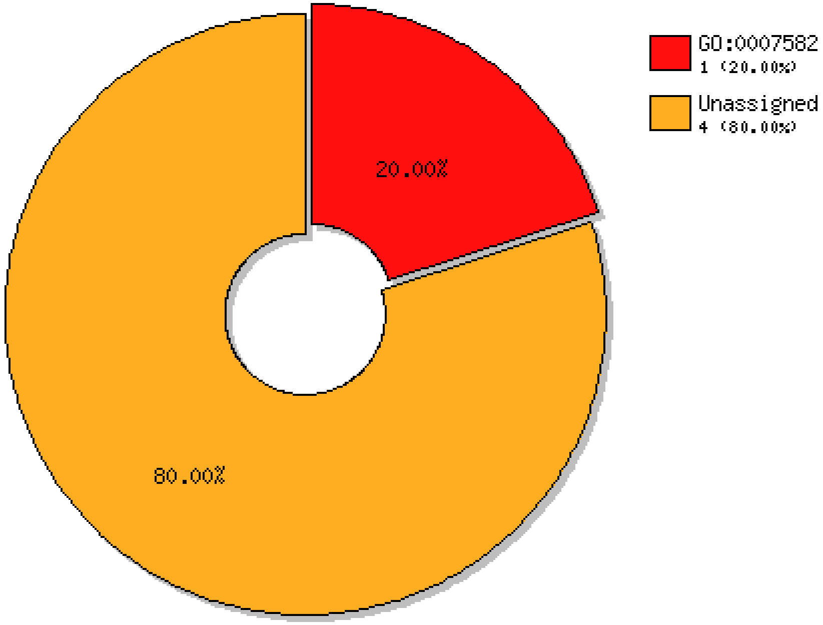

Gene_Ontology (GO:0003673)	0
biological_process (GO:0008150)	0
physiological_process (GO:0007582)	1
Unassigned (-)	4

PolyL

Subcellular Localization

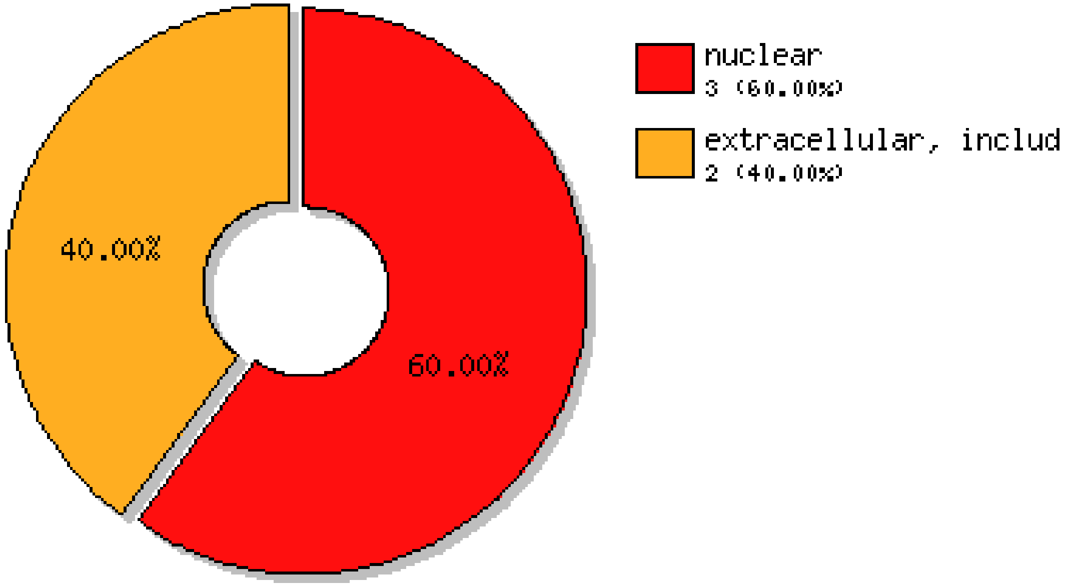

Location	Count	Ratio1
nuclear	3%	60.00%
extracellular, including cell wa	2%	40.00%

PolyL

Transcription Factors

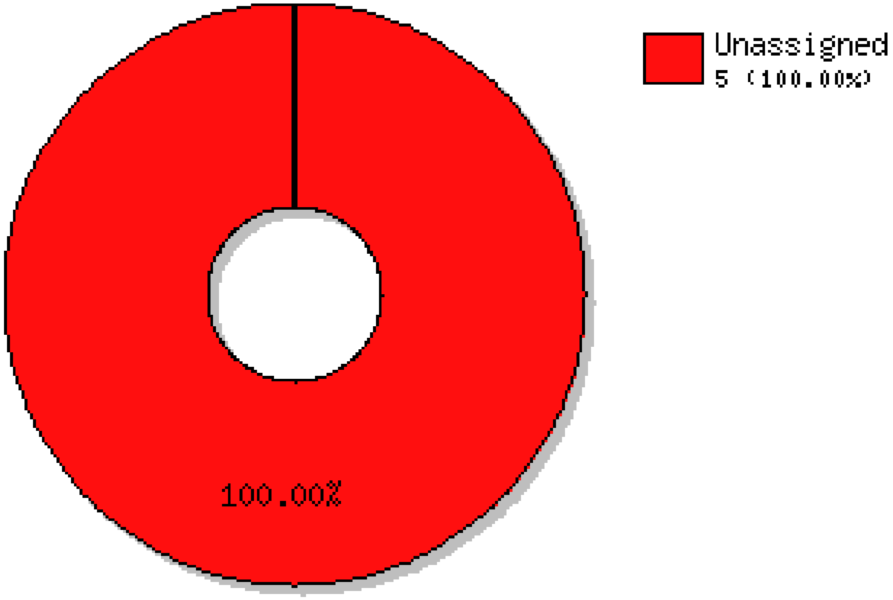

TF Family	Count	Ratio
Unassigned	5	0.00%

PolyV

Cellular Components

None

Molecular function

None

Biological Process

None

GO Term	Count
Unassigned (-)	6

PolyV Subcellular Localization

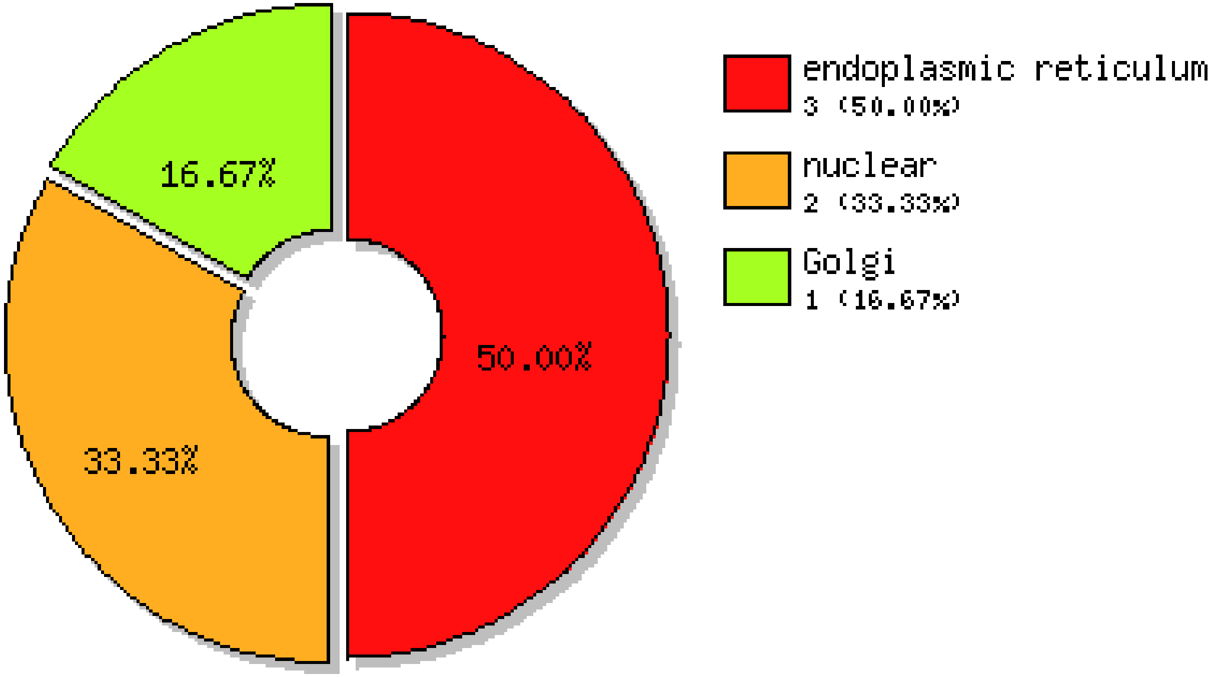

Location	Count	Ratio1
endoplasmic reticulum	3%	50.00%
nuclear	2%	33.33%
Golgi	1%	16.67%

PolyV

Transcription Factors

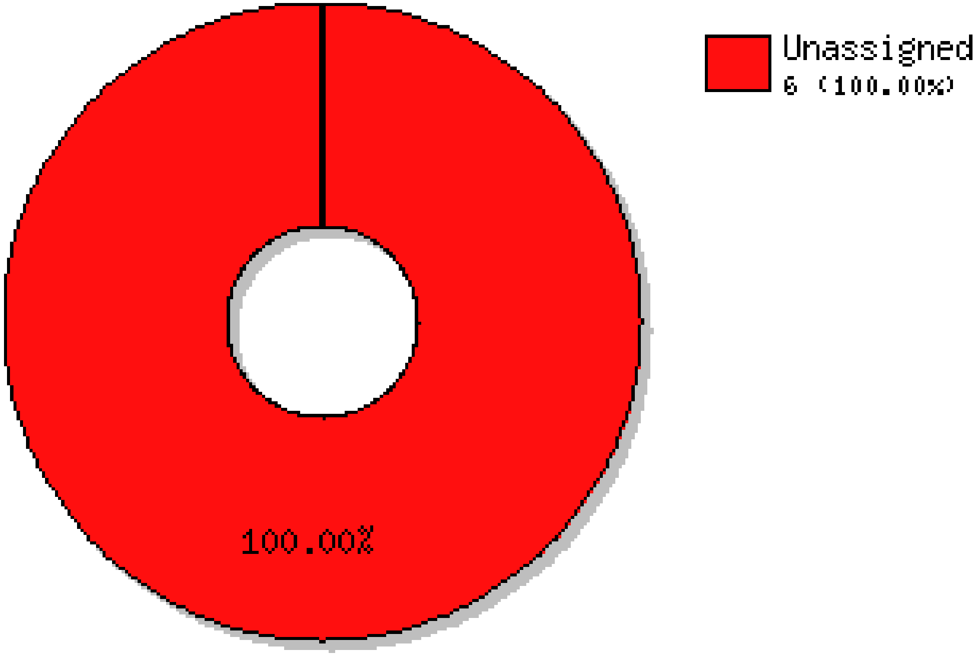

TF Family	Count	Ratio
Unassigned	600%	0.00%
