# Supplementary material for: Simple sequence repeats in Neurospora crassa: distribution, polymorphism and evolutionary inference
Source: BMC Genomics. 2008 Jan 23;9:31. doi: 10.1186/1471-2164-9-31 (PMC2257937; doi:10.1186/1471-2164-9-31)
Supplement: Additional file 3 — Abundant SSR types (AST) in the Neurospora crassa genome [file 1471-2164-9-31-S3.pdf]

Chromosome	Range -start	Range-end	Unit sequence	Length	Repeat number	Type
1	174803	174828	C	1	26	Exon
1	150670	150739	AAC/ACA/CAA	3	23.3	Exon
1	196826	196857	AAC/ACA/CAA	3	10.7	Exon
1	705422	705464	AAC/ACA/CAA	3	14.3	Exon
1	786655	786698	AAC/ACA/CAA	3	14.7	Exon
1	1218800	1218828	AAC/ACA/CAA	3	9.7	Exon
1	200004	200161	AAC/ACA/CAA	3	52.7	Exon
1	556811	556845	AAC/ACA/CAA	3	11.7	Exon
1	31217	31245	AAC/ACA/CAA	3	9.7	Exon
1	1588504	1588539	AAC/ACA/CAA	3	12	Exon
1	1588956	1588998	AAC/ACA/CAA	3	14.3	Exon
1	1659952	1660013	AAC/ACA/CAA	3	20.7	Exon
1	1664917	1664978	AAC/ACA/CAA	3	19.7	Exon
1	243512	243545	AAC/ACA/CAA	3	11.3	Exon
1	465446	465480	AAC/ACA/CAA	3	11.7	Exon
1	793643	793673	AAC/ACA/CAA	3	10.3	Exon
1	812709	812781	AAC/ACA/CAA	3	24.3	Exon
1	972786	972819	AAC/ACA/CAA	3	11.3	Exon
1	84020	84106	AAC/ACA/CAA	3	29	Exon
1	13170	13205	AAG/AGA/GAA	3	12	Exon
1	910242	910336	AAG/AGA/GAA	3	31.7	Exon
1	813986	814010	ACC/CCA/CAC	3	8.3	Exon
1	1285922	1285970	ACC/CCA/CAC	3	16.3	Exon
1	34248	34282	ACC/CCA/CAC	3	11.7	Exon
1	913443	913468	ACC/CCA/CAC	3	8.7	Exon
1	57367	7397	AGC/GCA/CAG	3	10.3	Exon
1	176294	176319	AGC/GCA/CAG	3	8.7	Exon
1	232922	232968	AGC/GCA/CAG	3	15.7	Exon
1	311750	311800	AGC/GCA/CAG	3	17	Exon
1	556844	556912	AGC/GCA/CAG	3	12	Exon
1	89779	89813	AGC/GCA/CAG	3	11.7	Exon
1	640786	640818	AGC/GCA/CAG	3	11	Exon
1	166482	166535	AGC/GCA/CAG	3	18	Exon
1	76409	76438	AGC/GCA/CAG	3	10	Exon
1	549706	549738	AGC/GCA/CAG	3	11	Exon
1	1221698	1221742	AGC/GCA/CAG	3	15	Exon
1	1525357	1525384	AGC/GCA/CAG	3	9.3	Exon
1	142869	142894	AGC/GCA/CAG	3	8.7	Exon
1	432843	432876	AGC/GCA/CAG	3	11.3	Exon
1	869996	870040	AGC/GCA/CAG	3	15	Exon
1	956127	956155	AGC/GCA/CAG	3	9.7	Exon
1	970951	970976	AGC/GCA/CAG	3	8.7	Exon
1	971272	971303	AGC/GCA/CAG	3	10.7	Exon
1	610318	610355	AGG/GGA/GAG	3	12.7	Exon
1	970376	970413	AGG/GGA/GAG	3	12.7	Exon
1	285607	285649	AGG/GGA/GAG	3	14.3	Exon
1	125930	125984	AGG/GGA/GAG	3	18.3	Exon
1	503362	503393	AGG/GGA/GAG	3	11	Exon
1	867775	867842	AGG/GGA/GAG	3	22.7	Exon
1	954265	954296	AGG/GGA/GAG	3	10.7	Exon
1	84087	84155	AGG/GGA/GAG	3	23	Exon
1	46563	46623	AGG/GGA/GAG	3	20	Exon
1	88248	88299	AGG/GGA/GAG	3	17.3	Exon
1	979862	979890	AGG/GGA/GAG	3	9.7	Exon
1	1491117	1491148	AGG/GGA/GAG	3	10.7	Exon
1	1518486	1518514	AGG/GGA/GAG	3	9.7	Exon
1	695375	695412	AGG/GGA/GAG	3	12.7	Exon
1	888975	889005	AGG/GGA/GAG	3	10.3	Exon
1	923309	923333	AGG/GGA/GAG	3	8.3	Exon
1	138437	138523	AGG/GGA/GAG	3	29	Exon
1	158041	158074	AGG/GGA/GAG	3	11.3	Exon
1	247476	247508	ATG/TGA/GAT	3	11	Exon
1	162848	162884	ATG/TGA/GAT	3	12.3	Exon
1	588474	588511	ATG/TGA/GAT	3	12.7	Exon
1	1395764	1395789	ATG/TGA/GAT	3	8.7	Exon
1	24948	24978	CCT/CTC/TCC	3	10.3	Exon
1	57126	57194	CCT/CTC/TCC	3	13	Exon
1	773786	773862	CCT/CTC/TCC	3	25.7	Exon
1	28954	28989	CCT/CTC/TCC	3	12	Exon
1	8763	8813	CCT/CTC/TCC	3	17	Exon
1	35307	35340	CCT/CTC/TCC	3	11.3	Exon
1	412838	412890	CCT/CTC/TCC	3	17.7	Exon
1	959593	959688	CCT/CTC/TCC	3	32	Exon
1	13096	13146	CCT/CTC/TCC	3	17.3	Exon
1	397564	397696	CCT/CTC/TCC	3	44.3	Exon
1	470382	470423	CCT/CTC/TCC	3	14	Exon
1	188862	188893	CCT/CTC/TCC	3	10.7	Exon
1	456942	456979	CCT/CTC/TCC	3	12.7	Exon
1	66500	66744	CTT/TTC/TCT	3	82.3	Exon
1	57096	57124	CTT/TTC/TCT	3	9.7	Exon
1	1071068	1071122	CTT/TTC/TCT	3	18.3	Exon
1	77628	77668	CTT/TTC/TCT	3	13.7	Exon
1	1187219	1187253	CTT/TTC/TCT	3	11.7	Exon
1	915525	915560	CTT/TTC/TCT	3	12	Exon
1	58270	58390	GTT/TTG/TGT	3	40.3	Exon
1	61493	1576	GTT/TTG/TGT	3	15.3	Exon
1	712041	712065	GTT/TTG/TGT	3	8.3	Exon
1	730978	731009	GTT/TTG/TGT	3	10.7	Exon
1	1100945	1100983	GTT/TTG/TGT	3	13	Exon
1	1267385	1267483	GTT/TTG/TGT	3	32.7	Exon
1	1307281	1307340	GTT/TTG/TGT	3	20	Exon
1	255912	255936	GTT/TTG/TGT	3	8.3	Exon
1	725296	725359	GTT/TTG/TGT	3	21.7	Exon
1	688053	688105	GTT/TTG/TGT	3	17.7	Exon
1	24340	24382	GTT/TTG/TGT	3	14.3	Exon
1	277495	277591	GTT/TTG/TGT	3	32.3	Exon
1	711489	711526	GTT/TTG/TGT	3	12.7	Exon
1	1270782	1270927	GTT/TTG/TGT	3	48.7	Exon
1	686007	686033	GTT/TTG/TGT	3	9	Exon
1	868832	868862	GTT/TTG/TGT	3	10.3	Exon
1	870105	870131	GTT/TTG/TGT	3	9	Exon
1	921696	921726	GTT/TTG/TGT	3	10.3	Exon
1	171953	172079	GTT/TTG/TGT	3	11.7	Exon
1	124984	125016	GCT/CTG/TGC	3	11	Exon
1	163032	163056	GCT/CTG/TGC	3	8.3	Exon
1	624769	624801	GCT/CTG/TGC	3	11	Exon
1	778731	778758	GCT/CTG/TGC	3	9.3	Exon
1	1069154	1069237	GCT/CTG/TGC	3	28	Exon
1	1263629	1263660	GCT/CTG/TGC	3	10.7	Exon
1	119953	119980	GCT/CTG/TGC	3	9.3	Exon
1	672909	672947	GCT/CTG/TGC	3	13	Exon
1	876242	876266	GCT/CTG/TGC	3	8.3	Exon
1	32305	32344	GCT/CTG/TGC	3	13.3	Exon
1	814636	814666	GCT/CTG/TGC	3	10.3	Exon
1	953976	954025	GCT/CTG/TGC	3	16.7	Exon
1	86079	86106	GCT/CTG/TGC	3	9.3	Exon
1	751361	751385	GCT/CTG/TGC	3	8.3	Exon
1	867491	867534	GCT/CTG/TGC	3	14.7	Exon
1	867998	868029	GCT/CTG/TGC	3	10.7	Exon
1	892198	892223	GCT/CTG/TGC	3	8.7	Exon
1	61814	61848	GCT/CTG/TGC	3	11.7	Exon
1	1208731	1208856	GGT/GTG/TGG	3	42	Exon
1	870602	870684	GGT/GTG/TGG	3	28.3	Exon
1	892341	892374	GGT/GTG/TGG	3	11.3	Exon
1	906588	906624	GGT/GTG/TGG	3	12.3	Exon
1	548484	548509	GGT/GTG/TGG	3	8.7	Exon
1	648688	648740	GGT/GTG/TGG	3	17.7	Exon
1	924185	924219	GGT/GTG/TGG	3	11.7	Exon
1	63515	63548	GGT/GTG/TGG	3	11.3	Exon
1	181576	181626	GGT/GTG/TGG	3	16.7	Exon
1	38240	38278	GGT/GTG/TGG	3	13	Exon
1	38035	38066	A	1	32	Intergenic
1	66247	66282	A	1	36	Intergenic
1	10762	10803	A	1	42	Intergenic
1	130976	131015	A	1	40	Intergenic
1	206693	206717	A	1	25	Intergenic
1	61115	61153	A	1	39	Intergenic
1	96211	96283	A	1	73	Intergenic
1	77501	77542	A	1	42	Intergenic
1	346793	346830	A	1	38	Intergenic
1	347214	347269	A	1	56	Intergenic
1	526282	526309	A	1	28	Intergenic
1	833106	833151	A	1	46	Intergenic
1	844577	844614	A	1	38	Intergenic

Chromosome	Range -start	Range-end	Unit sequence	Length	Repeat number	Type
1	869098	869151	A	1	54	Intergenic
1	903422	903465	A	1	44	Intergenic
1	1149393	1149431	A	1	39	Intergenic
1	1271855	1271907	A	1	53	Intergenic
1	43517	43544	A	1	28	Intergenic
1	65257	65283	A	1	27	Intergenic
1	344198	344292	A	1	95	Intergenic
1	358911	358939	A	1	29	Intergenic
1	409844	409882	A	1	39	Intergenic
1	436551	436591	A	1	41	Intergenic
1	523312	523337	A	1	26	Intergenic
1	1011561	1011607	A	1	47	Intergenic
1	1362	1423	A	1	62	Intergenic
1	40881	40910	A	1	30	Intergenic
1	72492	72550	A	1	59	Intergenic
1	93116	93169	A	1	54	Intergenic
1	9696	9731	A	1	36	Intergenic
1	9880	9910	A	1	31	Intergenic
1	41341	41365	A	1	25	Intergenic
1	19052	19092	A	1	41	Intergenic
1	34884	34934	A	1	51	Intergenic
1	15213	15243	A	1	31	Intergenic
1	80514	80596	A	1	83	Intergenic
1	84867	84896	A	1	30	Intergenic
1	170364	170390	A	1	27	Intergenic
1	193006	193059	A	1	54	Intergenic
1	252752	252779	A	1	28	Intergenic
1	532661	532697	A	1	37	Intergenic
1	603220	603256	A	1	37	Intergenic
1	103230	103257	A	1	28	Intergenic
1	218424	218491	A	1	68	Intergenic
1	55459	55492	A	1	34	Intergenic
1	77442	77469	A	1	28	Intergenic
1	106985	107015	A	1	31	Intergenic
1	220978	221016	A	1	39	Intergenic
1	270436	270461	A	1	26	Intergenic
1	288312	288356	A	1	45	Intergenic
1	316975	317013	A	1	39	Intergenic
1	416816	416857	A	1	42	Intergenic
1	465184	465213	A	1	30	Intergenic
1	690990	691032	A	1	43	Intergenic
1	691169	691195	A	1	27	Intergenic
1	758624	758659	A	1	36	Intergenic
1	769389	769426	A	1	38	Intergenic
1	778669	778709	A	1	41	Intergenic
1	795397	795429	A	1	33	Intergenic
1	845970	845994	A	1	25	Intergenic
1	953246	953294	A	1	49	Intergenic
1	970617	970657	A	1	41	Intergenic
1	1120815	1120839	A	1	25	Intergenic
1	1132651	1132702	A	1	52	Intergenic
1	1432402	1432447	A	1	46	Intergenic
1	1456462	1456496	A	1	35	Intergenic
1	1553770	1553822	A	1	53	Intergenic
1	1611117	1611142	A	1	26	Intergenic
1	1769366	1769396	A	1	31	Intergenic
1	31177	31203	A	1	27	Intergenic
1	75107	75155	A	1	49	Intergenic
1	158773	158816	A	1	44	Intergenic
1	179457	179481	A	1	25	Intergenic
1	377592	377623	A	1	32	Intergenic
1	538123	538148	A	1	26	Intergenic
1	547099	547154	A	1	56	Intergenic
1	561958	561983	A	1	26	Intergenic
1	646410	646441	A	1	32	Intergenic
1	662747	662776	A	1	30	Intergenic
1	727520	727559	A	1	40	Intergenic
1	788737	788775	A	1	39	Intergenic
1	805441	805467	A	1	27	Intergenic
1	814182	814222	A	1	41	Intergenic
1	862960	862984	A	1	25	Intergenic
1	906708	906745	A	1	38	Intergenic

Chromosome	Range -start	Range-end	Unit sequence	Length	Repeat number	Type
1	13696	13722	A	1	27	Intergenic
1	32916	32971	A	1	56	Intergenic
1	33164	33193	A	1	30	Intergenic
1	104274	104303	A	1	30	Intergenic
1	16757	16782	C	1	26	Intergenic
1	1028324	1028351	C	1	28	Intergenic
1	1259156	1259186	C	1	31	Intergenic
1	17995	18019	C	1	25	Intergenic
1	423714	423739	C	1	26	Intergenic
1	652833	652857	C	1	25	Intergenic
1	164226	164258	C	1	33	Intergenic
1	819444	819468	C	1	25	Intergenic
1	1104979	1105012	C	1	34	Intergenic
1	1429186	1429215	C	1	30	Intergenic
1	1592209	1592235	C	1	27	Intergenic
1	557692	557718	C	1	27	Intergenic
1	174388	174415	C	1	28	Intergenic
1	8929	8954	G	1	26	Intergenic
1	131326	131353	G	1	28	Intergenic
1	214976	215004	G	1	29	Intergenic
1	1082204	1082235	G	1	32	Intergenic
1	1051982	1052007	G	1	26	Intergenic
1	273864	273888	G	1	25	Intergenic
1	292009	292037	G	1	29	Intergenic
1	483071	483099	G	1	29	Intergenic
1	584000	584024	G	1	25	Intergenic
1	912173	912197	G	1	25	Intergenic
1	282641	282668	G	1	28	Intergenic
1	820896	820925	G	1	30	Intergenic
1	1218678	1218709	G	1	32	Intergenic
1	1612752	1612785	G	1	34	Intergenic
1	1743780	1743807	G	1	28	Intergenic
1	21390	21415	T	1	26	Intergenic
1	75388	75432	T	1	45	Intergenic
1	110946	111022	T	1	77	Intergenic
1	118947	118994	T	1	48	Intergenic
1	230780	230834	T	1	55	Intergenic
1	268577	268602	T	1	26	Intergenic
1	62221	62257	T	1	37	Intergenic
1	95576	95615	T	1	40	Intergenic
1	148571	148598	T	1	28	Intergenic
1	297474	297511	T	1	38	Intergenic
1	313343	313383	T	1	41	Intergenic
1	411806	411845	T	1	40	Intergenic
1	662072	662122	T	1	51	Intergenic
1	712502	712545	T	1	44	Intergenic
1	752105	752157	T	1	53	Intergenic
1	1060121	1060147	T	1	27	Intergenic
1	1147847	1147895	T	1	49	Intergenic
1	118454	118481	T	1	28	Intergenic
1	1222890	1222929	T	1	40	Intergenic
1	1238036	1238076	T	1	41	Intergenic
1	24316	24365	T	1	50	Intergenic
1	157465	157509	T	1	45	Intergenic
1	170942	170980	T	1	39	Intergenic
1	227652	227701	T	1	50	Intergenic
1	394769	394824	T	1	56	Intergenic
1	396662	396713	T	1	52	Intergenic
1	405169	405197	T	1	29	Intergenic
1	422551	422602	T	1	52	Intergenic
1	430766	430815	T	1	50	Intergenic
1	519596	519634	T	1	39	Intergenic
1	591458	591486	T	1	29	Intergenic
1	854634	854675	T	1	42	Intergenic
1	865645	865676	T	1	32	Intergenic
1	990000	990043	T	1	44	Intergenic
1	991289	991337	T	1	49	Intergenic
1	38800	38829	T	1	30	Intergenic
1	67051	67080	T	1	30	Intergenic
1	56114	56146	T	1	33	Intergenic
1	56294	56323	T	1	30	Intergenic
1	22788	22855	T	1	68	Intergenic

Chromosome	Range -start	Range-end	Unit sequence	Length	Repeat number	Type
1	22197	22239	T	1	43	Intergenic
1	124424	124458	T	1	35	Intergenic
1	583968	583996	T	1	29	Intergenic
1	651412	651439	T	1	28	Intergenic
1	781599	781662	T	1	64	Intergenic
1	809976	810028	T	1	53	Intergenic
1	838130	838157	T	1	28	Intergenic
1	849476	849540	T	1	65	Intergenic
1	913297	913322	T	1	26	Intergenic
1	65502	65529	T	1	28	Intergenic
1	144901	144928	T	1	28	Intergenic
1	243026	243056	T	1	31	Intergenic
1	308803	308847	T	1	45	Intergenic
1	108305	108354	T	1	50	Intergenic
1	22413	22450	T	1	38	Intergenic
1	84679	84707	T	1	29	Intergenic
1	123095	123133	T	1	39	Intergenic
1	135413	135464	T	1	52	Intergenic
1	195871	195899	T	1	29	Intergenic
1	46064	46114	T	1	51	Intergenic
1	540790	540833	T	1	44	Intergenic
1	672057	672096	T	1	40	Intergenic
1	838590	838617	T	1	28	Intergenic
1	933469	933500	T	1	32	Intergenic
1	951224	951264	T	1	41	Intergenic
1	1038370	1038418	T	1	49	Intergenic
1	1051001	1051050	T	1	50	Intergenic
1	1110106	1110179	T	1	74	Intergenic
1	1192231	1192261	T	1	31	Intergenic
1	1349524	1349563	T	1	40	Intergenic
1	1441535	1441566	T	1	32	Intergenic
1	1472800	1472827	T	1	28	Intergenic
1	1505572	1505610	T	1	39	Intergenic
1	1571474	1571502	T	1	29	Intergenic
1	1626054	1626134	T	1	81	Intergenic
1	1665938	1665971	T	1	34	Intergenic
1	1737246	1737274	T	1	29	Intergenic
1	34398	34446	T	1	49	Intergenic
1	110352	110410	T	1	59	Intergenic
1	144036	144074	T	1	39	Intergenic
1	159172	159203	T	1	32	Intergenic
1	200448	200500	T	1	53	Intergenic
1	367600	367646	T	1	47	Intergenic
1	528465	528516	T	1	52	Intergenic
1	537377	537408	T	1	32	Intergenic
1	557956	557992	T	1	37	Intergenic
1	603657	603711	T	1	55	Intergenic
1	619471	619522	T	1	52	Intergenic
1	702745	702781	T	1	37	Intergenic
1	729783	729835	T	1	53	Intergenic
1	932482	932522	T	1	41	Intergenic
1	1544	1586	T	1	43	Intergenic
1	131683	131710	T	1	28	Intergenic
1	132794	132851	T	1	58	Intergenic
1	267537	267564	AC/CA	2	14	Intergenic
1	896071	896119	AC/CA	2	25	Intergenic
1	939689	939736	AC/CA	2	24	Intergenic
1	882685	882716	AC/CA	2	16	Intergenic
1	116974	116998	AC/CA	2	12.5	Intergenic
1	129598	129630	AC/CA	2	16.5	Intergenic
1	636597	636632	AC/CA	2	18	Intergenic
1	715624	715670	AC/CA	2	23.5	Intergenic
1	164562	164596	AC/CA	2	17.5	Intergenic
1	692739	692771	AG/GA	2	16.5	Intergenic
1	71593	71625	AG/GA	2	16.5	Intergenic
1	322593	322621	AG/GA	2	14.5	Intergenic
1	662756	662788	AG/GA	2	17.5	Intergenic
1	50586	50619	AG/GA	2	17	Intergenic
1	273170	273203	AG/GA	2	17	Intergenic
1	304034	304089	AG/GA	2	28	Intergenic
1	936391	936417	AG/GA	2	13.5	Intergenic
1	73889	73925	AG/GA	2	18.5	Intergenic

Chromosome	Range -start	Range-end	Unit sequence	Length	Repeat number	Type
1	164596	164660	AG/GA	2	15.5	Intergenic
1	959882	959906	CT/TC	2	12.5	Intergenic
1	1258962	1259042	CT/TC	2	40.5	Intergenic
1	1302406	1302430	CT/TC	2	12.5	Intergenic
1	450290	450315	CT/TC	2	13	Intergenic
1	839521	839554	CT/TC	2	17	Intergenic
1	137780	137824	GT/TG	2	22.5	Intergenic
1	1149964	1149995	GT/TG	2	16	Intergenic
1	182807	182847	GT/TG	2	20.5	Intergenic
1	347257	347286	GT/TG	2	15	Intergenic
1	761405	761441	GT/TG	2	18.5	Intergenic
1	498629	498664	GT/TG	2	18	Intergenic
1	116446	116474	GT/TG	2	14.5	Intergenic
1	159700	159724	AAC/ACA/CAA	3	8.3	Intergenic
1	540577	540601	AAC/ACA/CAA	3	8.3	Intergenic
1	66556	66597	AAC/ACA/CAA	3	14	Intergenic
1	47365	47407	AAC/ACA/CAA	3	14.3	Intergenic
1	679283	679345	AAC/ACA/CAA	3	21	Intergenic
1	1365008	1365054	AAC/ACA/CAA	3	15.7	Intergenic
1	44841	44867	AAC/ACA/CAA	3	9	Intergenic
1	733498	733528	AAC/ACA/CAA	3	10.3	Intergenic
1	145825	145858	AAG/AGA/GAA	3	11.3	Intergenic
1	82234	82304	AAG/AGA/GAA	3	24	Intergenic
1	1258804	1258836	AAG/AGA/GAA	3	11	Intergenic
1	926896	926935	AAG/AGA/GAA	3	13.3	Intergenic
1	1056415	1056474	AAG/AGA/GAA	3	20	Intergenic
1	720782	720818	AAG/AGA/GAA	3	12.3	Intergenic
1	244860	244891	ACC/CCA/CAC	3	10.7	Intergenic
1	343952	343982	ACC/CCA/CAC	3	10.3	Intergenic
1	503771	503796	AGC/GCA/CAG	3	8.7	Intergenic
1	584053	584086	AGC/GCA/CAG	3	11.3	Intergenic
1	734373	734405	AGC/GCA/CAG	3	11	Intergenic
1	748475	748523	AGC/GCA/CAG	3	16.3	Intergenic
1	788985	789012	AGC/GCA/CAG	3	9.3	Intergenic
1	175900	175941	AGG/GGA/GAG	3	14	Intergenic
1	1247202	1247252	AGG/GGA/GAG	3	17	Intergenic
1	358753	358870	AGG/GGA/GAG	3	20.3	Intergenic
1	109668	109706	AGG/GGA/GAG	3	13	Intergenic
1	30295	30329	AGG/GGA/GAG	3	11.7	Intergenic
1	80037	80074	AGG/GGA/GAG	3	12.3	Intergenic
1	67606	67634	ATG/TGA/GAT	3	9.7	Intergenic
1	358696	358720	ATG/TGA/GAT	3	8.3	Intergenic
1	493915	493940	ATG/TGA/GAT	3	8.7	Intergenic
1	547868	547906	ATG/TGA/GAT	3	13	Intergenic
1	935933	935966	ATG/TGA/GAT	3	11	Intergenic
1	550900	550949	ATG/TGA/GAT	3	16.7	Intergenic
1	217959	217994	CCT/CTC/TCC	3	12	Intergenic
1	70887	70913	CCT/CTC/TCC	3	9	Intergenic
1	629657	629683	CCT/CTC/TCC	3	9	Intergenic
1	733997	734023	CCT/CTC/TCC	3	9	Intergenic
1	862765	863065	CCT/CTC/TCC	3	17.3	Intergenic
1	67800	67840	CTT/TTC/TCT	3	13.7	Intergenic
1	877613	877653	CTT/TTC/TCT	3	13.7	Intergenic
1	283443	283467	CTT/TTC/TCT	3	8.3	Intergenic
1	1648807	1648894	CTT/TTC/TCT	3	29	Intergenic
1	414141	414186	CTT/TTC/TCT	3	15.3	Intergenic
1	748687	748729	CTT/TTC/TCT	3	13.7	Intergenic
1	86816	86845	GTT/TTG/TGT	3	10	Intergenic
1	96753	96789	GTT/TTG/TGT	3	12.3	Intergenic
1	944628	944661	GTT/TTG/TGT	3	11.3	Intergenic
1	328861	328894	GTT/TTG/TGT	3	11.7	Intergenic
1	153736	153767	GTT/TTG/TGT	3	10.7	Intergenic
1	795228	795253	GCT/CTG/TGC	3	8.7	Intergenic
1	173099	173156	GCT/CTG/TGC	3	19.3	Intergenic
1	949419	949447	GGT/GTG/TGG	3	10	Intergenic
1	1084135	1084166	GGT/GTG/TGG	3	10.7	Intergenic
1	1247085	1247127	GGT/GTG/TGG	3	14.3	Intergenic
1	798091	798128	GGT/GTG/TGG	3	12.7	Intergenic
1	172339	172382	GGT/GTG/TGG	3	14.7	Intergenic
1	25572	25601	GGT/GTG/TGG	3	10	Intergenic
1	58404	58483	GGT/GTG/TGG	3	9.7	Intergenic
1	136185	136216	A	1	32	Intron

Chromosome	Range -start	Range-end	Unit sequence	Length	Repeat number	Type
1	33473	33503	A	1	31	Intron
1	36597	36628	A	1	32	Intron
1	40188	40213	A	1	26	Intron
1	817302	817332	A	1	31	Intron
1	155620	155644	A	1	25	Intron
1	108001	108038	A	1	38	Intron
1	541692	541763	A	1	72	Intron
1	1355980	1356004	A	1	25	Intron
1	352174	352212	A	1	39	Intron
1	686140	686171	A	1	32	Intron
1	730667	730694	A	1	28	Intron
1	71673	71731	A	1	59	Intron
1	96051	96080	A	1	30	Intron
1	128232	128277	A	1	46	Intron
1	94348	94388	C	1	41	Intron
1	252365	252390	C	1	26	Intron
1	84872	84896	C	1	25	Intron
1	232691	232723	C	1	33	Intron
1	886609	886636	G	1	28	Intron
1	159776	159818	T	1	43	Intron
1	66750	66808	T	1	59	Intron
1	174312	174350	T	1	39	Intron
1	232244	232302	T	1	59	Intron
1	107550	107612	T	1	63	Intron
1	897491	897519	T	1	29	Intron
1	1105822	1105851	T	1	30	Intron
1	131271	131327	T	1	57	Intron
1	168810	168856	T	1	47	Intron
1	105613	105653	T	1	41	Intron
1	38549	38599	T	1	51	Intron
1	1433394	1433419	T	1	26	Intron
1	451686	451714	T	1	29	Intron
1	667793	667844	T	1	52	Intron
1	8664	8703	T	1	40	Intron
1	181996	182037	T	1	42	Intron
1	559910	559946	CT/TC	2	19.5	Intron
1	887030	887127	CT/TC	2	48.5	Intron
1	117232	117259	CT/TC	2	14	Intron
1	61294	61318	GT/TG	2	12.5	Intron
1	700491	700535	ACC/CCA/CAC	3	15	Intron
1	774596	774620	AGC/GCA/CAG	3	8.3	Intron
1	214107	214154	ATG/TGA/GAT	3	16	Intron
1	685272	685297	ATG/TGA/GAT	3	8.7	Intron
1	98865	98901	CTT/TTC/TCT	3	12.3	Intron
1	1591147	1591183	CTT/TTC/TCT	3	12.3	Intron
1	13946	13980	GTT/TTG/TGT	3	11.7	Intron
1	57084	7108	GTT/TTG/TGT	3	8.3	Intron
1	798368	798400	GCT/CTG/TGC	3	11	Intron
2	374666	374698	A	1	33	Exon
2	557761	557791	AAC/ACA/CAA	3	10.3	Exon
2	647877	647960	AAC/ACA/CAA	3	28	Exon
2	869759	869826	AAC/ACA/CAA	3	22	Exon
2	245670	245723	AAC/ACA/CAA	3	18.3	Exon
2	361034	361272	AAC/ACA/CAA	3	79.7	Exon
2	224236	224278	AAC/ACA/CAA	3	14.3	Exon
2	245461	245493	AAC/ACA/CAA	3	11	Exon
2	720520	720544	AAC/ACA/CAA	3	8.3	Exon
2	884955	884984	AAC/ACA/CAA	3	10	Exon
2	39259	39348	AAC/ACA/CAA	3	30	Exon
2	210836	210872	AAC/ACA/CAA	3	12.3	Exon
2	11428	11452	AAC/ACA/CAA	3	8.3	Exon
2	40027	40055	AAC/ACA/CAA	3	9.7	Exon
2	19225	19250	AAC/ACA/CAA	3	8.7	Exon
2	58965	59025	AAC/ACA/CAA	3	20.3	Exon
2	255009	255067	AAG/AGA/GAA	3	19.7	Exon
2	702594	702688	AAG/AGA/GAA	3	31.7	Exon
2	142503	142530	AAG/AGA/GAA	3	9.3	Exon
2	151225	151253	AAG/AGA/GAA	3	9.7	Exon
2	174837	174862	ACC/CCA/CAC	3	8.7	Exon
2	96152	96186	ACC/CCA/CAC	3	11.7	Exon
2	274590	274624	ACC/CCA/CAC	3	11.7	Exon
2	647542	647585	AGC/GCA/CAG	3	14.7	Exon
2	837994	838019	AGC/GCA/CAG	3	8.7	Exon
2	7576	7606	AGC/GCA/CAG	3	10.3	Exon
2	175701	175793	AGC/GCA/CAG	3	31	Exon
2	609846	609877	AGC/GCA/CAG	3	10.7	Exon
2	637830	637856	AGC/GCA/CAG	3	9	Exon
2	91276	91304	AGC/GCA/CAG	3	9.7	Exon
2	94387	94415	AGC/GCA/CAG	3	9.7	Exon
2	663522	663548	AGG/GGA/GAG	3	9	Exon
2	154663	154688	AGG/GGA/GAG	3	8.7	Exon
2	743678	743710	AGG/GGA/GAG	3	11	Exon
2	810081	810115	AGG/GGA/GAG	3	11.7	Exon
2	67349	67379	ATG/TGA/GAT	3	10.3	Exon
2	246093	246139	ATG/TGA/GAT	3	15.7	Exon
2	142303	142334	ATG/TGA/GAT	3	10.7	Exon
2	443637	443732	ATG/TGA/GAT	3	32	Exon
2	999676	999795	ATG/TGA/GAT	3	9.7	Exon
2	215310	215339	CCT/CTC/TCC	3	10	Exon
2	552296	552320	CCT/CTC/TCC	3	8.3	Exon
2	10757	10805	CCT/CTC/TCC	3	16.3	Exon
2	77851	77879	CCT/CTC/TCC	3	9.7	Exon
2	145016	145053	CCT/CTC/TCC	3	12.7	Exon
2	171553	171641	CCT/CTC/TCC	3	29.7	Exon
2	57451	57481	CCT/CTC/TCC	3	10.3	Exon
2	129354	129411	CTT/TTC/TCT	3	19.7	Exon
2	68331	68363	CTT/TTC/TCT	3	11	Exon
2	523288	523331	CTT/TTC/TCT	3	14.7	Exon
2	87535	87571	CTT/TTC/TCT	3	12.3	Exon
2	338305	338550	GTT/TTG/TGT	3	82	Exon
2	822697	822726	GTT/TTG/TGT	3	10	Exon
2	823170	823209	GTT/TTG/TGT	3	13.3	Exon
2	153807	153851	GTT/TTG/TGT	3	15	Exon
2	255769	255808	GTT/TTG/TGT	3	13.3	Exon
2	352128	352172	GTT/TTG/TGT	3	15	Exon
2	133546	133579	GTT/TTG/TGT	3	11.3	Exon
2	300208	300253	GTT/TTG/TGT	3	15.3	Exon
2	69683	69708	GTT/TTG/TGT	3	8.7	Exon
2	116121	116161	GTT/TTG/TGT	3	13.7	Exon
2	294576	294616	GTT/TTG/TGT	3	13.7	Exon
2	61452	61491	GTT/TTG/TGT	3	13.3	Exon
2	248631	248717	GCT/CTG/TGC	3	29	Exon
2	76771	76795	GCT/CTG/TGC	3	8.3	Exon
2	132494	132520	GCT/CTG/TGC	3	9	Exon
2	274390	274417	GCT/CTG/TGC	3	9.3	Exon
2	71580	71610	GCT/CTG/TGC	3	10.3	Exon
2	574134	574161	GCT/CTG/TGC	3	9.3	Exon
2	37967	37993	GCT/CTG/TGC	3	9	Exon
2	34616	34642	GCT/CTG/TGC	3	9	Exon
2	81262	81287	GCT/CTG/TGC	3	8.7	Exon
2	103578	103602	GCT/CTG/TGC	3	8.3	Exon
2	145859	145892	GCT/CTG/TGC	3	11.3	Exon
2	148874	148953	GCT/CTG/TGC	3	26.7	Exon
2	115666	115690	GCT/CTG/TGC	3	8.3	Exon
2	151690	151714	GCT/CTG/TGC	3	8.3	Exon
2	294121	294145	GCT/CTG/TGC	3	8.3	Exon
2	169720	169754	GGT/GTG/TGG	3	11.7	Exon
2	170024	170076	GGT/GTG/TGG	3	17.7	Exon
2	928640	928669	GGT/GTG/TGG	3	10	Exon
2	225775	225814	GGT/GTG/TGG	3	13.3	Exon
2	142533	142587	GGT/GTG/TGG	3	8.3	Exon
2	207690	207729	GGT/GTG/TGG	3	13.3	Exon
2	45608	45703	GGT/GTG/TGG	3	31	Exon
2	64155	64181	A	1	27	Intergenic
2	234573	234597	A	1	25	Intergenic
2	313520	313562	A	1	43	Intergenic
2	344591	344648	A	1	58	Intergenic
2	452245	452269	A	1	25	Intergenic
2	458791	458831	A	1	41	Intergenic
2	495554	495605	A	1	52	Intergenic
2	547619	547660	A	1	42	Intergenic
2	582659	582687	A	1	29	Intergenic
2	704042	704086	A	1	45	Intergenic
2	735838	735869	A	1	32	Intergenic

Chromosome	Range -start	Range-end	Unit sequence	Length	Repeat number	Type
2	938054	938095	A	1	42	Intergenic
2	52191	52224	A	1	34	Intergenic
2	55668	55696	A	1	29	Intergenic
2	66189	66214	A	1	26	Intergenic
2	104180	104206	A	1	27	Intergenic
2	108783	108823	A	1	41	Intergenic
2	279244	279297	A	1	54	Intergenic
2	55619	55645	A	1	27	Intergenic
2	285020	285052	A	1	33	Intergenic
2	352649	352676	A	1	28	Intergenic
2	35068	35105	A	1	38	Intergenic
2	262698	262731	A	1	34	Intergenic
2	456961	457020	A	1	60	Intergenic
2	471342	471378	A	1	37	Intergenic
2	664569	664596	A	1	28	Intergenic
2	862708	862777	A	1	70	Intergenic
2	887808	887834	A	1	27	Intergenic
2	901204	901248	A	1	45	Intergenic
2	1026913	1026964	A	1	52	Intergenic
2	1065450	1065498	A	1	49	Intergenic
2	1096687	1096715	A	1	29	Intergenic
2	105870	105910	A	1	41	Intergenic
2	146079	146107	A	1	29	Intergenic
2	194517	194550	A	1	34	Intergenic
2	226851	226947	A	1	97	Intergenic
2	7440	7465	A	1	26	Intergenic
2	856932	856962	C	1	31	Intergenic
2	35168	35196	C	1	29	Intergenic
2	1026989	1027016	C	1	28	Intergenic
2	156867	156896	C	1	30	Intergenic
2	282723	282752	C	1	30	Intergenic
2	197557	197582	G	1	26	Intergenic
2	570530	570556	G	1	27	Intergenic
2	643067	643092	G	1	26	Intergenic
2	1025638	1025662	G	1	25	Intergenic
2	109413	109444	T	1	32	Intergenic
2	211167	211200	T	1	34	Intergenic
2	318699	318738	T	1	40	Intergenic
2	344891	344926	T	1	36	Intergenic
2	355897	355929	T	1	33	Intergenic
2	364433	364466	T	1	34	Intergenic
2	366330	366378	T	1	49	Intergenic
2	378444	378471	T	1	28	Intergenic
2	466507	466545	T	1	39	Intergenic
2	475298	475340	T	1	43	Intergenic
2	504069	504095	T	1	27	Intergenic
2	537000	537041	T	1	42	Intergenic
2	590675	590703	T	1	29	Intergenic
2	665072	665105	T	1	34	Intergenic
2	703586	703614	T	1	29	Intergenic
2	760156	760183	T	1	28	Intergenic
2	765135	765199	T	1	65	Intergenic
2	818493	818524	T	1	32	Intergenic
2	848652	848680	T	1	29	Intergenic
2	58108	58133	T	1	26	Intergenic
2	71046	71077	T	1	32	Intergenic
2	100354	100391	T	1	38	Intergenic
2	313932	313962	T	1	31	Intergenic
2	350510	350569	T	1	60	Intergenic
2	67117	67147	T	1	31	Intergenic
2	113285	113346	T	1	62	Intergenic
2	294605	294632	T	1	28	Intergenic
2	319178	319225	T	1	48	Intergenic
2	373769	373817	T	1	49	Intergenic
2	443107	443131	T	1	25	Intergenic
2	453158	453206	T	1	49	Intergenic
2	635179	635234	T	1	56	Intergenic
2	654409	654449	T	1	41	Intergenic
2	686826	686866	T	1	41	Intergenic
2	686973	687004	T	1	32	Intergenic
2	695002	695033	T	1	32	Intergenic
2	1121017	1121069	T	1	53	Intergenic

Chromosome	Range -start	Range-end	Unit sequence	Length	Repeat number	Type
2	92369	92397	T	1	29	Intergenic
2	160425	160458	T	1	34	Intergenic
2	183336	183384	T	1	49	Intergenic
2	143914	143939	T	1	26	Intergenic
2	178805	178862	T	1	58	Intergenic
2	184039	184078	T	1	40	Intergenic
2	43007	43049	T	1	43	Intergenic
2	77853	77886	T	1	34	Intergenic
2	189730	189776	T	1	47	Intergenic
2	221913	221941	T	1	29	Intergenic
2	256291	256324	T	1	34	Intergenic
2	59620	59655	T	1	36	Intergenic
2	259126	259160	AC/CA	2	17.5	Intergenic
2	373420	373469	AC/CA	2	25	Intergenic
2	914661	914699	AC/CA	2	19.5	Intergenic
2	29307	29352	AC/CA	2	23	Intergenic
2	3523	3549	AC/CA	2	13.5	Intergenic
2	125528	125562	AC/CA	2	17.5	Intergenic
2	344934	344967	AG/GA	2	17	Intergenic
2	34772	34815	AG/GA	2	22	Intergenic
2	928760	928813	AG/GA	2	27	Intergenic
2	71018	71060	AG/GA	2	21.5	Intergenic
2	249456	249498	AG/GA	2	21.5	Intergenic
2	636053	636122	CT/TC	2	35	Intergenic
2	884971	885017	CT/TC	2	23.5	Intergenic
2	44746	44779	CT/TC	2	17	Intergenic
2	341828	341953	CT/TC	2	63	Intergenic
2	866247	866290	CT/TC	2	22.5	Intergenic
2	53293	53328	CT/TC	2	18.5	Intergenic
2	442026	442067	GT/TG	2	21	Intergenic
2	499499	499532	GT/TG	2	17	Intergenic
2	501252	501279	GT/TG	2	14	Intergenic
2	144761	144787	GT/TG	2	13.5	Intergenic
2	25169	25198	GT/TG	2	15.5	Intergenic
2	382773	382805	GT/TG	2	16.5	Intergenic
2	526384	526460	GT/TG	2	36.5	Intergenic
2	504708	504826	AAC/ACA/CAA	3	12.3	Intergenic
2	809205	809242	AAC/ACA/CAA	3	12.7	Intergenic
2	904656	904692	AAC/ACA/CAA	3	12	Intergenic
2	53923	53950	AAG/AGA/GAA	3	9.3	Intergenic
2	494726	494759	AAG/AGA/GAA	3	11.3	Intergenic
2	886610	886635	AAG/AGA/GAA	3	8.7	Intergenic
2	90491	9516	AAG/AGA/GAA	3	8.7	Intergenic
2	365475	365534	ACC/CCA/CAC	3	19.7	Intergenic
2	20726	20752	ACC/CCA/CAC	3	9	Intergenic
2	214828	214872	AGC/GCA/CAG	3	15	Intergenic
2	304688	304712	AGC/GCA/CAG	3	8.3	Intergenic
2	252792	252818	AGG/GGA/GAG	3	9	Intergenic
2	397435	397466	AGG/GGA/GAG	3	10.7	Intergenic
2	464073	464108	AGG/GGA/GAG	3	12	Intergenic
2	126659	126685	AGG/GGA/GAG	3	9	Intergenic
2	186178	186208	AGG/GGA/GAG	3	10.3	Intergenic
2	212930	212964	AGG/GGA/GAG	3	11.7	Intergenic
2	127414	127446	AGG/GGA/GAG	3	11	Intergenic
2	355325	355351	ATG/TGA/GAT	3	9	Intergenic
2	225493	225533	ATG/TGA/GAT	3	13.7	Intergenic
2	37294	37321	CCT/CTC/TCC	3	9.3	Intergenic
2	71235	71267	CCT/CTC/TCC	3	11	Intergenic
2	21984	22009	CCT/CTC/TCC	3	8.7	Intergenic
2	20774	20861	CCT/CTC/TCC	3	12.7	Intergenic
2	494969	495005	CTT/TTC/TCT	3	12.3	Intergenic
2	704193	704253	CTT/TTC/TCT	3	20.3	Intergenic
2	299762	299809	CTT/TTC/TCT	3	16	Intergenic
2	19657	9707	GTT/TTG/TGT	3	17	Intergenic
2	25051	25083	GTT/TTG/TGT	3	11	Intergenic
2	510428	510462	GTT/TTG/TGT	3	11.7	Intergenic
2	590238	590281	GTT/TTG/TGT	3	14.7	Intergenic
2	603116	603164	GTT/TTG/TGT	3	15	Intergenic
2	799958	799993	GTT/TTG/TGT	3	12	Intergenic
2	889789	889815	GTT/TTG/TGT	3	9	Intergenic
2	499393	499423	GCT/CTG/TGC	3	10.3	Intergenic
2	700106	700137	GCT/CTG/TGC	3	10.7	Intergenic

Chromosome	Range -start	Range-end	Unit sequence	Length	Repeat number	Type
2	301848	301880	GCT/CTG/TGC	3	11	Intergenic
2	424445	424483	GCT/CTG/TGC	3	13	Intergenic
2	723347	723377	GCT/CTG/TGC	3	10.3	Intergenic
2	76980	77010	GCT/CTG/TGC	3	10.3	Intergenic
2	98995	99022	GGT/GTG/TGG	3	9.3	Intergenic
2	512812	512847	GGT/GTG/TGG	3	12	Intergenic
2	844761	844837	GGT/GTG/TGG	3	25.7	Intergenic
2	209562	209761	GGT/GTG/TGG	3	15.7	Intergenic
2	67345	67384	GGT/GTG/TGG	3	13.3	Intergenic
2	28540	28578	GGT/GTG/TGG	3	13	Intergenic
2	253577	253614	A	1	38	Intron
2	438702	438728	A	1	27	Intron
2	705913	705961	A	1	49	Intron
2	114780	114828	A	1	49	Intron
2	268651	268689	A	1	39	Intron
2	620887	620942	A	1	56	Intron
2	683900	683924	A	1	25	Intron
2	721391	721419	A	1	29	Intron
2	729936	729996	A	1	61	Intron
2	41782	41821	A	1	40	Intron
2	99967	99997	A	1	31	Intron
2	138	173	A	1	36	Intron
2	151901	151933	A	1	33	Intron
2	212422	212446	A	1	25	Intron
2	155352	155378	G	1	27	Intron
2	511182	511206	T	1	25	Intron
2	84241	84310	T	1	70	Intron
2	376049	376087	T	1	39	Intron
2	407528	407575	T	1	48	Intron
2	985931	985971	T	1	41	Intron
2	13042	13068	T	1	27	Intron
2	131999	132026	T	1	28	Intron
2	459498	459523	AC/CA	2	13	Intron
2	874855	874899	AC/CA	2	22.5	Intron
2	69352	69404	AC/CA	2	26.5	Intron
2	875259	875285	CT/TC	2	13.5	Intron
2	171819	171857	GT/TG	2	19.5	Intron
2	35590	35621	CCT/CTC/TCC	3	10.7	Intron
2	833653	833728	GTT/TTG/TGT	3	25.3	Intron
2	732697	732731	GCT/CTG/TGC	3	11.7	Intron
3	134771	134807	AAC/ACA/CAA	3	12.3	Exon
3	246781	246972	AAC/ACA/CAA	3	64	Exon
3	139770	139807	AAC/ACA/CAA	3	12.7	Exon
3	43839	43863	AAC/ACA/CAA	3	8.3	Exon
3	107106	107168	AAC/ACA/CAA	3	21.3	Exon
3	391113	391158	AAC/ACA/CAA	3	15.3	Exon
3	109231	109259	AAC/ACA/CAA	3	9.7	Exon
3	471054	471142	AAC/ACA/CAA	3	20.7	Exon
3	956374	956488	AAC/ACA/CAA	3	38.3	Exon
3	1135176	1135226	AAC/ACA/CAA	3	17	Exon
3	459551	459627	AAC/ACA/CAA	3	11.7	Exon
3	460452	460485	AAC/ACA/CAA	3	11.3	Exon
3	555204	555409	AAC/ACA/CAA	3	57.7	Exon
3	43810	43875	AAG/AGA/GAA	3	22	Exon
3	83662	83807	AAG/AGA/GAA	3	48.7	Exon
3	34391	34416	AAG/AGA/GAA	3	8.7	Exon
3	692695	692726	AAG/AGA/GAA	3	10.7	Exon
3	908823	908904	AAG/AGA/GAA	3	27	Exon
3	1211852	1211877	AAG/AGA/GAA	3	8.7	Exon
3	77201	77289	AAG/AGA/GAA	3	10	Exon
3	368832	368868	ACC/CCA/CAC	3	12.3	Exon
3	108072	108096	ACC/CCA/CAC	3	8.3	Exon
3	459509	459552	ACC/CCA/CAC	3	14.7	Exon
3	555171	555205	ACC/CCA/CAC	3	11.7	Exon
3	33978	34002	AGC/GCA/CAG	3	8.3	Exon
3	169082	169155	AGC/GCA/CAG	3	24.7	Exon
3	317630	317669	AGC/GCA/CAG	3	13.3	Exon
3	614830	614855	AGC/GCA/CAG	3	8.7	Exon
3	1135740	1135829	AGC/GCA/CAG	3	30	Exon
3	1442820	1442859	AGC/GCA/CAG	3	13	Exon
3	106026	106053	AGC/GCA/CAG	3	9.3	Exon
3	106078	106175	AGC/GCA/CAG	3	15.3	Exon
3	265539	265570	AGG/GGA/GAG	3	10.7	Exon
3	1873	2017	AGG/GGA/GAG	3	48.3	Exon
3	144496	144520	AGG/GGA/GAG	3	8.3	Exon
3	290521	290561	AGG/GGA/GAG	3	13.7	Exon
3	76427	76453	AGG/GGA/GAG	3	9	Exon
3	513370	513395	AGG/GGA/GAG	3	8.7	Exon
3	533943	533972	AGG/GGA/GAG	3	10	Exon
3	908903	909014	ATG/TGA/GAT	3	10.7	Exon
3	14620	14673	CCT/CTC/TCC	3	18.3	Exon
3	221811	221875	CCT/CTC/TCC	3	21.7	Exon
3	24715	24742	CCT/CTC/TCC	3	9.3	Exon
3	105758	105957	CTT/TTC/TCT	3	66.7	Exon
3	106370	106402	CTT/TTC/TCT	3	11	Exon
3	269119	269157	GTT/TTG/TGT	3	13	Exon
3	1018147	1018206	GTT/TTG/TGT	3	20	Exon
3	1334561	1334664	GTT/TTG/TGT	3	34.7	Exon
3	1521350	1521474	GTT/TTG/TGT	3	41.7	Exon
3	77142	77167	GTT/TTG/TGT	3	8.7	Exon
3	122079	122115	GCT/CTG/TGC	3	12.3	Exon
3	112389	112425	GCT/CTG/TGC	3	12.7	Exon
3	437246	437273	GCT/CTG/TGC	3	9.3	Exon
3	270856	270881	GGT/GTG/TGG	3	8.7	Exon
3	1278051	1278090	GGT/GTG/TGG	3	13.3	Exon
3	460817	460844	GGT/GTG/TGG	3	9.3	Exon
3	548767	548797	GGT/GTG/TGG	3	10.3	Exon
3	115156	115180	GGT/GTG/TGG	3	8.3	Exon
3	11811	11839	A	1	29	Intergenic
3	46562	46615	A	1	54	Intergenic
3	61319	61345	A	1	27	Intergenic
3	108512	108539	A	1	28	Intergenic
3	196818	196860	A	1	43	Intergenic
3	289745	289783	A	1	39	Intergenic
3	82761	82809	A	1	49	Intergenic
3	75952	75998	A	1	47	Intergenic
3	134930	134957	A	1	28	Intergenic
3	158808	158875	A	1	68	Intergenic
3	238055	238103	A	1	49	Intergenic
3	326786	326811	A	1	26	Intergenic
3	412238	412264	A	1	27	Intergenic
3	36012	36046	A	1	35	Intergenic
3	292551	292576	A	1	26	Intergenic
3	431508	431544	A	1	37	Intergenic
3	52124	52184	A	1	61	Intergenic
3	78571	78595	A	1	25	Intergenic
3	160185	160232	A	1	48	Intergenic
3	189877	189907	A	1	31	Intergenic
3	199333	199366	A	1	34	Intergenic
3	244246	244279	A	1	34	Intergenic
3	292483	292517	A	1	35	Intergenic
3	465644	465691	A	1	48	Intergenic
3	484431	484475	A	1	45	Intergenic
3	612130	612156	A	1	27	Intergenic
3	837637	837678	A	1	42	Intergenic
3	856582	856620	A	1	39	Intergenic
3	993796	993826	A	1	31	Intergenic
3	1014035	1014068	A	1	34	Intergenic
3	1161514	1161561	A	1	48	Intergenic
3	1241458	1241494	A	1	37	Intergenic
3	1459347	1459382	A	1	36	Intergenic
3	1575500	1575538	A	1	39	Intergenic
3	1759008	1759032	A	1	25	Intergenic
3	1764338	1764379	A	1	42	Intergenic
3	1827409	1827455	A	1	47	Intergenic
3	137561	137588	A	1	28	Intergenic
3	65281	65311	A	1	31	Intergenic
3	85342	85422	A	1	81	Intergenic
3	181503	181550	A	1	48	Intergenic
3	18513	18567	A	1	55	Intergenic
3	128694	128733	A	1	40	Intergenic
3	552587	552630	A	1	44	Intergenic
3	419944	419968	C	1	25	Intergenic
3	422986	423014	C	1	29	Intergenic

Chromosome	Range -start	Range-end	Unit sequence	Length	Repeat number	Type
3	19897	19927	C	1	31	Intergenic
3	16103	16127	G	1	25	Intergenic
3	23957	23984	G	1	28	Intergenic
3	292159	292186	G	1	28	Intergenic
3	62978	63003	G	1	26	Intergenic
3	64242	64325	T	1	84	Intergenic
3	157574	157617	T	1	44	Intergenic
3	23904	23938	T	1	35	Intergenic
3	41974	42004	T	1	31	Intergenic
3	209026	209052	T	1	27	Intergenic
3	239774	239802	T	1	29	Intergenic
3	89984	90020	T	1	37	Intergenic
3	93717	93741	T	1	25	Intergenic
3	291892	291939	T	1	48	Intergenic
3	131567	131602	T	1	36	Intergenic
3	407440	407498	T	1	59	Intergenic
3	25580	25637	T	1	58	Intergenic
3	115835	115886	T	1	52	Intergenic
3	235002	235041	T	1	40	Intergenic
3	386013	386049	T	1	37	Intergenic
3	390008	390035	T	1	28	Intergenic
3	78839	78881	T	1	43	Intergenic
3	119284	119321	T	1	38	Intergenic
3	159581	159622	T	1	42	Intergenic
3	174629	174656	T	1	28	Intergenic
3	469792	469818	T	1	27	Intergenic
3	488295	488332	T	1	38	Intergenic
3	524103	524148	T	1	46	Intergenic
3	537082	537133	T	1	52	Intergenic
3	542593	542625	T	1	33	Intergenic
3	571266	571296	T	1	31	Intergenic
3	731993	732043	T	1	51	Intergenic
3	1012970	1013011	T	1	42	Intergenic
3	1039624	1039664	T	1	41	Intergenic
3	1077592	1077630	T	1	39	Intergenic
3	1109416	1109445	T	1	30	Intergenic
3	1143874	1143917	T	1	44	Intergenic
3	1250195	1250222	T	1	28	Intergenic
3	1411821	1411872	T	1	52	Intergenic
3	1495476	1495514	T	1	39	Intergenic
3	1651121	1651160	T	1	40	Intergenic
3	1809376	1809411	T	1	36	Intergenic
3	29196	29237	T	1	42	Intergenic
3	119413	119484	T	1	72	Intergenic
3	22047	22079	T	1	33	Intergenic
3	487955	488070	T	1	116	Intergenic
3	149084	149121	AC/CA	2	19	Intergenic
3	157485	157512	AC/CA	2	14	Intergenic
3	898184	898214	AC/CA	2	15.5	Intergenic
3	1160765	1160816	AC/CA	2	26	Intergenic
3	89631	89664	AC/CA	2	17	Intergenic
3	208643	208668	AG/GA	2	13	Intergenic
3	120653	120684	AG/GA	2	16	Intergenic
3	127419	127453	AG/GA	2	17.5	Intergenic
3	85056	85085	CT/TC	2	15	Intergenic
3	135857	135895	CT/TC	2	19.5	Intergenic
3	181308	181337	CT/TC	2	15	Intergenic
3	18453	18549	CT/TC	2	48.5	Intergenic
3	199625	199674	CT/TC	2	26.5	Intergenic
3	105699	105724	CT/TC	2	13	Intergenic
3	856001	856027	GT/TG	2	13.5	Intergenic
3	999095	999163	GT/TG	2	34.5	Intergenic
3	1453937	1453980	GT/TG	2	22	Intergenic
3	238013	238056	AAC/ACA/CAA	3	14.7	Intergenic
3	210503	210540	AAC/ACA/CAA	3	12.7	Intergenic
3	213152	213177	AAC/ACA/CAA	3	8.7	Intergenic
3	998567	998606	AAC/ACA/CAA	3	13.3	Intergenic
3	550042	550096	AAG/AGA/GAA	3	18	Intergenic
3	659083	659111	AAG/AGA/GAA	3	9.7	Intergenic
3	743138	743185	AAG/AGA/GAA	3	16	Intergenic
3	140267	140301	AAG/AGA/GAA	3	11.7	Intergenic
3	6864	6890	ACC/CCA/CAC	3	9	Intergenic

Chromosome	Range -start	Range-end	Unit sequence	Length	Repeat number	Type
3	214888	214915	ACC/CCA/CAC	3	9.3	Intergenic
3	288957	288982	ACC/CCA/CAC	3	8.7	Intergenic
3	34984	35019	AGC/GCA/CAG	3	12	Intergenic
3	34569	34598	AGC/GCA/CAG	3	10	Intergenic
3	95594	95620	AGC/GCA/CAG	3	9	Intergenic
3	289671	289709	AGC/GCA/CAG	3	13	Intergenic
3	634584	634609	AGC/GCA/CAG	3	8.7	Intergenic
3	730845	730888	AGC/GCA/CAG	3	14.7	Intergenic
3	1440829	1440858	AGC/GCA/CAG	3	10	Intergenic
3	400773	400817	ATG/TGA/GAT	3	15	Intergenic
3	449075	449099	ATG/TGA/GAT	3	8.3	Intergenic
3	1387768	1387792	ATG/TGA/GAT	3	8.3	Intergenic
3	1452588	1452625	ATG/TGA/GAT	3	12.7	Intergenic
3	38313	38360	ATG/TGA/GAT	3	16	Intergenic
3	321145	321174	ATG/TGA/GAT	3	10	Intergenic
3	582191	582216	ATG/TGA/GAT	3	8.7	Intergenic
3	1686727	1686769	CCT/CTC/TCC	3	14	Intergenic
3	320804	320837	CCT/CTC/TCC	3	11.3	Intergenic
3	331147	331176	CCT/CTC/TCC	3	10	Intergenic
3	135511	135546	CTT/TTC/TCT	3	12	Intergenic
3	135965	135991	CTT/TTC/TCT	3	9	Intergenic
3	121599	121632	CTT/TTC/TCT	3	11.3	Intergenic
3	25541	25581	CTT/TTC/TCT	3	13.7	Intergenic
3	1384234	1384261	CTT/TTC/TCT	3	9.3	Intergenic
3	33881	33922	GTT/TTG/TGT	3	13.7	Intergenic
3	380491	380528	GTT/TTG/TGT	3	12.7	Intergenic
3	743372	743397	GTT/TTG/TGT	3	8.7	Intergenic
3	939113	939144	GTT/TTG/TGT	3	10.7	Intergenic
3	1101594	1101638	GTT/TTG/TGT	3	15	Intergenic
3	94702	4732	GTT/TTG/TGT	3	10.3	Intergenic
3	112341	112638	GTT/TTG/TGT	3	99.3	Intergenic
3	420473	420508	GCT/CTG/TGC	3	12	Intergenic
3	1008983	1009010	GCT/CTG/TGC	3	9.3	Intergenic
3	126487	126514	GGT/GTG/TGG	3	9.3	Intergenic
3	121034	121059	GGT/GTG/TGG	3	8.7	Intergenic
3	185673	185698	A	1	26	Intron
3	201904	201943	A	1	40	Intron
3	132228	132255	A	1	28	Intron
3	54635	54662	A	1	28	Intron
3	323090	323134	A	1	45	Intron
3	1090975	1091026	A	1	52	Intron
3	1100942	1100993	A	1	52	Intron
3	1499478	1499514	A	1	37	Intron
3	265223	265252	G	1	30	Intron
3	25230	25258	T	1	29	Intron
3	199360	199404	T	1	45	Intron
3	475687	475724	T	1	38	Intron
3	1084607	1084651	T	1	45	Intron
3	12420	12474	T	1	55	Intron
3	13897	13929	T	1	33	Intron
3	218684	218749	AG/GA	2	33	Intron
3	631979	632013	AG/GA	2	17.5	Intron
3	124387	124417	CT/TC	2	15.5	Intron
3	161323	161396	CT/TC	2	37.5	Intron
3	416564	416589	CT/TC	2	13	Intron
3	282363	282398	GT/TG	2	18	Intron
3	1009966	1010002	AAC/ACA/CAA	3	12.3	Intron
3	55403	55448	AAG/AGA/GAA	3	15.3	Intron
3	4055	4090	ACC/CCA/CAC	3	12	Intron
3	133085	133126	AGC/GCA/CAG	3	14.3	Intron
3	290958	291021	ATG/TGA/GAT	3	21	Intron
3	127994	128036	CCT/CTC/TCC	3	14.3	Intron
3	317268	317292	CCT/CTC/TCC	3	8.3	Intron
3	317291	317373	CTT/TTC/TCT	3	20.7	Intron
4	434579	434626	GT/TG	2	24	Exon
4	202513	202549	AAC/ACA/CAA	3	12.3	Exon
4	138179	138224	AAC/ACA/CAA	3	14.7	Exon
4	83125	83154	AAC/ACA/CAA	3	10	Exon
4	106714	106751	AAC/ACA/CAA	3	12.7	Exon
4	173992	174041	AAC/ACA/CAA	3	16.7	Exon
4	90931	90969	AAC/ACA/CAA	3	13	Exon
4	36909	36951	AAC/ACA/CAA	3	14.3	Exon

Chromosome	Range -start	Range-end	Unit sequence	Length	Repeat number	Type
4	439820	439856	AAC/ACA/CAA	3	12.3	Exon
4	19205	19356	AAG/AGA/GAA	3	50.7	Exon
4	206169	206236	AAG/AGA/GAA	3	22.7	Exon
4	240606	240631	AAG/AGA/GAA	3	8.7	Exon
4	621911	621950	AAG/AGA/GAA	3	13.3	Exon
4	73051	73093	AAG/AGA/GAA	3	14	Exon
4	286486	286624	AAG/AGA/GAA	3	20	Exon
4	228991	229016	AAG/AGA/GAA	3	8.7	Exon
4	442304	442376	AAG/AGA/GAA	3	24.3	Exon
4	46198	46280	AAG/AGA/GAA	3	27.7	Exon
4	18622	18651	ACC/CCA/CAC	3	10	Exon
4	547543	547569	ACC/CCA/CAC	3	9	Exon
4	580638	580678	ACC/CCA/CAC	3	13.7	Exon
4	8606	8632	AGC/GCA/CAG	3	9	Exon
4	48260	48365	AGC/GCA/CAG	3	35.3	Exon
4	183299	183332	AGC/GCA/CAG	3	11.3	Exon
4	55559	55585	AGC/GCA/CAG	3	9	Exon
4	160116	160157	AGC/GCA/CAG	3	14	Exon
4	161813	161848	AGC/GCA/CAG	3	12	Exon
4	237869	237904	AGC/GCA/CAG	3	12	Exon
4	346981	347019	AGC/GCA/CAG	3	13	Exon
4	590521	590552	AGC/GCA/CAG	3	10.7	Exon
4	620975	620999	AGC/GCA/CAG	3	8.3	Exon
4	106750	106814	AGC/GCA/CAG	3	9.7	Exon
4	272232	272257	AGC/GCA/CAG	3	8.7	Exon
4	153494	153525	AGC/GCA/CAG	3	10.7	Exon
4	56547	56571	AGG/GGA/GAG	3	8.3	Exon
4	248960	249034	AGG/GGA/GAG	3	21.3	Exon
4	340105	340154	AGG/GGA/GAG	3	16.7	Exon
4	129044	129077	AGG/GGA/GAG	3	11.3	Exon
4	24527	24554	AGG/GGA/GAG	3	9.3	Exon
4	260119	260171	AGG/GGA/GAG	3	17.7	Exon
4	281597	281657	ATG/TGA/GAT	3	20.3	Exon
4	169119	169154	ATG/TGA/GAT	3	12	Exon
4	28588	28708	CCT/CTC/TCC	3	40.3	Exon
4	141787	141849	CCT/CTC/TCC	3	21	Exon
4	49920	49957	CCT/CTC/TCC	3	12.7	Exon
4	139486	139517	CCT/CTC/TCC	3	10.7	Exon
4	474799	474823	CCT/CTC/TCC	3	8.3	Exon
4	72580	72611	CCT/CTC/TCC	3	10.7	Exon
4	14413	14445	CTT/TTC/TCT	3	11	Exon
4	20439	20477	GTT/TTG/TGT	3	13	Exon
4	24948	4986	GTT/TTG/TGT	3	13	Exon
4	347280	347432	GTT/TTG/TGT	3	51	Exon
4	380053	380079	GTT/TTG/TGT	3	9	Exon
4	138649	138692	GTT/TTG/TGT	3	14.7	Exon
4	321120	321154	GTT/TTG/TGT	3	11.7	Exon
4	562907	562968	GTT/TTG/TGT	3	20.7	Exon
4	640169	640200	GTT/TTG/TGT	3	10.7	Exon
4	340772	340807	GTT/TTG/TGT	3	12	Exon
4	150408	150436	GTT/TTG/TGT	3	9.7	Exon
4	153747	153780	GTT/TTG/TGT	3	11.3	Exon
4	51309	51363	GTT/TTG/TGT	3	18.3	Exon
4	84026	84060	GCT/CTG/TGC	3	11.7	Exon
4	120584	120751	GCT/CTG/TGC	3	56	Exon
4	407379	407414	GCT/CTG/TGC	3	12	Exon
4	128399	128428	GCT/CTG/TGC	3	10	Exon
4	221029	221073	GCT/CTG/TGC	3	15	Exon
4	562967	563058	GCT/CTG/TGC	3	10.7	Exon
4	249649	249678	GCT/CTG/TGC	3	10	Exon
4	431509	431544	GCT/CTG/TGC	3	12	Exon
4	290348	290391	GGT/GTG/TGG	3	14.7	Exon
4	481576	481610	GGT/GTG/TGG	3	11.7	Exon
4	149789	149816	GGT/GTG/TGG	3	9.3	Exon
4	191700	191727	GGT/GTG/TGG	3	9.3	Exon
4	197739	197766	GGT/GTG/TGG	3	9.3	Exon
4	14144	14175	GGT/GTG/TGG	3	10.7	Exon
4	6753	6779	A	1	27	Intergenic
4	166467	166539	A	1	73	Intergenic
4	198562	198587	A	1	26	Intergenic
4	44416	44448	A	1	33	Intergenic
4	181582	181617	A	1	36	Intergenic
4	209032	209087	A	1	56	Intergenic
4	400529	400556	A	1	28	Intergenic
4	622006	622034	A	1	29	Intergenic
4	189310	189338	A	1	29	Intergenic
4	2945	2980	A	1	36	Intergenic
4	111715	111756	A	1	42	Intergenic
4	257620	257657	A	1	38	Intergenic
4	162900	162924	A	1	25	Intergenic
4	232081	232121	A	1	41	Intergenic
4	96789	96830	A	1	42	Intergenic
4	97270	97296	A	1	27	Intergenic
4	189811	189839	A	1	29	Intergenic
4	217708	217756	A	1	49	Intergenic
4	302054	302090	A	1	37	Intergenic
4	9242	9270	A	1	29	Intergenic
4	38670	38704	A	1	35	Intergenic
4	119162	119199	A	1	38	Intergenic
4	329423	329458	A	1	36	Intergenic
4	430644	430695	A	1	52	Intergenic
4	148891	148942	A	1	52	Intergenic
4	25384	25439	A	1	56	Intergenic
4	50569	50610	A	1	42	Intergenic
4	118250	118282	A	1	33	Intergenic
4	58995	59043	A	1	49	Intergenic
4	134302	134341	A	1	40	Intergenic
4	193005	193034	A	1	30	Intergenic
4	219968	220016	A	1	49	Intergenic
4	224469	224507	A	1	39	Intergenic
4	241186	241228	A	1	43	Intergenic
4	280271	280311	A	1	41	Intergenic
4	285910	285950	A	1	41	Intergenic
4	49313	49338	A	1	26	Intergenic
4	53045	53084	A	1	40	Intergenic
4	79545	79585	A	1	41	Intergenic
4	88577	88602	A	1	26	Intergenic
4	276718	276747	A	1	30	Intergenic
4	312659	312683	A	1	25	Intergenic
4	349226	349268	A	1	43	Intergenic
4	349979	350022	A	1	44	Intergenic
4	447350	447386	A	1	37	Intergenic
4	540420	540453	A	1	34	Intergenic
4	614811	614853	A	1	43	Intergenic
4	649865	649889	A	1	25	Intergenic
4	258949	258982	C	1	34	Intergenic
4	104333	104360	C	1	28	Intergenic
4	154752	154782	G	1	31	Intergenic
4	169057	169084	G	1	28	Intergenic
4	234730	234755	G	1	26	Intergenic
4	220784	220811	G	1	28	Intergenic
4	360405	360433	G	1	29	Intergenic
4	4422	4457	T	1	36	Intergenic
4	58601	58629	T	1	29	Intergenic
4	41880	41923	T	1	44	Intergenic
4	160344	160431	T	1	88	Intergenic
4	256928	256966	T	1	39	Intergenic
4	30917	30957	T	1	41	Intergenic
4	210465	210490	T	1	26	Intergenic
4	293103	293145	T	1	43	Intergenic
4	318881	318925	T	1	45	Intergenic
4	319849	319903	T	1	55	Intergenic
4	358289	358322	T	1	34	Intergenic
4	404665	404708	T	1	44	Intergenic
4	519612	519642	T	1	31	Intergenic
4	519794	519832	T	1	39	Intergenic
4	597187	597234	T	1	48	Intergenic
4	597386	597410	T	1	25	Intergenic
4	649734	649779	T	1	46	Intergenic
4	653665	653702	T	1	38	Intergenic
4	4043	490	T	1	48	Intergenic
4	19723	19759	T	1	37	Intergenic
4	244097	244126	T	1	30	Intergenic
4	269915	269950	T	1	36	Intergenic

Chromosome	Range -start	Range-end	Unit sequence	Length	Repeat number	Type
4	27904	27954	T	1	51	Intergenic
4	30786	30823	T	1	38	Intergenic
4	46237	46278	T	1	42	Intergenic
4	194349	194383	T	1	35	Intergenic
4	209631	209656	T	1	26	Intergenic
4	24558	24599	T	1	42	Intergenic
4	116941	116983	T	1	43	Intergenic
4	186389	186447	T	1	59	Intergenic
4	202281	202310	T	1	30	Intergenic
4	157220	157250	T	1	31	Intergenic
4	159237	159285	T	1	49	Intergenic
4	56102	56134	T	1	33	Intergenic
4	169995	170057	T	1	63	Intergenic
4	402136	402187	T	1	52	Intergenic
4	236598	236639	T	1	42	Intergenic
4	248459	248486	T	1	28	Intergenic
4	354311	354338	T	1	28	Intergenic
4	443664	443706	T	1	43	Intergenic
4	529139	529173	T	1	35	Intergenic
4	140153	140206	T	1	54	Intergenic
4	227992	228030	T	1	39	Intergenic
4	231441	231526	T	1	86	Intergenic
4	243780	243813	T	1	34	Intergenic
4	181706	181731	T	1	26	Intergenic
4	221561	221592	T	1	32	Intergenic
4	66192	66219	T	1	28	Intergenic
4	210625	210654	T	1	30	Intergenic
4	441265	441299	T	1	35	Intergenic
4	517665	517690	T	1	26	Intergenic
4	538617	538646	T	1	30	Intergenic
4	544431	544477	T	1	47	Intergenic
4	566896	566940	T	1	45	Intergenic
4	614504	614542	T	1	39	Intergenic
4	633106	633142	T	1	37	Intergenic
4	657822	657877	T	1	56	Intergenic
4	667136	667197	T	1	62	Intergenic
4	106230	106293	AC/CA	2	32	Intergenic
4	349891	349915	AC/CA	2	12.5	Intergenic
4	140079	140104	AG/GA	2	13	Intergenic
4	34807	34837	AG/GA	2	15.5	Intergenic
4	365114	365138	AG/GA	2	12.5	Intergenic
4	139320	139345	CT/TC	2	13	Intergenic
4	7686	7717	CT/TC	2	17	Intergenic
4	542079	542116	CT/TC	2	19	Intergenic
4	69492	69529	GT/TG	2	19	Intergenic
4	5274	5310	GT/TG	2	18.5	Intergenic
4	175061	175128	GT/TG	2	34	Intergenic
4	514711	514745	GT/TG	2	17.5	Intergenic
4	139557	139587	GT/TG	2	15.5	Intergenic
4	193443	193697	GT/TG	2	127.5	Intergenic
4	224393	224431	GT/TG	2	19.5	Intergenic
4	323447	323477	GT/TG	2	15.5	Intergenic
4	375261	375301	GT/TG	2	21.5	Intergenic
4	60095	60140	AAC/ACA/CAA	3	15.3	Intergenic
4	31954	31984	AAC/ACA/CAA	3	10.3	Intergenic
4	13846	13895	AAC/ACA/CAA	3	16.7	Intergenic
4	154612	154640	AAC/ACA/CAA	3	9.7	Intergenic
4	270621	270662	AAC/ACA/CAA	3	14	Intergenic
4	280244	280268	AAG/AGA/GAA	3	8.3	Intergenic
4	224852	224891	AAG/AGA/GAA	3	13.3	Intergenic
4	473619	473657	AAG/AGA/GAA	3	13	Intergenic
4	252722	252750	ACC/CCA/CAC	3	9.7	Intergenic
4	244842	244866	ACC/CCA/CAC	3	8.3	Intergenic
4	276969	277010	ACC/CCA/CAC	3	14	Intergenic
4	376420	376446	ACC/CCA/CAC	3	9	Intergenic
4	381840	381878	ACC/CCA/CAC	3	13	Intergenic
4	431326	431355	ACC/CCA/CAC	3	10	Intergenic
4	42239	42273	AGC/GCA/CAG	3	11.7	Intergenic
4	251198	251240	AGC/GCA/CAG	3	14.3	Intergenic
4	648625	648662	AGG/GGA/GAG	3	12.3	Intergenic
4	557878	557902	ATG/TGA/GAT	3	8.3	Intergenic
4	526500	526525	ATG/TGA/GAT	3	8.7	Intergenic

Chromosome	Range -start	Range-end	Unit sequence	Length	Repeat number	Type
4	129541	129575	CCT/CTC/TCC	3	11.7	Intergenic
4	205969	205997	CCT/CTC/TCC	3	9.7	Intergenic
4	424233	424267	CCT/CTC/TCC	3	11.7	Intergenic
4	63216	63248	CTT/TTC/TCT	3	11.3	Intergenic
4	617949	617973	CTT/TTC/TCT	3	8.3	Intergenic
4	360248	360389	CTT/TTC/TCT	3	47.7	Intergenic
4	169249	169285	CTT/TTC/TCT	3	12.3	Intergenic
4	138789	138822	CTT/TTC/TCT	3	11.3	Intergenic
4	373105	373190	CTT/TTC/TCT	3	26.7	Intergenic
4	88530	8564	GTT/TTG/TGT	3	11.7	Intergenic
4	393240	393275	GTT/TTG/TGT	3	11.7	Intergenic
4	195828	195856	GTT/TTG/TGT	3	9.7	Intergenic
4	171173	171211	GTT/TTG/TGT	3	13	Intergenic
4	74760	74788	GTT/TTG/TGT	3	9.7	Intergenic
4	505127	505208	GTT/TTG/TGT	3	27.3	Intergenic
4	294293	294334	GTT/TTG/TGT	3	14.3	Intergenic
4	355497	355528	GTT/TTG/TGT	3	10.7	Intergenic
4	86122	86156	GCT/CTG/TGC	3	11.7	Intergenic
4	198245	198275	GCT/CTG/TGC	3	10.3	Intergenic
4	145323	145349	GGT/GTG/TGG	3	9	Intergenic
4	91924	91952	GGT/GTG/TGG	3	9.7	Intergenic
4	252789	252831	GGT/GTG/TGG	3	14	Intergenic
4	253645	253677	GGT/GTG/TGG	3	11	Intergenic
4	499078	499114	GGT/GTG/TGG	3	12.3	Intergenic
4	502817	502843	GGT/GTG/TGG	3	9	Intergenic
4	495040	495066	GGT/GTG/TGG	3	9	Intergenic
4	404742	404769	GGT/GTG/TGG	3	9.3	Intergenic
4	180673	180711	GGT/GTG/TGG	3	13	Intergenic
4	340008	340032	GGT/GTG/TGG	3	8.3	Intergenic
4	196218	196257	A	1	40	Intron
4	262356	262384	A	1	29	Intron
4	279121	279148	A	1	28	Intron
4	407061	407094	A	1	34	Intron
4	86734	86798	A	1	65	Intron
4	146642	146684	A	1	43	Intron
4	218413	218446	A	1	34	Intron
4	249104	249152	A	1	49	Intron
4	218349	218389	A	1	41	Intron
4	76444	76468	A	1	25	Intron
4	170419	170444	A	1	26	Intron
4	262000	262024	C	1	25	Intron
4	202752	202780	G	1	29	Intron
4	650451	650476	T	1	26	Intron
4	171873	171899	T	1	27	Intron
4	168435	168477	T	1	43	Intron
4	235986	236015	T	1	30	Intron
4	245356	245410	T	1	55	Intron
4	166174	166208	T	1	35	Intron
4	300137	300177	T	1	41	Intron
4	432225	432273	T	1	49	Intron
4	29004	29041	CT/TC	2	19	Intron
4	42487	42518	CT/TC	2	16	Intron
4	222855	222905	GT/TG	2	26	Intron
4	610906	610936	GT/TG	2	15.5	Intron
4	204217	204261	AAC/ACA/CAA	3	14.7	Intron
4	168177	168202	AAC/ACA/CAA	3	8.7	Intron
4	30277	30319	AAC/ACA/CAA	3	14	Intron
4	132631	132663	AGG/GGA/GAG	3	11	Intron
4	483775	483800	ATG/TGA/GAT	3	8.7	Intron
4	18689	18713	GGT/GTG/TGG	3	8.3	Intron
5	339761	339806	T	1	46	Exon
5	377985	378014	CT/TC	2	15	Exon
5	68378	68469	AAC/ACA/CAA	3	30.7	Exon
5	399363	399405	AAC/ACA/CAA	3	14.3	Exon
5	809902	809931	AAC/ACA/CAA	3	10	Exon
5	835809	835843	AAC/ACA/CAA	3	11.7	Exon
5	295990	296217	AAC/ACA/CAA	3	76.7	Exon
5	269174	269234	AAC/ACA/CAA	3	20.3	Exon
5	730224	730452	AAC/ACA/CAA	3	76.3	Exon
5	92791	92820	AAC/ACA/CAA	3	10	Exon
5	298184	298235	AAC/ACA/CAA	3	17.3	Exon
5	229996	230091	AAC/ACA/CAA	3	32	Exon

Chromosome	Range -start	Range-end	Unit sequence	Length	Repeat number	Type
5	342183	342233	AAC/ACA/CAA	3	17	Exon
5	645842	645916	AAC/ACA/CAA	3	25	Exon
5	27309	27352	AAC/ACA/CAA	3	14.7	Exon
5	109113	109180	AAC/ACA/CAA	3	22.7	Exon
5	159975	160001	AAC/ACA/CAA	3	9	Exon
5	76782	76882	AAG/AGA/GAA	3	33.7	Exon
5	717160	717196	AAG/AGA/GAA	3	12.3	Exon
5	15688	15732	AAG/AGA/GAA	3	15	Exon
5	244414	244450	ACC/CCA/CAC	3	12.3	Exon
5	288834	288860	ACC/CCA/CAC	3	9	Exon
5	845555	845592	ACC/CCA/CAC	3	12.3	Exon
5	99312	99350	ACC/CCA/CAC	3	13	Exon
5	111380	111409	ACC/CCA/CAC	3	10	Exon
5	68468	68625	AGC/GCA/CAG	3	22.7	Exon
5	292193	292230	AGC/GCA/CAG	3	12.7	Exon
5	20814	2839	AGC/GCA/CAG	3	8.7	Exon
5	75351	75385	AGC/GCA/CAG	3	11.7	Exon
5	264615	264639	AGC/GCA/CAG	3	8.3	Exon
5	51968	51992	AGC/GCA/CAG	3	8.3	Exon
5	863126	863157	AGC/GCA/CAG	3	10.7	Exon
5	45746	45790	AGC/GCA/CAG	3	15	Exon
5	223562	223611	AGC/GCA/CAG	3	16.7	Exon
5	126057	126111	AGG/GGA/GAG	3	18.3	Exon
5	76922	76960	AGG/GGA/GAG	3	13	Exon
5	659198	659246	AGG/GGA/GAG	3	16.3	Exon
5	806397	806428	AGG/GGA/GAG	3	10.7	Exon
5	731790	731824	AGG/GGA/GAG	3	11.7	Exon
5	743488	743516	AGG/GGA/GAG	3	9.7	Exon
5	1784	1952	AGG/GGA/GAG	3	56.3	Exon
5	178027	178065	AGG/GGA/GAG	3	13	Exon
5	738623	738654	AGG/GGA/GAG	3	10.7	Exon
5	42755	42798	ATG/TGA/GAT	3	14.7	Exon
5	308987	309013	ATG/TGA/GAT	3	9	Exon
5	15731	15823	ATG/TGA/GAT	3	16.7	Exon
5	550855	550884	CCT/CTC/TCC	3	10	Exon
5	764835	764878	CCT/CTC/TCC	3	14.7	Exon
5	506271	506354	CCT/CTC/TCC	3	28	Exon
5	147430	147467	CCT/CTC/TCC	3	12.7	Exon
5	269249	269391	CCT/CTC/TCC	3	22.7	Exon
5	689349	689381	CCT/CTC/TCC	3	11.3	Exon
5	215478	215509	CCT/CTC/TCC	3	10.7	Exon
5	262433	262469	CCT/CTC/TCC	3	12.3	Exon
5	65695	65723	CTT/TTC/TCT	3	9.7	Exon
5	10801	10828	CTT/TTC/TCT	3	9.3	Exon
5	141260	141328	CTT/TTC/TCT	3	23.3	Exon
5	696659	696684	CTT/TTC/TCT	3	8.7	Exon
5	235340	235375	CTT/TTC/TCT	3	12	Exon
5	361740	361779	CTT/TTC/TCT	3	13.3	Exon
5	367928	367959	GTT/TTG/TGT	3	10.7	Exon
5	892843	892877	GTT/TTG/TGT	3	11.7	Exon
5	892929	892980	GTT/TTG/TGT	3	17.3	Exon
5	260377	260414	GTT/TTG/TGT	3	12.7	Exon
5	406380	406411	GTT/TTG/TGT	3	10.7	Exon
5	442602	442626	GTT/TTG/TGT	3	8.3	Exon
5	112232	112263	GTT/TTG/TGT	3	10.7	Exon
5	267442	267470	GTT/TTG/TGT	3	9.7	Exon
5	7522	7613	GTT/TTG/TGT	3	31	Exon
5	128466	128667	GTT/TTG/TGT	3	57	Exon
5	143021	143141	GTT/TTG/TGT	3	40.3	Exon
5	20912	20947	GCT/CTG/TGC	3	12	Exon
5	76634	76666	GCT/CTG/TGC	3	11	Exon
5	134003	134032	GCT/CTG/TGC	3	10	Exon
5	202700	202726	GCT/CTG/TGC	3	9	Exon
5	272420	272452	GCT/CTG/TGC	3	11	Exon
5	330252	330293	GCT/CTG/TGC	3	14	Exon
5	364627	364652	GCT/CTG/TGC	3	8.7	Exon
5	396500	396537	GCT/CTG/TGC	3	12.7	Exon
5	562847	562878	GCT/CTG/TGC	3	10.7	Exon
5	690095	690119	GCT/CTG/TGC	3	8.3	Exon
5	449098	449139	GCT/CTG/TGC	3	14	Exon
5	106732	106764	GCT/CTG/TGC	3	11	Exon
5	538939	538972	GCT/CTG/TGC	3	11.3	Exon

Chromosome	Range -start	Range-end	Unit sequence	Length	Repeat number	Type
5	848805	848833	GCT/CTG/TGC	3	9.7	Exon
5	310784	310809	GCT/CTG/TGC	3	8.7	Exon
5	789786	789813	GGT/GTG/TGG	3	9.3	Exon
5	99789	99818	GGT/GTG/TGG	3	10	Exon
5	128435	128467	GGT/GTG/TGG	3	11	Exon
5	37981	38014	GGT/GTG/TGG	3	11.3	Exon
5	93096	93123	GGT/GTG/TGG	3	9.3	Exon
5	1460	1524	A	1	65	Intergenic
5	19132	19181	A	1	50	Intergenic
5	27963	28021	A	1	59	Intergenic
5	36803	36846	A	1	44	Intergenic
5	45628	45691	A	1	64	Intergenic
5	4137	4194	A	1	58	Intergenic
5	12976	13040	A	1	65	Intergenic
5	15483	15507	A	1	25	Intergenic
5	37016	37066	A	1	51	Intergenic
5	172711	172748	A	1	38	Intergenic
5	29382	29422	A	1	41	Intergenic
5	67081	67139	A	1	59	Intergenic
5	169345	169369	A	1	25	Intergenic
5	64879	64950	A	1	72	Intergenic
5	25736	25765	A	1	30	Intergenic
5	38135	38176	A	1	42	Intergenic
5	55212	55240	A	1	29	Intergenic
5	135187	135256	A	1	70	Intergenic
5	47361	47405	A	1	45	Intergenic
5	346282	346308	A	1	27	Intergenic
5	416867	416898	A	1	32	Intergenic
5	521910	521962	A	1	53	Intergenic
5	802493	802518	A	1	26	Intergenic
5	40658	40682	A	1	25	Intergenic
5	188042	188081	A	1	40	Intergenic
5	211251	211296	A	1	46	Intergenic
5	214975	215009	A	1	35	Intergenic
5	551246	551286	A	1	41	Intergenic
5	768530	768564	A	1	35	Intergenic
5	781107	781150	A	1	44	Intergenic
5	338241	338283	A	1	43	Intergenic
5	376807	376834	A	1	28	Intergenic
5	79895	79933	A	1	39	Intergenic
5	180194	180227	A	1	34	Intergenic
5	194435	194468	A	1	34	Intergenic
5	200131	200165	A	1	35	Intergenic
5	295640	295671	A	1	32	Intergenic
5	24159	24190	A	1	32	Intergenic
5	159800	159825	A	1	26	Intergenic
5	440145	440179	A	1	35	Intergenic
5	454878	454915	A	1	38	Intergenic
5	538786	538874	A	1	89	Intergenic
5	560314	560350	A	1	37	Intergenic
5	619861	619918	A	1	58	Intergenic
5	680696	680729	A	1	34	Intergenic
5	847916	847977	A	1	62	Intergenic
5	67492	67518	A	1	27	Intergenic
5	132880	132912	A	1	33	Intergenic
5	248822	248863	A	1	42	Intergenic
5	2780	2809	A	1	30	Intergenic
5	312680	312706	C	1	27	Intergenic
5	494487	494512	C	1	26	Intergenic
5	805782	805807	C	1	26	Intergenic
5	290173	290202	G	1	30	Intergenic
5	381561	381585	G	1	25	Intergenic
5	432108	432141	G	1	34	Intergenic
5	9103	9128	G	1	26	Intergenic
5	584034	584061	G	1	28	Intergenic
5	391853	391882	G	1	30	Intergenic
5	320393	320423	G	1	31	Intergenic
5	29671	29715	T	1	45	Intergenic
5	177247	177301	T	1	55	Intergenic
5	184496	184521	T	1	26	Intergenic
5	212793	212841	T	1	49	Intergenic
5	62579	62621	T	1	43	Intergenic

Chromosome	Range -start	Range-end	Unit sequence	Length	Repeat number	Type
5	97763	97801	T	1	39	Intergenic
5	251601	251626	T	1	26	Intergenic
5	328072	328106	T	1	35	Intergenic
5	347467	347496	T	1	30	Intergenic
5	414544	414568	T	1	25	Intergenic
5	432079	432107	T	1	29	Intergenic
5	466937	466984	T	1	48	Intergenic
5	87520	87561	T	1	42	Intergenic
5	155849	155882	T	1	34	Intergenic
5	166438	166493	T	1	56	Intergenic
5	172115	172155	T	1	41	Intergenic
5	226338	226378	T	1	41	Intergenic
5	319454	319504	T	1	51	Intergenic
5	468332	468359	T	1	28	Intergenic
5	498519	498561	T	1	43	Intergenic
5	772731	772801	T	1	71	Intergenic
5	107012	107037	T	1	26	Intergenic
5	216414	216467	T	1	54	Intergenic
5	373429	373468	T	1	40	Intergenic
5	479947	479981	T	1	35	Intergenic
5	165411	165459	T	1	49	Intergenic
5	170561	170585	T	1	25	Intergenic
5	217345	217393	T	1	49	Intergenic
5	386861	386897	T	1	37	Intergenic
5	442120	442147	T	1	28	Intergenic
5	676326	676361	T	1	36	Intergenic
5	677868	677896	T	1	29	Intergenic
5	683341	683376	T	1	36	Intergenic
5	41871	41924	T	1	54	Intergenic
5	51898	51935	T	1	38	Intergenic
5	57885	57921	T	1	37	Intergenic
5	232044	232078	T	1	35	Intergenic
5	242376	242410	T	1	35	Intergenic
5	306202	306246	T	1	45	Intergenic
5	88132	88177	T	1	46	Intergenic
5	225188	225231	T	1	44	Intergenic
5	157915	157952	T	1	38	Intergenic
5	214027	214069	T	1	43	Intergenic
5	325920	325962	T	1	43	Intergenic
5	8485	8546	T	1	62	Intergenic
5	75282	75329	T	1	48	Intergenic
5	140767	140792	T	1	26	Intergenic
5	144458	144493	T	1	36	Intergenic
5	156873	156899	T	1	27	Intergenic
5	20455	20484	T	1	30	Intergenic
5	127846	127890	T	1	45	Intergenic
5	70723	70777	AC/CA	2	27.5	Intergenic
5	97056	97082	AC/CA	2	13.5	Intergenic
5	160124	160173	AC/CA	2	26	Intergenic
5	225000	225029	AC/CA	2	15	Intergenic
5	275448	275544	AC/CA	2	48.5	Intergenic
5	487055	487079	AC/CA	2	12.5	Intergenic
5	55240	55282	AG/GA	2	21.5	Intergenic
5	55395	55427	AG/GA	2	16.5	Intergenic
5	96586	96690	AG/GA	2	52.5	Intergenic
5	885209	885275	AG/GA	2	33.5	Intergenic
5	338321	338353	AG/GA	2	16.5	Intergenic
5	564948	565014	AG/GA	2	33.5	Intergenic
5	243656	243682	AG/GA	2	13.5	Intergenic
5	164269	164298	AG/GA	2	15	Intergenic
5	292980	293034	AG/GA	2	27.5	Intergenic
5	198419	198484	CT/TC	2	33	Intergenic
5	54440	54478	CT/TC	2	19.5	Intergenic
5	493210	493238	CT/TC	2	14.5	Intergenic
5	38425	38455	CT/TC	2	15.5	Intergenic
5	294067	294104	CT/TC	2	18	Intergenic
5	125521	125677	CT/TC	2	78.5	Intergenic
5	8603	8704	GT/TG	2	51	Intergenic
5	270727	270765	GT/TG	2	19.5	Intergenic
5	250043	250068	GT/TG	2	13	Intergenic
5	721666	721753	GT/TG	2	44	Intergenic
5	658348	658379	GT/TG	2	16	Intergenic

Chromosome	Range -start	Range-end	Unit sequence	Length	Repeat number	Type
5	828101	828131	GT/TG	2	15.5	Intergenic
5	115653	115704	AAC/ACA/CAA	3	17.3	Intergenic
5	132777	132812	AAC/ACA/CAA	3	12	Intergenic
5	340199	340225	AAC/ACA/CAA	3	9	Intergenic
5	327016	327048	AAC/ACA/CAA	3	11	Intergenic
5	147165	147209	AAC/ACA/CAA	3	15	Intergenic
5	97787	97816	AAG/AGA/GAA	3	10.3	Intergenic
5	129693	129723	AAG/AGA/GAA	3	10.3	Intergenic
5	269597	269623	AAG/AGA/GAA	3	9	Intergenic
5	340003	340028	AAG/AGA/GAA	3	8.7	Intergenic
5	765292	765333	AAG/AGA/GAA	3	13.3	Intergenic
5	51247	51287	ACC/CCA/CAC	3	13.7	Intergenic
5	35197	35223	ACC/CCA/CAC	3	9	Intergenic
5	195260	195291	ACC/CCA/CAC	3	10.7	Intergenic
5	185750	185782	ACC/CCA/CAC	3	11	Intergenic
5	521169	521206	ACC/CCA/CAC	3	12.7	Intergenic
5	758598	758631	ACC/CCA/CAC	3	11.3	Intergenic
5	815497	815526	ACC/CCA/CAC	3	10	Intergenic
5	485994	486018	AGC/GCA/CAG	3	8.3	Intergenic
5	129722	129803	AGG/GGA/GAG	3	17.7	Intergenic
5	464017	464072	AGG/GGA/GAG	3	18.7	Intergenic
5	30949	30982	AGG/GGA/GAG	3	11.3	Intergenic
5	26548	26585	ATG/TGA/GAT	3	12.7	Intergenic
5	164030	164069	ATG/TGA/GAT	3	13.3	Intergenic
5	741593	741654	ATG/TGA/GAT	3	20.7	Intergenic
5	137431	137478	CCT/CTC/TCC	3	16.3	Intergenic
5	18206	18245	CCT/CTC/TCC	3	13.3	Intergenic
5	225789	225870	CTT/TTC/TCT	3	27.3	Intergenic
5	289919	289968	CTT/TTC/TCT	3	17	Intergenic
5	23727	23760	CTT/TTC/TCT	3	11	Intergenic
5	301632	301666	GTT/TTG/TGT	3	11	Intergenic
5	425087	425111	GTT/TTG/TGT	3	8.3	Intergenic
5	98557	8581	GTT/TTG/TGT	3	8.3	Intergenic
5	292643	292668	GCT/CTG/TGC	3	8.7	Intergenic
5	531135	531163	GCT/CTG/TGC	3	9.7	Intergenic
5	62915	62942	GCT/CTG/TGC	3	9.3	Intergenic
5	293188	293230	GGT/GTG/TGG	3	15	Intergenic
5	346550	346574	GGT/GTG/TGG	3	8.3	Intergenic
5	237823	237857	GGT/GTG/TGG	3	11.7	Intergenic
5	138163	138197	GGT/GTG/TGG	3	11.3	Intergenic
5	10306	10350	A	1	45	Intron
5	24759	24823	A	1	65	Intron
5	104610	104645	A	1	36	Intron
5	179201	179229	A	1	29	Intron
5	136509	136544	A	1	36	Intron
5	363697	363757	A	1	61	Intron
5	408568	408603	A	1	36	Intron
5	727057	727083	A	1	27	Intron
5	378699	378726	A	1	28	Intron
5	111993	112018	A	1	26	Intron
5	408699	408734	A	1	36	Intron
5	284320	284350	C	1	31	Intron
5	106504	106545	C	1	42	Intron
5	373639	373665	G	1	27	Intron
5	86035	86070	T	1	36	Intron
5	388280	388348	T	1	69	Intron
5	452970	453005	T	1	36	Intron
5	128803	128870	T	1	68	Intron
5	834180	834229	T	1	50	Intron
5	647172	647230	T	1	59	Intron
5	34101	34150	T	1	50	Intron
5	788232	788268	T	1	37	Intron
5	363209	363308	AC/CA	2	20	Intron
5	302181	302277	AAC/ACA/CAA	3	32.3	Intron
5	748233	748279	ACC/CCA/CAC	3	15.7	Intron
5	340747	340782	AGG/GGA/GAG	3	12	Intron
5	754579	754617	AGG/GGA/GAG	3	13	Intron
5	500331	500383	AGG/GGA/GAG	3	17.7	Intron
5	737566	737595	CTT/TTC/TCT	3	10	Intron
5	754616	754687	GGT/GTG/TGG	3	11.7	Intron
5	392741	392773	GGT/GTG/TGG	3	11	Intron
6	440302	440331	AAC/ACA/CAA	3	10	Exon

Chromosome	Range -start	Range-end	Unit sequence	Length	Repeat number	Type
6	10774	10799	AAC/ACA/CAA	3	8.7	Exon
6	39041	39092	AAC/ACA/CAA	3	17.3	Exon
6	404574	404606	AAC/ACA/CAA	3	11	Exon
6	619845	619896	AAC/ACA/CAA	3	17.3	Exon
6	748060	748091	AAC/ACA/CAA	3	10.7	Exon
6	54758	54783	AAG/AGA/GAA	3	8.7	Exon
6	374111	374148	AAG/AGA/GAA	3	12.7	Exon
6	484337	484398	AAG/AGA/GAA	3	20	Exon
6	795125	795174	AAG/AGA/GAA	3	16.7	Exon
6	920096	920180	AAG/AGA/GAA	3	23	Exon
6	36864	36913	ACC/CCA/CAC	3	16.3	Exon
6	46583	46616	AGC/GCA/CAG	3	11.3	Exon
6	507557	507594	AGC/GCA/CAG	3	12.7	Exon
6	285833	285860	AGC/GCA/CAG	3	9.3	Exon
6	354676	354709	AGC/GCA/CAG	3	11.3	Exon
6	634162	634199	AGC/GCA/CAG	3	12.7	Exon
6	425725	425753	AGG/GGA/GAG	3	9.7	Exon
6	842227	842264	AGG/GGA/GAG	3	12.7	Exon
6	263346	263376	ATG/TGA/GAT	3	10.3	Exon
6	555687	555736	CCT/CTC/TCC	3	16.7	Exon
6	181082	181117	CCT/CTC/TCC	3	12	Exon
6	503934	503959	CCT/CTC/TCC	3	8.7	Exon
6	216591	216634	CCT/CTC/TCC	3	14.7	Exon
6	955937	955962	CCT/CTC/TCC	3	8.7	Exon
6	962304	962329	CCT/CTC/TCC	3	8.7	Exon
6	425606	425630	CTT/TTC/TCT	3	8.3	Exon
6	118040	118065	CTT/TTC/TCT	3	8.7	Exon
6	828558	828646	CTT/TTC/TCT	3	29.7	Exon
6	331390	331414	GTT/TTG/TGT	3	8.3	Exon
6	464130	464189	GTT/TTG/TGT	3	20	Exon
6	524418	524454	GTT/TTG/TGT	3	12.3	Exon
6	487338	487393	GTT/TTG/TGT	3	18.7	Exon
6	518295	518321	GTT/TTG/TGT	3	9	Exon
6	484855	484890	GTT/TTG/TGT	3	12	Exon
6	490251	490280	GTT/TTG/TGT	3	10	Exon
6	595016	595060	GTT/TTG/TGT	3	15	Exon
6	996190	996239	GTT/TTG/TGT	3	16.7	Exon
6	996191	996216	GTT/TTG/TGT	3	8.3	Exon
6	518044	518073	GCT/CTG/TGC	3	10	Exon
6	525552	525578	GCT/CTG/TGC	3	9	Exon
6	382121	382147	GCT/CTG/TGC	3	9	Exon
6	205413	205445	GCT/CTG/TGC	3	11	Exon
6	230405	230441	GCT/CTG/TGC	3	12.3	Exon
6	347701	347731	GCT/CTG/TGC	3	10.3	Exon
6	412470	412501	GCT/CTG/TGC	3	10.7	Exon
6	385833	385870	GGT/GTG/TGG	3	12.7	Exon
6	347991	348043	GGT/GTG/TGG	3	17.7	Exon
6	29064	29094	GGT/GTG/TGG	3	10.3	Exon
6	46156	46181	GGT/GTG/TGG	3	8.7	Exon
6	50959	51059	GGT/GTG/TGG	3	32.7	Exon
6	56488	56535	A	1	48	Intergenic
6	67256	67290	A	1	35	Intergenic
6	95484	95527	A	1	44	Intergenic
6	343047	343072	A	1	26	Intergenic
6	367789	367819	A	1	31	Intergenic
6	368429	368458	A	1	30	Intergenic
6	583580	583606	A	1	27	Intergenic
6	88562	88595	A	1	34	Intergenic
6	486997	487041	A	1	45	Intergenic
6	189133	189175	A	1	43	Intergenic
6	201633	201662	A	1	30	Intergenic
6	218100	218129	A	1	30	Intergenic
6	266321	266388	A	1	68	Intergenic
6	376252	376362	A	1	111	Intergenic
6	32161	32210	A	1	50	Intergenic
6	257940	257980	A	1	41	Intergenic
6	291739	291771	A	1	33	Intergenic
6	297632	297671	A	1	40	Intergenic
6	343871	343911	A	1	41	Intergenic
6	456475	456533	A	1	59	Intergenic
6	672822	672904	A	1	83	Intergenic
6	822938	823002	A	1	65	Intergenic
6	891725	891754	A	1	30	Intergenic
6	899614	899674	A	1	61	Intergenic
6	954641	954680	A	1	40	Intergenic
6	966065	966089	A	1	25	Intergenic
6	973364	973409	A	1	46	Intergenic
6	1025137	1025183	A	1	47	Intergenic
6	1168072	1168113	A	1	42	Intergenic
6	361207	361236	C	1	30	Intergenic
6	118677	118702	C	1	26	Intergenic
6	331567	331594	C	1	28	Intergenic
6	138330	138366	G	1	37	Intergenic
6	389319	389346	G	1	28	Intergenic
6	967696	967720	G	1	25	Intergenic
6	12804	12829	T	1	26	Intergenic
6	165279	165337	T	1	59	Intergenic
6	341461	341492	T	1	32	Intergenic
6	125084	125133	T	1	50	Intergenic
6	135037	135088	T	1	52	Intergenic
6	314026	314056	T	1	31	Intergenic
6	445103	445155	T	1	53	Intergenic
6	458968	459011	T	1	44	Intergenic
6	521739	521764	T	1	26	Intergenic
6	541113	541137	T	1	25	Intergenic
6	582987	583042	T	1	56	Intergenic
6	587414	587445	T	1	32	Intergenic
6	631406	631440	T	1	35	Intergenic
6	36299	36335	T	1	37	Intergenic
6	140588	140654	T	1	67	Intergenic
6	247445	247491	T	1	47	Intergenic
6	248574	248612	T	1	39	Intergenic
6	287924	287962	T	1	39	Intergenic
6	406418	406452	T	1	35	Intergenic
6	407083	407108	T	1	26	Intergenic
6	408218	408250	T	1	33	Intergenic
6	510567	510625	T	1	59	Intergenic
6	59342	59374	T	1	33	Intergenic
6	332545	332584	T	1	40	Intergenic
6	393697	393743	T	1	47	Intergenic
6	417683	417711	T	1	29	Intergenic
6	465120	465145	T	1	26	Intergenic
6	548798	548829	T	1	32	Intergenic
6	182131	182166	T	1	36	Intergenic
6	195664	195705	T	1	42	Intergenic
6	276191	276230	T	1	40	Intergenic
6	310619	310672	T	1	54	Intergenic
6	341405	341435	T	1	31	Intergenic
6	471893	471960	T	1	68	Intergenic
6	643774	643822	T	1	49	Intergenic
6	771572	771599	T	1	28	Intergenic
6	789928	789973	T	1	46	Intergenic
6	1021373	1021411	T	1	39	Intergenic
6	1064660	1064698	T	1	39	Intergenic
6	1141158	1141195	T	1	38	Intergenic
6	147477	147507	AC/CA	2	15.5	Intergenic
6	147716	147751	AC/CA	2	18	Intergenic
6	313786	313810	AC/CA	2	12.5	Intergenic
6	23498	23563	AC/CA	2	33	Intergenic
6	394392	394418	AC/CA	2	13.5	Intergenic
6	307890	307925	AC/CA	2	18	Intergenic
6	95572	95620	AG/GA	2	24.5	Intergenic
6	322811	322835	CT/TC	2	12.5	Intergenic
6	79329	79382	CT/TC	2	27.5	Intergenic
6	112512	112536	GT/TG	2	12.5	Intergenic
6	739484	739520	GT/TG	2	18.5	Intergenic
6	596110	596175	GT/TG	2	33	Intergenic
6	501791	501828	AAC/ACA/CAA	3	12.7	Intergenic
6	59833	59907	AAG/AGA/GAA	3	26	Intergenic
6	111170	111208	AAG/AGA/GAA	3	13	Intergenic
6	372954	372987	AAG/AGA/GAA	3	11.3	Intergenic
6	210903	210961	AAG/AGA/GAA	3	19.7	Intergenic
6	218025	218098	AAG/AGA/GAA	3	25	Intergenic
6	258262	258331	AAG/AGA/GAA	3	23.3	Intergenic

Chromosome	Range -start	Range-end	Unit sequence	Length	Repeat number	Type
6	250817	250849	ACC/CCA/CAC	3	11	Intergenic
6	489102	489131	ACC/CCA/CAC	3	10	Intergenic
6	128145	128170	ACC/CCA/CAC	3	8.7	Intergenic
6	460371	460401	ACC/CCA/CAC	3	10.3	Intergenic
6	422896	422920	ACC/CCA/CAC	3	8.3	Intergenic
6	614597	614663	ACC/CCA/CAC	3	21.7	Intergenic
6	386583	386619	AGC/GCA/CAG	3	12.3	Intergenic
6	430119	430153	AGG/GGA/GAG	3	11.7	Intergenic
6	990653	990682	AGG/GGA/GAG	3	10	Intergenic
6	623700	623724	ATG/TGA/GAT	3	8.3	Intergenic
6	394792	394852	CCT/CTC/TCC	3	20.3	Intergenic
6	633706	633777	CCT/CTC/TCC	3	10	Intergenic
6	520203	520240	CCT/CTC/TCC	3	12.7	Intergenic
6	640023	640058	CTT/TTC/TCT	3	12	Intergenic
6	342402	342430	CTT/TTC/TCT	3	9.7	Intergenic
6	415757	415800	CTT/TTC/TCT	3	14.7	Intergenic
6	511893	511929	GTT/TTG/TGT	3	12	Intergenic
6	231198	231244	GCT/CTG/TGC	3	15.7	Intergenic
6	506243	506272	GCT/CTG/TGC	3	10	Intergenic
6	630776	630814	GCT/CTG/TGC	3	13	Intergenic
6	402399	402433	GGT/GTG/TGG	3	11.7	Intergenic
6	447268	447297	GGT/GTG/TGG	3	10	Intergenic
6	972152	972189	GGT/GTG/TGG	3	12.7	Intergenic
6	356718	356747	GGT/GTG/TGG	3	10	Intergenic
6	260655	260684	GGT/GTG/TGG	3	10	Intergenic
6	11755	11791	A	1	37	Intron
6	332055	332083	A	1	29	Intron
6	341052	341085	A	1	34	Intron
6	477713	477761	A	1	49	Intron
6	636833	636884	A	1	52	Intron
6	767962	767993	A	1	32	Intron
6	995901	995943	A	1	43	Intron
6	246163	246196	G	1	34	Intron
6	362830	362860	T	1	31	Intron
6	510768	510798	T	1	31	Intron
6	38851	38896	T	1	46	Intron
6	246597	246635	T	1	39	Intron
6	277532	277558	T	1	27	Intron
6	335514	335549	T	1	36	Intron
6	504297	504321	T	1	25	Intron
6	463909	463934	T	1	26	Intron
6	995728	995755	T	1	28	Intron
6	684536	684564	AG/GA	2	14.5	Intron
6	245477	245519	GT/TG	2	21.5	Intron
6	46102	46129	ACC/CCA/CAC	3	9.3	Intron
6	825653	825694	ACC/CCA/CAC	3	14	Intron
6	159017	159046	ACC/CCA/CAC	3	10	Intron
6	67324	67395	ATG/TGA/GAT	3	10	Intron
6	434261	434289	GCT/CTG/TGC	3	9.7	Intron
6	293442	293472	GGT/GTG/TGG	3	10.3	Intron
7	46844	46906	AAC/ACA/CAA	3	21	Exon
7	24819	24941	AAC/ACA/CAA	3	41	Exon
7	46689	46720	AAC/ACA/CAA	3	10.7	Exon
7	71475	71602	AAC/ACA/CAA	3	42.7	Exon
7	306223	306249	AAC/ACA/CAA	3	9	Exon
7	13668	13733	AAC/ACA/CAA	3	21.7	Exon
7	198589	198645	AAC/ACA/CAA	3	19	Exon
7	615080	615259	AAC/ACA/CAA	3	60	Exon
7	129139	129182	AAC/ACA/CAA	3	14.7	Exon
7	224725	224763	AAC/ACA/CAA	3	13	Exon
7	466667	466698	AAC/ACA/CAA	3	10.7	Exon
7	627808	627841	AAC/ACA/CAA	3	11.3	Exon
7	186099	186205	AAC/ACA/CAA	3	35.7	Exon
7	321165	321205	AAG/AGA/GAA	3	13.7	Exon
7	252450	252475	AAG/AGA/GAA	3	8.7	Exon
7	494272	494301	AAG/AGA/GAA	3	10	Exon
7	359208	359249	ACC/CCA/CAC	3	14	Exon
7	98019	98045	ACC/CCA/CAC	3	9	Exon
7	596920	596952	ACC/CCA/CAC	3	11	Exon
7	224145	224190	ACC/CCA/CAC	3	15.3	Exon
7	296700	296734	ACC/CCA/CAC	3	11.7	Exon
7	464973	465004	AGC/GCA/CAG	3	10.7	Exon
7	627412	627451	AGC/GCA/CAG	3	13.3	Exon
7	678491	678518	AGC/GCA/CAG	3	9.3	Exon
7	341443	341467	AGC/GCA/CAG	3	8.3	Exon
7	430962	431003	AGC/GCA/CAG	3	14	Exon
7	500246	500278	AGC/GCA/CAG	3	11	Exon
7	9575	9612	AGG/GGA/GAG	3	12.7	Exon
7	723333	723363	AGG/GGA/GAG	3	10.7	Exon
7	206812	206864	AGG/GGA/GAG	3	17.7	Exon
7	330070	330104	AGG/GGA/GAG	3	11.7	Exon
7	460139	460180	AGG/GGA/GAG	3	14	Exon
7	460352	460487	AGG/GGA/GAG	3	34.3	Exon
7	63346	63370	ATG/TGA/GAT	3	8.3	Exon
7	57835	57870	CCT/CTC/TCC	3	12	Exon
7	119482	119506	CCT/CTC/TCC	3	8.3	Exon
7	125572	125601	CCT/CTC/TCC	3	10.3	Exon
7	86506	86550	CCT/CTC/TCC	3	15	Exon
7	24674	24717	CCT/CTC/TCC	3	14.7	Exon
7	97220	97261	CCT/CTC/TCC	3	14	Exon
7	730780	730824	CCT/CTC/TCC	3	15	Exon
7	102167	102248	CCT/CTC/TCC	3	23.3	Exon
7	440124	440155	CCT/CTC/TCC	3	10.7	Exon
7	89514	89597	CTT/TTC/TCT	3	28.3	Exon
7	90821	90856	CTT/TTC/TCT	3	12	Exon
7	466939	466991	CTT/TTC/TCT	3	17.7	Exon
7	16214	6255	GTT/TTG/TGT	3	14	Exon
7	106583	106608	GTT/TTG/TGT	3	8.7	Exon
7	581435	581460	GTT/TTG/TGT	3	8.7	Exon
7	135238	135285	GTT/TTG/TGT	3	16	Exon
7	640100	640182	GTT/TTG/TGT	3	22.7	Exon
7	227086	227191	GTT/TTG/TGT	3	35.3	Exon
7	227420	227449	GTT/TTG/TGT	3	10	Exon
7	381317	381343	GTT/TTG/TGT	3	9	Exon
7	95382	95410	GCT/CTG/TGC	3	9.7	Exon
7	179772	179798	GCT/CTG/TGC	3	9	Exon
7	458820	458845	GCT/CTG/TGC	3	8.7	Exon
7	619458	619493	GCT/CTG/TGC	3	12.3	Exon
7	619779	619813	GCT/CTG/TGC	3	11.7	Exon
7	106328	106357	GGT/GTG/TGG	3	10	Exon
7	570316	570340	GGT/GTG/TGG	3	8.3	Exon
7	169091	169140	GGT/GTG/TGG	3	16.7	Exon
7	179815	179844	GGT/GTG/TGG	3	10	Exon
7	52248	52275	GGT/GTG/TGG	3	9.3	Exon
7	300022	300046	GGT/GTG/TGG	3	8.3	Exon
7	103836	103868	GGT/GTG/TGG	3	11	Exon
7	103361	103392	GGT/GTG/TGG	3	10.7	Exon
7	79179	79208	A	1	30	Intergenic
7	120193	120229	A	1	37	Intergenic
7	104282	104324	A	1	43	Intergenic
7	9688	9729	A	1	42	Intergenic
7	71797	71840	A	1	44	Intergenic
7	87629	87671	A	1	43	Intergenic
7	157872	157912	A	1	41	Intergenic
7	183215	183258	A	1	44	Intergenic
7	186570	186595	A	1	26	Intergenic
7	206959	207000	A	1	42	Intergenic
7	372109	372168	A	1	60	Intergenic
7	47135	47161	A	1	27	Intergenic
7	37450	37516	A	1	67	Intergenic
7	12883	12914	A	1	32	Intergenic
7	21704	21733	A	1	30	Intergenic
7	23672	23728	A	1	57	Intergenic
7	147670	147718	A	1	49	Intergenic
7	280820	280883	A	1	64	Intergenic
7	319323	319353	A	1	31	Intergenic
7	328850	328876	A	1	27	Intergenic
7	371459	371485	A	1	27	Intergenic
7	389789	389857	A	1	69	Intergenic
7	590227	590269	A	1	43	Intergenic
7	81387	81420	A	1	34	Intergenic
7	63264	63295	A	1	32	Intergenic
7	184084	184111	A	1	28	Intergenic
7	199320	199361	A	1	42	Intergenic

Chromosome	Range -start	Range-end	Unit sequence	Length	Repeat number	Type
7	209385	209418	A	1	34	Intergenic
7	252128	252158	A	1	31	Intergenic
7	356521	356557	A	1	37	Intergenic
7	432777	432807	A	1	31	Intergenic
7	635638	635669	A	1	32	Intergenic
7	873932	873972	A	1	41	Intergenic
7	924164	924202	A	1	39	Intergenic
7	89864	89926	A	1	63	Intergenic
7	92591	92617	A	1	27	Intergenic
7	204123	204166	A	1	44	Intergenic
7	211919	211949	A	1	31	Intergenic
7	200906	200944	A	1	39	Intergenic
7	268218	268242	A	1	25	Intergenic
7	351485	351517	A	1	33	Intergenic
7	408728	408766	A	1	39	Intergenic
7	448048	448088	A	1	41	Intergenic
7	515237	515276	A	1	40	Intergenic
7	64376	64403	C	1	28	Intergenic
7	142618	142645	C	1	28	Intergenic
7	28362	28389	G	1	28	Intergenic
7	209106	209136	G	1	31	Intergenic
7	31751	31805	T	1	55	Intergenic
7	51210	51243	T	1	34	Intergenic
7	125786	125819	T	1	34	Intergenic
7	104372	104474	T	1	103	Intergenic
7	275573	275603	T	1	31	Intergenic
7	12606	12656	T	1	51	Intergenic
7	41649	41680	T	1	32	Intergenic
7	55708	55761	T	1	54	Intergenic
7	305951	305985	T	1	35	Intergenic
7	342706	342753	T	1	48	Intergenic
7	476509	476549	T	1	41	Intergenic
7	98961	98987	T	1	27	Intergenic
7	80843	80884	T	1	42	Intergenic
7	116405	116440	T	1	36	Intergenic
7	198654	198681	T	1	28	Intergenic
7	283627	283654	T	1	28	Intergenic
7	311574	311622	T	1	49	Intergenic
7	407289	407359	T	1	71	Intergenic
7	481822	481853	T	1	32	Intergenic
7	537567	537609	T	1	43	Intergenic
7	823024	823083	T	1	60	Intergenic
7	861490	861538	T	1	49	Intergenic
7	888386	888457	T	1	72	Intergenic
7	910504	910539	T	1	36	Intergenic
7	98469	98518	T	1	50	Intergenic
7	166219	166252	T	1	34	Intergenic
7	98783	98814	T	1	32	Intergenic
7	27382	27429	T	1	48	Intergenic
7	372993	373023	T	1	31	Intergenic
7	417813	417839	T	1	27	Intergenic
7	453186	453223	T	1	38	Intergenic
7	178411	178445	AC/CA	2	17.5	Intergenic
7	161554	161598	AC/CA	2	22.5	Intergenic
7	1990913	199152	AC/CA	2	31	Intergenic
7	23294	23327	AG/GA	2	17.5	Intergenic
7	275287	275330	AG/GA	2	22	Intergenic
7	184586	184622	AG/GA	2	18.5	Intergenic
7	208952	209019	AG/GA	2	34	Intergenic
7	53105	53130	CT/TC	2	13	Intergenic
7	640319	640359	CT/TC	2	22	Intergenic
7	722692	722745	CT/TC	2	27	Intergenic
7	822789	822814	CT/TC	2	13	Intergenic
7	252056	252120	GT/TG	2	32.5	Intergenic
7	520190	520237	GT/TG	2	24	Intergenic
7	142198	142232	GT/TG	2	17.5	Intergenic
7	90564	90600	AAC/ACA/CAA	3	12.3	Intergenic
7	190150	190198	AAC/ACA/CAA	3	16.3	Intergenic
7	368233	368260	AAG/AGA/GAA	3	9.3	Intergenic
7	115097	115131	ACC/CCA/CAC	3	11.7	Intergenic
7	103594	103623	ACC/CCA/CAC	3	10	Intergenic
7	51033	51060	AGC/GCA/CAG	3	9.3	Intergenic
7	115974	116001	AGC/GCA/CAG	3	9.3	Intergenic
7	426640	426669	AGC/GCA/CAG	3	10	Intergenic
7	432883	432910	AGC/GCA/CAG	3	9.3	Intergenic
7	60626	60677	AGG/GGA/GAG	3	17.3	Intergenic
7	237693	237731	AGG/GGA/GAG	3	12.7	Intergenic
7	110026	110055	ATG/TGA/GAT	3	10	Intergenic
7	157800	157838	ATG/TGA/GAT	3	13	Intergenic
7	174470	174507	ATG/TGA/GAT	3	12.7	Intergenic
7	417610	417644	ATG/TGA/GAT	3	11.7	Intergenic
7	80385	80410	CCT/CTC/TCC	3	8.7	Intergenic
7	121929	121954	CTT/TTC/TCT	3	8.7	Intergenic
7	47462	47518	CTT/TTC/TCT	3	19	Intergenic
7	104133	104171	CTT/TTC/TCT	3	13	Intergenic
7	164587	164611	CTT/TTC/TCT	3	8.3	Intergenic
7	347366	347403	CTT/TTC/TCT	3	12.7	Intergenic
7	255758	255785	GTT/TTG/TGT	3	9.3	Intergenic
7	546937	546982	GTT/TTG/TGT	3	15.7	Intergenic
7	556615	556655	GTT/TTG/TGT	3	13.7	Intergenic
7	636481	636531	GTT/TTG/TGT	3	17	Intergenic
7	25389	25424	GCT/CTG/TGC	3	11.7	Intergenic
7	161867	161908	GCT/CTG/TGC	3	14	Intergenic
7	161988	162017	GCT/CTG/TGC	3	9.7	Intergenic
7	279298	279322	GGT/GTG/TGG	3	8.3	Intergenic
7	104899	104945	GGT/GTG/TGG	3	15.7	Intergenic
7	221137	221170	GGT/GTG/TGG	3	11.3	Intergenic
7	88127	88155	GGT/GTG/TGG	3	9.7	Intergenic
7	115799	115837	A	1	39	Intron
7	123892	123948	A	1	57	Intron
7	84674	84698	A	1	25	Intron
7	669315	669366	A	1	52	Intron
7	253106	253157	A	1	52	Intron
7	558305	558337	G	1	33	Intron
7	17533	17571	T	1	39	Intron
7	41305	41342	T	1	38	Intron
7	266114	266157	T	1	44	Intron
7	415841	415872	T	1	32	Intron
7	94528	94582	T	1	55	Intron
7	201860	201902	T	1	43	Intron
7	563907	563937	T	1	31	Intron
7	174866	174906	T	1	41	Intron
7	175226	175303	T	1	78	Intron
7	3703	3738	T	1	36	Intron
7	343626	343662	T	1	37	Intron
7	332631	332657	AGC/GCA/CAG	3	9	Intron
7	210836	210866	CTT/TTC/TCT	3	10	Intron
7	624049	624091	GCT/CTG/TGC	3	14.3	Intron
