# Supplementary material for: Simple sequence repeats in Neurospora crassa: distribution, polymorphism and evolutionary inference
Source: BMC Genomics. 2008 Jan 23;9:31. doi: 10.1186/1471-2164-9-31 (PMC2257937; doi:10.1186/1471-2164-9-31)
Supplement: Additional file 4 — The distribution of the randomly selected SSRs by the unit number [file 1471-2164-9-31-S4.pdf]

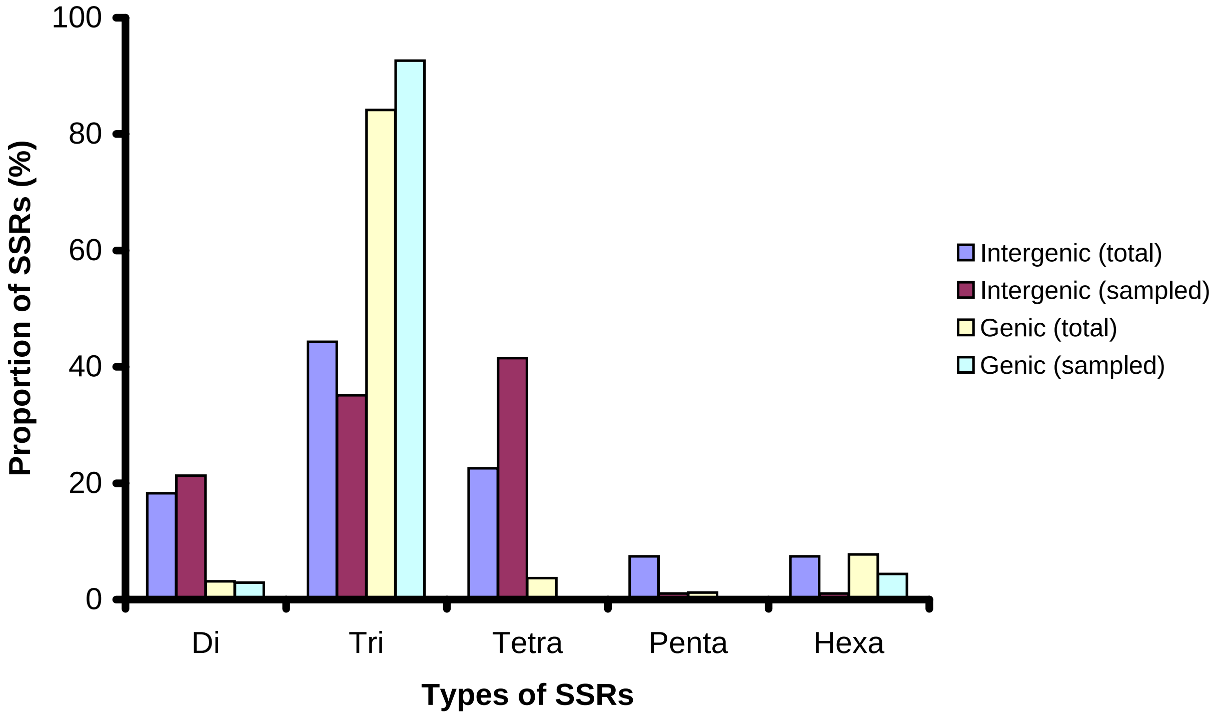

No	Chromosome	Contig Number	Position within contig	Unit Sequence	Repeat Number
1	3	1	221811 ~ 221875	CCT	21.7
2	3	1	346699 ~ 346744	CCTT	11.3
3	3	1	730845 ~ 730888	AGC	14.7
4	3	1	750394 ~ 750428	TTTC	8.8
5	3	1	1024444 ~ 1024480	GGTA	9.3
6	3	1	1272386 ~ 1272428	GTC	14.3
7	3	1	1500560 ~ 1500605	TACA	11.5
8	1	2	277495 ~ 277591	GTT	32.3
9	1	2	380469 ~ 380523	TTCC	13.8
10	1	2	524605 ~ 524640	CTTC	9
11	1	2	882110 ~ 882179	CAACAC	11.7
12	1	2	1056415 ~ 1056474	AAG	20
13	1	2	1270782 ~ 1270927	GTT	48.7
14	1	2	1588956 ~ 1588998	CAA	14.3
15	1	3	232922 ~ 232968	CAG	15.7
16	1	3	343335 ~ 343379	GACT	11.3
17	1	3	652024 ~ 652064	ACG	13.7
18	1	3	896071 ~ 896119	AC	25
19	1	3	1247202 ~ 1247252	AGG	17
20	1	3	1258962 ~ 1259042	TC	40.5
21	6	4	307890 ~ 307925	AC	18
22	6	4	595016 ~ 595060	TTG	15
23	6	4	972152 ~ 972189	TGG	12.7

24	6	4	1022466 ~ 1022511	TTGG	11
25	2	5	170024 ~ 170076	GGT	17.7
26	2	5	424445 ~ 424483	TGC	13
27	2	5	523288 ~ 523331	TCT	14.7
28	2	5	833653 ~ 833728	GTT	25.3
29	1	6	66556 ~ 66597	ACA	14
30	1	6	311750 ~ 311800	GCA	17
31	1	7	70610 ~ 70651	AAGA	10.5
32	1	7	414141 ~ 414186	TCT	15.3
33	1	7	550900 ~ 550949	GAT	16.7
34	1	7	972109 ~ 972173	CAGCAA	10.8
35	2	8	225775 ~ 225814	GGT	13.3
36	2	8	445938 ~ 445986	CAT	16.3
37	2	8	590238 ~ 590281	TGT	14.7
38	2	8	860683 ~ 860717	TAGG	8.8
39	1	9	261776 ~ 261829	ACAT	13.5
40	1	9	679283 ~ 679345	ACA	21
41	1	9	881547 ~ 881586	CCTG	10
42	7	10	174470 ~ 174507	TGA	12.7
43	7	10	252056 ~ 252120	TG	32.5
44	7	10	624049 ~ 624091	CTG	14.3
45	7	10	902607 ~ 902748	AGGT	35.5
46	5	11	160124 ~ 160173	CA	26
47	5	11	320460 ~ 320499	GCCA	10
48	5	11	754219 ~ 754276	ACGA	14.5
49	5	11	764835 ~ 764878	CTC	14.7
50	6	12	79329 ~ 79382	TC	27.5
51	6	12	422999 ~ 423051	GGTA	13.3
52	6	12	634162 ~ 634199	AGC	12.7
53	6	12	825653 ~ 825694	CAC	14
54	5	13	264664 ~ 264705	AGGT	10.3
55	5	13	521169 ~ 521206	ACC	12.7
56	5	13	831848 ~ 831892	CAT	15
57	5	14	166889 ~ 166952	TGTAG	12.8
58	5	14	275448 ~ 275544	AC	48.5
59	5	14	731790 ~ 731824	GAG	11.7
60	6	16	147716 ~ 147751	AC	18
61	6	16	385833 ~ 385870	TGG	12.7
62	6	16	501791 ~ 501828	ACA	12.7
63	3	17	323626 ~ 323662	GTC	12.3
64	4	18	61400 ~ 61434	CCAT	8.8
65	4	18	347280 ~ 347432	GTT	51
66	4	19	481576 ~ 481610	GGT	11.7
67	4	19	534736 ~ 534787	ATAC	13
68	4	20	204217 ~ 204261	AAC	14.7
69	4	20	256193 ~ 256291	TCACCA	16.5
70	4	20	505127 ~ 505208	TGT	27.3
71	7	21	275287 ~ 275330	GA	22
72	7	21	615080 ~ 615259	ACA	60
73	6	22	128438 ~ 128527	CCTA	22.5
74	6	22	536453 ~ 536488	GAAA	9
75	7	23	194233 ~ 194352	TCA	40

76	7	23	343505 ~ 343587	GTA	27.7
77	3	25	25541 ~ 25581	TTC	13.7
78	4	26	171173 ~ 171211	GTT	13
79	4	26	340105 ~ 340154	GGA	16.7
80	3	27	189900 ~ 189944	ATGG	11.3
81	3	27	400773 ~ 400817	GAT	15
82	4	28	138649 ~ 138692	TGT	14.7
83	4	28	407379 ~ 407414	CTG	12
84	1	29	13096 ~ 13146	CTC	17.3
85	2	30	255769 ~ 255808	TTG	13.3
86	7	32	24674 ~ 24717	CTC	14.7
87	7	32	262267 ~ 262346	GGGAAA	13.3
88	3	1	838136 ~ 838171	ACTG	9
89	3	1	1452588 ~ 1452625	TGA	12.7
90	1	2	700491 ~ 700535	CAC	15
91	1	2	1056415 ~ 1056474	AAG	20
92	1	2	1648807 ~ 1648894	TCT	29
93	1	3	711452 ~ 711509	AAAG	14.3
94	6	4	293442 ~ 293472	GTG	10.3
95	6	4	795125 ~ 795174	GAA	16.7
96	2	5	224236 ~ 224278	CAA	14.3
97	2	5	914661 ~ 914699	AC	19.5
98	1	6	200004 ~ 200161	CAA	52.7
99	1	7	636597 ~ 636632	AC	18
100	1	7	823852 ~ 823909	AAGC	14.5
101	2	8	602669 ~ 602703	CAGC	8.8
102	1	9	478497 ~ 478537	CGTT	10.3
103	7	10	174470 ~ 174507	TGA	12.7
104	7	10	722692 ~ 722745	CT	27
105	5	11	225789 ~ 225870	TCT	27.3
106	6	12	218025 ~ 218098	AAG	25
107	6	12	825653 ~ 825694	CAC	14
108	5	13	361740 ~ 361779	TTC	13.3
109	5	14	449098 ~ 449139	GCT	14
110	5	14	564948 ~ 565014	AG	33.5
111	6	16	245477 ~ 245519	GT	21.5
112	4	19	270621 ~ 270662	CAA	14
113	4	19	439820 ~ 439856	CAA	12.3
114	4	20	621911 ~ 621950	AAG	13.3
115	7	21	157800 ~ 157838	TGA	13
116	6	22	23498 ~ 23563	AC	33
117	7	23	430962 ~ 431003	AGC	14
118	5	24	290074 ~ 290135	GGTA	15.5
119	3	25	289671 ~ 289709	AGC	13
120	4	26	237869 ~ 237904	AGC	12
121	3	27	153363 ~ 153415	TGCC	13.3
122	3	27	238013 ~ 238056	AAC	14.7
123	4	28	141787 ~ 141849	CTC	21
124	4	28	407379 ~ 407414	CTG	12
125	1	29	13096 ~ 13146	CTC	17.3
126	7	32	351411 ~ 351449	CAGG	9.8
127	2	33	341828 ~ 341953	TC	63

128	6	34	241860 ~ 241935	TACC	19
129	4	35	134360 ~ 134438	GGAT	19.8
130	4	35	224393 ~ 224431	TG	19.5
131	4	36	260119 ~ 260171	GGA	17.7
132	5	37	223562 ~ 223611	GCA	16.7
133	1	38	157604 ~ 157643	AAGA	10
134	1	39	137780 ~ 137824	TG	22.5
135	3	40	218684 ~ 218749	AG	33
136	5	41	229996 ~ 230091	ACA	32
137	3	42	246781 ~ 246972	AAC	64
138	4	43	5332 ~ 5374	GACT	10.8
139	2	44	116121 ~ 116161	TGT	13.7
140	3	45	236636 ~ 236676	AACG	10.3
141	5	46	132777 ~ 132812	CAA	12
142	4	47	84629 ~ 84670	AGCA	10.8
143	5	48	109113 ~ 109180	ACA	22.7
144	4	51	173992 ~ 174041	CAA	16.7
145	7	52	161554 ~ 161598	CA	22.5
146	2	54	210836 ~ 210872	ACA	12.3
147	1	56	88744 ~ 88785	CTTG	10.5
148	2	57	104129 ~ 104172	CCGA	11
149	1	58	110853 ~ 110887	GACC	8.8
150	4	60	106230 ~ 106293	CA	32
151	1	62	8763 ~ 8813	CCT	17
152	5	63	115653 ~ 115704	CAA	17.3
153	1	65	38240 ~ 38278	GTG	13
154	7	66	71475 ~ 71602	AAC	42.7
155	2	68	34772 ~ 34815	GA	22
156	3	69	83662 ~ 83807	AGA	48.7
157	1	70	67709 ~ 67744	TGTA	9
158	2	71	61452 ~ 61491	GTT	13.3
159	3	74	2479 ~ 2513	TAT	11.7
160	7	75	24819 ~ 24941	AAC	41
161	7	78	46844 ~ 46906	ACA	21
162	4	79	60095 ~ 60140	AAC	15.3
