# Supplementary material for: Simple sequence repeats in Neurospora crassa: distribution, polymorphism and evolutionary inference
Source: BMC Genomics. 2008 Jan 23;9:31. doi: 10.1186/1471-2164-9-31 (PMC2257937; doi:10.1186/1471-2164-9-31)
Supplement: Additional file 5 — The physical location and PIC values of 131 SSR loci in the Neurospora crassa genome [file 1471-2164-9-31-S5.pdf]

No	Name	Chromosome	Contig	Location	Length	Unit	unit number	Repeat #	Allele number[a]	Allele total tested[b]	PIC[c]
1	MN001	3	1	221811 ~ 221875	65	CCT	3	21.7	4	7	0.69
2	MN003	3	1	730845 ~ 730888	44	AGC	3	14.7	5	7	0.78
3	MN007	3	1	500560 ~ 1500605	46	TACA	4	11.5	5	6	0.78
4	MN008	1	2	277495 ~ 277591	97	GTT	3	32.3	5	7	0.78
5	MN009	1	2	380469 ~ 380523	55	TTCC	4	13.8	5	5	0.80
6	MN010	1	2	524605 ~ 524640	36	CTTC	4	9	3	5	0.64
7	MN011	1	2	882110 ~ 882179	70	CAACAC	6	11.7	6	7	0.82
8	MN014	1	2	588956 ~ 1588998	43	CAA	3	14.3	4	4	0.75
9	MN015	1	3	232922 ~ 232968	47	CAG	3	15.7	7	7	0.86
10	MN016	1	3	343335 ~ 343379	45	GACT	4	11.3	5	7	0.78
11	MN017	1	3	652024 ~ 652064	41	ACG	3	13.7	5	6	0.78
12	MN018	1	3	896071 ~ 896119	49	AC	2	25	6	7	0.82
13	MN019	1	3	247202 ~ 1247252	51	AGG	3	17	6	6	0.83
14	MN023	6	4	972152 ~ 972189	38	TGG	3	12.7	5	6	0.78
15	MN024	6	4	022466 ~ 102251	46	TTGG	4	11	4	5	0.72
16	MN026	2	5	424445 ~ 424483	39	TGC	3	13	6	7	0.82
17	MN027	2	5	523288 ~ 523331	44	TCT	3	14.7	5	7	0.73
18	MN028	2	5	833653 ~ 833728	76	GTT	3	25.3	7	7	0.86
19	MN029	1	6	66556 ~ 66597	42	ACA	3	14	3	4	0.63
20	MN030	1	6	311750 ~ 311800	51	GCA	3	17	5	5	0.80
21	MN032	1	7	70610 ~ 70651	42	AAGA	4	10.5	4	5	0.72
22	MN033	1	7	414141 ~ 414186	46	TCT	3	15.3	6	7	0.82
23	MN034	1	7	550900 ~ 550949	50	GAT	3	16.7	6	7	0.82
24	MN035	1	7	972109 ~ 972173	65	CAGCAA	6	10.8	3	3	0.67
25	MN036	2	8	225775 ~ 225814	40	GGT	3	13.3	5	6	0.78
26	MN037	2	8	445938 ~ 445986	49	CAT	3	16.3	7	7	0.86
27	MN038	2	8	590238 ~ 590281	44	TGT	3	14.7	4	5	0.72
28	MN039	2	8	860683 ~ 860717	35	TAGG	4	8.8	3	3	0.67
29	MN041	1	9	261776 ~ 261829	54	ACAT	4	13.5	7	7	0.86
30	MN042	1	9	679283 ~ 679345	63	ACA	3	21	5	5	0.80
31	MN045	7	10	252056 ~ 252120	65	TG	2	32.5	3	3	0.67
32	MN046	7	10	624049 ~ 624091	43	CTG	3	14.3	6	7	0.82
33	MN047	7	10	902607 ~ 902748	142	AGGT	4	35.5	6	7	0.82
34	MN048	5	11	160124 ~ 160173	50	CA	2	26	6	7	0.82
35	MN049	5	11	320460 ~ 320499	40	GCCA	4	10	4	4	0.75
36	MN051	5	11	764835 ~ 764878	44	CTC	3	14.7	7	7	0.86
37	MN052	6	12	79329 ~ 79382	54	TC	2	27.5	3	4	0.63
38	MN053	6	12	422999 ~ 423051	53	GGTA	4	13.3	6	7	0.82
39	MN054	6	12	634162 ~ 634199	38	AGC	3	12.7	6	7	0.82
40	MN057	5	13	264664 ~ 264705	42	AGGT	4	10.3	5	5	0.80
41	MN058	5	13	521169 ~ 521206	38	ACC	3	12.7	6	7	0.82
42	MN059	5	13	831848 ~ 831892	45	CAT	3	15	6	7	0.82
43	MN060	5	14	166889 ~ 166952	64	TGTAG	5	12.8	4	4	0.75
44	MN061	5	14	275448 ~ 275544	97	AC	2	48.5	4	6	0.80
45	MN062	5	14	731790 ~ 731824	35	GAG	3	11.7	5	7	0.73
46	MN065	6	16	147716 ~ 147751	36	AC	2	18	5	6	0.78
47	MN066	6	16	385833 ~ 385870	38	TGG	3	12.7	4	4	0.75
48	MN067	6	16	501791 ~ 501828	38	ACA	3	12.7	6	7	0.82
49	MN068	3	17	323626 ~ 323662	37	GTC	3	12.3	7	7	0.86
50	MN072	4	19	481576 ~ 481610	35	GGT	3	11.7	6	6	0.83
51	MN073	4	19	534736 ~ 534787	52	ATAC	4	13	5	5	0.80
52	MN074	4	20	204217 ~ 204261	45	AAC	3	14.7	5	7	0.78
53	MN075	4	20	256193 ~ 256291	99	TCACCA	6	16.5	6	7	0.82
54	MN076	4	20	505127 ~ 505208	82	TGT	3	27.3	5	5	0.80
55	MN077	7	21	275287 ~ 275330	44	GA	2	22	5	5	0.80
56	MN078	7	21	615080 ~ 615259	180	ACA	3	60	4	6	0.72
57	MN079	6	22	128438 ~ 128527	90	CCTA	4	22.5	6	7	0.82
58	MN080	6	22	536453 ~ 536488	36	GAAA	4	9	7	7	0.86
59	MN081	7	23	194233 ~ 194352	120	TCA	3	40	3	3	0.67
60	MN082	7	23	343505 ~ 343587	83	GTA	3	27.7	4	4	0.75
61	MN084	3	25	25541 ~ 25581	41	TTC	3	13.7	5	7	0.78
62	MN086	4	26	171173 ~ 171211	39	GTT	3	13	5	5	0.80
63	MN087	4	26	340105 ~ 340154	50	GGA	3	16.7	3	3	0.67
64	MN089	3	27	400773 ~ 400817	45	GAT	3	15	6	7	0.82
65	MN090	4	28	138649 ~ 138692	44	TGT	3	14.7	7	7	0.86
66	MN092	1	29	13096 ~ 13146	51	CTC	3	17.3	3	3	0.67
67	MN094	2	30	255769 ~ 255808	40	TTG	3	13.3	5	5	0.80
68	MN095	7	32	24674 ~ 24717	44	CTC	3	14.7	4	4	0.75
69	MN096	7	32	262267 ~ 262346	80	GGGAAA	6	13.3	4	4	0.75
70	MN104	3	1	838136 ~ 838171	36	ACTG	4	9	4	4	0.75

71	MN108	3	1	452588 ~ 1452625	38	TGA	3	12.7	4	6	0.72
72	MN112	1	2	700491 ~ 700535	45	CAC	3	15	5	7	0.78
73	MN114	1	2	056415 ~ 1056474	60	AAG	3	20	4	4	0.75
74	MN116	1	2	648807 ~ 1648894	88	TCT	3	29	3	3	0.67
75	MN117	1	3	711452 ~ 711509	58	AAAG	4	14.3	4	4	0.75
76	MN119	6	4	293442 ~ 293472	31	GTG	3	10.3	4	6	0.72
77	MN121	6	4	795125 ~ 795174	50	GAA	3	16.7	5	7	0.78
78	MN127	2	5	914661 ~ 914699	39	AC	2	19.5	3	5	0.64
79	MN128	1	6	200004 ~ 200161	158	CAA	3	52.7	5	7	0.78
80	MN129	1	7	636597 ~ 636632	36	AC	2	18	5	7	0.64
81	MN131	1	7	823852 ~ 823909	58	AAGC	4	14.5	3	4	0.63
82	MN132	2	8	602669 ~ 602703	35	CAGC	4	8.8	4	5	0.72
83	MN136	1	9	478497 ~ 478537	41	CGTT	4	10.3	6	7	0.82
84	MN142	7	10	722692 ~ 722745	54	CT	2	27	3	3	0.67
85	MN150	5	13	361740 ~ 361779	40	TTC	3	13.3	5	7	0.78
86	MN153	5	14	449098 ~ 449139	42	GCT	3	14	5	5	0.80
87	MN154	5	14	564948 ~ 565014	67	AG	2	33.5	4	4	0.75
88	MN157	6	16	245477 ~ 245519	43	GT	2	21.5	5	7	0.78
89	MN162	4	19	270621 ~ 270662	42	CAA	3	14	6	6	0.83
90	MN164	4	19	439820 ~ 439856	37	CAA	3	12.3	5	5	0.80
91	MN167	4	20	621911 ~ 621950	40	AAG	3	13.3	4	6	0.72
92	MN168	7	21	157800 ~ 157838	39	TGA	3	13	6	6	0.83
93	MN170	7	23	430962 ~ 431003	42	AGC	3	14	5	6	0.78
94	MN171	5	24	290074 ~ 290135	62	GGTA	4	15.5	4	6	0.72
95	MN173	3	25	289671 ~ 289709	39	AGC	3	13	5	7	0.78
96	MN178	3	27	153363 ~ 153415	53	TGCC	4	13.3	4	5	0.72
97	MN179	3	27	238013 ~ 238056	44	AAC	3	14.7	5	5	0.80
98	MN182	4	28	141787 ~ 141849	63	CTC	3	21	6	6	0.83
99	MN184	1	29	13096 ~ 13146	51	CTC	3	17.3	5	5	0.80
100	MN186	7	32	351411 ~ 351449	39	CAGG	4	9.8	6	7	0.82
101	MN188	2	33	341828 ~ 341953	126	TC	2	63	3	3	0.67
102	MN191	4	35	134360 ~ 134438	79	GGAT	4	19.8	4	4	0.75
103	MN192	4	35	224393 ~ 224431	39	TG	2	19.5	6	6	0.83
104	MN194	4	36	260119 ~ 260171	53	GGA	3	17.7	4	6	0.72
105	MN196	5	37	223562 ~ 223611	50	GCA	3	16.7	4	5	0.72
106	MN197	1	38	157604 ~ 157643	40	AAGA	4	10	6	7	0.82
107	MN199	1	39	137780 ~ 137824	45	TG	2	22.5	5	5	0.80
108	MN201	3	40	218684 ~ 218749	66	AG	2	33	7	7	0.83
109	MN203	5	41	229996 ~ 230091	96	ACA	3	32	7	7	0.83
110	MN205	3	42	246781 ~ 246972	192	AAC	3	64	3	3	0.67
111	MN208	2	44	116121 ~ 116161	41	TGT	3	13.7	5	7	0.78
112	MN213	5	46	132777 ~ 132812	36	CAA	3	12	4	4	0.75
113	MN215	4	47	84629 ~ 84670	42	AGCA	4	10.8	5	7	0.78
114	MN220	4	51	173992 ~ 174041	50	CAA	3	16.7	4	7	0.69
115	MN225	2	54	210836 ~ 210872	37	ACA	3	12.3	5	7	0.78
116	MN227	1	56	88744 ~ 88785	42	CTTG	4	10.5	5	6	0.78
117	MN229	2	57	104129 ~ 104172	44	CCGA	4	11	5	7	0.73
118	MN231	1	58	110853 ~ 110887	35	GACC	4	8.8	4	7	0.69
119	MN234	1	62	8763 ~ 8813	51	CCT	3	17	3	4	0.63
120	MN236	5	63	115653 ~ 115704	52	CAA	3	17.3	3	4	0.63
121	MN239	1	65	38240 ~ 38278	39	GTG	3	13	5	7	0.73
122	MN240	7	66	71475 ~ 71602	128	AAC	3	42.7	5	5	0.80
123	MN241	2	68	34772 ~ 34815	44	GA	2	22	5	5	0.80
124	MN242	3	69	83662 ~ 83807	146	AGA	3	48.7	4	4	0.75
125	MN243	1	70	67709 ~ 67744	36	TGTA	4	9	3	4	0.63
126	MN245	3	74	2479 ~ 2513	35	TAT	3	11.7	3	5	0.64
127	MN246	7	75	24819 ~ 24941	123	AAC	3	41	3	3	0.67
128	MN248	7	78	46844 ~ 46906	63	ACA	3	21	5	5	0.80
129	MN249	4	79	60095 ~ 60140	46	AAC	3	15.3	6	4	0.72
											0.76

[a], The allele number refer to the number of different allele in a given SSR locus.

[b], The total allele number refer to the tested allele from the possible 7 alleles in a SSR locus.

[c], PIC stands for polymorphism information contents (see method)

[d], NA stands for Not acquired
