# Supplementary material for: Simple sequence repeats in Neurospora crassa: distribution, polymorphism and evolutionary inference
Source: BMC Genomics. 2008 Jan 23;9:31. doi: 10.1186/1471-2164-9-31 (PMC2257937; doi:10.1186/1471-2164-9-31)
Supplement: Additional file 6 — Comparison of PIC values of the AC/CA SSR type in two different population sizes [file 1471-2164-9-31-S6.pdf]

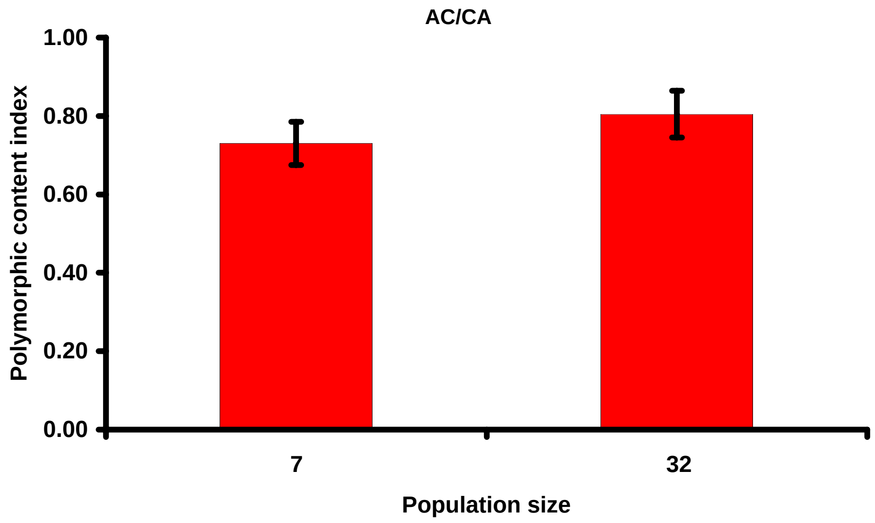

ID	chromosome	contig	position	length	SSR type	unit number	repeat number	PIC from 7 strains	PIC from 32 strains
MNSSR061	5	14	275448 ~ 275544	97	AC	2	48.5	0.80	0.91
MNSSR065	6	16	147716 ~ 147751	36	AC	2	18	0.75	0.78
MNSSR129	1	7	636597 ~ 636632	36	AC	2	18	0.64	0.73
							average	0.73	0.80
							standard error	0.055	0.060
