# Supplementary figures and images for: Simple sequence repeats in Neurospora crassa: distribution, polymorphism and evolutionary inference
Source: BMC Genomics. 2008 Jan 23;9:31. doi: 10.1186/1471-2164-9-31 (PMC2257937; doi:10.1186/1471-2164-9-31)

Hypothesis #1

Hypothesis #2

Strain #1

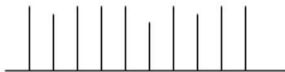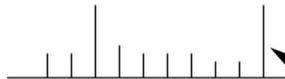

Strain #2

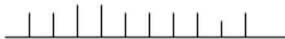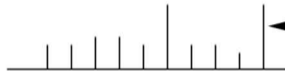

# of repeats  
in a SSR

Strain #3

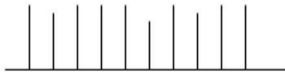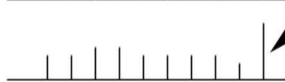

Hypothesis #3

Supplement: Additional file 7 — Three hypotheses for the size variation of SSRs [file 1471-2164-9-31-S7.pdf]
