# Supplementary material for: Simple sequence repeats in Neurospora crassa: distribution, polymorphism and evolutionary inference
Source: BMC Genomics. 2008 Jan 23;9:31. doi: 10.1186/1471-2164-9-31 (PMC2257937; doi:10.1186/1471-2164-9-31)
Supplement: Additional file 8 — The list of 33 SSR loci for statistical analyses [file 1471-2164-9-31-S8.pdf]

Marker number	4825	2223	4720	4715	4724	3223	2489	chromosome	genomic region (genic vs intergenic)	genomic region (exon vs intron vs intergenic)	**unit size**	SSR type
1	10	4	3	3	2	2	22	3	genic	Exon	3	cct/ctc/tcc
3	10	15	13	14	4	15	15	3	Intergenic	Intergenic	3	agc/gca/cag
11	15	13	8	10	8	14	12	1	Intergenic	Intergenic	6	caacac/aacacc/acacca/caccaa/accaac/ccaaca
18	20	28	13	10	11	24	25	1	Intergenic	Intergenic	2	ac/ca
26	7	14	12	16	8	14	13	2	Intergenic	Intergenic	3	tgc/gct/ctg
27	10	12	10	10	8	14	15	2	genic	Exon	3	tct/ctt/ttc
28	10	9	22	11	7	9	25	2	genic	Intron	3	gtt/ttg/tgt
33	19	17	15	17	8	18	15	1	Intergenic	Intergenic	3	tct/ctt/ttc
34	11	10	13	10	3	31	17	1	Intergenic	Intergenic	3	gat/atg/tga
37	14	11	10	12	5	17	16	2	genic	Intron	3	cat/atc/tca
41	9	23	15	12	10	10	14	1	Intergenic	Intergenic	4	acat/cata/atac/taca
46	17	12	12	14	11	12	14	7	genic	Intron	3	ctg/tgc/gct
48	14	16	67	65	12	14	26	5	Intergenic	Intergenic	2	ac/ca
53	9	12	9	15	8	17	13	6	Intergenic	Intergenic	4	ggta/gtag/tagg/aggt
54	15	11	3	6	4	13	13	6	genic	Exon	3	agc/gca/cag
59	17	14	4	9	8	9	15	5	Intergenic	Intergenic	3	cat/atc/tca
67	9	10	8	15	6	7	13	6	Intergenic	Intergenic	3	aac/aca/caa
68	10	10	13	12	19	9	12	3	Intergenic	Intergenic	3	gtc/tcg/cgt
121	15	12	12	12	6	13	17	6	genic	Exon	3	gaa/aag/aga
125	7	5	5	7	5	14	14	2	genic	Exon	3	aac/aca/caa
129	10	9	5	13	8	9	18	1	Intergenic	Intergenic	2	ac/ca
136	6	11	5	13	10	11	10	1	non genic	Intergenic	4	cgtt/gtta/ttag/tagt
150	8	11	4	6	6	11	13	5	genic	Exon	3	tct/ctt/ttc
157	21	25	15	17	22	21	22	6	genic	Intron	2	gt/tg
173	9	13	9	6	9	14	13	3	Intergenic	Intergenic	3	agc/gca/cag
201	14	18	13	12	53	58	33	3	genic	Intron	2	ag/ga
203	12	7	6	10	9	15	32	5	genic	Exon	3	aac/aca/caa
215	14	13	8	8	13	14	11	4	Intergenic	Intergenic	4	agca/gcaa/caag/aagc
220	7	6	6	6	8	6	17	4	genic	Exon	3	aac/aca/caa
225	5	9	5	15	7	9	12	2	genic	Exon	3	aac/aca/caa
229	9	9	8	9	13	7	11	2	Intergenic	Intergenic	4	ggca/gcag/cagg/aggc
239	12	16	14	18	16	16	14	1	genic	Exon	3	gtt/ttg/tgt
250	19	12	5	8	4	6	12	5	genic	Intron	3	ctg/tgc/gct
