# Supplementary material for: Simple sequence repeats in Neurospora crassa: distribution, polymorphism and evolutionary inference
Source: BMC Genomics. 2008 Jan 23;9:31. doi: 10.1186/1471-2164-9-31 (PMC2257937; doi:10.1186/1471-2164-9-31)
Supplement: Additional file 9 — The marker quality of the mapped SSR loci [file 1471-2164-9-31-S9.pdf]

Population	Chromosome	SSR Locus	Contig	Number of progenies tested[a]	Proprotion of progenies tested[b]	χ^2value	p value[c]
n4	3	MN003	1	175	93%	4.17	0.01<p<0.05
n4	1	MN010	2	139	74%	28.55	<0.001
n6	1	MN013	2	175	93%	10.57	<0.001
n6	1	MN015	3	166	88%	11.66	<0.001
n2	1	MN017	3	138	73%	10.46	< 0.001
n6	1	MN018	3	154	82%	14.96	<0.001
n4	1	MN019	3	154	82%	10.39	<0.001
n2	2	MN027	5	135	72%	41.67	<0.001
n6	1	MN128	6	184	98%	33.07	<0.001
n6	1	MN035	7	160	85%	13.23	<0.001
n2	1	MN129	7	166	88%	16.29	<0.001
n6	1	MN129	7	179	95%	16.90	<0.001
n4	2	MN037	8	162	86%	5.56	0.01<p<0.05
n6	2	MN037	8	177	94%	10.45	<0.001
n6	2	MN038	8	167	89%	11.07	<0.001
n6	1	MN042	9	167	89%	31.91	<0.001
n6	1	MN136	9	155	82%	6.20	0.01<p<0.05
n6	5	MN051	11	175	93%	25.65	<0.001
n4	5	MN150	11	159	85%	6.85	0.001<p<0.01
n6	6	MN053	12	165	88%	7.42	0.001<p<0.01
n6	6	MN054	12	151	80%	10.07	0.001<p<0.01
n4	5	MN059	13	169	90%	6.44	0.01<p<0.05
n6	5	MN155	15	184	98%	7.85	0.001<p<0.01
n2	6	MN067	16	185	98%	50.86	<0.001
n6	6	MN157	16	161	86%	9.45	0.001<p<0.01
n4	4	MN074	20	166	88%	29.52	<0.001
n4	4	MN075	20	153	81%	6.28	0.01<p<0.05
n4	4	MN167	20	141	75%	15.67	<0.001
n6	7	MN078	21	183	97%	10.10	0.001<p<0.01
n2	7	MN168	21	130	69%	7.88	0.001<p<0.01
n6	6	MN080	22	169	90%	6.44	0.01<p<0.05
n2	7	MN082	23	133	71%	8.19	0.001<p<0.01
n6	5	MN083	24	167	89%	6.52	0.01<p<0.05
n4	4	MN086	26	180	96%	28.80	<0.001
n6	3	MN089	27	165	88%	11.21	<0.001
n4	4	MN090	28	141	75%	24.69	<0.001
n6	4	MN191	35	181	96%	14.37	<0.001
n2	1	MN199	39	182	97%	12.66	< 0.001
n2	4	MN215	47	123	65%	61.54	<0.001
n2	4	MN220	51	137	73%	11.10	<0.001
n6	4	MN220	51	171	91%	16.43	<0.001
n6	4	MN223	53	170	90%	15.91	<0.001
n4	5	MN236	63	134	71%	28.69	<0.001
n6	5	MN236	63	183	97%	23.09	<0.001
n6	7	MN247	76	164	87%	7.05	0.001<p<0.01
n6	5	MN250	80	166	88%	16.29	<0.001

[a] The number progenies genotyped in each SSR locus.

[b] # of genotypes progeny / # of total progeny (188) * 100

[c] The p values were deteremined based on χ2 distribution in df=1. In df=1, p =0.05 is equal to 3.84 in χ2 value and p=0.01 is 6.65, p=0.001 is 10.8 respectively.

The availabilities of the polymorphic markers are predominately population specific and the mapped SSR loci varied in the different mapping populations. Of the 109 mapped loci, only 17 loci (13% of the mapped loci) were mapped into all the mapping populations and 47 markers were common in at least two mapping populations. About 18%-34% of the SSRs depending on the population showed significant segregation distortion. We detected 7 different genomic regions where the segregation distortions were observed in at least two populations. Especially, the region covering contig 7 at chromosome 1R consistently revealed the deviated segregations in the mapped SSR loci (six SSR loci out of 8 SSR loci). The total genetic length and loci density of the three genetic maps are summarized in Table 5. The total genetic distances varied in the three line-cross populations; N2 cross, 547.1 cM with an average marker distance (amd) of 13.0 cM; N4 cross, 882.7 cM with an amd of 13.0 cM; N6 cross, 934.8 cM with an amd of 13.7 cM.
